# Triethylamine-Catalyzed Cyclization of Unsaturated Hydroperoxides in the Presence of Triethylammonium Hydrochloride: A Synthesis of 1,2-Dioxanes

**DOI:** 10.1021/acs.orglett.4c04629

**Published:** 2025-02-26

**Authors:** John P. Stasiak, K. A. Woerpel

**Affiliations:** Department of Chemistry, New York University, New York, New York 10003, United States

## Abstract

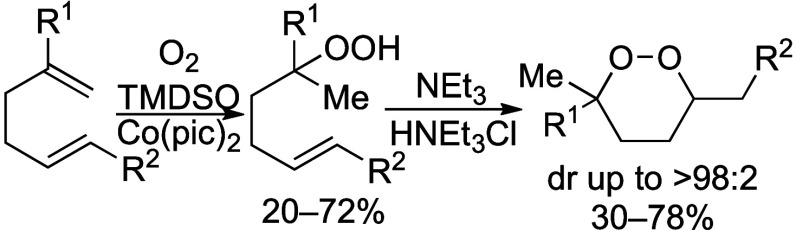

Unsaturated hydroperoxides were synthesized from dienes using a regioselective cobalt-catalyzed hydroperoxidation reaction. Subsequent intramolecular oxa-Michael reactions in the presence of triethylammonium hydrochloride (HNEt_3_Cl) and catalytic Et_3_N formed 1,2-dioxanes, in several cases with high diastereoselectivity. These 1,2-dioxanes could be transformed to their respective carboxylic acids, without affecting the integrity of the peroxide linkage, to form compounds with structures that resemble biologically active natural products.

The six-membered-ring cyclic peroxide (1,2-dioxane) motif is found in many natural products^1^ and small molecules with antimalarial,^2,3^ anticancer,^4^ and antifungal^5^ activity, as illustrated by peroxyplakoric acid A_1_ methyl ester (**1**),^6^ ethyl plakortide Z (**2**),^5^ stolonoxide E (**3**),^7^ and diacarnoxide B (**4**)^8,9^ (Figure 1). Because of the biological activity of these compounds, efforts have been made to develop methods for their synthesis.^10−12^

**Figure 1:**
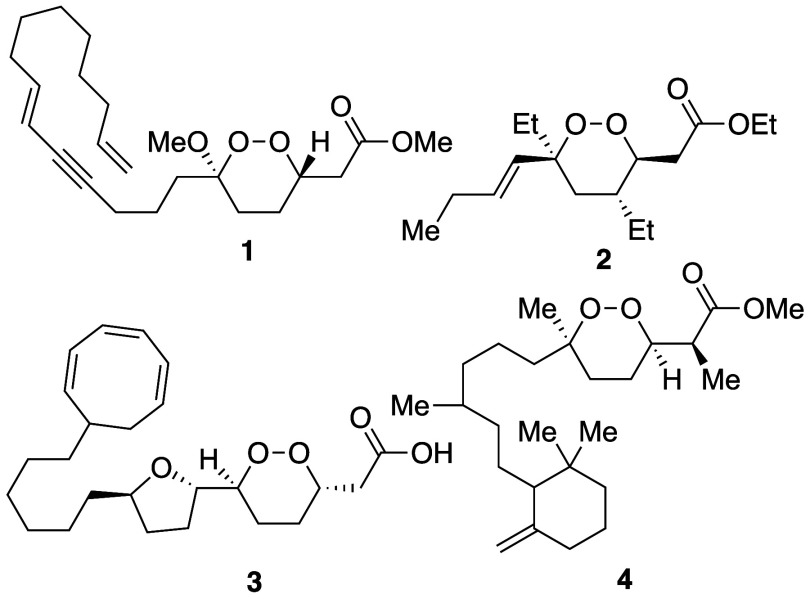
Natural and biologically active 1,2-dioxanes.

We envisioned that 1,2-dioxanes could be synthesized^13,14^ in two steps from readily available dienes **5** (Scheme 1). This route presented two challenges, however. The regioselective peroxidation of a compound containing two different C=C linkages was not assured,^15^ and cyclizations of intermediate peroxyl radicals would form undesired products.^16^ Even if this reaction could be made regioselective and chemoselective, the base-mediated cyclization of an OOH group onto a tethered enoate^6,10,11^ is not typically high-yielding because the enolate intermediate that would be formed^17^ can attack the peroxide group to form an epoxide.^18^

**Scheme 1:**
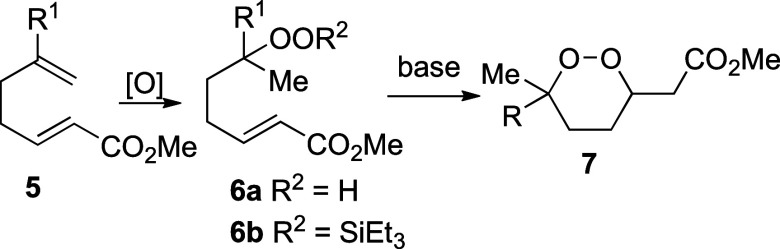
Synthetic Pathway for Synthesis of 1,2-Dioxanes

In this Letter, we report a synthesis of 1,2-dioxanes by a regioselective peroxidation of a compound containing two alkene moieties followed by an intramolecular oxa-Michael addition reaction. Hydroperoxidation with oxygen and HMe_2_Si–O–SiMe_2_H (TMDSO) catalyzed by cobalt(II) picolinate (Co(pic)_2_) occurred regioselectively. Cyclization reactions under buffered acidic conditions limited the amount of side-products, forming the desired 1,2-dioxanes with high diastereoselectivity in several cases. Subsequent derivatization of these 1,2-dioxanes furnished carboxylic acids whose structures mimic natural products (Figure 1).

Initial efforts began with the optimization of the regioselective peroxidation using diene **8**, which was used as a mixture of regioisomers and diastereomers. Peroxidation using catalysts **10**–**13** (Scheme 2) under standard conditions gave complex mixtures of peroxide products (Table 1, entries 1–4). No resonances were detected by NMR spectroscopy that could be attributed to alkenes or to monoperoxides **9a** or **9aa**. By contrast, subjecting dienes **8aa**, **8ab**, and **8ac** to standard hydroperoxidation conditions using cobalt(II) 5,10,15,20-tetraphenylporphine (Co^II^(tpp))^19^ (**14**) and Co(pic)_2_^20^ (**15**) formed hydroperoxide **9a** (Table 1, entries 5–7), although small amounts (15%) of products were also formed where peroxidation had occurred at both C=C double bonds. The reaction with Co(pic)_2_ (**15**) was optimal because cobalt-containing materials could be removed from the unpurified reaction mixture by filtration through a small pad of silica gel (eq 1).
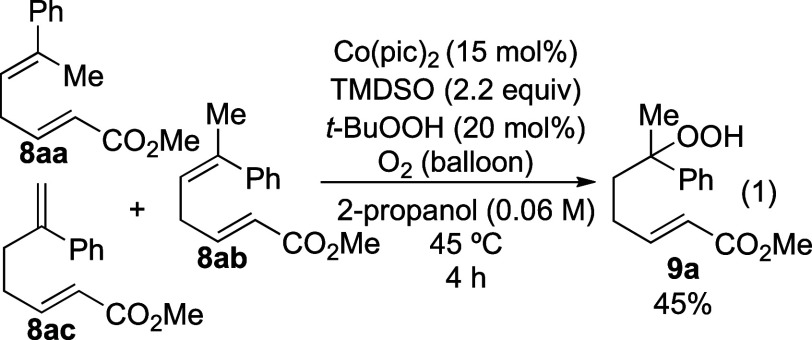
1

**Scheme 2:**
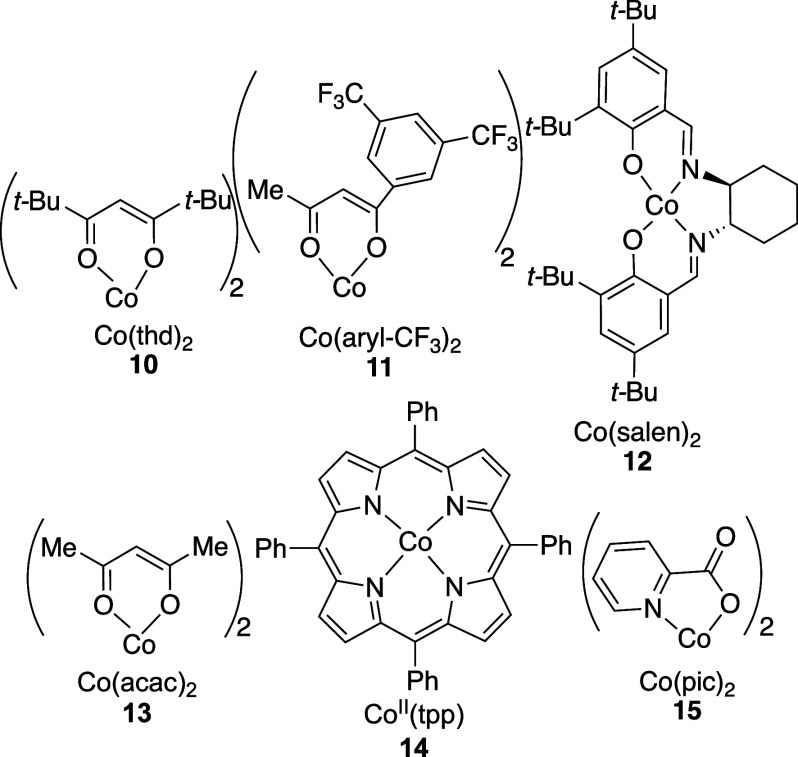
Catalysts Employed

**Table 1:**
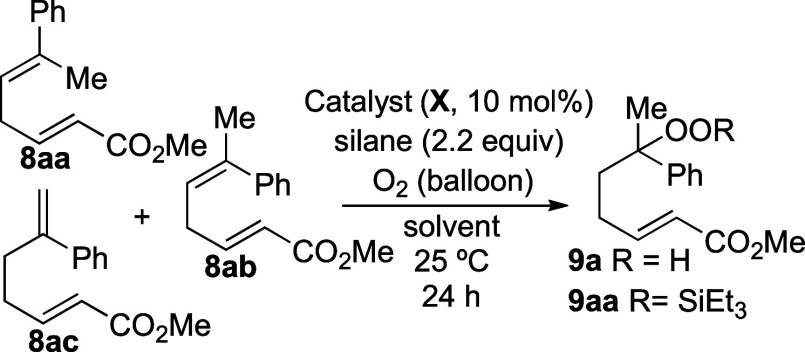
Optimization of Peroxidation

entry	catalyst	silane	solvent	conv (%)	product
1	**10**	SiEt_3_H	ClCH_2_CH_2_Cl	100	–
2	**11**	SiEt_3_H	ClCH_2_CH_2_Cl	100	–
3	**12**	SiEt_3_H	ClCH_2_CH_2_Cl	50	–
4	**13**	SiEt_3_H	ClCH_2_CH_2_Cl	100	–
5^a^	**14**^b^	SiEt_3_H	*i*-PrOH:CH_2_Cl_2_	30	**9a**
6	**14**	SiEt_3_H	*i*-PrOH:CH_2_Cl_2_	100	**9a**
7	**15**^c^	TMDSO	*i*-PrOH	100	**9a**

a 1.1 equiv of silane was used.

b 0.0010 mol % of catalyst was used.

c 5.0 mol % of catalyst was used.

With conditions that provided unsaturated hydroperoxide **9a**, it was then necessary to establish conditions for the cyclization step. The use of basic conditions that had been reported previously (Table 2)^10,11,17^ did result in formation of product **16a**, but the epoxide **17**,^17^ which was presumably formed from the β-peroxy enolate **18** (eq 2), was also formed. The use of *n*-Bu_4_NF, which had been successful in reactions in the case of unsaturated lactones,^21^ was also ineffective (Table 2, entry 4). The yield of the desired product could not be optimized beyond 59% under these conditions.
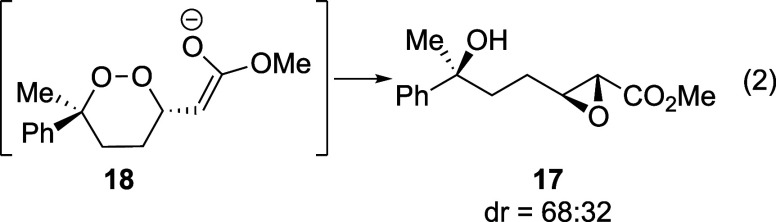
2

**Table 2:**
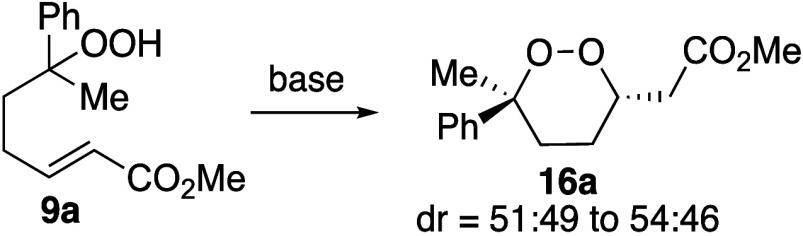
Cyclization of Unsaturated Hydroperoxide **9a** Using Known Conditions

entry	base (equiv)	solvent	NMR yield (%)^a^	time (h)
1	Et_2_NH (0.06)	CF_3_CH_2_OH	26	16
2	Et_2_NH (0.07)	CF_3_CH_2_OH:CH_2_Cl_2_	59	16
3	CsOH (6)	(F_3_C)_2_CHOH:MeOH	29	72
4	*n*-Bu_4_NF (2.4)	CF_3_CH_2_OH	0	24

a Mesitylene was used as an internal standard.

b Calculated from one-pulse ^1^H NMR spectra.

To avoid the formation of epoxide **17**, the β-peroxy enolate **18** would need to be trapped before it could break the O–O bond. At first, LiCl was added in attempts to trap the enolate (Table 3, entry 1). Instead of using basic conditions, use of a Lewis acidic metal salt and mildly acidic conditions, HNEt_3_Cl buffered with Et_3_N,^22^ gave better yields (Table 3, entries 2–3). The use of the polar protic solvent^11,23,24^ trifluoroethanol gave the highest yields (Table 3, entries 3–4), likely because the conditions solvated the anion, preventing formation of the epoxide.^25−27^ Under these conditions, the presence of lithium chloride was not required (Table 3, entry 4). Adjusting the amount of NEt_3_ and HNEt_3_Cl led to optimal yields. Excess HNEt_3_Cl was necessary to obtain higher isolated yields of **16a**, suggesting that this ammonium salt protonates the enolate intermediate^11^ to minimize the formation of epoxide **17** (as illustrated in Table 3, entries 4–5). Using stoichiometric and catalytic amounts of Et_3_N gave comparable yields, highlighting the more important role played by HNEt_3_Cl than Et_3_N. The optimized conditions are shown in eq 3.
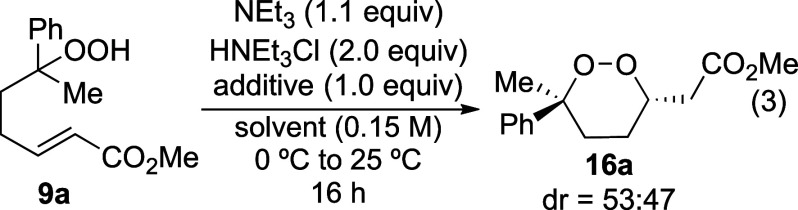
3

**Table 3:** Optimization of Cyclization Conditions using **9a**

entry	additive	solvent	NMR yield (%)^a^	dr^b^
1	LiCl	CH_2_Cl_2_	51	65:35
2	LiCl	(CF_3_)_2_HCOH	51	61:39
3	LiCl	CF_3_CH_2_OH	69	57:43
4	_	CF_3_CH_2_OH	80	53:47
5^c^	_	CF_3_CH_2_OH	17	52:48

a Mesitylene was used as an internal standard.

b Calculated from one-pulse ^1^H NMR spectra.

c 0.3 equiv of HNEt_3_Cl was used.

The optimized hydroperoxidation and cyclization reactions were general for the synthesis of 1,2-dioxanes (Scheme 3). Substitution on the terminal double bond in diene substrates did not affect regioselectivity, as seen by comparing reactions to form unsaturated hydroperoxides **9d** and *trans*-**9g**. This result showcases the high regioselectivity of hydrometalation of alkenes by Co(pic)_2_-derived metal hydrides^28^ compared to hydrides derived from cobalt(II) porphyrin complexes, which reacted with electron-deficient conjugated dienes more efficiently.^15,19,20^ The relative configuration of the enoate did not affect the regioselectivity of hydroperoxidation. Lower yields for the hydroperoxidation reactions to form hydroperoxides **9e** and **9i** reflect the sensitivity of the reaction to steric effects. In these cases, higher quantities (50%) of bis(hydroperoxides) were formed.

**Scheme 3:**
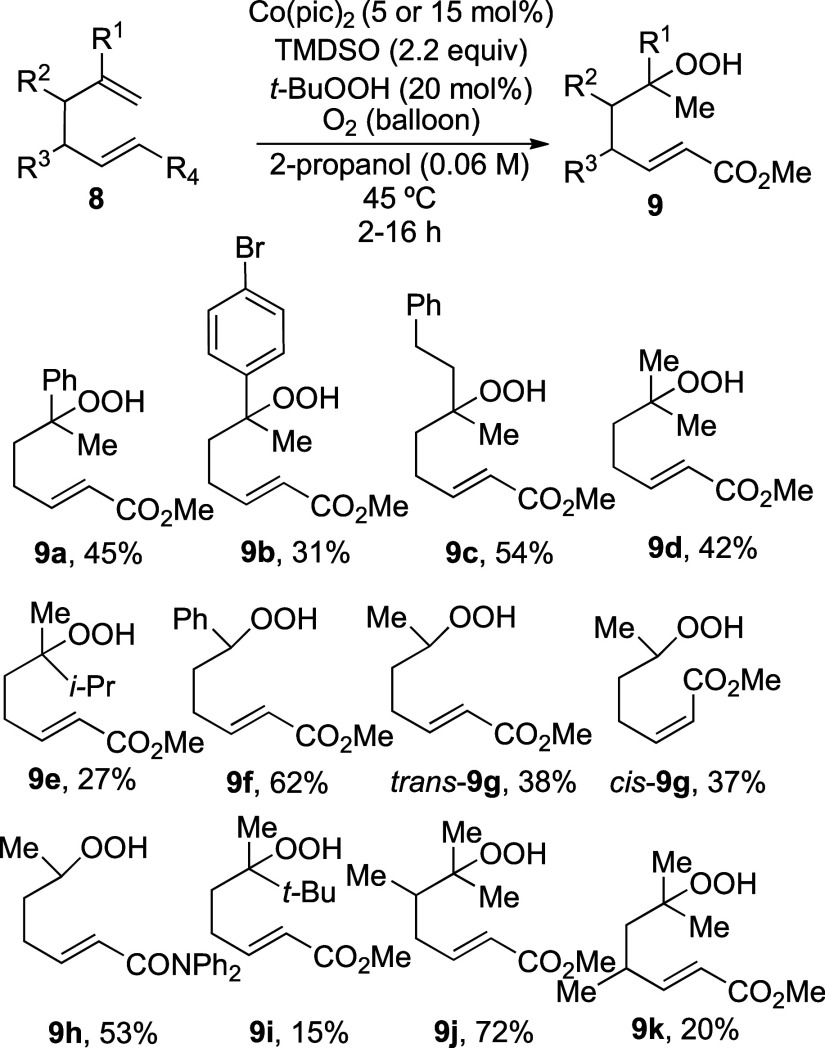
Synthesis of Unsaturated Hydroperoxides

The cyclization of the hydroperoxides formed in Scheme 3 occurred in 30–78% yield under the optimized conditions (Scheme 4). 1,2-Dioxanes **16a**–**c** and **16e** were formed as a mixture of diastereomers, possibly due to the similar sizes of the groups at the geminally substituted carbon atom.^29^ When the groups near the OOH group were sterically different in size, however, as illustrated by formation of 1,2-dioxane **16f**, the product was formed as a single diastereomer, whose stereochemistry was determined by X-ray crystallographic analysis. Similar high stereoselectivity was observed for the formation of 1,2-dioxanes **16g**, **16h**, and **16i**. Formation of 1,2-dioxane **16h** required longer reaction times, possibly due to the decrease in the electrophilicity of an enamide when compared to an enoate.^28^ The observed 1,2-*trans* and 1,4-*trans* stereoselectivity of these reactions is likely due to the preference for substituents at C-2 or C-4 to be equatorial in the transition state leading to the product (Figure 2).^30^ An analogous argument would explain the 1,3-*cis* stereoselectivity seen in dioxane **16j** with the same preference for the substituent to be equatorial in the transition state. The 1,2-*trans* stereochemical relationship is the same as found in ethyl plakortide Z (**2**, Figure 1).^31^

**Scheme 4:**
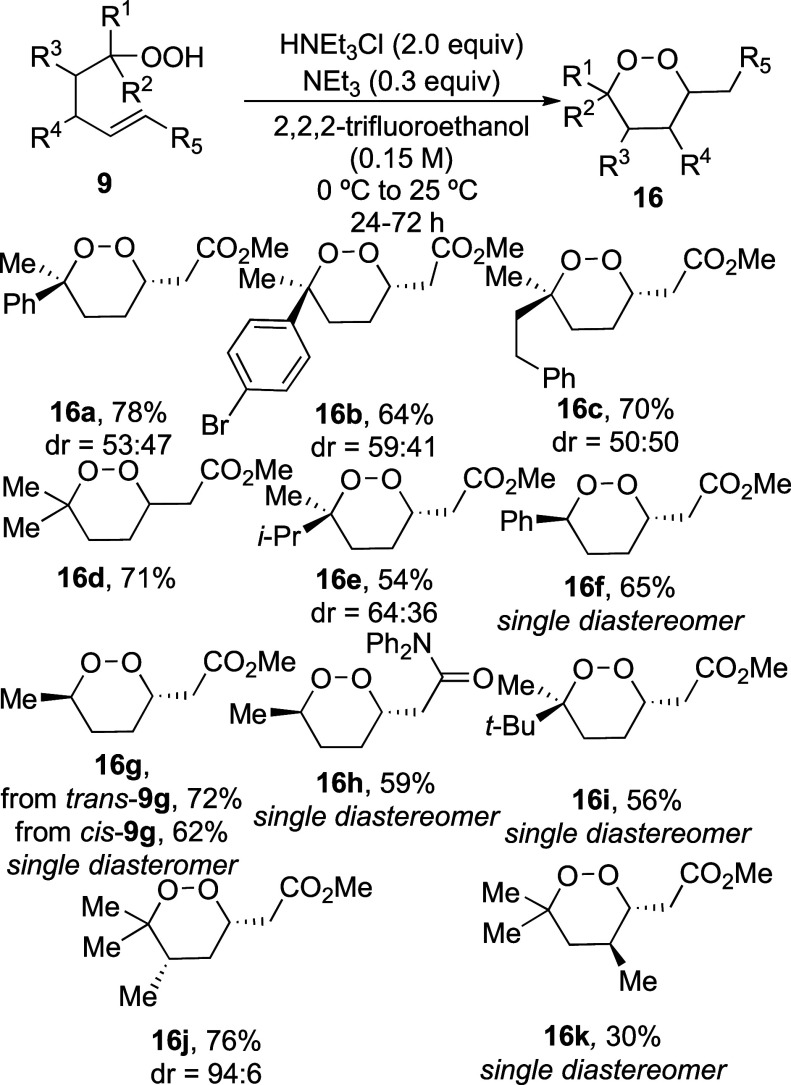
Substrate Scope of 1,2-Dioxane Synthesis

**Figure 2:**
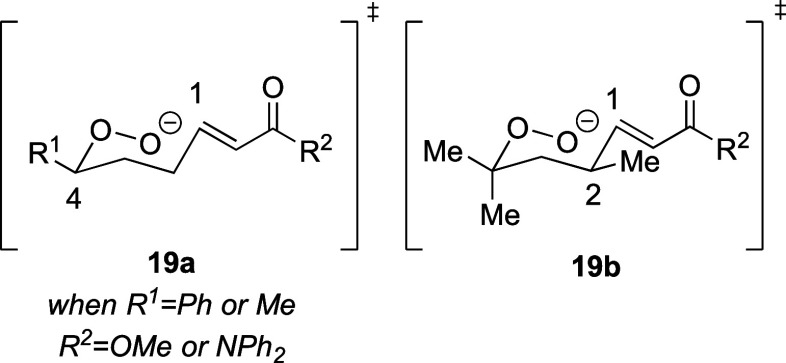
Origin of 1,4-diastereoselectivity.

Because many 1,2-dioxane natural products contain a free CO_2_H group in one of the substituents,^1,32^ it was important to optimize conditions to reveal this group in the presence of the endoperoxide group. Subjecting 1,2-dioxane **16a** to base-mediated hydrolysis conditions with lithium hydroxide^33^ led to epoxide products. This result is consistent with the optimization studies, which indicate that basic conditions can form an enolate intermediate,^11^ leading to epoxide formation. To circumvent this decomposition pathway, methyl ester **16a** was instead reduced to an aldehyde intermediate using diisobutylaluminum hydride, which did not reduce the peroxide moiety.^34,35^ The aldehyde intermediate was then oxidized^36^ to form carboxylic acid **20a** (Scheme 5). These conditions formed a number of 1,2-dioxanes that resemble natural products such as **3**.

**Scheme 5:**
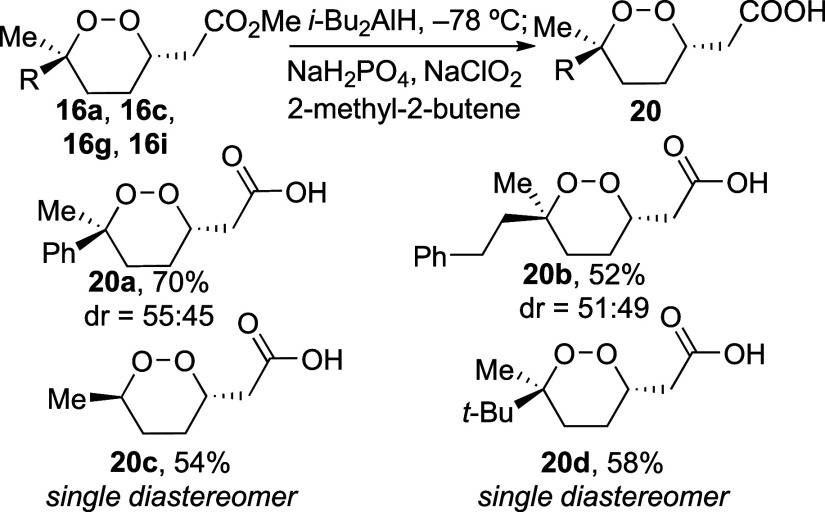
Synthesis of Carboxylic Acid Derivatives of 1,2-Dioxanes

In conclusion, nonconjugated dienes containing various functional groups can be transformed to unsaturated hydroperoxides regioselectively using Co(pic)_2_ as the catalyst. These unsaturated hydroperoxides underwent acid-catalyzed cyclization using triethylammonium hydrochloride and catalytic triethylamine in 2,2,2-trifluoroethanol to afford 1,2-dioxanes in good yield and diastereoselectively in cases where substituents were sterically differentiated.

## Data Availability

The data underlying this study are available in the published article and its Supporting Information.

## Supporting Information Available

Detailed experimental procedures, X-ray data, stereochemical proofs, and NMR spectra of all compounds (PDF)
