# Supplementary material for: Triethylamine-Catalyzed Cyclization of Unsaturated Hydroperoxides in the Presence of Triethylammonium Hydrochloride: A Synthesis of 1,2-Dioxanes
Source: Org Lett. 2025 Feb 26;27(9):2037–41. doi: 10.1021/acs.orglett.4c04629 (PMC11894669; doi:10.1021/acs.orglett.4c04629)
Supplement: Supplementary file 1 — ol4c04629_si_001.pdf [file ol4c04629_si_001.pdf]

Supporting Information for

Triethylamine-Catalyzed Cyclization of Unsaturated Hydroperoxides in the Presence of Triethylammonium Hydrochloride: A Synthesis of 1,2-Dioxanes

John P. Stasiak, K. A. Woerpel*

Department of Chemistry, New York University, New York, New York 10003, United States
*Email: K.Woerpel@nyu.edu

Table of Contents

I. General Experimental……………………………………………………………S2
II. Substrate Synthesis……………………………………...………………...S2-S23
III. Synthesis of Cobalt Catalysts…………………………………...……………..S23-S24
IV. Synthesis of Unsaturated Hydroperoxides………….…………….………..S24-S30
V. Synthesis of 1,2-Dioxanes……………………………………………………..…S30-S39
VI. Stereochemical Correlations and Proofs………………………......……..S39-S41
VII. Crystallographic Data of Compounds **16f** & **16h**……………………...…...…S41-S43
VIII. References…………………………………………………………………..S44-S45
IX. Spectra Of Substrates……………………………………………………..…S46-S186

I. General Experimental

^{1}H NMR and ^{13}C NMR spectra were measured at ambient temperature (unless otherwise noted) using Bruker AV400 (400 MHz and 100 MHz, respectively) and Bruker AVIII-400 (400 MHz and 100 MHz, respectively) spectrometers unless otherwise noted. All spectroscopic data were reported as follows: chemical shifts in ppm on the δ scale referenced from residual solvent peaks (^{1}H NMR: $CDCl_3$ δ 7.26 ppm, C_6D_6 δ 7.16 ppm, $(CD_3)_2SO)$ δ 2.50 ppm; ^{13}C NMR: $CDCl_3$ δ 77.16 ppm, C_6D_6 δ 128.06 ppm, $(CD_3)_2SO)$ δ 39.52 ppm), multiplicity (s = singlet, br = broad, d = doublet, t = triplet, q = quartet, sept = septet, m = multiplet, AB = AB system), coupling constants (Hz), and integration.[1] $^{19}F\{^{1}H\}$ NMR spectra were externally referenced to *α,α,α*-trifluorotoluene ($^{19}F\{^{1}H\}$ NMR: $CDCl_3$ δ −63.72). Multiplicity of carbon peaks were determined using HSQC and/or ^{13}C DEPT experiments. Ratios of products were determined by ^{13}C NMR spectroscopic experiments[2] and confirmed by ^{1}H NMR spectroscopic experiments. (IR) spectra were acquired by Nicolet 6700 FT-IR spectrometer through attenuated total reflectance (ATR). High-resolution mass spectra (HRMS) were recorded using an Agilent 6224 Accurate-Mass time-of-flight spectrometer with atmospheric pressure chemical ionization (APCI) or electrospray ionization (ESI) ionization sources. Analytical thin layer chromatography was performed on silica get 60 Å F254 plates. All reactions were performed under a nitrogen atmosphere in glassware that had been flame-dried under vacuum unless otherwise noted. Non-deuterated solvents were dried and purified through alumina prior to use. Aqueous solutions were prepared from nanopore water with a resistivity over 18 MΩ-cm. All reagents were commercially available unless otherwise stated. $Co(pic)_2$ was synthesized from known procedures.[3]

II. Substrate Synthesis

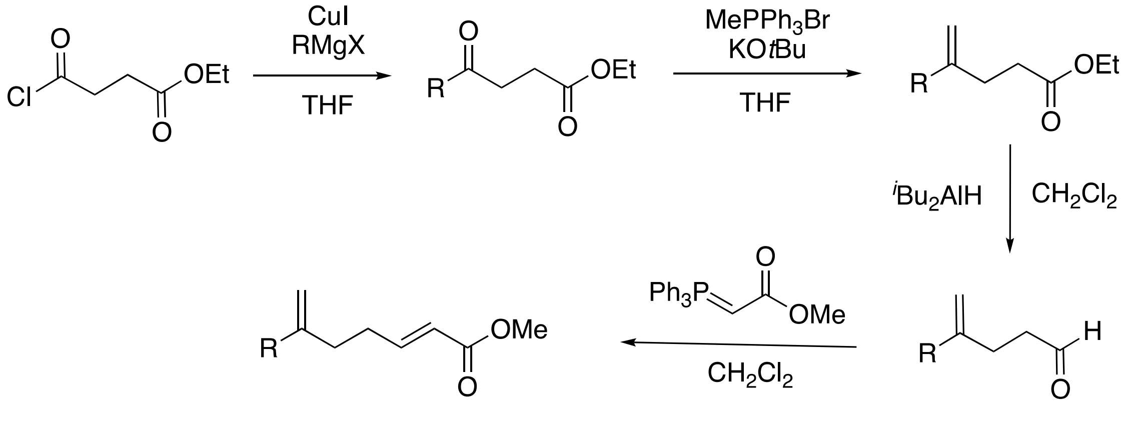

General procedure for the synthesis of *γ*-ketoesters: According to a procedure,[4] to a three-neck round-bottom flask equipped with an addition funnel was charged with ethyl succinyl chloride (1.00 equiv) and copper (I) iodide (1.00 equiv) in THF (1.00 M in the ethyl succinyl chloride) and cooled to 0 ºC. Grignard reagent (2.00 equiv, 1.00 M in THF) was added dropwise via the addition funnel at 0 ºC while stirring. After stirring for 2 h, saturated aqueous NH_4Cl (10.0 mL per mmol of ethyl succinyl chloride) was added and the reaction mixture extracted with

EtOAc (3 x 10.0 mL per mmol of ethyl succinyl chloride). The combined organic layers were washed with brine (10.0 mL per mmol of ethyl succinyl chloride), dried over anhydrous $MgSO_4$, filtered, and concentrated *in vacuo*. The unpurified γ-ketoester was then purified by flash column chromatography using hexane and ethyl acetate as the eluent.

General procedure for the synthesis of unsaturated esters: A reported procedure was modified to prepare unsaturated esters.[4] To a cooled (0ºC) solution of methytriphenylphosphonium bromide (1.50 equiv) in THF (0.300 M in the γ-ketoester) was added potassium *tert*-butoxide (1.50 equiv) in one portion. After 0.5 h, γ-ketoester in THF (1.00 mL per mmol of γ-ketoester) was added to the reaction flask dropwise at 0 ºC while stirring. The reaction mixture was then allowed to stir at 25 ºC for 16 h or until TLC showed that all starting material had been consumed. Saturated aqueous NH_4Cl (10.0 mL per mmol of γ-ketoester) was added to the reaction mixture and extracted with Et_2O (3 x 10.0 mL per mmol of γ-ketoester). The combined organic layers were washed with brine (10.0 mL per mmol of γ-ketoester), dried over anhydrous $MgSO_4$, filtered, and concentrated *in vacuo*. The unsaturated ester was then purified by flash column chromatography using hexane and ethyl acetate.

General procedure A for the synthesis of dienes: A three-neck round-bottom flask equipped with an addition funnel was charged with unsaturated ester (1.00 equiv) and CH_2Cl_2 (0.200 M in the unsaturated ester) and cooled to –78 ºC. Diisobutylaluminum hydride (1.2 M in PhMe or 1.00 M in CH_2Cl_2, 1.1 equiv) was added dropwise using an addition funnel. The reaction mixture was allowed to stir at –78 ºC for 2 h. MeOH (5.00 mL per mmol of diisobutylaluminum hydride) and 1.00 M aqueous HCl (5.00 mL per mmol of diisobutylaluminum hydride) were added to the reaction mixture at –78 ºC. The reaction mixture was then warmed to 25 ºC and stirred for 1 h at 25 ºC. The reaction mixture was extracted with CH_2Cl_2 (10.0 mL per mmol of unsaturated ester). The combined organic layers were washed with brine (10.0 mL per mmol unsaturated ester), dried over Na_2SO_4, filtered, and concentrated *in vacuo*. The unpurified aldehyde was then used immediately in the next step without further purification. To a solution of unpurified aldehyde (1.00 equiv) in CH_2Cl_2 (0.300 M to unpurified aldehyde) at 25 ºC, methyl (triphenylphosphoranylidene) acetate (1.50 equiv) was added and the reaction mixture was allowed to stir for 16 h. The reaction mixture was concentrated *in vacuo* and the product was purified by flash column chromatography using hexane and ethyl acetate.

General procedure B for the synthesis of dienes: To a solution of aldehyde (1 equiv) in CH_2Cl_2 (0.300 M in the aldehyde) was added stabilized Wittig reagent (1.10 equiv). The reaction mixture was stirred for 16 h at 25 ºC (unless noted otherwise). After 16 h, the reaction mixture was concentrated *in vacuo* and the product was purified by flash column chromatography using hexane and ethyl acetate.

General procedure for synthesis of unsaturated hydroperoxides: Co(pic)$_2$ (5.00 mol% or 15.0 mol% with respect to diene) was added to a stirring solution of diene (1.00 equiv) in 2-propanol (0.06 M in the diene) at 25 ºC. The reaction mixture was sonicated to ensure complete dissolution of catalyst. 1,1,3,3-Tetramethyldisiloxane (TMDSO, 2.20 equiv) and *t*-BuOOH (1.0 M in CH_2Cl_2, 0.200 equiv) were added sequentially to the reaction mixture. Oxygen was bubbled into the reaction mixture for 60 s using a balloon. The reaction mixture was then heated to 45 ºC in an oil bath under an O_2 atmosphere until TLC showed complete consumption of starting material (2–16 h). The reaction mixture was then concentrated *in vacuo* and filtered through a pad of silica gel (60:40 EtOAc:hexanes). The unpurified hydroperoxide was concentrated *in vacuo*, purified by flash column chromatography, and used immediately in the next step. If the unsaturated hydroperoxide were not used immediately, it was stored in a –20 ºC freezer under nitrogen gas. Using unpurified hydroperoxides resulted in lower yields in the next step and thus the hydroperoxides required purification to obtain higher yields as reported using silica gel (unless noted otherwise) using ethyl acetate and hexanes. Unsaturated hydroperoxides were mostly characterized by ^{1}H, ^{13}C NMR signals and HRMS due to their sensitivity, but full characterizations were performed on synthesized 1,2-dioxanes. Note: Despite multiple attempts to purify products by flash column chromatography, unsaturated hydroperoxides eluted with small amounts of impurities. These products could used in the subsequent step, and any impurities could be removed by chromatography at that point. Caution: Although no safety issues were encountered in the course of this work, any preparative work with peroxides should be performed with an appropriate awareness for the potential hazards arising from spontaneous, exothermic, and/or explosive decomposition.

General procedure for synthesis of 1,2-dioxanes: To a stirring solution of unsaturated hydroperoxide (1.00 equiv) in 2,2,2-trifluoroethanol (TFE, 0.150 M in the unsaturated hydroperoxide) was added triethylammonium hydrochloride ($HNEt_3Cl$, 2.00 equiv) at 25 ºC. The reaction mixture was then cooled to 0 ºC. NEt_3 (0.300 equiv) was then added dropwise. The reaction mixture was allowed to stir at 0 ºC for 2 h and then at 25 ºC for 16 h (unless noted otherwise). The reaction mixture was then concentrated *in vacuo* and purified by flash column chromatography using ethyl acetate and hexanes.

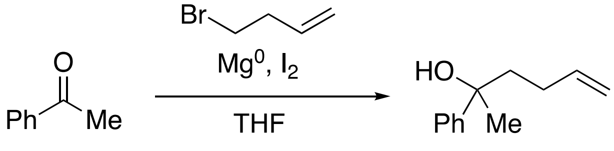

S1 **S2**

2-Phenylhex-5-en-2-ol (S2). A reported procedure was modified to prepare this compound.[5] A mixture of magnesium turnings (2.63 g, 108.3 mmol) and a small crystal of iodine (0.100 g, 0.394 mmol) was heated with a heat gun until the iodine vapors were evenly dispersed inside the flask. A solution of 4-bromobutene (13.3 g, 98.5 mmol) in THF (60 mL) was added dropwise by addition funnel over the course of 30 min to ensure a gentle reflux. The reaction mixture was

cooled (0 ºC) and a solution of acetophenone (5.92 g, 49.3 mmol) in THF (50 mL) was added dropwise by addition funnel. After 2 h, saturated aqueous NH_4Cl was added (200 mL) and the reaction mixture was extracted with Et_2O (3 x 200 mL). The combined organic phases were dried over $MgSO_4$, filtered, and concentrated i*n vacuo*. Purification by flash chromatography (10:90 EtOAc:hexanes) afforded 2-phenylhex-5-en-2-ol as a colorless oil (6.33 g, 73%). The spectroscopic data were consistent with those previously reported:[6]
^{1}H NMR (400 MHz, $CDCl_3$) δ 7.44 – 7.42 (m, 2H), 7.37 – 7.33 (m, 2H), 7.26 – 7.23 (m, 1H), 5.85 – 5.75 (m, 1H), 4.99 – 4.91 (m, 2H), 2.09 – 1.88 (m, 4H), 1.81–1.79 (m, 1H), 1.57 (s, 3H); ^{13}C{^{1}H} NMR (100 MHz, $CDCl_3$) δ 147.8 (C), 138.9 (CH), 128.3 (CH), 126.7 (CH), 124.9 (CH), 114.7 (CH_2), 74.9 (C), 43.2 (CH_2), 30.5 (CH_2), 28.7 (CH_3).

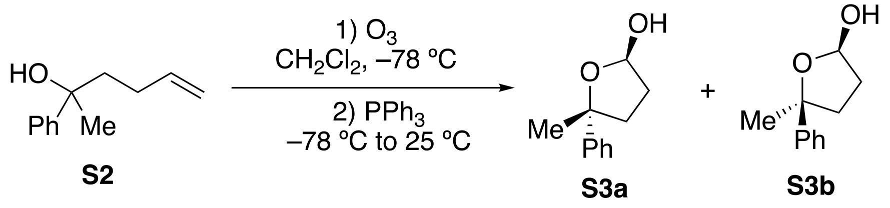

(2*R,5*R**)-5-Methyl-5-phenyltetrahydrofuran-2-ol (S3a) & (2*R**,5*S**)-5-Methyl-5-phenyltetrahydrofuran-2-ol (S3b).** A reported procedure was modified to prepare this compound.[7] To a cooled (–78 ºC) solution of 2-phenylhex-5-en-2-ol (7.00 g, 39.1 mmol) in CH_2Cl_2 (160 mL) was added a stream of ozone until a light blue color persisted. The reaction flask was purged with nitrogen gas for 1 h. Triphenylphosphine (12.3 g, 47.0 mmol) was added to the reaction mixture and the reaction mixture was warmed to 25 ºC. The reaction mixture was allowed to stir for 16 h. The reaction mixture was concentrated *in vacuo* and purified by flash column chromatography to afford the products as a colorless oil (5.90 g, 90%) as a mixture of diastereomers in a 60:40 ratio. The relative stereochemistry was not determined. Characterization was performed on a mixture of diastereomers (60:40). The spectroscopic data were consistent with those reported:[8]
^{1}H NMR (400 MHz, $CDCl_3$) δ 7.50–7.48 (m, 0.81H), 7.39–7.30 (m, 3.33H), 7.25–7.20 (m, 1.01H), 5.69–5.66 (m, 1.00H), 3.08–2.98 (m, 0.59H), 2.74 (m, 0.40H), 2.39–2.11 (m, 2.60H), 1.98–1.84 (m, 1.71H), 1.70 (s, 1.86H), 1.50 (s, 1.24H);
Peaks attributed to major diastereomer **3a**:
^{13}C{^{1}H} NMR (100 MHz, $CDCl_3$) δ 147.8 (C), 128.2 (CH, overlapping with minor diastereomer as determined by HSQC), 126.6 (CH), 124.7 (CH), 99.0 (CH), 86.4 (C), 37.9 (CH_2), 33.3 (CH_2), 31.5 (CH_3);
Peaks attributed to major diastereomer **3b**:
^{13}C{^{1}H} NMR (100 MHz, $CDCl_3$) δ 148.9 (C), 128.2 (CH, overlapping with major diastereomer as determined by HSQC), 126.7 (CH), 125.0 (CH), 99.2 (CH), 86.2 (C), 37.3 (CH_2), 33.9 (CH_2), 30.2 (CH_3).

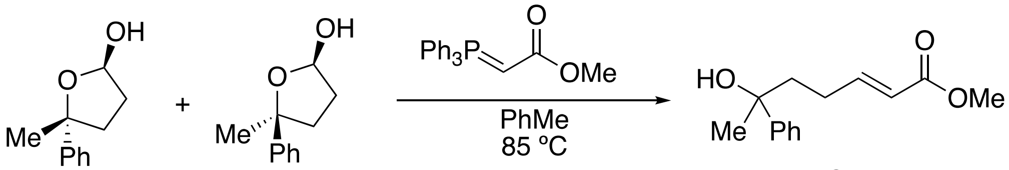

S3a S3b S4

Methyl (*E*)-6-hydroxy-6-phenylhept-2-enoate (S4). To a stirring solution of (2*R**,5*R**)-5-methyl-5-phenyltetrahydrofuran-2-ol & (2*R**,5*S**)-5-methyl-5-phenyltetrahydrofuran-2-ol (5.65 g, 31.7 mmol) as a mixture of diastereomers (60:40) in PhMe (160 mL) was added methyl 2-(triphenylphosphaneylidene)acetate (13.8 g, 41.2 mmol) at 25 ºC. The reaction mixture was heated to 85 ºC in an oil bath for 16 h. The reaction mixture was cooled to 25 ºC and concentrated *in vacuo*. Purification by flash column chromatography (25:75 EtOAc:hexanes) afforded methyl (*E*)-6-hydroxy-6-phenylhept-2-enoate as a clear viscous oil (7.18 g, 97%) as a single diastereomer. The relative configuration of the double bond was determined by analysis of *J* coupling constants:

^{1}H NMR (400 MHz, $CDCl_3$) δ 7.43–7.40 (m, 2H), 7.37–7.33 (m, 2H), 7.27–7.23 (m, 1H), 6.92 (dt, *J* = 15.7, 6.7 Hz, 1H), 5.75 (dt, *J* = 15.7, 1.6 Hz, 1H), 3.70 (s, 3H), 2.26–2.18 (m ,1H), 2.06–1.91 (m, 3H), 1.76–1.72 (m, 1H), 1.59 (s, 3H);

$^{13}C\{^1H\}$ NMR (100 MHz, $CDCl_3$) δ 167.2 (C), 149.7 (CH), 147.2 (C), 128.4 (CH), 126.9 (CH), 124.8 (CH), 120.9 (CH), 74.5 (C), 51.5 (CH_3), 42.3 (CH_2), 30.7 (CH_2), 27.2 (CH_3);

HRMS (ESI) *m* / *z* calcd for $C_{14}H_{18}NaO_3$ $(M + Na)^+$ 257.1148, found 257.1141;

IR (ATR) 3463, 2950, 1703, 1436, 1359, 1253 cm^{-1}.

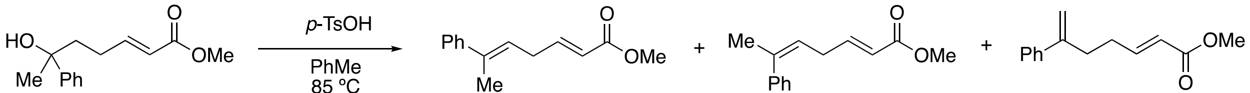

S4 8aa 8ab 8ac

Methyl (2*E*,5*E*)-6-phenylhepta-2,5-dienoate (8aa), Methyl (2*E*,5*Z*)-6-phenylhepta-2,5-dienoate (8ab) & Methyl (*E*)-6-phenylhepta-2,6-dienoate (8ac). A reported procedure was modified to prepare this compound.[9] *p*-Toluenesulfonic acid monohydrate (1.30 g, 6.81 mmol) was added to a stirring solution of methyl (E)-6-hydroxy-6-phenylhex-2-enoate (1.00 g, 4.54 mmol) in PhMe (45.0 mL). The reaction mixture was heated to 85 ºC in an oil bath for 2 h. The reaction mixture was cooled to 25 ºC and saturated aqueous $NaHCO_3$ (25 mL) was added. The reaction mixture was extracted with EtOAc (3 x 30 mL). The combined organic layers were washed with brine (50 mL), dried over $MgSO_4$, filtered, and concentrated *in vacuo*. Purification by flash column chromatography (5:95 EtOAc:hexanes) afforded the products as a light yellow oil (0.627 g, 68%) as a mixture of regioisomers in a 71:20:9 ratio. The relative configuration of the double bonds were determined by *J* coupling analysis. The relative stereochemistry of the products were not determined. Characterization was performed as a mixture of regioisomers (71:20:9):

^{1}H NMR (400 MHz, $CDCl_3$) δ 7.40–7.30 (m, 5.36H), 7.29–7.23 (m, 1.76H), 7.16–7.14 (m, 0.47H), 7.07–6.92 (m, 1.36H), 5.90 (dt, *J* = 15.7, 1.8 Hz, 1.00H), 5.86–5.76 (m, 1.46H), 5.47 (td,

J = 7.5, 1.4 Hz, 0.23H), 5.31 (s, 0.11H), 5.09 (dd, J = 2.5, 1.2 Hz, 0.11H), 3.75–3.73 (m, 3.91H), 3.13–3.10 (m, 2.12H), 2.87–2.83 (m, 0.47H), 2.68–2.65 (m, 0.24H), 2.39–2.33 (m, 0.24H), 2.09–2.05 (m, 3.96H);

Peaks attributed to s diene **8aa**:

$^{13}C\{^1H\}$ NMR (100 MHz, $CDCl_3$) δ 167.23 (C), 147.2 (CH), 143.3 (C), 138.0 (C), 128.4 (CH), 127.2 (CH), 125.8 (CH), 122.5 (CH), 121.3 (CH), 51.59 (CH_3), 31.5 (CH_2), 16.1 (CH_3);

Peaks attributed to s diene **8ab**:

$^{13}C\{^1H\}$ NMR (100 MHz, $CDCl_3$) δ 167.3 (C), 148.4 (CH), 141.4 (C), 139.7 (C), 128.5 (CH), 127.04 (CH), 126.2 (CH), 121.9 (CH), 121.0 (CH), 51.56 (CH_3), 32.0 (CH_2), 25.7 (CH_3);

Peaks attributed to s diene **8ac**:

$^{13}C\{^1H\}$ NMR (100 MHz, $CDCl_3$, characteristic peaks) δ 167.18 (C), 148.7 (CH), 140.8 (C), 128.3 (CH), 127.7 (CH), 126.96 (CH), 124.3 (CH), 113.3 (CH_2), 51.3 (CH_3), 33.9 (CH_2), 30.9 (CH_2);

HRMS (ESI) *m / z* calcd for $C_{14}H_{16}NaO_2$ $(M + Na)^+$ 239.1043, found 239.1053;

IR (ATR) 2949, 1720, 1493, 1434, 1326, 1268 cm^{-1}.

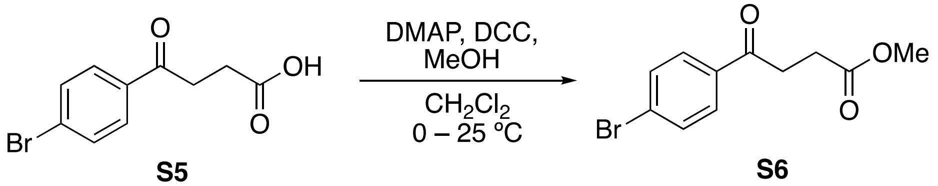

S5 **S6**

Methyl 4-(4-bromophenyl)-4-oxobutanoate (S6). A reported procedure was modified to prepare this compound.[10] A two-neck round bottom flask with an additional funnel was charged with 4-(4-bromophenyl)-4-oxobutanoic acid (10.0 g, 41.4 mmol), methanol (2.50 mL, 61.7 mmol), DMAP (0.502 g, 4.11 mmol) and CH_2Cl_2 (274 mL) and was stirred at 25 ºC. The reaction mixture was then cooled to 0 ºC by ice-water bath. To the additional funnel was added a solution of *N*,*N*'-dicyclohexylcarbodiimide (DCC, 12.7 g, 61.7 mmol) in CH_2Cl_2 (150 mL) and was added dropwise to the reaction mixture at 0 ºC while stirring. Upon complete addition of DCC, the ice-water bath was removed and the reaction mixture was allowed to stir at 25 ºC for 16 h. The reaction mixture was filtered twice through a Büchner funnel and concentrated *in vacuo*. The crude residue was redissolved in 200 mL of EtOAc and washed with 1 M aqueous HCl (2 x 100 mL), saturated aqueous $NaHCO_3$ (2 x 100 mL), and 200 mL of brine. The combined organic layers were dried over $MgSO_4$ and concentrated *in vacuo* to afford the product as a white solid (9.69 g, 87%) which was used in the next step without further purification. The spectroscopic data were consistent with those reported:[11]

^1H NMR (400 MHz, $CDCl_3$) δ 7.87–7.83 (m, 2H), 7.63–7.60 (m, 2H), 3.71 (s, 3H), 3.28 (t, J = 6.6 Hz, 2H), 2.77 (t, J = 6.6 Hz, 2H);

$^{13}C\{^1H\}$ NMR (100 MHz, $CDCl_3$) δ 197.2 (C), 173.4 (C), 135.4 (C), 132.1 (CH), 129.7 (CH), 128.6 (C), 52.0 (CH_3), 33.5 (CH_2), 28.1 (CH_2);

mp = 50–51 ºC.

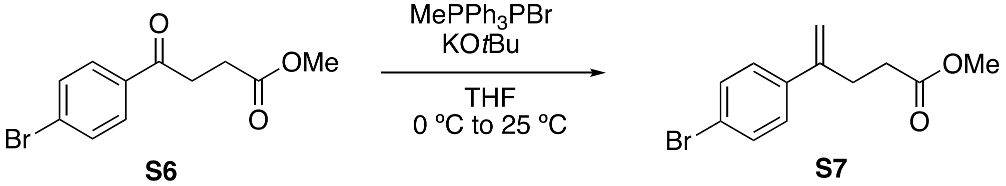

Methyl 4-(4-bromophenyl)pent-4-enoate (S7). Methyl 4-(4-bromophenyl)pent-4-enoate was prepared according to the general procedure for the synthesis of unsaturated esters. Methyl 4-(4-bromophenyl)-4-oxobutanoate (9.00 g, 33.2 mmol) in THF (33 mL) was mixed with methyltriphenylphosphonium bromide (17.8 g, 49.8 mmol) and potassium *tert*-butoxide (5.59 g, 49.8 mmol) in THF (110 mL). Purification by flash column chromatography (5:95 EtOAc:hexanes) afforded the product as a light yellow oil (6.23 g, 70%). The spectroscopic data were consistent with those reported:[12]

^{1}H NMR (400 MHz, $CDCl_3$) δ 7.46–7.42 (m, 2H), 7.27–7.23 (m, 2H), 5.28 (s, 1H), 5.09 (s, 1H), 3.65 (s, 3H), 2.79 (t, *J* = 8.1 Hz, 2H), 2.45 (t, *J* = 8.1 Hz, 2H);

$^{13}C\{^{1}H\}$ NMR (100 MHz, $CDCl_3$) δ 173.5 (C), 146.0 (C), 139.6 (C), 131.6 (CH), 127.9 (CH), 121.7 (C), 113.6 (CH_2), 51.8 (CH_3), 33.0 (CH_2), 30.4 (CH_2).

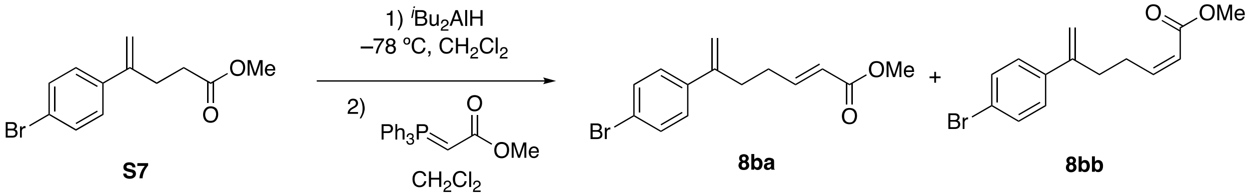

Methyl (*E*)-6-(4-bromophenyl)hepta-2,6-dienoate (8ba) & methyl (*Z*)-6-(4-bromophenyl)hepta-2,6-dienoate (8bb). Methyl (*E*)-6-(4-bromophenyl)hepta-2,6-dienoate and methyl (*Z*)-6-(4-bromophenyl)hepta-2,6-dienoate were prepared according to the general procedure for the synthesis of dienes. Diisobutylaluminum hydride (10.2 mL, 1.2 M in PhMe, 12 mmol) was mixed with a solution of methyl 4-(4-bromophenyl)pent-4-enoate (3.00 g, 11.1 mmol) in CH_2Cl_2 (56 mL) at –78 °C. The resulting unpurified aldehyde was used immediately in the next step. The unpurified aldehyde dissolved in CH_2Cl_2 (36 mL) was mixed with methyl(triphenylphosphoranylidene) acetate (5.50 g, 16.4 mmol). Purification by flash column chromatography (5:95 EtOAc:hexanes) afforded the product as a white solid as a mixture of *E*:*Z* isomers in a 88:12 ratio (2.72 g, 83%). The configuration of the double bonds were determined by analysis of *J* coupling constants. Characterization was performed on a mixture of *E*:*Z* isomers (88:12):

Peaks attributed to diene **8ba**: ^{1}H NMR (400 MHz, $CDCl_3$) δ 7.47–7.45 (m, 2H), 7.25–7.23 (m, 2H), 6.95 (dt, *J* = 15.7, 6.9 Hz, 1H), 5.79 (dt, *J* = 15.7, 1.6 Hz, 1H), 5.30 (d, *J* = 1.1 Hz, 1H), 5.10 (q, *J* = 1.1 Hz, 1H), 3.72 (s, 3H), 2.63 (dt, *J* = 6.1, 1.3 Hz, 2H), 2.37–2.31 (m, 2H);

$^{13}C\{^{1}H\}$ NMR (100 MHz, $CDCl_3$) δ 167.1 (C), 148.3 (CH), 146.1 (C), 139.8 (C), 131.7 (CH), 127.9 (2CH, overlapping as determined by HSQC), 121.6 (CH), 113.9 (CH_2), 51.6 (CH_3), 33.7 (CH_2), 30.8 (CH_2);

Peaks attributed to diene **8bb**: ^{1}H NMR (400 MHz, $CDCl_3$) δ 6.22 (dt, *J* = 11.5, 7.4 Hz, 1H), 5.78 (dt, *J* = 11.5, 1.7 Hz, 1H), 5.31–5.30 (m, 1H), 5.21 (d, *J* = 1.4 Hz, 1H), 3.67 (s, 3H), 2.84–2.78 (m, 2H);
$^{13}C\{^1H\}$ NMR (100 MHz, $CDCl_3$, characteristic peaks) δ 149.2 (CH), 131.6 (CH), 128.0 (CH), 51.2 (CH_3);
HRMS (ESI) calcd for $C_{14}H_{16}BrO_2$ $(M + H)^+$ 295.0328, found 295.0328;
HRMS (ESI) calcd for $C_{14}H_{15}BrNaO_2$ $(M + Na)^+$ 317.0148, found 317.0155;
IR (ATR) 2991, 1725, 1437, 1264, 1009, 733 cm^{-1};
mp = 43–45 °C.

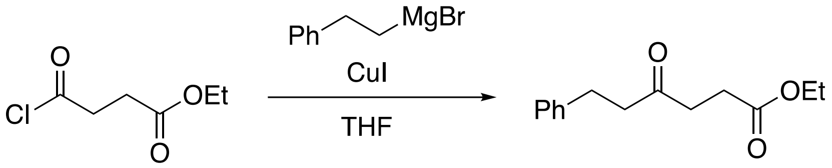

S8 S9

Ethyl 4-oxo-6-phenylhexanoate (S9). Ethyl 4-oxo-6-phenylhexanoate was prepared according to the general procedure for the synthesis of γ-ketoesters. Ethyl succinyl chloride (6.58 g, 40.0 mmol) and copper (I) iodide (7.62 g, 40.0 mmol) in THF (40 mL) was mixed with phenethylmagnesium bromide (80.0 mL, 1.00 M in THF, 80.0 mmol). Purification by flash column chromatography (15:85 EtOAc:hexanes) afforded the product as a yellow oil (4.27 g, 46%). The spectroscopic data were consistent with those reported:[13]
^{1}H NMR (400 MHz, $CDCl_3$) δ 7.30–7.26 (m, 2H), 7.20–7.17 (m, 3H), 4.13 (q, *J* = 7.2 Hz, 2H), 2.92 (t, *J* = 7.4 Hz, 2H), 2.78 (t, *J* = 7.4 Hz, 2H), 2.70 (t, *J* = 7.4 Hz, 2H), 2.58 (t, *J* = 7.4 Hz, 2H), 1.25 (t, *J* = 7.2 Hz, 3H);
$^{13}C\{^1H\}$ NMR (100 MHz, $CDCl_3$) δ 208.1 (C), 172.9 (C), 141.1 (C), 128.6 (CH), 128.4 (CH), 126.2 (CH), 60.8 (CH_2), 44.4 (CH_2), 37.4 (CH_2), 29.8 (CH_2), 28.1 (CH_2), 14.3 (CH_3).

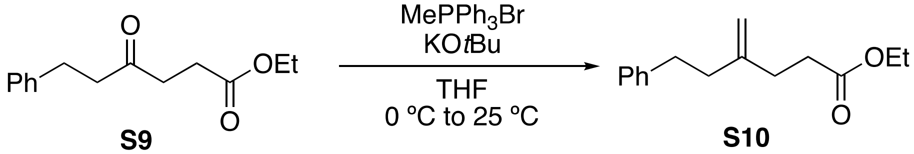

S9 S10

Ethyl 4-methylene-6-phenylhexanoate (S10). Ethyl 4-methylene-6-phenylhexanoate was prepared according to the general procedure for the synthesis of unsaturated esters. Ethyl 4-oxo-6-phenylhexanoate (4.00 g, 17.1 mmol) in THF (20.0 mL) was mixed with methyltriphenylphosphonium bromide (9.18 g, 25.7 mmol) and potassium *tert*-butoxide (2.88 g, 25.7 mmol) in THF (45 mL). Purification by flash column chromatography (5:95 EtOAc:hexanes) afforded the product as a light yellow oil (2.52 g, 63%). The spectroscopic data were consistent with those reported:[14]
^{1}H NMR (400 MHz, $CDCl_3$) δ 7.36–7.32 (m, 3H), 7.26–7.22 (m, 2H), 4.86 (s, 1H), 4.83 (s, 1H), 4.20 (q, *J* = 7.1 Hz, 2H), 2.82 (t, *J* = 7.9 Hz, 2H), 2.55–2.51 (m, 2H), 2.47–2.38 (m, 4H), 1.31 (t, *J* = 7.1 Hz, 3H);

$^{13}C\{^1H\}$ NMR (100 MHz, $CDCl_3$) δ 173.4 (C), 147.6 (C), 142.1 (C), 128.5 (2CH, as determined by HSQC and DEPT), 126.0 (CH), 109.9 (CH_2), 60.5 (CH_2), 38.2 (CH_2), 34.4 (CH_2), 32.9 (CH_2), 31.2 (CH_2), 14.4 (CH_3).

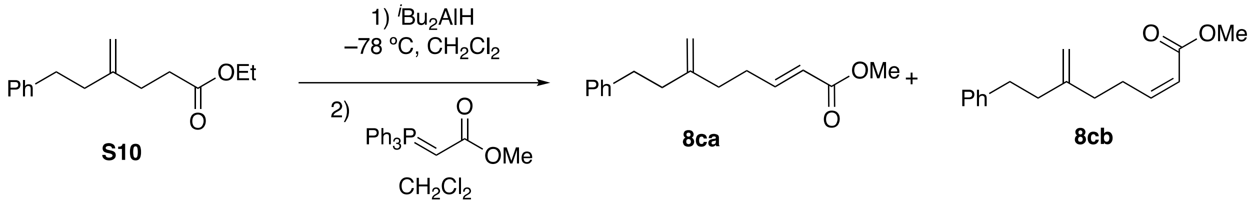

Methyl (*E*)-6-methylene-8-phenyloct-2-enoate (8ca) & Methyl (*Z*)-6-methylene-8-phenyloct-2-enoate (8cb). Methyl (*E*)-6-methylene-8-phenyloct-2-enoate and methyl (*Z*)-6-methylene-8-phenyloct-2-enoate were prepared according to the general procedure for the synthesis of dienes. Diisobutylaluminum hydride (5.00 mL, 1.2 M in PhMe, 5.9 mmol) was mixed with a solution of ethyl 4-methylene-6-phenylhexanoate (1.25 g, 5.38 mmol) in CH_2Cl_2 (27.0 mL). The resulting unpurified aldehyde was used immediately in the next step. The unpurified aldehyde in CH_2Cl_2 (12.0 mL) was mixed with methyl(triphenylphosphoranylidene) acetate (1.74 g, 5.21 mmol). Purification by flash column chromatography (5:95 EtOAc:hexanes) afforded the product as a light yellow oil as a mixture of *E*:*Z* isomers in a 95:5 ratio (0.707 g, 54% over two steps). The configuration of the double bonds were determined by analysis of *J* coupling constants.

Characterization was performed on a mixture of *E*:*Z* isomers (95:5):
Peaks attributed to diene **8ca**: ^1H NMR (400 MHz, $CDCl_3$) δ 7.30–7.27 (m, 2H), 7.21–7.17 (m, 3H), 6.98 (dt, *J* = 15.7, 6.8 Hz, 1H), 5.85 (dt, *J* = 15.7, 1.6 Hz, 1H), 4.83 (s, 1H), 4.79 (s, 1H), 3.73 (s, 3H), 2.78–2.74 (m, 2H), 2.40–2.31 (m, 4H), 2.23–2.19 (m, 2H);
$^{13}C\{^1H\}$ NMR (100 MHz, $CDCl_3$) δ 167.2 (C), 149.0 (CH), 147.6 (C), 142.1 (C), 128.5 (CH), 128.4 (CH), 126.0 (CH), 121.3 (CH), 110.3 (CH_2), 51.6 (CH_3), 38.0 (CH_2), 34.6 (CH_2), 34.4 (CH_2), 30.5 (CH_2);
Peaks attributed to diene **8cb**: ^1H NMR (400 MHz, $CDCl_3$, characteristic peaks) δ 6.23 (dt, *J* = 11.5, 7.3 Hz, 1H), 5.79 (dt, *J* = 11.5, 1.8 Hz, 1H), 3.71 (s, 3H), 2.84 (dq, *J* = 14.2, 2.7 Hz, 2H),
$^{13}C\{^1H\}$ NMR (400 MHz, $CDCl_3$, characteristic peaks) δ 150.1(CH), 125.9 (CH), 119.7 (CH), 110.2 (CH_2), 51.2 (CH_3), 37.8 (CH_2), 35.4 (CH_2), 27.2 (CH_2);
HRMS (ESI) *m* / *z* calcd for $C_{16}H_{20}NaO_2$ $(M + Na)^+$ 267.1356, found 267.1353;
IR (ATR) 3026, 2946, 1721, 1657, 1434, 1152 cm^{-1}.

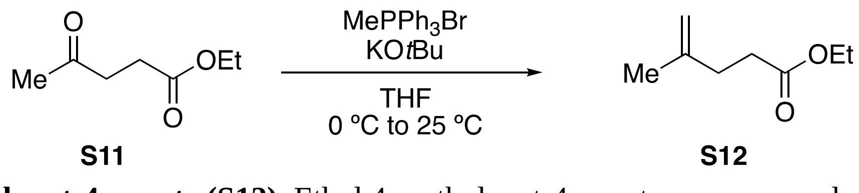

Ethyl 4-methylpent-4-enoate (S12). Ethyl 4-methylpent-4-enoate was prepared according to the general procedure for the synthesis of unsaturated esters. Ethyl levulinate (8.28g, 54.7 mmol) in THF (60 mL) was mixed with methyltriphenylphosphonium bromide (30.5 g, 86.1 mmol) and potassium *tert*-butoxide (9.65 g, 86.1 mmol) in THF (300 mL). Purification by flash column

chromatography (5:95 EtOAc:hexanes) afforded the product as a volatile, colorless oil (5.40 g, 66%). The spectroscopic data were consistent with those reported:[15]

^{1}H NMR (400 MHz, $CDCl_3$) δ 4.69 (s, 1H), 4.64 (s, 1H), 4.08 (q, *J* = 7.1 Hz, 2H), 2.42–2.38 (m, 2H), 2.30–2.26 (m, 2H), 1.69 (s, 3H), 1.20 (t, *J* = 7.1 Hz, 3H);

^{13}C{^{1}H} NMR (100 MHz, $CDCl_3$) δ 173.4 (C), 144.3 (C), 110.5 (CH_2), 60.5 (CH_2), 32.84 (CH_2), 32.78 (CH_2), 22.6 (CH_3), 14.4 (CH_3).

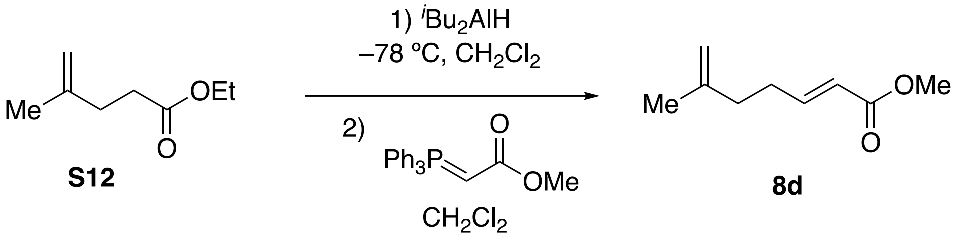

Methyl (*E*)-6-methylhepta-2,6-dienoate (8d). Methyl (*E*)-6-methylhepta-2,6-dienoate was prepared according to the general procedure of dienes. Diisobutylaluminum hydride (19.3 mL, 1.2 M in PhMe, 23 mmol) was mixed with ethyl 4-methylpent-4-enoate (3.00 g, 21.0 mmol) in CH_2Cl_2 (84.0 mL). The resulting unpurified aldehyde was used immediately in the next step. The unpurified aldehyde in CH_2Cl_2 (50.0 mL) was mixed with methyl(triphenylphosphoranylidene) acetate (8.43 g, 25.2 mmol). Purification by flash column chromatography (3:97 EtOAc:hexanes) afforded the product as a colorless oil (1.35 g, 42%). The spectroscopic data were consistent with those reported:[16]

^{1}H NMR (400 MHz, $CDCl_3$) δ 6.97 (dt, *J* = 15.6, 6.8 Hz, 1H), 5.84 (dt, *J* = 15.6, 1.6 Hz, 1H), 4.76 (s, 1H), 4.70 (s, 1H), 3.73 (s, 3H), 2.38–2.33 (m, 2H), 2.16 (t, *J* = 7.6 Hz, 2H), 1.73 (s, 3H);

^{13}C{^{1}H} NMR (100 MHz, $CDCl_3$) δ 167.2 (C), 149.1 (CH), 144.3 (C), 121.2 (CH), 110.9 (CH_2), 51.6 (CH_3), 36.1 (CH_2), 30.4 (CH_2), 22.5 (CH_2).

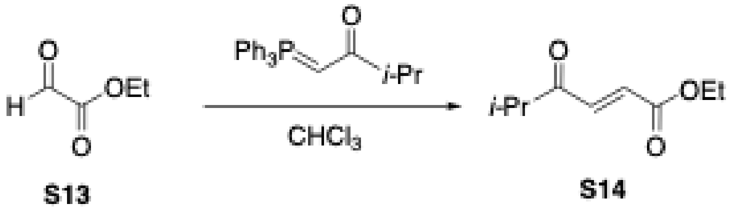

Ethyl (*E*)-5-methyl-4-oxohex-2-enoate (S14). According to a procedure,[16] to a stirring solution of 3-methyl-1-(triphenylphosphaneylidene)butan-2-one (10.0 g, 28.9 mmol), synthesized by known procedures,[17] in $CHCl_3$ (53.0 mL) was added ethyl 2-oxoacetate (5.40 mL, 50% in PhMe, 26 mmol) at 25 ºC. The reaction mixture was allowed to stir for 16 h. The reaction mixture was concentrated *in vacuo* and purified by flash column chromatography (5:95 to 10:90 EtOAc:hexanes) to afford the product as a yellow oil (3.47 g, 78%) as a single diastereomer. The spectroscopic data were consistent with those reported:[18]

^{1}H NMR (400 MHz, $CDCl_3$) δ 7.18 (d, *J* = 15.8 Hz, 1H), 6.72 (d, *J* = 15.8 Hz, 1H), 4.26 (q, *J* = 7.1 Hz, 2H), 2.68 (sept, *J* = 6.9 Hz, 1H), 1.32 (t, *J* = 7.1 Hz, 3H), 1.15 (d, *J* = 6.9 Hz, 6H);

^{13}C{^{1}H} NMR (100 MHz, $CDCl_3$) δ 203.1 (C), 165.8 (C), 138.1 (CH), 131.0 (CH), 61.5 (CH_2), 40.1 (CH), 18.0 (CH_3), 14.3 (CH_3).

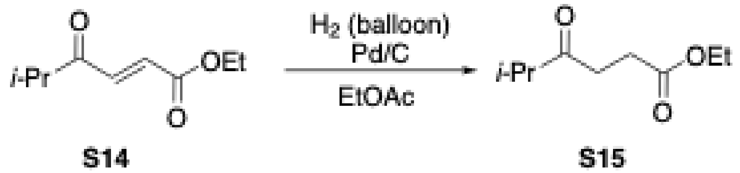

Ethyl 5-methyl-4-oxohexanoate (S15). According to a procedure,[19] Pd/C (0.590 g, 5.51 mmol) was added to a stirring solution of ethyl (*E*)-5-methyl-4-oxohex-2-enoate (3.47 g, 20.4 mmol) in EtOAc (205 mL). The reaction flask was purged with molecular hydrogen using a balloon. The reaction mixture was allowed to stir under an atmosphere of molecular hydrogen for 16 h. The reaction mixture was filtered over diatomaceous earth and concentrated *in vacuo* to afford ethyl 5-methyl-4-oxohexanoate as a light yellow oil (2.25 g, 65%), which was used in the next step without any further purification. The spectroscopic data were consistent with those reported:[20]
^{1}H NMR (400 MHz, $CDCl_3$) δ 4.12 (q, *J* = 7.1 Hz, 2H), 2.76 (t, *J* = 6.6 Hz, 2H), 2.64 (sept, *J* = 6.9 Hz, 1H), 2.57 (t, *J* = 6.6 Hz, 2H), 1.24 (t, *J* = 7.1 Hz, 3H), 1.12 (d, *J* = 6.9 Hz, 6H);
^{13}C{^{1}H} NMR (100 MHz, $CDCl_3$) δ 212.9 (C), 173.0 (C), 60.7 (CH_2), 40.9 (CH), 34.9 (CH_2), 28.2 (CH_2), 18.4 (CH_3), 14.3 (CH_3).

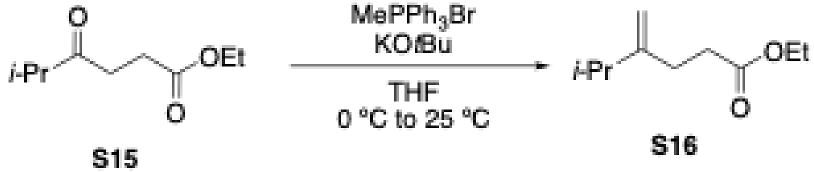

Ethyl 5-methyl-4-methylenehexanoate (S16). Ethyl 5-methyl-4-methylenehexanoate was prepared according to the general procedure for the synthesis of unsaturated esters. Ethyl 5-methyl-4-oxohexanoate (2.99 g, 17.6 mmol) in THF (20 mL) was mixed with methyltriphenylphosphonium bromide (9.43 g, 26.4 mmol) and potassium *tert*-butoxide (2.96 g, 2641 mmol) in THF (60 mL). Purification by flash column chromatography (5:95 EtOAc:hexanes) afforded the product as a volatile, colorless oil (1.40 g, 47%). The spectroscopic data were consistent with those reported:[15]
^{1}H NMR (400 MHz, $CDCl_3$) δ 4.78 (s, 1H), 4.66 (s, 1H), 4.13 (q, *J* = 7.1 Hz, 2H), 2.48–2.44 (m, 2H), 2.39–2.34 (m, 2H), 2.25 (sept, *J* = 6.9 Hz, 1H), 1.26 (t, *J* = 7.1 Hz, 3H), 1.04 (d, *J* = 6.9 Hz, 6H);
^{13}C{^{1}H} NMR (100 MHz, $CDCl_3$) δ 173.6 (C), 154.4 (C), 106.8 (CH_2), 60.4 (CH_2), 34.3 (CH), 33.1 (CH_2), 29.2 (CH_2), 21.9 (CH_3), 14.4 (CH_3).

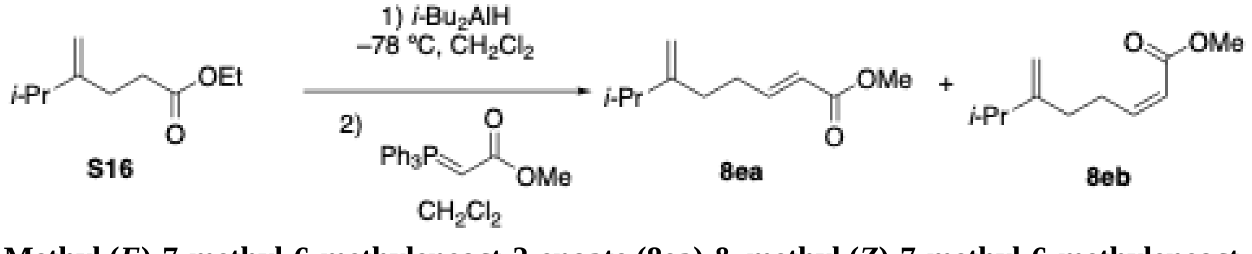

Methyl (*E*)-7-methyl-6-methyleneoct-2-enoate (8ea) & methyl (*Z*)-7-methyl-6-methyleneoct-2-enoate (8eb). Methyl (*E*)-7-methyl-6-methyleneoct-2-enoate & methyl (*Z*)-7-methyl-6-

methyleneoct-2-enoate were prepared according to the general procedure for the synthesis of dienes. Diisobutylaluminum hydride (10.0 mL, 1.2 M in PhMe, 12 mmol) was mixed with ethyl 5-methyl-4-methylenehexanoate (1.83 g, 10.7 mmol) in CH_2Cl_2 (54.0 mL) at –78 ºC. The resulting unpurified aldehyde was used immediately in the next step. The unpurified aldehyde dissolved in CH_2Cl_2 (50.0 mL) was mixed with methyl(triphenylphosphoranylidene) acetate (7.16 g, 21.4 mmol). Purification by flash column chromatography (5:95 EtOAc:hexanes) afforded the product as a light yellow oil as a mixture of *E*:*Z* isomers in a 94:6 ratio (1.21 g, 62%). The configuration of the double bonds were determined by analysis of *J* coupling constants. Characterization was performed on a mixture of *E*:*Z* isomers (94:6) ratio:

Peaks attributed to diene **8ea**: ^{1}H NMR (400 MHz, $CDCl_3$) δ 6.99 (dt, *J* = 15.7, 6.8 Hz, 1H), 5.84 (dt, *J* = 15.7, 1.5 Hz, 1H), 4.80 (s, 1H), 4.68 (d, *J* = 1.2 Hz, 1H), 3.72 (s, 3H), 2.38–2.33 (m, 2H), 2.26–2.15 (m, 3H), 1.03 (d, *J* = 6.8 Hz, 6H);

^{13}C{^{1}H} NMR (100 MHz, $CDCl_3$) δ 167.2 (C), 154.4 (C), 149.3 (CH), 121.2 (CH), 107.3 (CH_2), 51.5 (CH_3), 34.0 (CH), 32.5 (CH_2), 30.8 (CH_2), 21.9 (CH_3);

Peaks attributed to diene **8eb**: ^{1}H NMR (400 MHz, $CDCl_3$, characteristic peaks) δ 6.23 (dt, *J* = 11.5, 7.3 Hz, 1H), 5.78 (dt, *J* = 11.5, 1.7 Hz, 1H), 4.71 (d, *J* = 1.3 Hz, 1H), 3.71 (s, 3H), 2.84–2.78 (m, 2H);

^{13}C{^{1}H} NMR (100 MHz, $CDCl_3$, characteristic peaks) δ 167.0 (C), 154.7 (C), 150.5 (CH), 119.4 (CH), 51.2 (CH_3), 33.8 (CH), 33.5 (CH_2), 27.5 (CH_2), 21.9 (CH_3);

HRMS (ESI) *m* / *z* calcd for $C_{11}H_{18}NaO_2$ $(M + Na)^+$ 205.1199, found 205.1202;

IR (ATR) 2960, 2927, 1724, 1644, 1269, 1197 cm^{-1}.

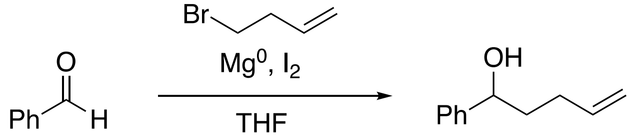

S17 **S18**

1-Phenylpent-4-en-1-ol (S18). A reported procedure was modified to prepare this compound.[5] A mixture of magnesium turnings (2.66 g, 110 mmol) and a small crystal of iodine (0.267 g, 0.394 mmol) was heated with a heat gun until the iodine vapors were evenly dispersed inside the flask. A solution of 4-bromobutene (13.5 g, 100 mmol) in THF (70.0 mL) was added dropwise by addition funnel over the course of 30 min to ensure a gentle reflux. The reaction mixture was cooled (0 ºC) and a solution of benzaldehyde (5.31 g, 50.0 mmol) in THF (50.0 mL) was added dropwise by addition funnel. After 2 h, saturated aqueous NH_4Cl was added (200 mL) and the reaction mixture was extracted with Et_2O (3 x 200 mL). The combined organic layers were washed with brine (200 mL), dried over $MgSO_4$, filtered, and concentrated i*n vacuo* and used without any further purification as a light yellow oil (7.62 g, 94%). The unpurified product was used without any further purification. The spectroscopic data were consistent with those previously reported:[5]

^{1}H NMR (400 MHz, $CDCl_3$) δ 7.36–7.33 (m, 4H), 7.29–7.26 (m, 1H), 5.84 (ddt, *J* = 17.0, 10.3, 6.7 Hz, 1H), 5.07–4.96 (m, 2H), 4.69 (dd, *J* = 7.6, 5.6 Hz, 1H), 2.22–2.05 (m, 2H), 1.95–1.76 (m, 3H);

$^{13}C\{^1H\}$ NMR (100 MHz, $CDCl_3$) δ 144.7 (CH), 138.3 (C), 128.6 (CH), 127.7 (CH), 126.0 (CH), 115.1 (CH_2), 74.2 (CH), 38.2 (CH_2), 30.2 (CH_2).

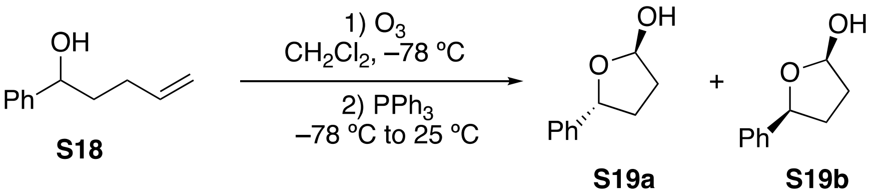

(2*R,5*R**)-5-Phenyltetrahydrofuran-2-ol (S19a) & (2*R**,5*S**)-5-Phenyltetrahydrofuran-2-ol (S19b).** A reported procedure was modified to prepare this compound.[7] To a cooled (–78 ºC) solution of 1-phenylpent-4-en-1-ol (7.62 g, 47.0 mmol) in CH_2Cl_2 (235 mL) was added a stream of ozone until a light blue color persisted. The reaction flask was purged with nitrogen gas for 1 h. Triphenylphosphine (14.8 g, 56.4 mmol) was added and the reaction mixture was warmed to 25 ºC. The reaction mixture was allowed to stir for 16 h. The reaction mixture was concentrated *in vacuo* and purified by flash column chromatography (15:85 EtOAc:hexanes) to afford the products as a colorless oil (6.21 g, 80%) as a mixture of diastereomers in a 56:44 ratio. The relative stereochemistry was not determined. Characterization was performed on a mixture of diastereomers (56:44). The spectroscopic data were consistent with those reported:[21]

^1H NMR (400 MHz, $CDCl_3$) δ 7.45–7.24 (m, 5H), 5.78–5.76 (m, 0.52H), 5.63 (br s, 0.39H), 5.26 (t, J = 7.0 Hz, 0.52H), 5.03–5.00 (m, 0.41H), 3.17–3.09 (m, 0.87H), 2.52–2.44 (m, 0.55H), 2.31–2.04 (m, 2.38H), 1.99–1.92 (m, 0.58H), 1.85–1.76 (m, 0.63H);

Peaks attributed to major diastereomer **S19a**:

$^{13}C\{^1H\}$ NMR (100 MHz, $CDCl_3$) δ 142.5 (C), 128.5 (CH,), 127.5 (CH), 125.8 (CH), 99.1 (CH), 79.9 (CH), 32.3 (CH_2), 32.9 (CH_2);

Peaks attributed to major diastereomer **S19b**:

$^{13}C\{^1H\}$ NMR (100 MHz, $CDCl_3$) δ 142.9 (C), 128.5 (CH,), 127.6 (CH), 126.5 (CH), 98.8 (CH), 83.1 (CH), 34.7 (CH_2), 32.8 (CH_2).

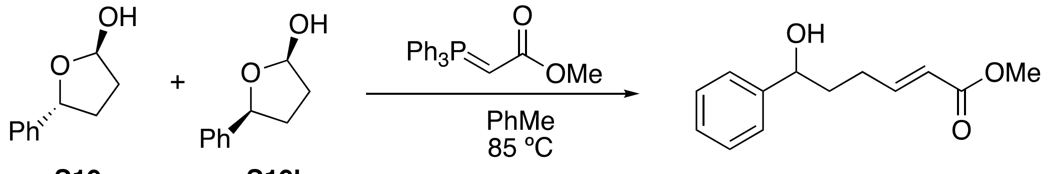

Methyl (E)-6-hydroxy-6-phenylhex-2-enoate (S20). A reported procedure was modified to prepare this compound.[22] To a stirring solution of (2*R*,5*R*)-5-methyl-5-phenyltetrahydrofuran-2-ol & (2*R*,5*S*)-5-methyl-5-phenyltetrahydrofuran-2-ol (6.11 g, 37.2 mmol) as a mixture of diastereomers (53:47) in PhMe (186 mL) was added methyl 2-(triphenylphosphaneylidene)acetate (16.2 g, 48.4 mmol) at 25 ºC. The reaction mixture was heated to 85 ºC in an oil bath for 16 h. The reaction mixture was cooled to 25 ºC and concentrated *in vacuo*. Purification by flash column chromatography (25:75 EtOAc:hexanes) afforded methyl (*E*)-6-hydroxy-6-phenylhex-2-enoate as a clear viscous oil (6.50 g, 79%) as a single diastereomer. The relative configuration of the double bond was determined by analysis of *J* coupling constants. The spectroscopic data were consistent with those reported:[22]

^{1}H NMR (400 MHz, $CDCl_3$) δ 7.37–7.27 (m, 5H), 6.98 (dt, *J* = 15.6, 6.9 Hz, 1H), 5.84 (dt, *J* = 15.6, 1.6 Hz, 1H), 4.69 (t, *J* = 6.5 Hz, 1H), 3.71 (s, 3H), 2.36–2.22 (m, 2H), 1.99–1.81 (m, 3H);
$^{13}C\{^1H\}$ NMR (100 MHz, $CDCl_3$) δ 167.2 (C), 148.9 (CH), 144.3 (C), 128.7 (CH), 128.0 (CH), 126.0 (CH), 121.4 (CH), 73.8 (CH), 51.8 (CH_3), 37.2 (CH_2), 28.6 (CH_2).

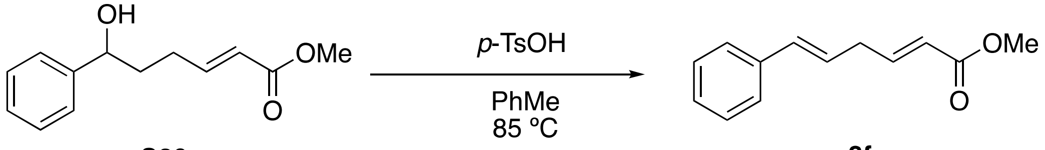

Methyl (2*E*,5*E*)-6-phenylhexa-2,5-dienoate (8f). A reported procedure was modified to prepare this compound.[9] *p*-Toluenesulfonic acid monohydrate (1.30 g, 6.81 mmol) was added to a stirring solution of methyl (E)-6-hydroxy-6-phenylhex-2-enoate (1.00 g, 4.54 mmol) in PhMe (45.0 mL). The reaction mixture was heated to 85 ºC in an oil bath for 2 h. The reaction mixture was cooled to 25 ºC and saturated aqueous $NaHCO_3$ (25 mL) was added. The reaction mixture was extracted with EtOAc (3 x 30 mL). The combined organic layers were washed with brine (50 mL), dried over $MgSO_4$, filtered, and concentrated *in vacuo*. Purification by flash column chromatography (5:95 EtOAc:hexanes) afforded the product as a light yellow oil (0.627 g, 68%) as a single diastereomer. The relative configuration of the double bonds were determined by *J* coupling analysis:
^{1}H NMR (400 MHz, $CDCl_3$) δ 7.37–7.29 (m, 4H), 7.25–7.20 (m, 1H), 7.05 (dt, *J* = 15.7, 6.5 Hz, 1H), 6.45 (d, *J* = 15.9 Hz, 1H), 6.19 (dt, *J* = 15.9, 6.7 Hz, 1H), 5.91 (dt, *J* = 15.7, 1.6 Hz, 1H), 3.74 (s, 3H), 3.11 (tt, *J* = 6.7, 1.6 Hz, 2H);
$^{13}C\{^1H\}$ NMR (100 MHz, $CDCl_3$) δ 167.1 (C), 146.9 (CH), 137.2 (C), 132.6 (CH), 128.7 (CH), 127.6 (CH), 126.3 (CH), 125.5 (CH), 122.0 (CH), 51.6 (CH_3), 35.4 (CH_2);
HRMS (ESI) *m* / *z* calcd for $C_{13}H_{14}NaO_2$ $(M + Na)^+$ 225.0886, found 225.0884;
IR (ATR) 3207, 2950, 1717, 1435, 1269, 1201 cm^{-1}.

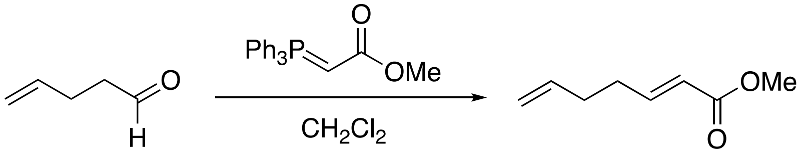

Methyl (*E*)-hepta-2,6-dienoate (*trans*-8g). According to a procedure,[23] to a stirring solution of 4-pentenal (1.09 g, 13.0 mmol) in CH_2Cl_2 (30.0 mL) was added methyl(triphenylphosphoranylidene) acetate (6.54 g, 19.5 mmol) at 25 ºC and the reaction mixture was allowed to stir for 16 h. The reaction mixture was concentrated *in vacuo* and the product was purified by flash column chromatography (10:90 EtOAc:hexanes) to afford methyl (*E*)-hepta-2,6-dienoate as a light yellow oil (1.27 g, 69%). The spectroscopic data were consistent with those reported:[23]
^{1}H NMR (400 MHz, $CDCl_3$) δ 6.97 (dt, *J* = 15.6, 6.7 Hz, 1H), 5.87–5.75 (m, 2H), 5.08–5.00 (m, 2H), 3.72 (s, 3H), 2.34–2.28 (m, 2H), 2.25–2.19 (m, 2H);

$^{13}C\{^1H\}$ NMR (100 MHz, $CDCl_3$) δ 167.2 (C), 148.7 (CH), 137.2 (CH), 121.4 (CH), 115.7 (CH_2), 51.6 (CH_3), 32.2 (CH_2), 31.6 (CH_2).

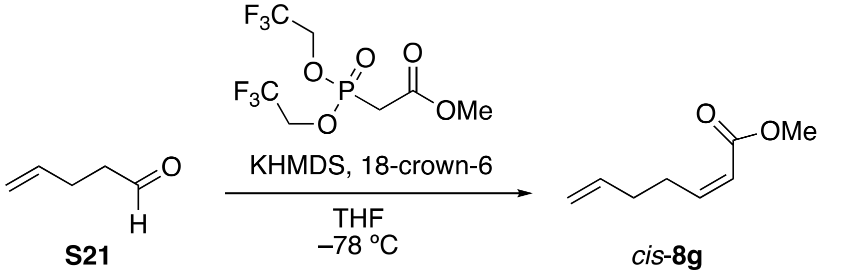

Methyl (*Z*)-hepta-2,6-dienoate (*cis*-8g). To a cooled (–78 ºC) solution of 18-crown-6 (10.6 g, 40.2 mmol) and methyl 2-(bis(2,2,2-trifluoroethoxy)phosphoryl)acetate (3.85 g, 12.1 mmol) in THF (100 mL) was added KHMDS (15.0 mL, 0.700 M in PhMe, 10.5 mmol) dropwise. The reaction mixture was allowed to stir at –78 ºC for 0.5 h. To the reaction mixture was added a solution of 4-pentenal (0.676 g, 8.04 mmol) in THF (8.00 mL) dropwise. The reaction mixture was allowed to stir at –78 ºC for 3 h. Et_2O (50.0 mL) and 1 M aqueous HCl (50.0 mL) were added to the reaction mixture and the reaction mixture was allowed to warm to 25 ºC. The reaction mixture was extracted with Et_2O (3 x 30 mL). The combined organic layers were washed with brine (100 mL), dried over $MgSO_4$, filtered, and concentrated *in vacuo*. The unpurified product was purified by flash column chromatography (3:97 EtOAc:hexanes) to afford methyl (*Z*)-hepta-2,6-dienoate as a volatile, colorless oil (0.424 g, 38%). The spectroscopic data were consistent with those reported:[24]

^1H NMR (400 MHz, $CDCl_3$) δ 6.22 (dt, *J* = 11.5, 7.4 Hz, 1H), 5.87–5.76 (m, 2H), 5.08–4.98 (m, 2H), 3.71 (s, 3H), 2.76 (m, 2H), 2.23–2.18 (m 2H);

$^{13}C\{^1H\}$ NMR (100 MHz, $CDCl_3$) δ 166.9 (C), 149.9 (CH), 137.7 (CH), 119.8 (CH), 115.4 (CH_2), 51.2 (CH_3), 33.1 (CH_2), 28.3 (CH_2).

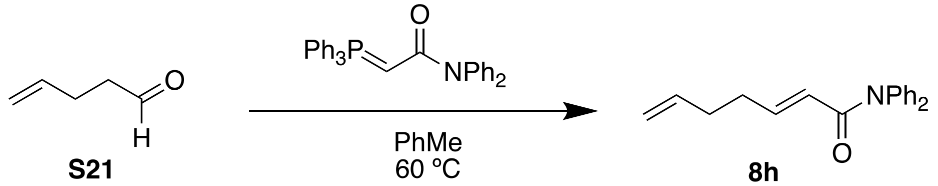

(*E*)-*N*,*N*-Diphenylhepta-2,6-dienamide (8h). To a stirring solution of 4-pentenal (0.510 g, 5.96 mmol) in PhMe (30.0 mL) was added *N*,*N*-diphenyl-2-(triphenylphosphaneylidene)acetamide (1.87 g, 3.97 mmol), synthesized by known procedures,[25] at 25 ºC. The reaction mixture was heated to 60 ºC in an oil bath for 16 h. The reaction mixture was cooled to 25 ºC and concentrated *in vacuo*. Purification by flash column chromatography (10:90 EtOAc:hexanes) afforded the product as a white solid as a single diastereomer (0.534 g, 49% to ylide). Relative configuration of the double bond was determined by analysis of *J* coupling constants:

^1H NMR (400 MHz, $CDCl_3$) δ 7.38–7.34 (m, 4H), 7.26–7.21 (m, 6H), 7.01 (dt, *J* = 15.1, 6.7 Hz, 1H), 5.87 (dt, *J* = 15.1, 1.4 Hz, 1H), 5.74 (m, 1H), 5.01–4.94 (m, 2H), 2.25–2.19 (m, 2H), 2.16–2.11 (m, 2H);

$^{13}C\{^1H\}$ NMR (100 MHz, $CDCl_3$) δ 166.2 (C), 146.3 (CH), 142.9 (CH), 137.5 (CH), 129.3 (CH), 127.8 (C), 126.9 (CH), 123.1 (CH), 115.5 (CH_2), 32.3 (CH_2), 31.7 (CH_2);
HRMS (ESI) *m / z* calcd for $C_{19}H_{19}NNaO$ $(M + Na)^+$ 300.1359, found 300.1364;
IR (ATR) 2981, 1667, 1489, 1349, 1253, 1156 cm^{-1};
mp = 91–92 ºC

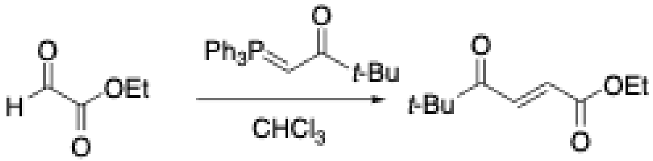

S13 S22

Ethyl (*E*)-5,5-dimethyl-4-oxohex-2-enoate (S22). According to a procedure,[16] to a stirring solution of 3,3-dimethyl-1-(triphenylphosphaneylidene)butan-2-one (9.01 g, 24.9 mmol), synthesized by known procedures,[26] in $CHCl_3$ (50.0 mL) was added ethyl 2-oxoacetate (50% in PhMe, 5.00 mL, 24.9 mmol) at 25 ºC. The reaction mixture was allowed to stir for 16 h. The reaction mixture was concentrated *in vacuo* and purified by flash column chromatography (5:95 EtOAc:hexanes) to afford the product as a light yellow oil (2.26 g, 49%) as a single diastereomer. The spectroscopic data were consistent with those reported:[27]
^1H NMR (400 MHz, $CDCl_3$) δ 7.50 (d, J = 15.5 Hz, 1H), 6.77 (d, J = 15.5 Hz, 1H), 4.26 (q, J = 7.1 Hz, 2H), 1.32 (t, J = 7.1 Hz, 3H), 1.19 (s, 9H);
$^{13}C\{^1H\}$ NMR (100 MHz, $CDCl_3$) δ 203.8 (C), 165.8 (C), 135.5 (CH), 131.6 (CH), 61.4 (CH_2), 43.7 (C), 25.9 (CH_3), 14.3 (CH_3).

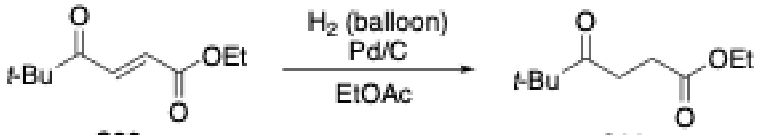

S22 S23

Ethyl 5,5-dimethyl-4-oxohexanoate (S23). According to a procedure,[19] Pd/C (0.360 g, 3.38 mmol) was added to a stirring solution of ethyl (*E*)-5,5-dimethyl-4-oxohex-2-enoate (2.26 g, 12.3 mmol) in EtOAc (123 mL). The reaction flask was purged with hydrogen using a balloon. The reaction mixture was allowed to stir under an atmosphere of hydrogen for 16 h. The reaction mixture was filtered over diatomaceous earth and concentrated *in vacuo* to afford the product as a clear oil (1.91 g, 84%) and was used in the next step without any further purification. The spectroscopic data were consistent with those reported:[28]
^1H NMR (400 MHz, $CDCl_3$) δ 4.12 (q, J = 7.1 Hz, 2H), 2.81 (t, J = 6.5 Hz, 2H), 2.55 (t, J = 6.5 Hz, 2H), 1.25 (t, J = 7.1 Hz, 3H), 1.16 (s, 9H);
$^{13}C\{^1H\}$ NMR (100 MHz, $CDCl_3$) δ 214.3 (C), 173.2 (C), 60.6 (CH_2), 44.1 (C), 31.6 (CH_2), 28.3 (CH_2), 26.6 (CH_3), 14.3 (CH_3).

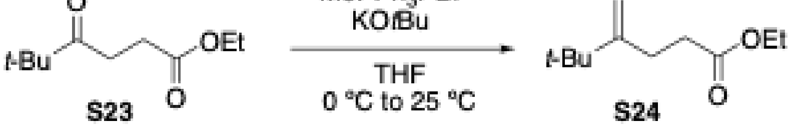

S23 S24

Ethyl 5,5-dimethyl-4-methylenehexanoate (S24). Ethyl 5,5-dimethyl-4-methylenehexanoate was prepared according to the general procedure for the synthesis of unsaturated esters. Ethyl 5,5-dimethyl-4-oxohexanoate (2.60 g, 14.0 mmol) in THF (14.0 mL) was mixed with methyltriphenylphosphonium bromide (7.50 g, 21.0 mmol) and potassium *tert*-butoxide (2.36 g, 21.0 mmol). Purification by flash column chromatography afforded the product as a clear oil (1.31 g, 51%). The spectroscopic data were consistent with those reported:[15]
^{1}H NMR (400 MHz, $CDCl_3$) δ 4.88 (s, 1H), 4.64 (s, 1H), 4.14 (q, *J* = 7.1 Hz, 2H), 2.49–2.45 (m, 2H), 2.39–2.36 (m, 2H), 1.25 (t, *J* = 7.1, 3H), 1.07 (s, 9H);
^{13}C{^{1}H} NMR (100 MHz, $CDCl_3$) δ 173.7 (C), 156.6 (C), 106.2 (CH_2), 60.4 (CH_2), 36.4 (C), 33.8 (CH_2), 29.4 (CH_3), 26.3 (CH_2), 14.4 (CH_3).

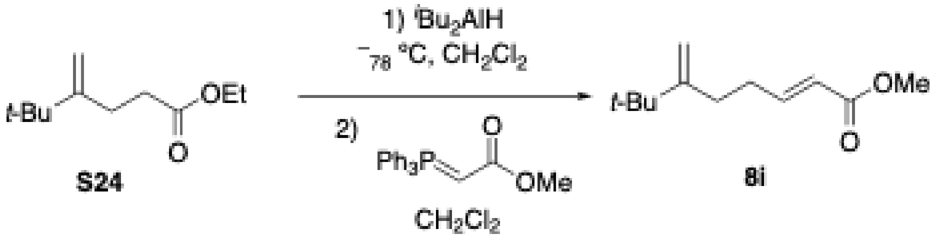

Methyl (*E*)-7,7-dimethyl-6-methyleneoct-2-enoate (8i). Methyl (*E*)-7,7-dimethyl-6-methyleneoct-2-enoate was prepared according to the general procedure for the synthesis of dienes. Diisobutylaluminum hydride (11.0 mL, 1.0 M in hexanes, 11 mmol) was mixed with ethyl 5,5-dimethyl-4-methylenehexanoate (1.77 g, 9.50 mmol) in CH_2Cl_2 (48.0 mL) at –78 ºC. The resulting unpurified aldehyde was used immediately in the next step. The unpurified aldehyde dissolved in CH_2Cl_2 (30.0 mL) was mixed with methyl(triphenylphosphoranylidene) acetate (6.35 g, 19.0 mmol). Purification by flash column chromatography (3:97 EtOAc:hexanes) afforded the product as a colorless oil as a single diastereomer (0.981 g, 53%). The configuration of the double bonds were determined by analysis of *J* coupling constants.
^{1}H NMR (400 MHz, $CDCl_3$) δ 7.01 (dt, *J* = 15.7, 6.8 Hz, 1H), 5.86 (dt, *J* = 15.7, 1.5 Hz, 1H), 4.91 (s, 1H), 4.68 (s, 1H), 3.73 (s, 3H), 2.38 (ddt, *J* = 15.2, 6.8, 1.5 Hz, 2H), 2.19 (m, 2H), 1.06 (s, 9H);
^{13}C{^{1}H} NMR (100 MHz, $CDCl_3$) δ 167.3 (C), 156.5 (C), 149.5 (CH), 121.1 (CH), 106.7 (CH_2), 51.5 (CH_3), 36.3 (C), 31.7 (CH_2), 29.5 (CH_2), 29.3 (CH_3);
HRMS (ESI) calcd for $C_{12}H_{20}NaO_2$ $(M + Na)^+$ 219.1356, found 219.1348;
HRMS (ESI) calcd for $C_{12}H_{21}O_2$ $(M + H)^+$ 197.1536, found 197.1532;
IR (ATR) 2966, 1724, 1435, 1268, 1153, 1059 cm^{-1}.

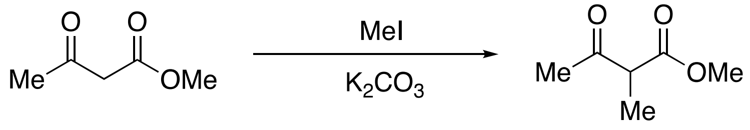

S25 **S26**

Methyl 2-methyl-3-oxobutanoate (S26). According to a procedure,[29] potassium carbonate (12.9 g, 93.2 mmol) was added slowly to a cooled (0 ºC) solution of iodomethane (9.69 g, 68.3 mmol) and methylacetoacetate (7.21 g, 62.1 mmol). The reaction mixture was stirred for 2 h and was

allowed to stir at 25 ºC for 16 h. The reaction mixture was filtered over diatomaceous earth, which was washed with Et_2O (50 mL), and concentrated *in vacuo*. Purification by flash column chromatography (10:90 EtOAc:hexanes) afforded methyl 2-methyl-3-oxobutanoate as a colorless oil (5.01 g, 62%). The spectroscopic data were consistent with those reported.[29]

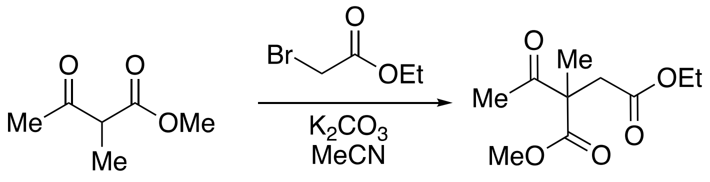

S26 **S27**

4-Ethyl-1-methyl 2-acetyl-2-methylsuccinate (S27). Potassium carbonate (10.6 g, 76.8 mmol) was added to a stirring solution of methyl 2-methyl-3-oxobutanoate (5.00 g, 38.4 mmol) and ethyl 2-bromoacetate (7.05 g, 42.2 mmol) in MeCN (80.0 mL) at 25 ºC. After 16 h, 1 M aqueous HCl (100 mL) was added to the reaction mixture and extracted with EtOAc (3 x 100 mL). The combined organics were washed with brine (100 mL), dried on $MgSO_4$, filtered, and concentrated *in vacuo*. Purification by flash column chromatography (10:90 EtOAc:hexanes) afforded 4-ethyl-1-methyl 2-acetyl-2-methylsuccinate as a colorless oil (6.82 g, 82%):

^{1}H NMR (400 MHz, $CDCl_3$) δ 4.11(q, J = 7.1 Hz, 2H), 3.75 (s, 3H), 2.88 (ABq, $\Delta\delta_{AB}$ = 0.08, J_{AB} = 16.4 Hz, 2H), 2.24 (s, 3H), 1.50 (s, 3H), 1.23 (t, J = 7.1 Hz, 3H);

^{13}C{^{1}H} NMR (100 MHz, $CDCl_3$) δ 204.6 (C), 172.5 (C), 170.9 (C), 60.9 (CH_2), 57.3 (C), 52.9 (CH_3), 40.2 (CH_2), 26.4 (CH_3), 20.3 (CH_3), 14.2 (CH_3);

HRMS (ESI) m/z calcd for $C_{10}H_{16}NaO_5$ $(M + Na)^+$ 239.0890, found 239.0891;

IR (ATR) 2986, 1732, 1716, 1371, 1197, 1155 cm^{-1}.

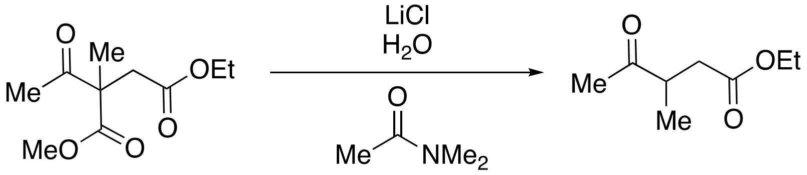

S27 **S28**

Ethyl 3-methyl-4-oxopentanoate (S28). A reported procedure was modified to prepare this compound.[30] LiCl (1.65 g, 39.0 mmol) was added to a stirring solution of ethyl-1-methyl 2-acetyl-2-methylsuccinate (4.22 g, 19.5 mmol) and H_2O (0.702 g, 39.0 mmol) in *N*,*N*-dimethylacetamide (20.0 mL) at 25 ºC. The reaction mixture was heated to 120 ºC in an oil bath for 16 h. The reaction mixture was cooled to 25 ºC and Et_2O (50 mL) was added to the reaction mixture. The reaction mixture was extracted with Et_2O (3 x 50 mL). The combined organic layers were washed with brine (100 mL), dried over $MgSO_4$, filtered, and concentrated *in vacuo*. Purification by flash column chromatography (15:85 EtOAc:hexanes) afforded ethyl 3-methyl-4-oxopentanoate as a light yellow oil (3.10 g, 97%). The spectroscopic data is consistent with those reported:[31]

^{1}H NMR (400 MHz, $CDCl_3$) δ 4.05 (q, J = 7.2 Hz, 2H), 3.01–2.98 (m, 1H), 2.75 (dd, J = 16.7, 8.6 Hz, 1H), 2.28 (dd, J = 16.7, 5.4 Hz, 1H), 2.21 (s, 3H), 1.24 (t, J = 7.2 Hz, 3H), 1.14 (d, J = 7.2 Hz, 3H);

$^{13}C\{^{1}H\}$ NMR (100 MHz, $CDCl_3$) δ 210.9 (C), 172.4 (C), 60.7 (CH_2), 42.9 (CH), 37.1 (CH_2), 28.5 (CH_3), 16.4 (CH_3), 14.3 (CH_3).

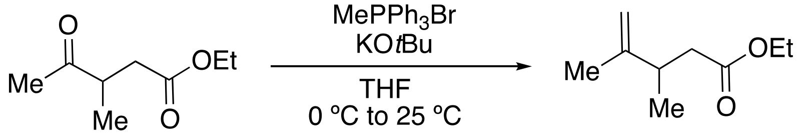

S28 S29

Ethyl 3,4-dimethylpent-4-enoate (S29). Ethyl 3,4-dimethylpent-4-enoate was prepared according to the general procedure for the synthesis of unsaturated esters. Ethyl 3-methyl-4-oxopentanoate (3.00 g, 19.0 mmol) in THF (20.0 mL) was mixed with methyltriphenylphosphonium bromide (8.82 g, 24.7 mmol) and potassium *tert*-butoxide (2.77 g, 24.7 mmol) in THF (65.0 mL). Purification by flash column chromatography (3:97 EtOAc:hexanes) afforded the product as a colorless oil (1.86 g, 63%). The spectroscopic data were consistent with those reported:[32]

^{1}H NMR (400 MHz, $CDCl_3$) δ 4.72 (s, 1H), 4.70 (s, 1H), 4.11 (q, J = 7.1 Hz, 2H), 2.67 (sext, J = 7.1 Hz, 1H), 2.43 (dd, J = 14.7, 7.1 Hz, 1H), 2.26 (dd, J = 14.7, 8.0 Hz, 1H), 1.72 (s, 3H), 1.24 (t, J = 7.2 Hz, 3H), 1.06 (d, J = 7.1 Hz, 3H);

$^{13}C\{^{1}H\}$ NMR (100 MHz, $CDCl_3$) δ 172.9 (C), 148.8 (C), 109.8 (CH_2), 60.3 (CH_2), 40.6 (CH_2), 37.9 (CH), 19.9 (CH_3), 19.5 (CH_3), 14.3 (CH_3);

HRMS (ESI) m / z calc for $C_9H_{17}O_2$ $(M + H)^+$ 157.1223, found 157.1229;

IR (ATR) 2966, 1735, 1444, 1369, 1162, 1096 cm^{-1}.

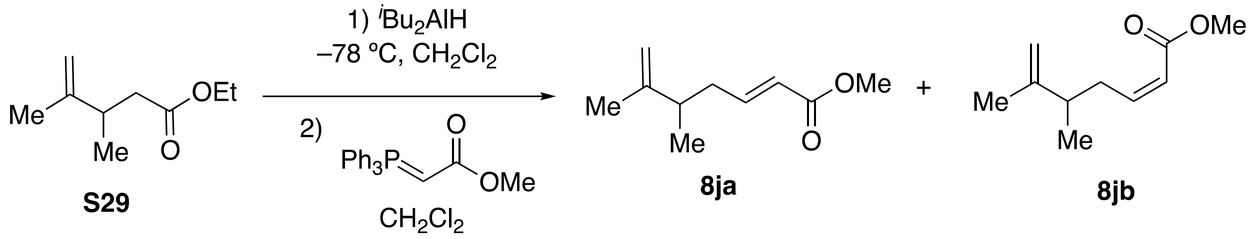

S29 8ja 8jb

Methyl (*E*)-5,6-dimethylhepta-2,6-dienoate (8ja) & Methyl (*Z*)-5,6-dimethylhepta-2,6-dienoate (8jb). Methyl (*E*)-5,6-dimethylhepta-2,6-dienoate & methyl (*Z*)-5,6-dimethylhepta-2,6-dienoate were prepared according to the general procedure for the synthesis of dienes. Diisobutylaluminum hydride (9.0 mL, 1.2 M in PhMe, 11 mmol) was mixed with Ethyl 3,4-dimethylpent-4-enoate (1.40 g, 9.85 mmol) in CH_2Cl_2 (50.0 mL). The resulting unpurified aldehyde was used immediately in the next step. The unpurified aldehyde in CH_2Cl_2 (50.0 mL) was mixed with methyl(triphenylphosphoranylidene) acetate (4.95 g, 14.8 mmol). Purification by flash column chromatography (3:97 EtOAc:hexanes) afforded the products as a colorless oil as a mixture of E:Z isomers in a 95:5 ratio (0.873 g, 53%). The relative configuration of the double bonds were determined by analysis of J coupling constants. Characterization was performed as a mixture of E:Z isomers (95:5):

Peaks attributed to diene **8ja**: ^{1}H NMR (400 MHz, $CDCl_3$) δ 6.88 (dt, J = 15.6, 7.2 Hz, 1H), 5.81 (dt, J = 15.6, 1.2 Hz, 1H), 4.73–4.70 (m, 2H), 3.72 (s, 3H), 2.35–2.26 (m, 2H), 2.23–2.14 (m, 1H), 1.68 (s, 3H), 1.04 (d, J = 6.6 Hz, 3H);

$^{13}C\{^1H\}$ NMR (100 MHz, $CDCl_3$) δ 167.1 (C), 148.7 (C), 148.2 (CH), 122.0 (CH), 110.3 (CH_2), 51.5 (CH_3), 40.4 (CH), 37.9 (CH_2), 19.7 (CH_3), 19.4 (CH_3);
Peaks attributed to diene **8jb**:^1H NMR (400 MHz, $CDCl_3$, characteristic peaks) δ 6.18 (dt, J = 11.6, 7.3 Hz, 1H), 3.70 (s, 3H), 2.73 (td, J = 7.3, 1.7 Hz, 2H);
$^{13}C\{^1H\}$ NMR (100 MHz, $CDCl_3$, characteristic peaks) δ 167.0 (C), 149.5 (CH), 149.1 (C), 119.8 (CH), 110.2 (CH_2), 51.1 (CH_3), 41.1 (CH);
HRMS (ESI) *m / z* calc for $C_{10}H_{16}NaO_2$ $(M + Na)^+$ 191.1043, found 191.1050;
IR (ATR) 2968, 1726, 1650, 1433, 1320, 1183 cm^{-1}.

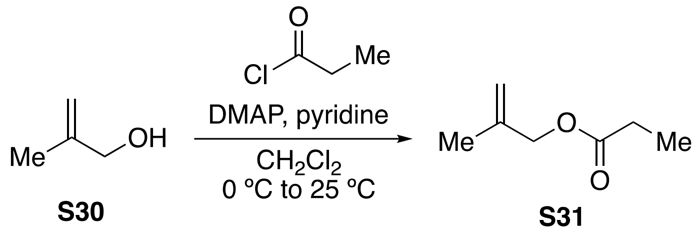

2-Methylallyl propionate (S31). To a cooled (0 ºC) solution of 2-methyl-2-propen-1-ol (3.50 g, 48.5 mmol), DMAP (0.592 g, 4.85 mmol), and pyridine (7.69 g, 97.0 mmol) in CH_2Cl_2 (100 mL) was added propionyl chloride (5.40 g, 58.2 mmol) dropwise by syringe. After complete addition, the reaction mixture was allowed to stir at 25 ºC for 16 h. Saturated aqueous $NaHCO_3$ (100 mL) was added to the reaction mixture and the resulting mixture was extracted with CH_2Cl_2 (3 x 100 mL). The combined organic layers were washed with aqueous 1 M HCl (100 mL) and brine (100 ml), dried over $MgSO_4$, filtered, and concentrated *in vacuo* to afford the product as a clear oil (6.20 g, 99%). The product was used without any further purification:
^1H NMR (400 MHz, $CDCl_3$) δ 4.96 (s, 1H), 4.91 (s, 1H), 4.50 (s, 2H), 2.37 (q, J = 7.6 Hz, 2H), 1.75 (s, 3H), 1.16 (t, J = 7.6 Hz, 3H);
$^{13}C\{^1H\}$ NMR (400 MHz, $CDCl_3$) δ 174.3 (C), 140.2 (C), 112.8 (CH_2), 67.7 (CH_2), 27.7 (CH_2), 19.6 (CH_3), 9.3 (CH_3);
HRMS (ESI) *m / z* calcd for $C_7H_{16}NO_2$ $(M + NH_4)^+$ 146.1176, found 146.1170;
IR (ATR) 2980, 2944, 1738, 1463, 1271, 1173 cm^{-1}.

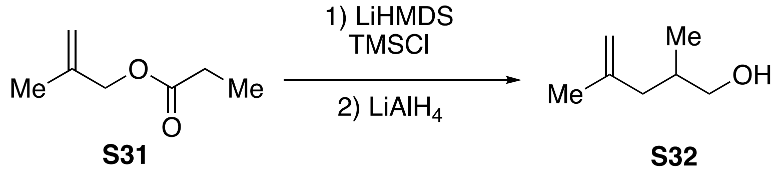

2,4-Dimethylpent-4-en-1-ol (S32). To a cooled (-78 ºC) solution of 2-methylallyl propionate (1.20 g, 9.36 mmol) and trimethylsilyl chloride (TMSCl, 1.52 g, 14.0 mmol) in THF (14.0 mL) was added dropwise lithium hexamethyldisilazide (LiHMDS, 14.0 mL, 1.0 M in THF, 14 mmol). After complete addition, the reaction mixture was warmed to 25 ºC and stirred for 1 h. The reaction mixture was then heated to 60 ºC in an oil bath for 16 h. The reaction mixture was cooled to 25 ºC and 25 mL of aqueous 1 M HCl was added. The reaction mixture was extracted with CH_2Cl_2 (3 x 30 mL), washed with brine (50 mL), dried over $MgSO_4$, filtered, and

concentrated *in vacuo* to afford the unpurified carboxylic acid as a yellow oil, which was used immediately in the next step without any further purification.

To a cooled (0 ºC) solution of the unpurified carboxylic acid in THF (10.0 mL) was added $LiAlH_4$ (0.781 g, 20.6 mmol) in four portions. Upon complete addition, the reaction mixture was allowed to stir for 2 h at 25 ºC. EtOAc (20 mL) and aqueous 1 M NaOH (20 mL) was added to the reaction mixture dropwise and the resulting mixture extracted with Et_2O (3 x 30 mL). The combined organic layers were washed with brine (50 mL), dried over Mg_2SO_4, filtered, and concentrated *in vacuo*. Purification by flash column chromatography (1:2 Et_2O:hexanes) afforded the product as a colorless oil (0.936 g, 88% over two steps). The spectroscopic data were consistent with those reported:[33]

^{1}H NMR (400 MHz, $CDCl_3$) δ 4.77 (s, 1H), 4.72 (s, 1H), 3.52 (dd, *J* = 10.6, 5.6 Hz, 1H), 3.45 (dd, *J* = 10.6, 5.8 Hz, 1H), 2.17–2.10 (m, 1H), 1.93–1.81 (m, 2H), 1.73 (s, 3H), 0.90 (s, 3H); ^{13}C{^{1}H} NMR (400 MHz, $CDCl_3$) δ 144.7 (C), 111.9 (CH_2), 68.6 (CH_2), 42.5 (CH_2), 33.7 (CH), 22.4 (CH_3), 16.8 (CH_3).

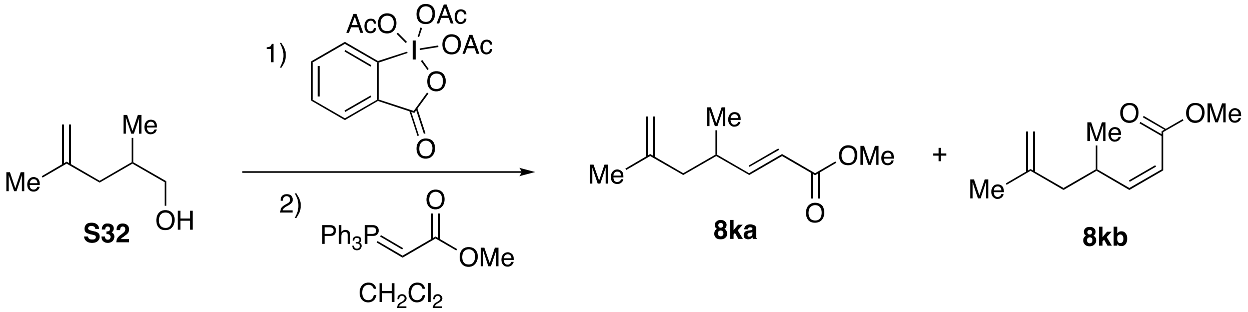

Methyl (*E*)-4,6-dimethylhepta-2,6-dienoate (8ka) & Methyl (*Z*)-4,6-dimethylhepta-2,6-dienoate (8kb). To a stirring solution of 2,4-dimethylpent-4-en-1-ol (1.82 g, 15.9 mmol) in CH_2Cl_2 (106 mL) was added Dess–Martin periodinane (8.10 g, 19.1 mmol) in one portion and the reaction mixture was allowed to stir at 25 ºC for 3 h. Saturated aqueous $NaHCO_3$ (50.0 mL) and saturated aqueous NaS_2O_3 (50.0 mL) were added to the reaction mixture and the reaction mixture was allowed to stir at 25 ºC for 1 h. The reaction mixture was extracted with CH_2Cl_2 (3 x 50 mL). The combined organic phases were washed with brine (100 mL), dried over Na_2SO_4, filtered, and concentrated *in vacuo*. The unpurified aldehyde was used immediately in the next step without any further purification. To a stirring solution of unpurified aldehyde in CH_2Cl_2 (29.0 mL) was added methyl(triphenylphosphoranylidene) acetate (5.85 g, 17.5 mmol). The reaction mixture was allowed to stir at 25 ºC for 16 h. The reaction mixture was then concentrated *in vacuo*. Purification by flash column chromatography (3:97 EtOAc:hexanes) afforded the product as a clear oil as a mixture of *E*:*Z* isomers in a 97:3 ratio (1.15 g, 36% over two steps). The relative configuration of the double bonds were determined by analysis of *J* coupling constants. Characterization was performed as a mixture of *E*:*Z* isomers (97:3):

Peaks attributed to diene **8ka**: ^{1}H NMR (400 MHz, $CDCl_3$) δ 6.90 (dd, *J* = 15.7, 7.4 Hz, 1H), 5.79 (dd, *J* = 15.7, 1.3 Hz, 1H), 4.77 (s, 1H), 4.68 (s, 1H), 3.72 (s, 3H), 2.57–2.46 (m, 1H), 2.12 (dd, *J* = 13.8, 7.2 Hz, 1H), 2.02 (dd, *J* = 13.8, 7.2 Hz, 1H), 1.69 (s, 3H), 1.03 (d, *J* = 6.7 Hz, 3H);

$^{13}C\{^1H\}$ NMR (100 MHz, $CDCl_3$) δ 167.4 (C), 154.5 (CH), 143.1 (C), 119.3 (CH), 112.7 (CH_2), 51.6 (CH_3), 44.6 (CH_2), 34.4 (CH), 22.3 (CH_3), 19.1 (CH_3);
Peaks attributed to diene **8kb**: ^1H NMR (400 MHz, $CDCl_3$, characteristic peaks) δ 4.92 (s, 1H), 4.84 (s, 1H), 2.79–2.74 (m, 1H), 2.38 (dd, J = 14.2, 8.2 Hz, 1H), 2.22 (dd, J = 14.2, 8.2 Hz, 1H), 1.32 (d, J = 7.0 Hz, 3H);
$^{13}C\{^1H\}$ NMR (100 MHz, $CDCl_3$, characteristic peaks) δ 156.2 (CH), 118.0 (CH), 114.3 (CH_2), 42.2 (CH_2), 22.1 (CH_3), 17.9 (CH_3);
HRMS (ESI) m / z calcd for $C_{10}H_{16}NaO_2$ $(M + Na)^+$ 191.1043, found 191.1050;
IR (ATR) 2970, 1722, 1654, 1435, 1313, 1193 cm^{-1}.

III. Synthesis of Cobalt Catalysts

(S,S)-N,N'-Bis(3,5-di-tert-butylsalicylidene)-1,2-cyclohexanediaminocobalt(II) ($Co(salen)_2$, **12**) and 5,10,15,20-Tetraphenyl-21H, 23H-porphinecobalt(II) (Co^{II}(tpp), **14**) were purchased from AmBeed and used without further purification. Bis(2,4-pentanedionato)cobalt(II) ($Co(acac)_2$, **13**) was purchased from TCI America and used without further purification.

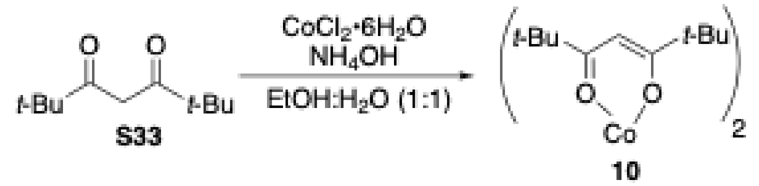

Bis(2,2,6,6-tetramethylheptan-3,5,-dionato)cobalt(II) ($Co(thd)_2$, 10). According to a procedure,[34] 2,2,6,6-tetramethyl-3,5-dione (1.88 g, 10.1 mmol) was added to a stirring solution of cobalt(II) chloride hexahydrate (1.20 g, 5.04 mmol) in 50% aqueous ethanol (14.0 mL). Ammonium hydroxide (50% v/v aqueous solution, 0.400 mL, 10.1 mmol) was added dropwise to the reaction mixture. After 1 h, H_2O (28 mL) was added to the reaction mixture and stirred. After 1 h, the reaction mixture was filtered, and the resulting solid was washed with H_2O (3 x 40 mL). The solid was dried at 25 ºC and atmospheric pressure for 18 h to yield $Co(thd)_2$ **10** as a pink solid (1.62 g, 76%) and was used without further purification. The spectroscopic data were consistent with those reported:[35]

^1H NMR (400 MHz, $(CD_3)_2SO$) δ 5.74 (s, 2H), 1.18 (s, 36H);
HRMS (ESI) m / z calcd for $C_{22}H_{39}CoO_4$ $(M + H)^+$ 426.2175, found 426.2164;
HRMS (ESI) m / z calcd for $C_{22}H_{42}CoNO_4$ $(M + NH_4)^+$ 433.2440, found 433.2446.
mp = 101–103 ºC

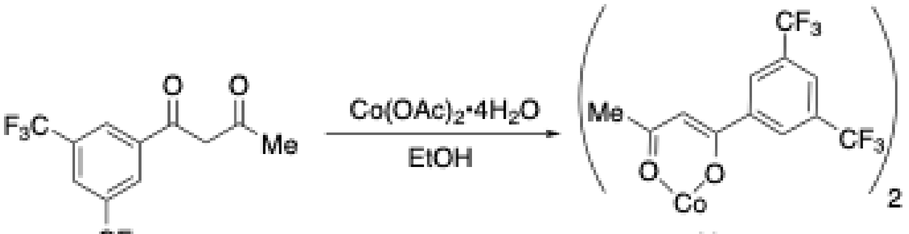

S34 11

Bis-{4-[3,5-bis-(trifluoromethyl)-phenyl]-(2-oxo-κ*O*)-but-3-en-(4-olato-κ*O*)}-cobalt(II) (Co(aryl-CF_3)$_2$, 11). According to a procedure,[36] cobalt(II) acetate tetrahydrate (0.0820 g, 0.330 mmol) was added to a stirring solution of 1-(3,5-bis(trifluoromethyl)phenyl)butane-1,3-dione (0.200 g, 0.671 mmol), synthesized from known procedures,[36] in ethanol (2.70 mL) at 25 ºC. After 1 h, the reaction mixture was filtered, and the resulting solid was washed with ethanol that was cooled in an ice-water bath (3 x 5 mL). The solid was dried at 25 ºC under vacuum for 3 h to yield Co(aryl-CF_3)$_2$ **11** as a yellow-orange solid (0.187 g, 87%) and was used without any further purification. ^{1}H and ^{13}C{^{1}H} NMR data could not be obtained. The ^{19}F{^{1}H} NMR spectroscopic data was consistent with those reported:[37]

^{19}F{^{1}H} NMR (471 MHz, (($(CD_3)_2CO$:$CDCl_3$ (1:1 v/v)) δ –56.3 (s);
HRMS (ESI) *m* / *z* calcd for $C_{24}H_{14}CoF_{12}KO_4$ (M + K)$^+$ 691.9664, found 691.9690;
mp= 101–103 ºC.

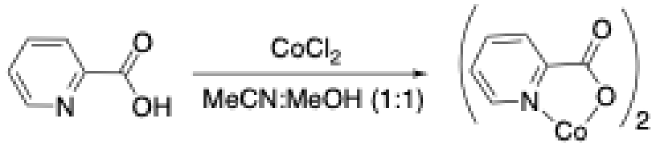

15

Cobalt(II) picolinate (Co(pic)$_2$, 15). According to a procedure,[3] to a stirring solution of anhydrous cobalt(II) chloride (1.62 g, 12.5 mmol) in acetonitrile (16.0 mL) was added a solution of picolinic acid (3.85 g, 31.3 mmol) in methanol (16 mL) at 25 ºC. After 16 h, the reaction mixture was filtered, and the resulting solid was washed with Et_2O (3 x 30 mL). The solid was dried at 25 ºC under vacuum for 3 h to yield Co(pic)$_2$ **15** as a pale-pink solid (3.66 g, 97%) and was used without any further purification. The NMR characterization data refers to peaks observed. The spectroscopic data were consistent with those reported:[38]

^{1}H NMR (400 MHz, $CDCl_3$) δ 74.70, 66.47, 47.76, 23.08, 8.07;
HRMS (ESI) *m* / *z* calcd for $C_{12}H_{12}CoN_3O_4$ (M + NH_4)$^+$ 321.0154, found 321.0142;
mp = > 225 ºC.

IV. Synthesis of Unsaturated Hydroperoxides

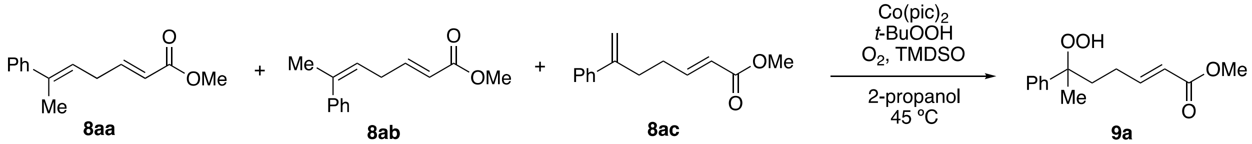

8aa 8ab 8ac 9a

Unsaturated Hydroperoxide 9a. Unsaturated hydroperoxide **9a** was prepared according to the general procedure for the synthesis of unsaturated hydroperoxides. Dienes **8aa**, **8ab** and, **8ac** in 2-propanol (61.0 mL) was mixed with Co(pic)$_2$ (0.168 g, 0.555 mmol), TMDSO (1.09 g, 8.14 mmol), and *t*BuOOH (1.0 M in CH_2Cl_2, 0.740 mL, 0.74 mmol) under an atmosphere of oxygen for 4 h. Purification by flash column chromatography (15:85 EtOAc:hexanes) afforded unsaturated hydroperoxide **9a** as a colorless, viscous oil (0.420 g, 45 %):
^{1}H NMR (400 MHz, C_6D_6) δ 7.22–7.20 (m, 2H), 7.14–7.04 (m, 3H), 6.92 (dt, *J* = 15.6, 6.8 Hz, 1H), 5.74 (dt, *J* = 15.6, 1.6 Hz, 1H), 3.41 (s, 3H), 1.97–1.85 (m, 2H), 1.75–1.67 (m, 2H), 1.37 (s, 3H);
^{13}C{^{1}H} NMR (100 MHz, C_6D_6) δ 166.7 (C), 149.1 (CH), 144.4 (C), 128.6 (CH), 127.4 (CH), 125.8 (CH), 121.2 (CH), 85.2 (C), 51.0 (CH_3), 37.7 (CH_2), 26.9 (CH_2), 23.6 (CH_3);
HRMS (ESI) *m / z* calcd for $C_{14}H_{18}NaO_4$ $(M + Na)^+$ 273.1097, found 273.1093.

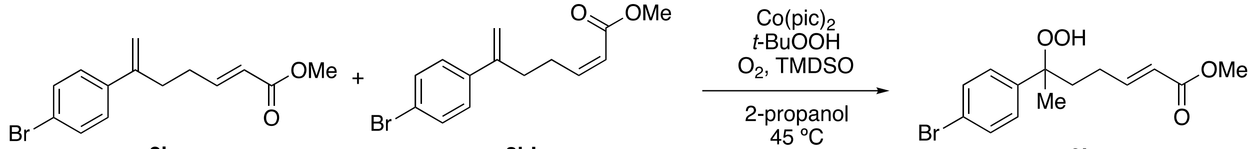

8ba 8bb 9b

Unsaturated Hydroperoxide 9b. Hydroperoxide **9b** was prepared according to the general procedure for the synthesis of unsaturated hydroperoxides. Dienes **8ba** and **8bb** (0.800 g, 2.71 mmol), as mixture of *E*:*Z* isomers in a 88:12 ratio, in 2-propanol (45.0 mL) was mixed with Co(pic)$_2$ (0.123 g, 0.407 mmol), TMDSO (1.10 mL, 5.96 mmol), and *t*-BuOOH (1.0 M, 0.550 mL, 0.55 mmol) under an atmosphere of oxygen for 2 h. Purification by flash column chromatography (25:75 EtOAc:hexanes) on Davisil® grade silica gel afforded unsaturated hydroperoxide **9b** as a viscous colorless oil (0.282 g, 31%). A sample analyte was used for the characterization of unsaturated hydroperoxide **9b**:
^{1}H NMR (400 MHz, $CDCl_3$) δ 7.51 (d, *J* = 8.5 Hz, 2H), 7.33 (s, br, 1H), 7.29 (d, *J* = 8.6 Hz, 2H), 6.89 (dt, *J* = 15.7, 6.8 Hz, 1H), 5.78 (dt, *J* = 15.7, 1.7 Hz, 1H), 3.71 (s, 3H), 2.18–2.14 (m, 2H), 1.98–1.94 (m, 2H), 1.63 (s, 3H);
^{13}C{^{1}H} NMR (100 MHz, $CDCl_3$) δ 167.0 (C), 148.6 (CH), 142.7 (C), 131.9 (CH), 127.6 (CH), 121.8 (C), 121.3 (CH), 85.5 (C), 51.6 (CH_3), 37.7 (CH_2), 26.8 (CH_2), 23.2 (CH_3);
HRMS (ESI) calcd for $C_{14}H_{16}BrO_3$ $[(M + H) – H_2O]^+$ 311.0277, found 311.0273;
HRMS (ESI) calcd for $C_{14}H_{17}BrNaO_4$ $(M + Na)^+$ 351.0202, found 351.0204.

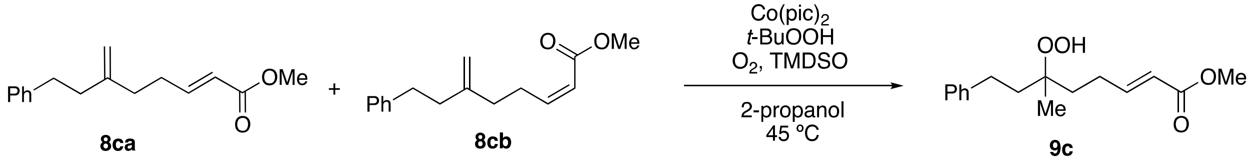

8ca 8cb 9c

Unsaturated Hydroperoxide 9c. Unsaturated hydroperoxide **9c** was prepared according to the general procedure for the synthesis of unsaturated hydroperoxides. Dienes **8ca** and **8cb** (0.500 g, 2.05 mmol), as a mixture of *E*:*Z* isomers in a 95:5 ratio, in 2-propanol (34.0 mL) was mixed with

Co(pic)$_2$ (0.0310 g, 0.103 mmol), TMDSO (0.800 mL, 4.51 mmol), and *t*-BuOOH (1.0 M, 0.410 mL, 0.41 mmol) under an atmosphere of oxygen for 6 h. Purification by flash column chromatography afforded **9c** as a viscous colorless oil (0.309 g, 54%):

^{1}H NMR (400 MHz, CDCl$_3$) δ 7.31–7.27 (m, 2H), 7.22–7.18 (m, 3H), 7.11–7.10 (m, 1H), 7.01 (dt, J = 15.7, 6.9 Hz, 1H), 5.86 (dt, J = 15.7, 1.5 Hz, 1H), 3.73 (s, 3H), 2.68–2.64 (m, 2H), 2.32–2.26 (m, 2H), 1.97–1.68 (m, 4H), 1.26 (s, 3H);

^{13}C{^{1}H} NMR (100 MHz, CDCl$_3$) δ 167.3 (C), 149.4 (CH), 142.4 (C), 128.6 (CH), 128.5 (CH), 126.1 (CH), 121.1 (CH), 84.0 (C), 51.6 (CH$_3$), 38.8 (CH$_2$), 35.1 (CH$_2$), 30.1 (CH$_2$), 26.6 (CH$_2$), 21.4 (CH$_3$);

HRMS (ESI) calcd for $C_{16}H_{22}NaO_4$ (M + Na)$^+$ 301.1410, found 301.1414.

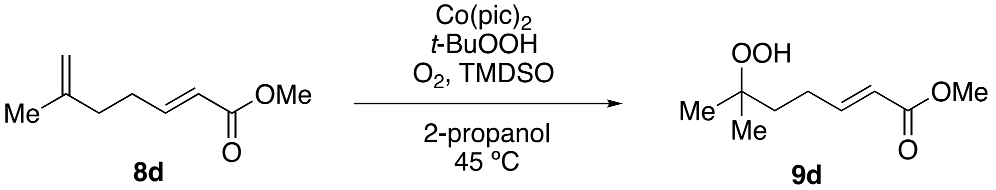

Unsaturated Hydroperoxide 9d. Unsaturated hydroperoxide **9d** was prepared according to the general procedure for the synthesis of unsaturated hydroperoxides. Diene **8d** (1.32 g, 8.56 mmol) in 2-propanol (143 mL) was mixed with Co(pic)$_2$ (0.130 g, 0.428 mmol), TMDSO (2.49 g, 18.8 mmol), and *t*-BuOOH (1.0 M, 1.70 mL, 1.7 mmol) under an atmosphere of oxygen for 6 h. Purification by flash column chromatography (15:85 EtOAc:hexanes) afforded the product as a colorless viscous oil (0.680 g, 42%):

^{1}H NMR (500 MHz, C_6D_6) δ 7.13–7.00 (m, 2H), 5.85 (dt, J = 15.6, 1.5 Hz, 1H), 3.43 (s, 3H), 1.98–1.93 (m, 2H), 1.42–1.39 (m, 2H), 0.98 (s, 6H);

^{13}C{^{1}H} NMR (150 MHz, C_6D_6) δ 167.0 (C), 149.8 (CH), 121.1 (CH), 81.3 (C), 51.0 (CH$_3$), 36.6 (CH$_2$), 26.9 (CH$_2$), 24.0 (CH$_3$);

HRMS (ESI) m / z calcd for $C_9H_{16}NaO_4$ (M + Na)$^+$ 211.0941, found 211.0934.

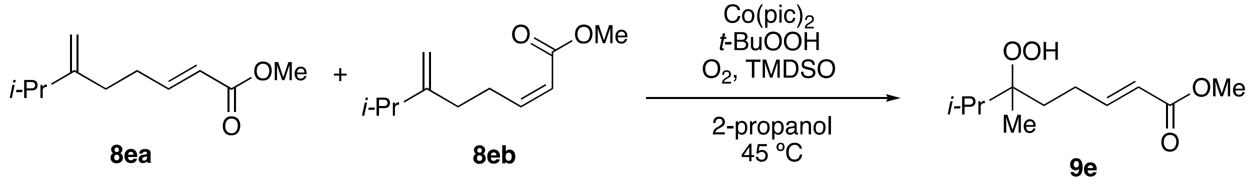

Unsaturated Hydroperoxide 9e. Unsaturated hydroperoxide **9e** was prepared according to the general procedure for the synthesis of unsaturated hydroperoxides. Diene **8ea** and **8eb** (0.600 g, 3.29 mmol), as a mixture of *E*:*Z* isomers in a 94:6 ratio, in 2-propanol (55.0 mL) was mixed with Co(pic)$_2$ (0.0500 g, 0.165 mmol), TMDSO (1.30 mL, 7.24 mmol), and *t*-BuOOH (1.0 M, 0.660 mL, 0.66 mmol) under an atmosphere of oxygen for 6 h. Purification by flash column chromatography (15:85 EtOAc:hexanes) afforded **9e** as a viscous colorless oil (0.105 g, 27%):

^{1}H NMR (400 MHz, C_6D_6) δ 7.09 (dt, J = 15.6, 7.0 Hz, 1H), 6.44 (s, 1H), 5.90 (dt, J = 15.6, 1.5 Hz, 1H), 3.46 (s, 3H), 2.16–1.86 (m, 3H), 1.55–1.48 (m, 1H), 1.45–1.38 (m, 1H), 0.85 (d, J = 6.8 Hz, 3H), 0.82 (s, 3H), 0.68 (d, J = 6.8 Hz, 3H);

$^{13}C\{^1H\}$ NMR (100 MHz, C_6D_6) δ 166.8 (C), 150.0 (CH), 121.2 (CH), 85.6 (C), 51.0 (CH_3), 33.1 (CH_2), 32.8 (CH), 26.3 (CH_2), 17.7 (CH_3), 17.0 (CH_3), 16.7 (CH_3);
HRMS (ESI) *m / z* calcd for $C_{11}H_{20}NaO_4$ $(M + Na)^+$ 239.1254, found 239.1256.

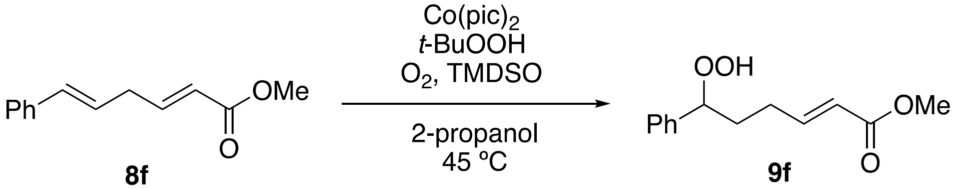

Unsaturated Hydroperoxide 9f. Unsaturated hydroperoxide **9f** was prepared according to the general procedure for the synthesis of unsaturated hydroperoxides. Diene **8f** (0.600 g, 2.97 mmol) in 2-propanol (50.0 mL) was mixed with $Co(pic)_2$ (0.0450 g, 0.149 mmol), TMDSO (0.877 g, 6.53 mmol), and *t*-BuOOH (1.0 M in CH_2Cl_2, 0.600 mL, 0.60 mmol) under an atmosphere of oxygen for 5 h. Purification by flash column chromatography (15:85 EtOAc:hexanes) afforded unsaturated hydroperoxide **9f** as a viscous, clear oil (0.436 g, 62%):
^1H NMR (400 MHz, $CDCl_3$) δ 7.87–7.78 (br s, 1H), 7.41–7.32 (m, 5H), 6.95 (dt, J = 15.7, 7.3 Hz, 1H), 5.83 (dt, J = 15.7, 1.5 Hz, 1H), 4.92 (t, J = 6.9 Hz, 1H), 3.72 (s, 3H), 2.31–2.23 (m, 2H), 2.07–2.01 (m, 1H), 1.87–1.79 (m, 1H);
$^{13}C\{^1H\}$ NMR (100 MHz, $CDCl_3$) δ 167.1 (C), 148.2 (CH), 140.0 (C), 128.9 (CH), 128.7 (CH), 127.0 (CH), 121.7 (C), 87.2 (CH), 51.6 (CH_3), 32.7 (CH_2), 28.5 (CH_2);
HRMS (ESI) *m / z* calcd for $C_{13}H_{20}NO_4$ $(M + NH_4)^+$ 254.1387, found 254.1396.

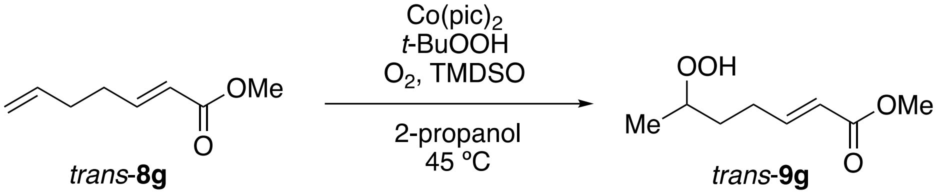

Unsaturated Hydroperoxide *trans*-9g. Unsaturated hydroperoxide *trans*-**9g** was prepared according to the general procedure for the synthesis of unsaturated hydroperoxides. Diene *trans*-**8g** (0.400 g, 2.85 mmol) in 2-propanol (48.0 mL) was mixed with $Co(pic)_2$ (0.0430 g, 0.143 mmol), TMDSO (0.842 g, 6.27 mmol), and *t*-BuOOH (1.0 M in CH_2Cl_2, 0.570 mL, 0.57 mmol) under an atmosphere of oxygen for 16 h. Purification by flash column chromatography (15:85 EtOAc:hexanes) afforded unsaturated hydroperoxide *trans*-**9g** as a viscous, clear oil (0.188 g, 38%). The spectroscopic data were consistent with those reported:[39]
^1H NMR (400 MHz, C_6D_6) δ 7.94–7.93 (br s, 1H), 6.97 (dt, J = 15.7, 6.9 Hz, 1H), 5.85 (dt, J = 15.7, 1.6 Hz, 1H), 4.12–4.05 (m, 1H), 3.72 (s, 3H), 2.33–2.27 (m, 2H), 1.83–1.74 (m, 1H), 1.66–1.57 (m, 1H), 1.24 (d, J = 6.2 Hz, 3H);
$^{13}C\{^1H\}$ NMR (100 MHz, C_6D_6) δ 167.3 (C), 149.0 (CH), 121.4 (C), 80.7 (CH), 51.6 (CH_3), 32.3 (CH_2), 28.2 (CH_2), 18.2 (CH_3);
HRMS (ESI) *m / z* calcd for $C_8H_{13}O_3$ $[(M + H) - H_2O]^+$ 157.0859, found 157.0864.

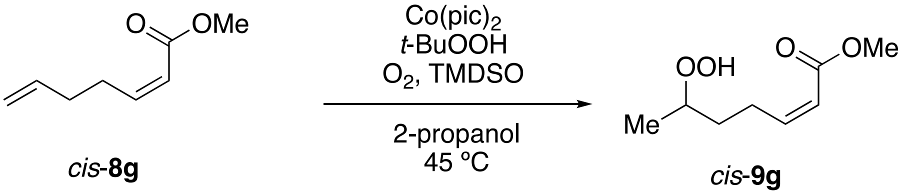

cis-**8g** *cis*-**9g**

Unsaturated Hydroperoxide *cis*-9g. Unsaturated hydroperoxide *cis*-**9g** was prepared according to the general procedure for the synthesis of unsaturated hydroperoxides. Diene *cis*-**8g** (0.286 g, 2.04 mmol) in 2-propanol (34.0 mL) was mixed with $Co(pic)_2$ (0.0310 g, 0.102 mmol), TMDSO (0.603 g, 4.49 mmol), and *t*-BuOOH (1.0 M in CH_2Cl_2, 0.410 mL, 0.41 mmol) under an atmosphere of oxygen for 16 h. Purification by flash column chromatography (15:85 EtOAc:hexanes) afforded unsaturated hydroperoxide *cis*-**9g** as a viscous, clear oil (0.130 g, 37%):

^{1}H NMR (400 MHz, C_6D_6) δ 8.08–8.03 (br s, 1H), 5.86 (dt, *J* = 11.4, 7.9 Hz, 1H), 5.70 (dt, *J* = 11.4, 1.3 Hz, 1H), 3.89 (sext, *J* = 6.2 Hz, 1H), 3.29 (s, 3H), 2.73–2.58 (m, 2H), 1.61–1.26 (m, 4H), 1.09 (d, *J* = 6.3 Hz, 3H);

$^{13}C\{^1H\}$ NMR (100 MHz, C_6D_6) δ 166.9 (C), 150.6 (CH), 119.9 (C), 80.4 (CH), 50.9 (CH_3), 33.3 (CH_2), 24.9 (CH_2), 18.2 (CH_3);

HRMS (ESI) *m* / *z* calcd for $C_8H_{15}O_4$ $(M + H)^+$ 175.0965, found 175.0966.

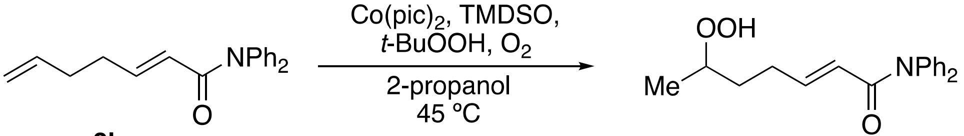

8h **9h**

Unsaturated Hydroperoxide 9h. Unsaturated hydroperoxide **9h** was prepared according to the general procedure for the synthesis of unsaturated hydroperoxides. Diene **8h** (0.429 g, 1.55 mmol) in 2-propanol (26.0 mL) was mixed with $Co(pic)_2$ (0.0230 g, 0.0775 mmol), TMDSO (0.458 g, 3.41 mmol), and *t*-BuOOH (1.0 M, 0.310 mL, 0.31 mmol) under an atmosphere of oxygen for 16 h. Purification by flash column chromatography (50:50 EtOAc:hexanes) afforded the product as a yellow viscous oil (0.166 g, 53%):

^{1}H NMR (400 MHz, C_6D_6) δ 7.74 (s, 1H), 7.27 (dt, *J* = 15.1, 7.4 Hz, 1H), 5.95 (dt, *J* = 15.1, 1.5 Hz, 1H), 7.12 (br s, 3H), 7.02 (t, *J* = 7.7 Hz, 5H), 6.92–6.88 (m, 2H), 5.95 (dt, *J* = 15.1, 1.5 Hz, 1H), 3.76 (sext, *J* = 6.2 Hz, 1H), 1.93–1.87 (m, 2H), 1.56–1.47 (m, 1H), 1.29–1.20 (m, 1H), 0.96 (d, *J* = 6.2 Hz, 3H);

$^{13}C\{^1H\}$ NMR (100 MHz, C_6D_6) δ 165.7 (C), 146.8 (CH), 143.6 (C), 129.3 (CH), 127.8 (CH), 126.5 (CH), 123.5 (CH), 80.1 (CH), 32.8 (CH_2), 28.5 (CH_2), 18.2 (CH_3);

HRMS (ESI) calcd for $C_{19}H_{22}NO_3$ $(M + H)^+$ 313.1627, found 313.1630.

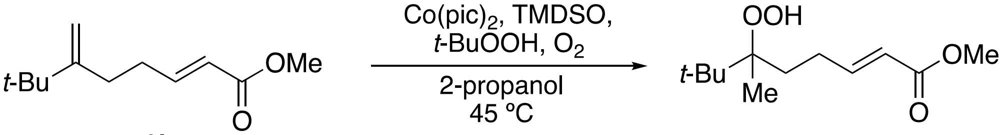

8i **9i**

Unsaturated Hydroperoxide 9i. Unsaturated hydroperoxide **9i** was prepared according to the general procedure for the synthesis of unsaturated hydroperoxides. Diene **8i** (0.500 g, 2.55 mmol) in 2-propanol (43.0 mL) was mixed with Co(pic)$_2$ (0.0390 g, 0.128 mmol), TMDSO (0.990 mL, 5.61 mmol), and *t*-BuOOH (1.0 M, 0.510 mL, 0.51 mmol) under an atmosphere of oxygen for 6 h. Purification by flash column chromatography (15:85 EtOAc:hexanes) afforded X as a viscous colorless oil (0.0350 g, 15%):

^{1}H NMR (400 MHz, C_6D_6) δ 7.37 (br, 1H), 7.11 (dt, *J* = 15.6, 6.9 Hz, 1H), 5.90 (dt, *J* = 15.6, 1.5 Hz, 1H), 3.44 (s, 3H), 2.28–2.23 (m, 1H), 2.06–1.96 (m, 1H), 1.91–1.84 (m, 1H), 1.35–1.27 (m, 1H), 0.94–0.93 (m, 12H);

$^{13}C\{^1H\}$ NMR (100 MHz, C_6D_6) δ 167.2 (C), 150.6 (CH), 121.0 (CH), 87.2 (C), 51.1 (CH_3), 38.4 (C), 31.6 (CH_2), 27.9 (CH_2), 26.7 (CH_3), 17.2 (CH_3);

HRMS (ESI) *m / z* calcd for $C_{12}H_{22}NaO_4$ $(M + Na)^+$ 253.1410, found 253.1401;

HRMS (ESI) *m / z* calcd for $C_{12}H_{21}O_3$ $[(M + H)^+ – H_2O]$ 213.1485, found 213.1476.

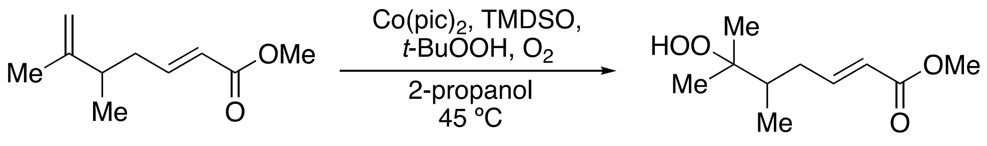

8j **9j**

Unsaturated Hydroperoxide 9j. Unsaturated hydroperoxide **9j** was synthesized according to the general procedure for the synthesis of unsaturated hydroperoxides. Diene **8j** (0.170 g, 1.01 mmol) in 2-propanol (17.0 mL) was mixed with Co(pic)$_2$ (0.0150 g, 0.0505 mmol), TMDSO (0.298 g, 2.22 mmol), and *t*-BuOOH (1.0 M in CH_2Cl_2, 0.200 mL, 0.20 mmol) under an atmosphere of oxygen for 7 h. Purification by flash column chromatography (15:85 EtOAc:hexanes) afforded unsaturated hydroperoxide X as a viscous, clear oil (0.148 g, 72%):

^{1}H NMR (400 MHz, C_6D_6) δ 7.05 (ddd, *J* = 15.5, 9.0, 6.2 Hz, 1H), 6.94 (br s, 1H), 5.87 (ddd, *J* = 15.5, 1.8, 1.0 Hz, 1H), 3.43 (s, 3H), 2.37–2.30 (m, 1H), 1.84 (dtd, *J* = 10.4, 7.0, 3.4 Hz, 1H), 1.54 (dddd, *J* = 14.1, 10.4, 9.0, 1.3 Hz, 1H), 1.00 (s, 3H), 0.90 (s, 3H), 0.65 (d, *J* = 7.0 Hz, 3H);

$^{13}C\{^1H\}$ NMR (100 MHz, C_6D_6) δ 166.8 (C), 149.5 (CH), 122.3 (CH), 84.2 (C), 51.0 (CH_3), 38.7 (CH), 35.0 (CH_2), 22.3 (CH_3), 20.3 (CH_3), 14.6 (CH_3);

HRMS (ESI) *m / z* calcd for $C_{10}H_{18}NaO_4$ $(M + Na)^+$ 225.1097, found 225.1090.

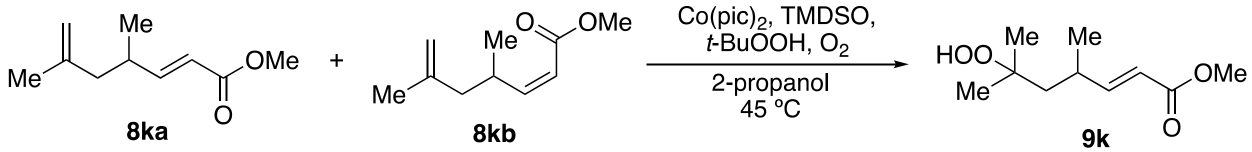

8ka **8kb** **9k**

Unsaturated Hydroperoxide 9k. Unsaturated hydroperoxide **9k** was prepared according to the general procedure for the synthesis of unsaturated hydroperoxides. Dienes **8ka** and **8kb** (0.300 g, 1.78 mmol) as a mixture of *E*:*Z* isomers in a 97:3 ratio, in 2-propanol (30.0 mL) was mixed with Co(pic)$_2$ (0.0810 g, 0.267 mmol), TMDSO (0.527 g, 3.92 mmol), and *t*-BuOOH (1.0 M, 0.360 mL, 0.36 mmol) under an atmosphere of oxygen for 6 h. Purification by flash column chromatography (10:90 EtOAc:hexanes) afforded the product as a colorless viscous oil (0.0710 g, 20%):

^{1}H NMR (400 MHz, C_6D_6) δ 7.07 (dd, J = 15.7, 8.4 Hz, 1H), 6.88 (br s, 1H), 5.83 (dd, J = 15.7, 1.0 Hz, 1H), 3.42 (s, 3H), 2.33–2.23 (m, 1H), 1.45 (dd, J = 14.6, 8.0 Hz, 1H), 1.33 (dd, J = 14.6, 4.7 Hz, 1H), 1.01 (s, 3H), 0.99 (s, 3H), 0.83 (d, J = 6.8 Hz, 3H);
$^{13}C\{^1H\}$ NMR (100 MHz, C_6D_6) δ 167.1 (C), 155.9 (CH), 119.2 (CH), 81.8 (C), 51.0 (CH_3), 44.4 (CH_2), 32.8 (CH), 24.7 (CH_3), 24.6 (CH_3), 21.5 (CH_3);
HRMS (ESI) *m* / *z* calcd for $(M + Na)^+$ $C_{10}H_{18}NaO_4$ 225.1097, found 225.1090;
HRMS (ESI) *m* / *z* calcd for $(M + NH_4)^+$ $C_{10}H_{22}NO_4$ 220.1543, found 220.1535.

IV. Synthesis of 1,2-Dioxanes

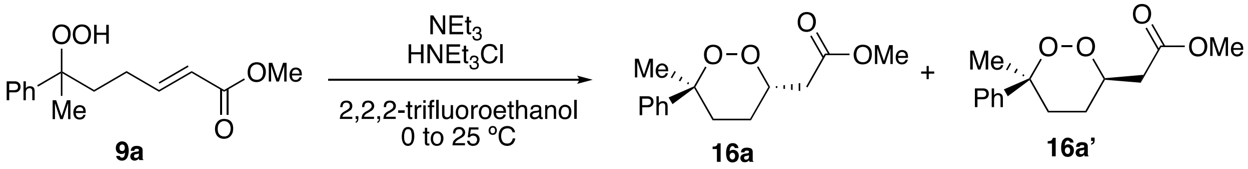

1,2-Dioxane 16a & 16a'. 1,2-Dioxane **16a** and **16a'** was prepared according to the general procedure for the synthesis of 1,2-dioxanes. Triethylammonium hydrochloride (0.457 g, 3.32 mmol) in TFE (11.1 mL) was mixed with unsaturated hydroperoxide **9a** (0.415 g, 1.66 mmol) and NEt_3 (0.0700 mL, 0.500 mmol). Purification by flash column chromatography (15:85 EtOAc:hexanes) afforded 1,2-dioxane **16a** and **16a'** as a clear oil as a mixture of diastereomers in a 53:47 ratio (0.322 g, 78%). Characterization was performed on a mixture of diastereomers (53:47):
^{1}H NMR (400 MHz, $CDCl_3$) δ 7.45–7.40 (m, 3.66H), 7.38–7.33 (m, 3.68H), 7.29–7.24 (m, 2.69H), 4.62 (dddd, J = 11.0, 7.9, 6.0, 2.5 Hz, 1.00H), 4.53 (ddt, J = 7.8, 5.8, 3.9 Hz, 0.82H), 3.72 (s, 2.37H), 3.65 (s, 2.91H), 2.83 (dd, J = 15.6, 7.6 Hz, 0.83H), 2.61–2.50 (m, 1.89H), 2.40–2.35 (dd, J = 15.9, 7.4 Hz, 1.00H), 2.26–2.15 (m, 1.92H), 2.11–1.89 (m, 2.86H), 1.81–1.71 (m, 1.91H), 1.56 (s, 2.81H), 1.50–1.40 (m, 1.21H), 1.36 (s, 3.03H);
Major diastereomer **16a**:
$^{13}C\{^1H\}$ NMR (100 MHz, $CDCl_3$) δ 170.7 (C), 144.7 (C), 128.4 (CH), 126.9 (CH), 125.0 (CH), 82.5 (C), 77.6 (CH, overlapping as determined by HSQC), 52.0 (CH_3), 38.2 (CH_2), 31.8 (CH_2), 26.0 (CH_3), 25.4 (CH_2);
Minor diasteromer **16a'**:
$^{13}C\{^1H\}$ NMR (100 MHz, $CDCl_3$) δ 171.0 (C), 143.8 (C), 128.5 (CH), 127.4 (CH), 125.8 (CH), 81.8 (C), 76.9 (CH, overlapping as determined by HSQC), 52.1 (CH_3), 37.9 (CH_2), 32.7 (CH_2), 30.3 (CH_3), 26.4 (CH_2);
HRMS (ESI) *m / z* calcd for $C_{14}H_{18}NaO_4$ $(M + Na)^+$ 273.1097, found 273.1084;
HRMS (ESI) *m / z* calcd for $C_{14}H_{18}KO_4$ $(M + K)^+$ 289.0838, found 289.0826;
IR (ATR) 2980, 2952, 1736, 1435, 1155, 922 cm^{-1}.

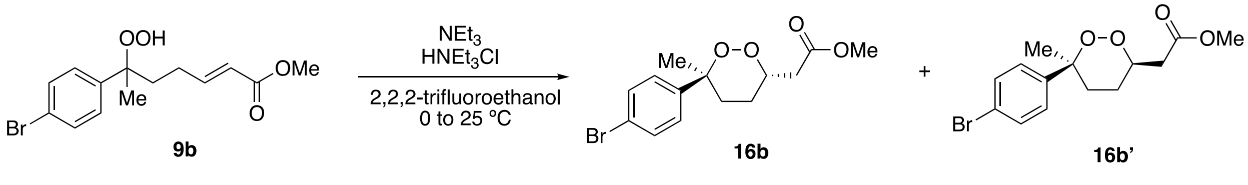

1,2-Dioxane 16b & 16b': 1,2-Dioxane **16b** and **16b'** was prepared according to the general procedure for the synthesis of 1,2-dioxanes. Triethylammonium hydrochloride (0.275 g, 2.00 mmol) in TFE (6.66 mL) was mixed with unsaturated hydroperoxide (0.329 g, 1.00 mmol) and NEt_3 (0.0420 mL, 0.300 mmol). Purification by flash column chromatography (15:85 EtOAc:hexanes) afforded 1,2-dioxane **16b** and **16b'** as a clear viscous oil (0.211 g, 64%) as a mixture of diastereomers in a 59:41 ratio. Characterization was performed on a mixture of diastereomers (59:41):

^1H NMR (400 MHz, $CDCl_3$) δ 7.49–7.46 (m, 3.28H), 7.32–7.27 (m, 3.37H), 4.61 (dddd, J = 10.7, 7.7, 5.7, 2.3 Hz, 1.00H), 4.51 (ddt, J = 7.5, 5.8, 4.1 Hz, 0.66H), 3.71 (s, 2.01H), 3.66 (s, 2.90H), 2.85 (dd, J = 15.7, 7.7 Hz, 0.65H), 2.61–2.56 (m, 0.90H), 2.46 (dt, J = 14.0, 3.7 Hz, 1.01H), 2.36 (dd, J = 15.9, 7.4 Hz, 0.99H), 2.24 (dd, J = 15.9, 5.9Hz, 1.02H), 2.17–1.88 (m, 3.26H) 1.78–1.69 (m, 1.71H), 1.51 (s, 2.10H), 1.45–1.38 (m, 0.98H), 1.33 (s, 2.89H);

Major diastereomer **16b**:

$^{13}C\{^1H\}$ NMR (100 MHz, $CDCl_3$) δ 170.6 (C), 143.8 (C), 131.6 (CH, overlapping peaks with minor diastereomer as determined by HSQC), 127.8 (CH), 120.9 (C), 82.3 (C), 76.8 (CH), 52.0 (CH_3), 38.1 (CH_2), 32.7 (CH_2), 26.2 (CH_3), 25.1 (CH_2);

Minor diastereomer **16b'**:

$^{13}C\{^1H\}$ NMR (100 MHz, $CDCl_3$) δ 171.0 (C), 142.9 (C), 131.6 (CH, overlapping peaks with major diastereomer as determined by HSQC), 127.0 (CH), 121.4 (C), 81.6 (C), 77.6 (CH), 52.1 (CH_3), 37.7 (CH_2), 31.5 (CH_2), 30.1 (CH_3), 26.3 (CH_2);

HRMS (ESI) m/z calcd for $C_{14}H_{17}BrNaO_4$ $(M + Na)^+$ 351.0202, found 351.0208;

HRMS (ESI) m/z calcd for $C_{14}H_{21}BrNO_4$ $(M + NH_4)^+$ 346.0648, found 346.0657;

IR (ATR) 2980, 2952, 1735, 1177, 1008, 821 cm^{-1}.

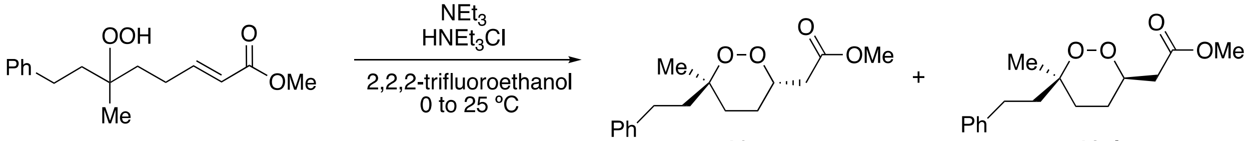

9c 16c 16c'

1,2-Dioxane 16c & 16c'. 1,2-Dioxane **16c** an **16c'** was prepared according to the general procedure for the synthesis of 1,2-dioxanes. Triethylammonium chloride (0.479 g, 3.48 mmol) in TFE (11.6 mL) was mixed with unsaturated hydroperoxide **9c** (0.485 g, 1.74 mmol) and NEt_3 (0.0750 mL, 0.522 mmol). Purification by flash column chromatography (15:85 EtOAc:hexanes) afforded 1,2-dioxane **16c** and **16c'** as a colorless, viscous oil (0.340 g, 70%) as a mixture of diastereomers in a 51:49 ratio. Characterization was performed was a mixture of diastereomers (51:49):

^1H NMR (400 MHz, $CDCl_3$) δ 7.31–7.26 (m, 2.02H), 7.23–7.16 (m, 2.95H), 4.52–4.47 (m, 1H), 3.71 (s, 1.57H), 3.70 (s, 1.29H), 2.74–2.55 (m, 3.08H), 2.49–2.40 (m, 1.06H), 2.26–2.18 (td, J = 13.1, 4.5 Hz, 0.46H), 1.85–1.67 (m, 5.76H), 1.38 (s, 1.69H), 1.26 (s, 0.21H), 1.21 (s, 1.42H);

Major diastereomer **16c**:

$^{13}C\{^1H\}$ NMR (100 MHz, $CDCl_3$) δ 170.9 (C), 142.3 (C), 128.6 (CH), 128.4 (CH), 126.0 (CH), 80.0 (C), 76.9 (overlapping with minor diastereomer as determined by HSQC, CH), 52.1 (CH_3),

41.9 (CH_2), 38.3 (CH_2), 32.2 (CH_2), 29.5 (CH_2), 25.6 (overlapping with minor diastereomer as determined by HSQC, CH_2), 20.9 (CH_3);

Minor diastereomer **16c'**:

$^{13}C\{^1H\}$ NMR (100 MHz, $CDCl_3$) δ 170.8 (C), 142.7 (C), 128.51 (CH), 128.49 (CH), 125.9 (CH), 79.8 (C), 76.9 (overlapping with minor diastereomer as determined by HSQC, CH), 52.0 (CH_3), 38.2 (CH_2), 37.2 (CH_2), 32.7 (CH_2), 29.9 (CH_2), 25.6 (overlapping with minor diastereomer as determined by HSQC, CH_2), 23.9 (CH_3);

HRMS (ESI) *m* / *z* calcd for $C_{16}H_{22}NaO_4$ $(M + Na)^+$ 301.1410, found 301.1404;

HRMS (ESI) *m* / *z* calcd for $C_{16}H_{22}KO_4$ $(M + K)^+$ 317.1150, found 317.1152;

IR (ATR) 3025, 2933, 1736, 1436, 1198, 1070 cm^{-1}.

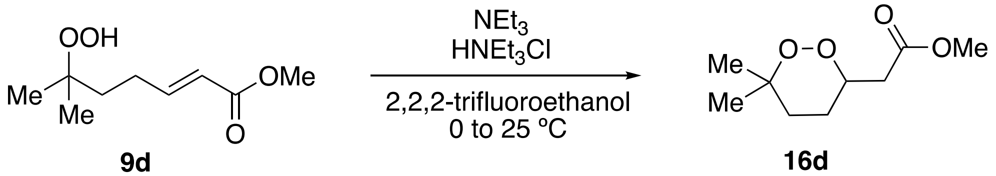

9d **16d**

1,2-Dioxane 16d. 1,2-Dioxane **16d** was prepared according to the general procedure for the synthesis of 1,2-dioxanes. Triethylammonium hydrochloride (0.278 g, 2.02 mmol) in TFE (6.70 mL) was mixed with unsaturated hydroperoxide **9d** (0.190 g, 1.01 mmol) and NEt_3 (0.0500 mL, 0.303 mmol). Purification by flash column chromatography (15:85 EtOAc:hexanes) afforded 1,2-dioxane **16d** as a colorless oil (0.135 g, 71%).

^1H NMR (400 MHz, $CDCl_3$) δ 4.45 (dddd, *J* = 10.6, 7.5, 5.9, 2.9 Hz, 1H), 3.69 (s, 3H), 2.58 (dd, *J* = 15.6, 7.5 Hz, 1H), 2.43 (dd, *J* = 15.5, 5.9 Hz, 1H), 1.78–1.65 (m, 4H), 1.33 (s, 3H), 1.18 (s, 3H);

$^{13}C\{^1H\}$ NMR (100 MHz, C_6D_6) δ 170.3 (C), 77.4 (C), 77.0 (CH), 51.2 (CH_3), 38.2 (CH_2), 33.7 (CH_2), 26.8 (CH_3), 26.0 (CH_2), 23.2 (CH_3);

HRMS (ESI) *m* / *z* calcd for $C_9H_{17}O_4$ $(M + H)^+$ 189.1121, found 189.1112;NH4

IR (ATR) 2980, 1737, 1437, 1197, 1154, 997 cm^{-1}.

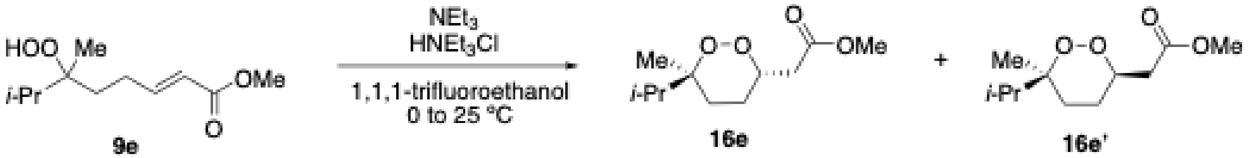

9e **16e** **16e'**

1,2-Dioxane 16e & 16e'. 1,2-Dioxane **16e** was prepared according to the general procedure for the synthesis of 1,2-dioxanes. Triethylammonium hydrochloride (0.363 g, 2.64 mmol) in TFE (8.80 mL) was mixed with unsaturated hydroperoxide **9e** (0.285 g, 1.32 mmol) and NEt_3 (0.0600 mL, 0.396 mmol). Purification by flash column chromatography (10:90 EtOAc:hexanes) afforded the 1,2-dioxane **16e** and **16e'** as a clear oil (0.154 g, 54%) as a mixture of diastereomers in a 64:36 ratio. Characterization was performed on a mixture of diastereomers (64:36). Spectroscopic data were recorded at 70 ºC to help resolve peaks:

^1H NMR (400 MHz, $(CD_3)_2SO$) δ 4.36–4.31 (m, 0.53H), 4.30–4.24 (m, 1.00H), 3.61 (s, 4.49H), 2.57–2.51 (m, 1.95H), 2.48–2.42 (m, 1.23H), 2.27 (sept, *J* = 6.8 Hz, 0.63H), 1.88 (dt, *J* = 13.4,

4.8 Hz, 0.63H), 1.76–1.56 (m, 6.39H), 1.51–1.44 (m, 0.69H), 1.13 (s, 2.86H), 0.93 (s, 1.79H), 0.85–0.82 (m, 9.56H);

Peaks attributed to major diastereomer **16e**:

$^{13}C\{^1H\}$ NMR (100 MHz, $(CD_3)_2SO$) δ 169.9 (C), 81.3 (C), 76.5 (CH), 51.0 (overlapping peaks with minor diastereomer as determined by HSQC, CH_3), 37.5 (CH_2), 34.8 (overlapping peaks with minor diastereomer as determined by HSQC, CH), 29.3 (CH_2), 24.5 (CH_2), 17.8 (CH_3), 16.3 (CH_3), 15.9 (CH_3);

Peaks attributed to minor diastereomer **16e'**:

$^{13}C\{^1H\}$ NMR (100 MHz, $(CD_3)_2SO$) δ 170.0 (C), 81.6 (C), 76.0 (CH), 51.0 (overlapping with major diastereomer as determined by HSQC, CH_3), 37.1 (CH_2), 34.8 (overlapping peaks with minor diastereomer as determined by HSQC, CH), 29.2 (CH_2), 24.0 (CH_2), 17.0 (CH_3), 16.0 (CH_3), 15.6 (CH_3);

HRMS (ESI) *m / z* calcd for $C_{11}H_{20}NaO_4$ $(M + Na)^+$ 239.1254, found 239.1245;

HRMS (ESI) *m / z* calcd for $C_{11}H_{24}NO_4$ $(M + NH_4)^+$ 234.1700, found 234.1694;

IR (ATR) 2986, 1738, 1649, 1291, 1149, 1013 cm^{-1}.

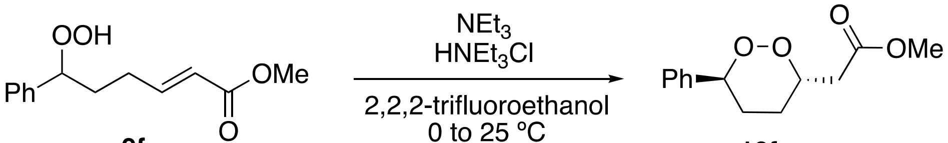

9f **16f**

1,2-Dioxane 16f. 1,2-Dioxane **16f** was prepared according to the general procedure for the synthesis of 1,2-dioxanes. Triethylammonium hydrochloride (0.281 g, 2.04 mmol) in TFE (6.80 mL) was mixed with unsaturated hydroperoxide **9f** (0.240 g, 1.02 mmol) and triethylamine (0.0450 mL, 0.306 mmol). Purification by flash column chromatography (15:85 EtOAc:hexanes) afforded 1,2-dioxane **16f** as white crystals (0.155 g, 65%) as a single diastereomer that were used to obtain a crystal structure to determine the relative stereochemistry:

^1H NMR (400 MHz, $CDCl_3$) δ 7.35–7.31 (m, 5H), 5.09 (dd, *J* = 7.8, 5.5 Hz, 1H), 4.69 (dddd, *J* = 11.1, 7.3, 5.9, 1.7 Hz, 1H), 3.72 (s, 3H), 2.56 (dd, *J* =15.7 Hz, 7.4 Hz, 1H), 2.45 (dd, *J* =15.7 Hz, 5.9 Hz, 1H), 2.09–2.01 (m, 3H), 1.80–1.69 (m, 1H);

$^{13}C\{^1H\}$ NMR (100 MHz, $CDCl_3$) δ 170.6 (C), 138.4 (C), 128.8 (CH), 128.7 (CH), 127.1 (CH), 83.5 (CH), 77.9 (CH), 52.1 (CH_3), 38.5 (CH_2), 30.3 (CH_2), 29.8 (CH_2);

HRMS (ESI) *m* / *z* calcd for $C_{13}H_{16}NaO_4$ $(M + Na)^+$ 259.0941, found 259.0938;

HRMS (ESI) *m / z* calcd for $C_{13}H_{20}NO_4$ $(M + NH_4)^+$ 254.1387, found 254.1388;

IR (ATR) 2953, 1735, 1438, 1280, 1160, 1066 cm^{-1};

mp = 52–53 °C.

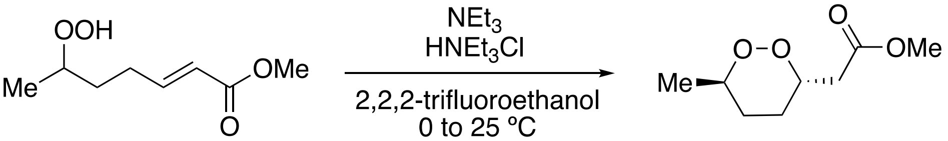

trans-**9g** **16g**

1,2-Dioxane 16g. 1,2-Dioxane **16g** was prepared according to the general procedure for the synthesis of 1,2-dioxanes. Triethylammonium hydrochloride (0.292 g, 2.12 mmol) in TFE (7.0

mL), was mixed with unsaturated hydroperoxide *trans*-**9g** (0.178 g, 1.02 mmol) and triethylamine (0.0500 mL, 0.306 mmol). Purification by flash column chromatography (10:90 EtOAc:hexanes) afforded 1,2-dioxane **16g** as a colorless oil (0.128 g, 72%) as a single diastereomer. The spectroscopic data were consistent with those previously reported:[39]

^{1}H NMR (400 MHz, C_6D_6) δ 4.54 (dddd, *J* = 10.2, 7.2, 5.6, 2.6 Hz, 1H), 4.00–3.93 (m, 1H), 3.27 (s, 3H), 2.30 (dd, *J* = 15.6, 7.5 Hz, 1H), 2.03 (dd, *J* = 15.6, 5.8 Hz, 1H), 1.44–1.35 (m, 1H), 1.24–1.12 (m, 3H), 0.84 (d, *J* = 6.4 Hz, 3H);

^{13}C{^{1}H} NMR (100 MHz, C_6D_6) δ 170.0 (C), 77.5 (CH), 77.1 (CH), 51.3 (CH_3), 38.5 (CH_2), 31.4 (CH_2), 29.6 (CH_2), 18.7 (CH_3);

HRMS (ESI) *m / z* calcd for $C_8H_{15}O_4$ $(M + H)^+$ 175.0965, found 175.0957;

IR (ATR) 2935, 1738, 1436, 1354, 1286, 1171 cm^{-1}.

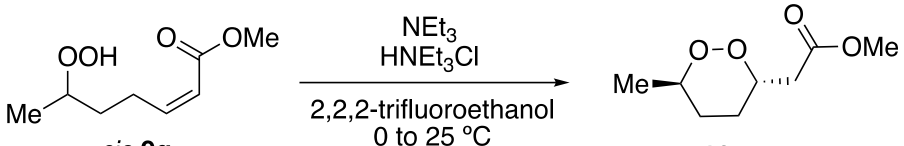

cis-**9g** **16g**

1,2-Dioxane 16g. 1,2-Dioxane **16g** was synthesized according to the general procedure for the synthesis of 1,2-dioxanes. Triethylammonium hydrochloride (0.292 g, 2.12 mmol) in TFE (7.00 mL) was mixed with unsaturated hydroperoxide *cis*-**9g** (0.184 g, 1.06 mmol) and NEt_3 (0.0500 mL, 0.318 mmol). Purification by flash column chromatography (10:90 EtOAc:hexanes) afforded the 1,2-dioxane **16g** as a clear oil (0.114 g, 62%) as a single diastereomer. The spectroscopic data were equivalent to 1,2-dioxane **16g** synthesized from *trans*-**9g**.

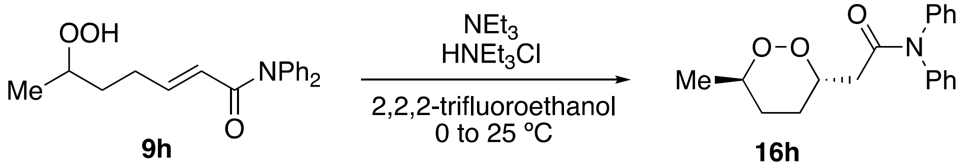

9h **16h**

1,2-Dioxanes 16h. 1,2-Dioxane **16h** was prepared according to the general procedure for the synthesis of 1,2-dioxanes. Triethylammonium hydrochloride (0.127 g, 0.926 mmol) in TFE (3.00 mL) was mixed with unsaturated hydroperoxide **9h** (0.144 g, 0.463 mmol) and NEt_3 (0.0200 mL, 0.139 mmol) for 72 h. Purification by flash column chromatography (30:70 EtOAc:hexanes) afforded 1,2-dioxane **9h** as white crystals as a single diastereomer that were used to obtain a crystal structure to determine the relative stereochemistry (0.0850 g, 59%). Spectroscopic data were recorded at 70 ºC to help resolve peaks:

^{1}H NMR (400 MHz, $(CD_3)_2SO$) δ 7.42–7.39 (m, 4H), 7.36–7.27 (m, 6H), 4.42 (dtd, *J* = 10.4, 6.5, 2.8 Hz, 1H), 4.08–4.00 (m, 1H), 2.29 (d, *J* = 6.4 Hz, 2H), 1.84–1.73 (m, 2H), 1.46–1.34 (m, 2H), 1.02 (d, *J* = 6.3 Hz, 3H);

^{13}C{^{1}H} NMR (100 MHz, $(CD_3)_2SO$) δ 168.2 (C), 142.5 (C), 128.9 (CH), 127.4 (CH), 126.6 (CH), 77.6 (CH), 76.4 (CH), 38.0 (CH_2), 30.5 (CH_2), 28.6 (CH_2), 18.0 (CH_3);

HRMS (ESI) *m / z* calcd for $C_{19}H_{22}NO_3$ $(M + H)^+$ 312.1594, found 312.1597;

HRMS (ESI) *m / z* calcd for $C_{19}H_{21}NNaO_3$ $(M + Na)^+$ 334.1414, found 334.1416;

IR (ATR) 2932, 1668, 1592, 1490, 1366, 1298 cm^{-1};
mp = 115–117 ºC.

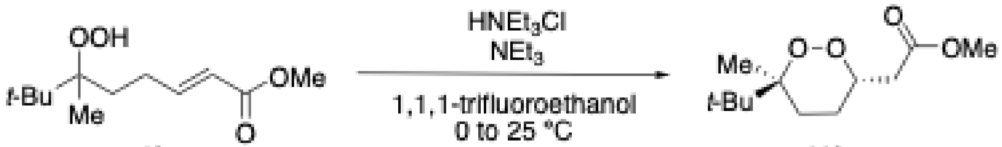

9i **16i**

1,2-Dioxane 16i. 1,2-Dioxane **16i** was prepared according to the general procedure for the synthesis of 1,2-dioxanes. Triethylammonium hydrochloride (0.195 g, 1.42 mmol) in TFE (4.30 mL) was mixed with unsaturated hydroperoxide **9i** (0.149 g, 0.647 mmol) and NEt_3 (0.0300 mL, 0.194 mmol). Purification by flash column chromatography (5:95 to 10:90 EtOAc:hexanes) afforded 1,2-dioxane **16i** as a colorless oil (0.0830 g, 56%) and as single diastereomer. The relative stereochemistry was determined by nOe analysis:
^{1}H NMR (600 MHz, C_6D_6) δ 4.41 (dddd, *J* = 10.8, 7.6, 5.7, 3.0 Hz, 1H), 3.28 (s, 3H), 2.33 (dd, 15.4, 7.6 Hz, 1H), 2.07 (dd, 15.4, 5.7 Hz, 2H), 1.65 (dt, *J* = 13.0, 5.7 Hz, 1H), 1.40 (tdd, *J* = 13.0, 11.3, 4.7 Hz, 1H) 1.32 (dtd, *J* = 10.7, 5.4, 2.6 Hz, 1H), 1.21 (s, 3H), 1.04 (ddd, *J* = 13.0, 4.7, 2.6 Hz, 1H), 0.87 (s, 9H);
^{13}C{^{1}H} NMR (100 MHz, C_6D_6) δ 170.2 (C), 83.1 (C), 77.2 (CH), 51.2 (CH_3), 38.7 (CH_2), 37.3 (C), 26.9 (CH_2), 26.4 (CH_2), 25.1 (CH_3), 16.6 (CH_3);
HRMS (ESI) *m* / *z* calcd for $C_{12}H_{22}NaO_4$ $(M + Na)^+$ 253.1410, found 253.1401;
IR (ATR) 2959, 1740, 1436, 1368, 1168, 990 cm^{-1}.

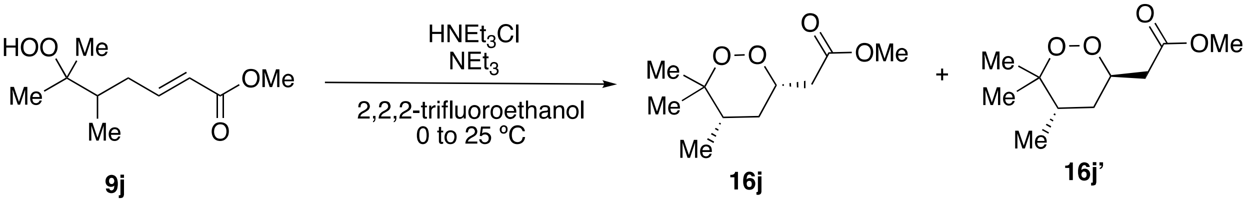

9j **16j** **16j’**

1,2-Dioxane 16j & 16j. 1,2-Dioxane **16j** and **16j’** was synthesized according to the general procedure for the synthesis of 1,2-dioxanes. Triethylammonium hydrochloride (0.187 g, 1.36 mmol) in TFE (4.50 mL) was mixed with unsaturated hydroperoxide **9j** (0.138 g, 0.682 mmol) and NEt_3 (0.0300 mL, 0.205 mmol). Purification by flash column chromatography (10:90 EtOAc:hexanes) afforded the 1,2-dioxane **16j** and **16j’** as a clear oil (0.105 g, 76%) as a mixture of diastereomers in a 94:6 ratio. The relative stereochemistry was determined by nOe analysis. Characterization was performed on a mixture of diastereomers (94:6):
^{1}H NMR (400 MHz, $(CD_3)_2SO$) δ 4.48–4.43 (m, 0.06H), 4.36 (dddd, *J* = 11.5, 8.5, 4.5, 2.4 Hz, 1H), 3.60 (s, 3.35H), 2.61 (dd, *J* = 15.9, 4.9 Hz, 0.04H), 2.54 (dd, *J* = 15.9, 4.5 Hz, 1.18H, overlapping with DMSO peak), 2.35 (dd, *J* = 15.9, 8.5 Hz, 1.00H), 1.75 (dqd, *J* = 12.5, 6.9, 4.8 Hz, 1.04H), 1.59 (ddd, *J* = 13.4, 4.5, 2.4 Hz, 1H), 1.37–1.27 (m, 1.05H), 1.15 (s, 0.26H), 1.14 (s, 2.99H) 1.10 (s, 0.21H), 1.05 (s, 3.04H), 0.87 (d, *J* = 6.9 Hz, 0.24H), 0.84 (d, *J* = 6.9 Hz, 3.07H);
Peaks attributed to major diastereomer **16j**:
^{13}C{^{1}H} NMR (100 MHz, $(CD_3)_2SO$) δ 170.3 (C), 81.3 (C), 77.8 (CH), 51.5 (CH_3), 37.7 (CH_2), 37.6 (CH), 33.7 (CH_2), 24.4 (CH_3), 17.8 (CH_3), 16.5 (CH_3);

Peaks attributed to minor diastereomer **16j'**:
$^{13}C\{^1H\}$ NMR (100 MHz, $(CD_3)_2SO$, characteristic peaks) δ 171.0 (C), 81.0 (C), 74.7 (CH), 51.4 (CH_3), 36.4 (CH_2), 32.4 (CH), 31.4 (CH_2), 24.7 (CH_3), 15.9 (CH_3);
HRMS (ESI) *m / z* calcd for $C_{10}H_{18}NaO_4$ $(M + Na)^+$ 225.1097, found 225.1088;
HRMS (ESI) *m / z* calcd for $C_{10}H_{22}NO_4$ $(M + NH_4)^+$ 220.1543, found 220.1537;
HRMS (ESI) *m / z* calcd for $C_{10}H_{19}O_4$ $(M + H)^+$ 203.1278, found 203.1272;
IR (ATR) 2971, 1738, 1437, 1273, 1159, 1057 cm^{-1}.

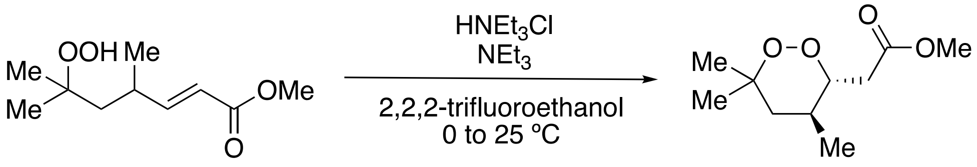

9k **16k**

1,2-Dioxane 16k. 1,2-Dioxane **16k** was prepared according to the general procedure for the synthesis of 1,2-dioxanes. Triethylammonium chloride (0.149 g, 1.08 mmol) in TFE (3.60 mL) was mixed with unsaturated hydroperoxide **9k** (0.109 g, 0.539 mmol) and NEt_3 (0.0300 mL, 0.162 mmol). Purification by flash column chromatography (10:90 EtOAc:hexanes) afforded 1,2-dioxane **16k** as a colorless oil (0.0330 g, 30%) as a single diastereomer. The relative stereochemistry was determined by *J* coupling constants; details are provided in Section III:
^1H NMR (400 MHz, C_6D_6) δ 4.17 (ddd, *J* = 10.1, 8.7, 3.6 Hz, 1H), 3.30 (s, 3H), 2.32 (dd, *J* = 15.5, 3.6 Hz, 1H), 2.18 (dd, *J* = 15.5, 8.7 Hz, 1H), 1.56–1.45 (m, 1H), 1.25 (s, 3H), 1.17 (dd, *J* = 13.2, 4.5 Hz, 1H), 1.06–0.96 (m, 4H), 0.49 (d, *J* = 6.6 Hz, 3H);
$^{13}C\{^1H\}$ NMR (100 MHz, C_6D_6) δ 170.6 (C), 83.5 (CH), 78.5 (C), 51.3 (CH_3), 43.4 (CH_2), 36.2 (CH_2), 30.8 (CH), 27.5 (CH_3), 23.2 (CH_3), 17.0 (CH_3);
HRMS (ESI) *m / z* calcd for $C_{10}H_{18}NaO_4$ $(M + Na)^+$ 225.1097, found 225.1086;
HRMS (ESI) *m / z* calcd for $C_{10}H_{22}NO_4$ $(M + NH_4)^+$ 220.1543, found 220.1533;
HRMS (ESI) *m / z* calcd for $C_{10}H_{19}O_4$ $(M + H)^+$ 203.1278, found 203.1268;
IR (ATR) 2981, 1739, 1540, 1255, 1141, 1068 cm^{-1}.

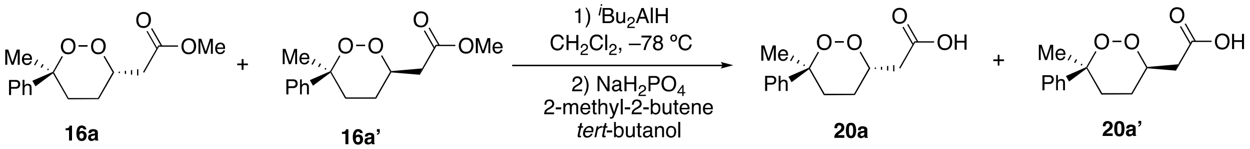

16a **16a'** **20a** **20a'**

Carboxylic Acid 20a & 20a'. To a cooled (–78 °C) solution of 1,2-dioxane **16a** and **16a'** (0.150 g, 0.600 mmol), as a mixture of diasteromers in a 53:47 ratio, in CH_2Cl_2 (3.00 mL) was added dropwise a solution of diisobutylaluminum hydride (0.550 mL, 1.2 M in PhMe, 0.66 mmol). After 2 h, MeOH (3 mL) and aqueous 1 M HCl (3 mL) were added and the mixture was allowed to stir at 25 °C for 2 h. The reaction mixture was extracted with CH_2Cl_2 (3 x 10 mL) and the combined organic layers were washed with brine (15 mL), dried over Na_2SO_4, filtered, and concentrated *in vacuo*. The unpurified aldehyde was used immediately in the next step without further purification.

To a stirring solution of unpurified aldehyde and 2-methyl-2-butene (0.320 mL, 3.00 mmol) in *tert*-butanol (6.00 mL) was added a solution of NaH_2PO_4 (0.280 g, 2.34 mmol) and

$NaClO_2$ (0.195 g, 2.16 mmol) in water (0.770 mL) dropwise at 25 ºC. The reaction mixture was allowed to stir for 16 h. EtOAc (10 mL) and aqueous 1 M HCl (5 mL) were added to the reaction mixture. The reaction mixture was extracted with EtOAc (3 x 10 mL). The combined organic layers were washed with saturated aqueous Na_2SO_3 (25 mL) and brine (25 mL), dried over $MgSO_4$, filtered, and concentrated *in vacuo*. Purification by flash column chromatography (50:50 to 60:40 EtOAc:hexanes) afforded carboxylic acid **20a** and **20a'** as a white solid (0.099 g, 70%) as a mixture of diastereomers in a 55:45 ratio. Characterization was performed on a mixture of diastereomers (55:45):

^{1}H NMR (400 MHz, C_6D_6) δ 10.3 (br s, 1.17H), 7.38–7.35 (m, 2.14H), 7.32–7.30 (m, 1.60H), 7.18–7.11 (m, 4.50H), 7.05–7.03 (m, 1.86H), 4.49 (dddd, *J* = 10.4, 7.8, 5.4, 2.6 Hz, 1.00H), 4.33 (tdd, *J* = 7.7, 5.4, 4.1 Hz, 0.75H), 2.60 (dd, *J* = 16.0, 7.8 Hz, 0.72H), 2.16 (dd, *J* = 16.0, 5.4 Hz, 0.78H), 2.02 (td, *J* = 13.9, 3.7 Hz, 2.02H), 1.76–1.70 (m, 1.82H), 1.58–1.50 (m, 1.93H), 1.40–1.34 (m, 0.95H), 1.32 (s, 2.41H), 1.24–1.02 (m, 6.56H);

Peaks attributed to major diasteromer **20a**:

^{13}C{^{1}H} NMR (100 MHz, C_6D_6) δ 177.0 (C), 145.4 (C), 128.6 (CH), 127.0 (CH), 125.4 (CH), 82.3 (C), 76.4 (CH), 38.0 (CH_2), 31.8 (CH_2), 30.2 (CH_3), 25.6 (CH_2);

Peaks attributed to minor diasteromer **20'**:

^{13}C{^{1}H} NMR (100 MHz, C_6D_6) δ 177.5 (C), 144.3 (C), 128.5 (CH), 127.3 (CH), 126.1 (CH), 81.6 (C), 77.3 (CH), 37.8 (CH_2), 32.7 (CH_2), 26.3 (CH_2), 26.1 (CH_3);

HRMS (ESI) *m / z* calcd for $C_{13}H_{16}NaO_4$ $(M + Na)^+$ 259.0941, found 259.0944;

IR (ATR) 2981 (br), 1710, 1446, 1299, 1271, 1161 cm^{-1};

mp = 83–85 ºC.

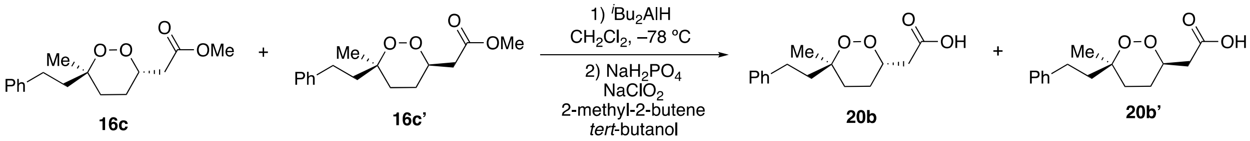

Carboxylic Acid 20b & 20b'. To a cooled (–78 ºC) solution of 1,2-dioxane **16c** and **16c'** (0.0500 g, 0.180 mmol) in CH_2Cl_2 (0.900 mL) as a mixture of diastereomers (51:49) was added dropwise a solution of diisobutylaluminum hydride (0.170 mL, 1.2 M in PhMe, 0.20 mmol) while the reaction mixture was stirring. After 2 h, MeOH (2 mL) and aqueous 1 M HCl (2 mL) were added and the reaction mixture was allowed to stir at 25 ºC for 2 h. The reaction mixture was extracted with CH_2Cl_2 (3 x 5 mL) and the combined organic layers were washed with brine (10 mL), dried over Na_2SO_4, filtered, and concentrated *in vacuo*. The unpurified aldehyde was used immediately in the next step without further purification.

To a stirring solution of unpurified aldehyde and 2-methyl-2-butene (0.100 mL, 0.900 mmol) in *tert*-butanol (1.80 mL) were added a mixture of NaH_2PO_4 (0.0840 g, 0.702 mmol) and $NaClO_2$ (0.0750 g, 0.648 mmol) in water (0.240 mL) dropwise at 25 ºC. The reaction mixture was allowed to stir for 16 h. EtOAc (5 mL) and aqueous 1 M HCl (3 mL) were added to the reaction mixture. The reaction mixture was extracted with EtOAc (3 x 10 mL). The combined organic layers were washed with saturated aqueous Na_2SO_3 (15 mL) and brine (15 mL), dried over $MgSO_4$, filtered, and concentrated *in vacuo*. Purification by flash column chromatography

(50:50 to 60:40 EtOAc:hexanes) afforded carboxylic acid **20b** and **20b'** as a clear viscous oil (0.0250 g, 52%) as a mixture of diastereomers in a 51:49 ratio. Characterization was performed on a mixture of diastereomers (51:49):
^{1}H NMR (400 MHz, C_6D_6) δ 7.21–7.19 (m, 1.13 H), 7.14–7.04 (m, 3.81H), 4.45–4.37 (m, 1.00H), 2.76 (td, *J* = 12.9, 4.4 Hz, 0.53H), 2.61 (td, *J* = 12.9, 4.8 Hz, 0.51H), 2.54–2.31 (m, 2.05H), 2.20–2.00 (m, 1.59H), 1.81–1.74 (m, 0.48H), 1.60–1.50 (m, 1.11H), 1.32–1.24 (m, 3.99H), 1.26–1.20 (m, 1H), 1.16 (s, 1.86H), 0.98 (s, 1.57H);
Peaks attributed to major diastereomer **20b**:
^{13}C{^{1}H} NMR (100 MHz, C_6D_6) δ 176.86 (C), 143.0 (C), 128.74 (CH), 128.73 (CH), 126.08 (CH), 79.3 (C), 76.5 (CH), 38.0 (CH_2), 37.5 (CH_2), 32.0 (CH_2), 30.1 (CH_2), 25.4 (CH_2, overlapping peak with minor diastereomer as determined by HSQC), 23.7 (CH_3);
Peaks attributed to minor diastereomer **20b'**:
^{13}C{^{1}H} NMR (100 MHz, C_6D_6) δ 176.93 (C), 142.6 (C), 128.77 (CH), 128.69 (CH), 126.14 (CH), 79.4 (C), 76.7 (CH), 42.0 (CH_2), 38.2 (CH_2), 32.7 (CH_2), 29.8 (CH_2), 25.4 (CH_2, overlapping peak with major diastereomer as determined by HSQC), 21.0 (CH_3);
HRMS (ESI) *m* / *z* calcd for $C_{15}H_{20}NaO_4$ $(M + Na)^+$ 287.1254, found 287.1243;
IR (ATR) 2938 (br), 1712, 1452, 1298, 1156, 1099 cm^{-1}.

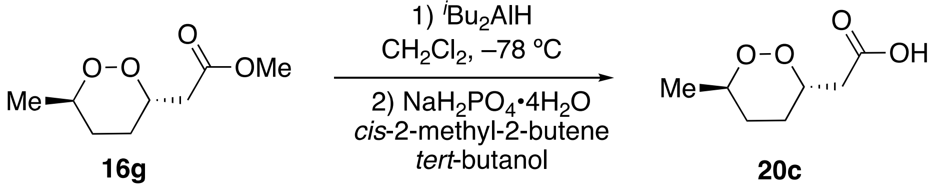

Carboxylic Acid 20c. To a cooled (–78 ºC) solution of 1,2-dioxane **16g** (0.160 g, 0.918 mmol) in CH_2Cl_2 (4.60 mL) was added dropwise a solution of diisobutylaluminum hydride (1.2 M in PhMe, 0.840 mL, 1.0 mmol) while the reaction mixture was stirring. After 2 h, MeOH (5 mL) and aqueous 1 M HCl (5 mL) were added and the reaction mixture was allowed to stir at 25 ºC for 2 h. The reaction mixture was extracted with CH_2Cl_2 (3 x 10 mL) and the combined organic layers were washed with brine (10 mL), dried over Na_2SO_4, filtered, and concentrated *in vacuo*. The unpurified aldehyde was used immediately in the next step without further purification.

To a stirring solution of unpurified aldehyde and 2-methyl-2-butene (0.490 mL, 4.59 mmol) in *tert*-butanol (9.20 mL) were added a solution of NaH_2PO_4 (0.427 g, 3.58 mmol) and $NaClO_2$ (0.298 g, 3.30 mmol) in water (1.30 mL) dropwise at 25 ºC. The reaction mixture was allowed to stir for 16 h. EtOAc (5 mL) and aqueous 1 M HCl (3 mL) was added to the reaction mixture. The reaction mixture was extracted with EtOAc (3 X 10 mL). The combined organic layers were washed with saturated aqueous Na_2SO_3 (15 mL) and brine (15 mL), dried over $MgSO_4$, filtered, and concentrated *in vacuo*. Purification by flash column chromatography (50:50 to 60:40 EtOAc:hexanes) afforded carboxylic acid **20c** as a white solid (0.0800 g, 54%):
^{1}H NMR (400 MHz, C_6D_6) δ 4.45 (dddd, *J* = 10.4, 7.6, 5.7, 2.4, 1H), 3.98–3.91 (m, 1H), 2.24 (dd, *J* = 15.6, 7.6 Hz, 1H), 1.96 (dd, *J* = 15.6, 5.7 Hz, 1H), 1.33–1.31 (m, 1H), 1.22–1.09 (m, 3H), 0.83 (d, *J* = 6.4 Hz, 3H);

$^{13}C\{^1H\}$ NMR (100 MHz, C_6D_6) δ 177.0 (C), 77.1 (CH), 77.0 (CH), 38.4 (CH_2), 31.2 (CH_2), 29.4 (CH_2), 18.7 (CH_3);
HRMS (ESI) *m* / *z* calcd for $C_7H_{12}NaO_4$ $(M + Na)^+$ 183.0628, found 183.0632;
IR (ATR) 2974 (br), 1171, 1432, 1370, 1290, 1179 cm^{-1};
mp = 80–82 ºC.

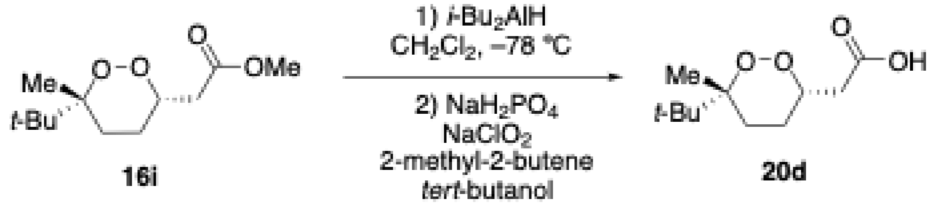

Carboxylic Acid 20d. To a cooled (–78 ºC) solution of 1,2-dioxane **16i** (0.0550 g, 0.239 mmol) in CH_2Cl_2 (1.20 mL) was added dropwise a solution of diisobutylaluminum hydride (1.2 M in PhMe, 0.220 mL, 0.26 mmol) while the reaction mixture was stirring. After 2 h, MeOH (2 mL) and aqueous 1 M HCl (2 mL) were added and the reaction mixture was allowed to stir at 25 ºC for 2 h. The reaction mixture was extracted with CH_2Cl_2 (3 x 10 mL) and the combined organic layers were washed with brine (10 mL), dried over Na_2SO_4, filtered, and concentrated *in vacuo*. The unpurified aldehyde was used immediately in the next step without further purification.

To a stirring solution of unpurified aldehyde and 2-methyl-2-butene (0.120 mL, 1.12 mmol) in *tert*-butanol (2.40 mL) was added a mixture of NaH_2PO_4 (0.111 g, 0.932 mmol) and $NaClO_2$ (0.0980 g, 0.860 mmol) in water (0.310 mL) dropwise at 25 ºC. The reaction mixture was allowed to stir for 16 h. EtOAc (5 mL) and aqueous 1 M HCl (3 mL) was added to the reaction mixture. The reaction mixture was extracted with EtOAc (3 x 10 mL). The combined organic layers were washed with saturated aqueous Na_2SO_3 (15 mL) and brine (15 mL), dried over $MgSO_4$, filtered, and concentrated *in vacuo*. Purification by flash column chromatography (50:50 to 60:40 EtOAc:hexanes) afforded carboxylic acid **20d** as a white solid (0.0300 g, 58%):
^1H NMR (400 MHz, C_6D_6) δ 4.31 (dddd, *J* = 10.7, 7.8, 5.7, 2.8 Hz, 1H), 2.26 (dd, *J* = 15.8, 7.8 Hz, 1H), 1.99 (dd, *J* = 15.8, 5.7 Hz, 1H), 1.61 (td, *J* = 13.0, 5.9 Hz, 1H), 1.36–1.27 (m, 1H), 1.26–1.20 (m, 1H), 1.18 (s, 3H), 1.00 (ddd, *J* = 13.1, 4.4, 2.8 Hz, 1H), 0.87 (s, 9H);
$^{13}C\{^1H\}$ NMR (100 MHz, C_6D_6) δ 175.2 (C), 83.1 (C), 76.8 (CH), 38.4 (CH_2), 37.3 (C), 26.8 (CH_2), 26.2 (CH_2), 25.1 (CH_3), 16.5 (CH_3);
HRMS (ESI) *m* / *z* calcd for $C_{11}H_{24}NO_4$ $(M + NH_4)^+$ 234.1700, found 234.1696;
IR (ATR) 2959 (br), 1712, 1438, 1372, 1250, 1192 cm^{-1};
mp = 115–116 ºC.

V. Stereochemical Correlations and Proofs

A. Assignment of Relative Stereochemical Configurations by ^1H NMR Coupling Constants

a. ^1H NMR Coupling Constants of 1,2-Dioxanes

Peaks were assigned in the ^{1}H NMR spectra by ^{1}H NMR chemical shifts and ^{1}H NMR coupling constants. The stereochemical configurations of the products were determined using ^{1}H NMR coupling constants because the coupling constants between protons on six-membered rings are well documented.[40] Protons will either adopt an axial or equatorial orientation on the ring (or a mixture of these two conformers), and the coupling between adjacent protons (the coupling constant, J) will help to elucidate those orientations. The relative orientation between protons on the six-membered ring are represented by the coupling constant, and they are as follows: axial–axial, 8 to 13 Hz; axial–equatorial, 2 to 6 Hz; equatorial–equatorial, 2 to 9 Hz.[40]

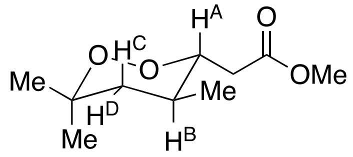

1,2-Dioxane 16k (1,2-*trans*, major diastereomer):
^{1}H NMR (400 MHz, C_6D_6) δ 4.17 (H^A: ddd, J = 10.1(ax^A–ax^B), 8.7, 3.6 Hz, 1H).

Note: The splitting pattern of H^A indicates that H^A is axial (ddd, J = 10.1, 8.7, 3.6 Hz), which places the larger CH_2COMe group and the methyl group in equatorial positions. The structure is 1,2-*trans* equatorial because the larger groups adopt an equatorial orientation.

B. Assignment of Relative Stereochemical Configurations by nOe analysis

General Procedure for DPFGSE–nOe experiments

All DPFGSE–nOe data were collected for samples prepared in degassed C_6D_6 with a mixing time of 0.50 seconds. All peaks in the ^{1}H NMR spectra were assigned using $^{1}H/^{1}H$ COSY, $^{1}H/^{13}C$ HSQC, $^{1}H/^{13}C$ HMBC, and ^{1}H NMR chemical shifts. Stereochemical assignments for 1,2-dioxanes **16i** and **16j** were determined using the nOe data obtained.

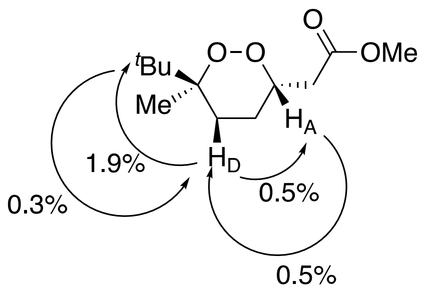

Relevant nOe data for 1,2-dioxane **16i** in C_6D_6

H_A irradiated: H_D (0.5%)

H_D irradiated: H_A (0.5%), *t*Bu (1.8%)

*t*Bu irradiated: H_D (0.3%)

Note: The nOe observed between H_A, H_D, and *t*Bu indicate that these protons are *syn*.

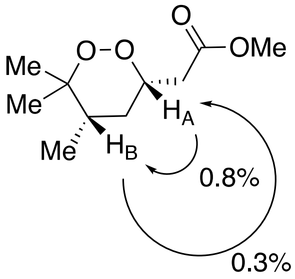

Relevant nOe data for 1,2-dioxane **16j** in C_6D_6

H_A irradiated: H_B (0.3%)

H_B irradiated: H_A (0.8%)

Note: The nOe observed between H_A and H_B indicate that these protons are *syn*.

VI. Crystallographic Data of 16f & 16h

Crystals were mounted on a MiTeGen MicroMount with Type B immersion oil (Cargille Labs). Single crystal X-ray intensity data were measured on a Bruker D8 SMART APEXII 'three-circle diffractometer' system equipped with a Incoatec 'microfocus sealed X-ray tube' (MoKα radiation, λ = 0.71073 Å), a multilayer optics monochromator and a PHOTON-II-C14 detector. Crystal temperature was controlled by an Oxford Cryosystems 700+ Cooler. Full datasets were collected with ω scans at $T = 100(2)$ K. The frames were integrated with the Bruker SAINT software package using a narrow-frame algorithm and the data were corrected for absorption effects using the Multi-Scan method with the SADABS software. The structures were solved by intrinsic phasing methods (SHELXT) and the structure models were completed and refined using the full-

matrix least-square methods on F^2 (SHELXL). All non-hydrogen atoms were refined with anisotropic displacement parameters, and hydrogen atoms on carbons were placed in idealized positions (C-H = 0.95-1.00 Å) and included using a riding model with U_{iso}(H) = 1.2 or 1.5 U_{eq}(non-H).

Crystals of **16f** were obtained by slow diffusion from the solution of EtOAc layered in n-hexane. Crystals of **16h** were obtained by slow evaporation in a solution of benzene. The crystal was kept at 100 K during data collection. Crystallographic data for **16f** and **16h** have been deposited with the Cambridge Crystallographic Data Centre (CCDC) under deposition numbers 2393611 and 2393612, respectively (the ellipsoid contour 50% probability levels).

Figure S1. Molecular structure of **16f**. The hydrogen atoms are omitted for clarity. The thermal ellipsoids are shown at 50% probability.

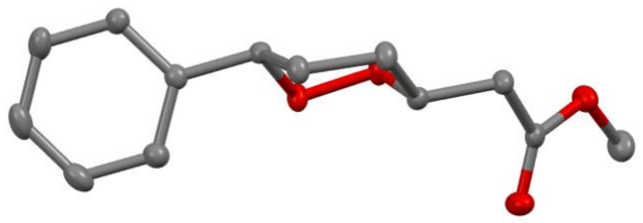

Figure S2. Molecular structure of **16h**. The hydrogen atoms are omitted for clarity. The thermal ellipsoids are shown at 50% probability.

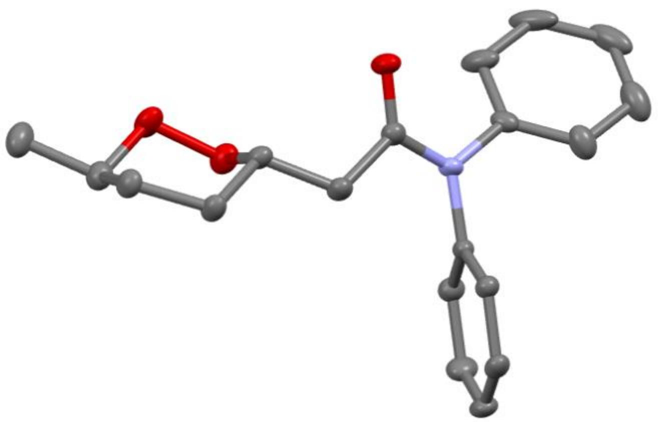

Table S1. Crystallographic data for **16f**

Identification Code	23kaw4s
Chemical Formula	$C_{13}H_{16}O_4$
Formula Weight	236.26
Temperature	100 K
Wavelength	0.71073 Å
Crystal System	Monoclinic
Space Group	P 1 21/n 1
a	10.1222(4)
b	18.4649(7)
c	13.6988(6)
α (°)	90
β (°)	109.950(2)
γ (°)	90
Volume	2406.73(17)
Z	8
Calculated density (g/cm^{-3})	1.304
Absorption Coefficient	0.096 mm^{-1}
F (000)	1008.0
Theta range for data collection (°)	0.995 to 28.550
Final R indices	R1 = 0.0429, wR2 = 0.1067

Table S2. Crystallographic data for **16h**

Identification Code	24kaw5s
Chemical Formula	$C_{19}H_{21}O_3$
Formula Weight	311.37
Temperature	100 K
Wavelength	0.71073 Å
Crystal System	Monoclinic
Space Group	P 1 21/c 1
a	9.6040(6)
b	19.0511(12)
c	9.4356(6)
α (°)	90
β (°)	108.122(3)
γ (°)	90
Volume	1640.77(18)
Z	4
Calculated density (g/cm^{-3})	1.260
Absorption Coefficient	0.085 mm^{-1}
F (000)	664.0
Theta range for data collection (°)	0.991 to 28.650
Final R indices	R1 = 0.0723, wR2 = 0.1822

VII. References

1. Gottlieb, H. E.; Kotlyar, V.; Nudelman, A. *J. Org. Chem.* **1997**, *62*, 7512–7515.
2. Otte, D. A.; Borchmann, D. E.; Lin, C.; Weck, M.; Woerpel, K. A. *Org. Lett*. **2014**, *16*, 1566–1569.
3. Peralta-Neel, Z; Woerpel, K. A. *Org. Lett.* **2021**, 23, 5002–5006.
4. Nakatsuji, H.; Sawamura, Y.; Sakakura, A.; Ishihara, K. *Angew. Chem. Int. Ed.* **2014**, *53*, 6974–6977.
5. Theodorou, A.; Kokotos, C. G. *Green Chem*. **2017**, *19*, 670–674.
6. Wolfe, J. P; Hardin, A. H; Hay, M. B. *J. Org. Chem.* **2005**, *70*, 3099–3107.
7. Koh, M.; Kim, H.; Shin, N.; Kim, H. S.; Yoo, D.; Kim, Y. G. *Bull. Korean. Chem. Soc.* **2012**, *33*, 1873–1878.
8. Klake, R. K.; Gargaro, S. L.; Gentry, S. L.; Elele, S. O.; Sieber, J. D. *J. Am. Chem. Soc.* **2021**, *143*, 13489–13494.
9. Kiyokawa, K.; Noguchi, I.; Nagata, T.; Minakata, S. *Org. Lett*. **2023**, *25*, 2537–2542.
10. Xiao, G.; Xie, C.; Guo, Q.; Zi, G.; Hou, G.; Huang, Y. *Org. Lett.* **2022**, *24*, 2722–2727.
11. Kallemeyn, J. M.; Mulhern, M. M.; Ku, Y. *Synlett* **2011**, *4*, 535–538.
12. Harada, S.; Koyama, R.; Masuda, R.; Arai, S. *Eur. J. Org. Chem.* **2023**, *26*, e202300747.
13. Li, S.; Zhang, J.; Li, H.; Feng, L.; Jiao, Peng, J. *J. Org. Chem.* **2019**, *84*, 9460–9473.
14. Karyakarte, S. D.; Um, C.; Berhane, I. A.; Chemler, S. R. *Angew. Chem. Int. Ed.* **2018**, *57*, 12921–12924.
15. Hansen, A. L.; Ebran. J.; Gøgsig, T. M.; Skrydstrup, T. *J. Org. Chem.* **2007**, *72*, 6464–6472.
16. Banwell, M. G.; Bissett, B. D.; Bui, C. T.; Pham, H. T. T.; Simpson, G. W. *Aust. J. Chem.* **1998**, *51*, 9–18.
17. Wei, H.; Li, Y.; Xiao, K.; Cheng, B.; Wang, H.; Hu, L.; Zhai, H. *Org. Lett.* **2015**, *17*, 5974–5977.
18. Kowalczyk, R.; Wierzba, A, J.; Boratynski, P. J.; Bakowicz, J. *Tetrahedron,* **2014**, *70*, 5834–5842.
19. Nchinda, A. T.; Chibale, K.; Redelinghuys, P.; Sturrock, E. D. *Bioorg. Med. Chem. Lett.* **2006**, *16*, 4612–4615.
20. Guijarro, D.; Pablo, O.; Yus, M. *J. Org. Chem.* **2013**, *78*, 3647–3654.
21. Airiau, E.; Chemin, C.; Girard, N.; Lonzi, G.; Mann, A.; Petricci, E.; Salvadori, J.; Taddei, M. *Synthesis* **2010**, *17*, 2901–2914.
22. Schuch, D.; Fries, P.; Dönges, M.; Pérez, B. M.; Hartung, J. *J. Am. Chem. Soc.* **2009**, *131*, 12918–12920.
23. Garnes-Portolés, F.; Miguélez, R.; Grayson, M. N.; Barrio, P. *Eur. J. Org. Chem.* **2021**, 3492–3495.
24. Harcken, C.; Brückner, R.; Rank, E. *Chem. Eur. J.* **1998**, *4*, 2342–2352.
25. Manzano, R.; Romaniega, A.; Prieto, L.; Diaz, E.; Reyes, E.; Uris, U.; Carrillo, L.; Vicario, J. L. *Org. Lett.* **2020**, *22*, 4721–4725.

26. Ohkuma, T.; Sandoval, C. A.; Srinivasan, R.; Lin, Q.; Wei, Y.; Muñiz, K.; Noyori, R. *J. Am. Chem. Soc.* **2005**, *127*, 8288–8289.
27. Jain, A.; Kumari, A.; Saha, S. K.; Shukla, K.; Chauhan, A.; Metre, R. K.; Rana, N. K. *J. Org. Chem.* **2023**, *88*, 11346–11351.
28. Sato, T.; Okazaki, H.; Otera, J.; Nozaki, H. *J. Am. Chem. Soc.* **1998**, *110*, 5209–5211.
29. Nawrat, C. C.; Lewis, W.; Moody, C. J. *J. Org. Chem.* **2011**, *76*, 7872–7881.
30. Nakashima, K.; Inoue, K.; Sono, M.; Tori, M. *J. Org. Chem.* **2002**, *67*, 6034–6040.
31. Geraghty, N. W. A.; McArdle, P.; Mullen, L. M. A. *Tetrahedron* **2011**, *67*, 3546–3552.
32. Wollowitz, S.; Halpern, J. *J. Am. Chem. Soc.* **1988**, *110*, 3112–3120.
33. Rossini, A. F. C.; Dias, L. C. *J. Braz. Chem. Soc.* **2019**, *30*, 1567–1578.
34. Zukerman, D. S.; Woerpel, K. A. *Tetrahedron* **2019**, *75*, 4118–4129.
35. Nandurkar, N. S.; Patil, D. S.; Bhanage, B. M. *Inorg. Chem. Commun.* **2008**, 11, 733– 736.
36. Wu, D.; Forsyth, C, J. *Org. Lett.* **2013**, *15*, 1178–1181.
37. (a) Fries, P.; Halter, D.; Kleinschek, A.; Hartung, J. *J. Am. Chem. Soc.* **2011**, *133*, 3906–3912. (b) Pérez, B. M.; Hartung, J. *Tetrahedron Lett.* **2009**, *50*, 960–962.
38. Novitski, G.; Silagi-Dumitresku, I.; Paraskivesku, A.; Gulya, A. *Russ. J. Coord. Chem.* **2001**, *27*, 722– 725.
39. Guerra, F. M.; Zubía, E.; Ortega, M. J.; Moreno-Dorado, F. J.; Massanet, G. M. *Tetrahedron* **2010**, *66*, 157–163.
40. Haasnoot, C. A. G. *J. Am. Chem. Soc.* **1993**, *115*, 1460–1468

VIII. Spectra

400 MHz, $CDCl_3$

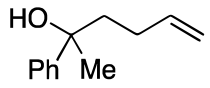

S2

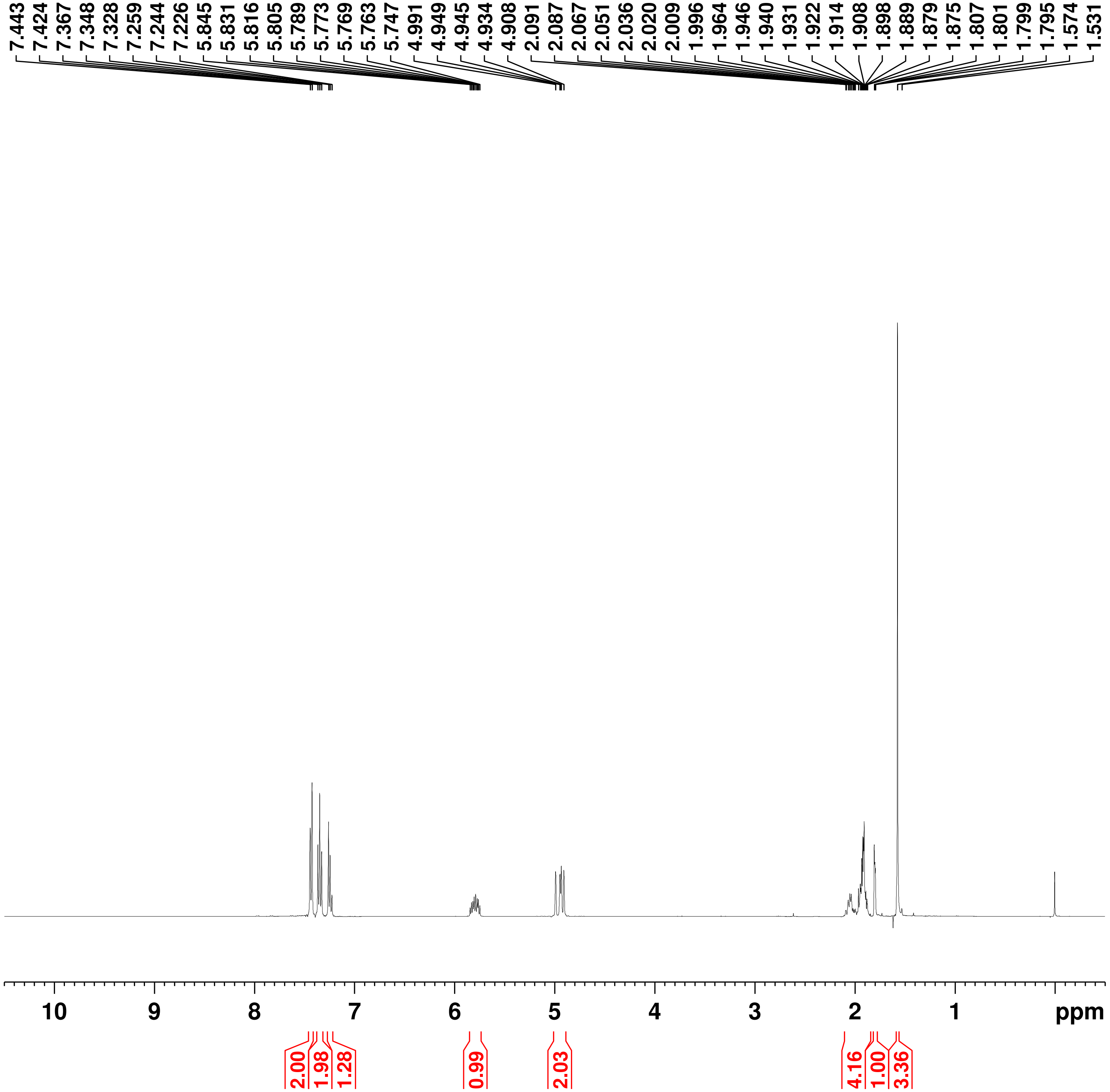

^{1}H spectrum of compound **S2**

100 MHz, $CDCl_3$

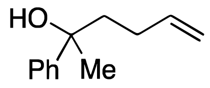

S2

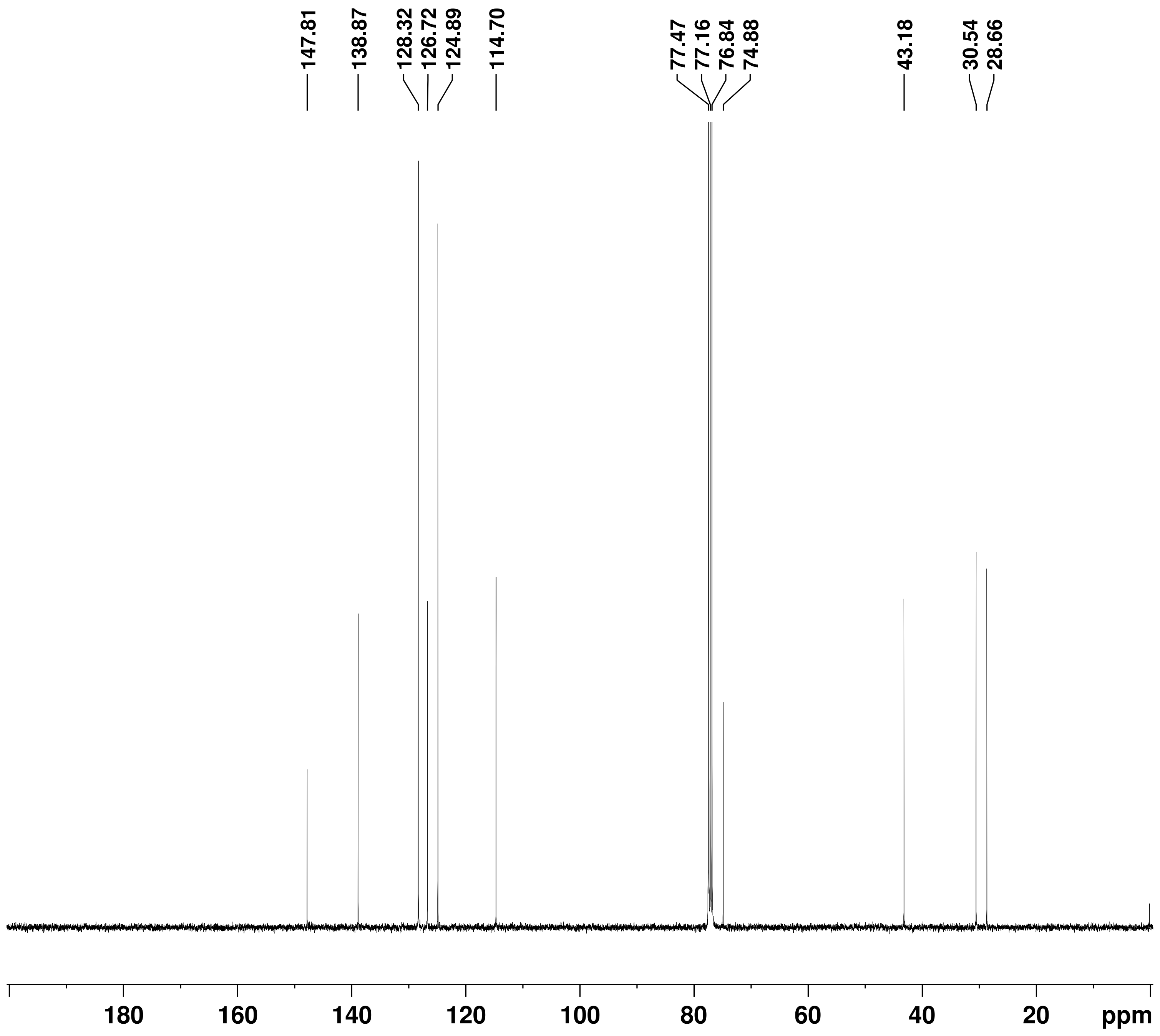

^{13}C spectrum of compound **S2**

100 MHz, $CDCl_3$

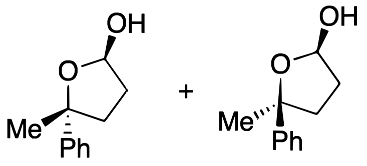

S3a S3b

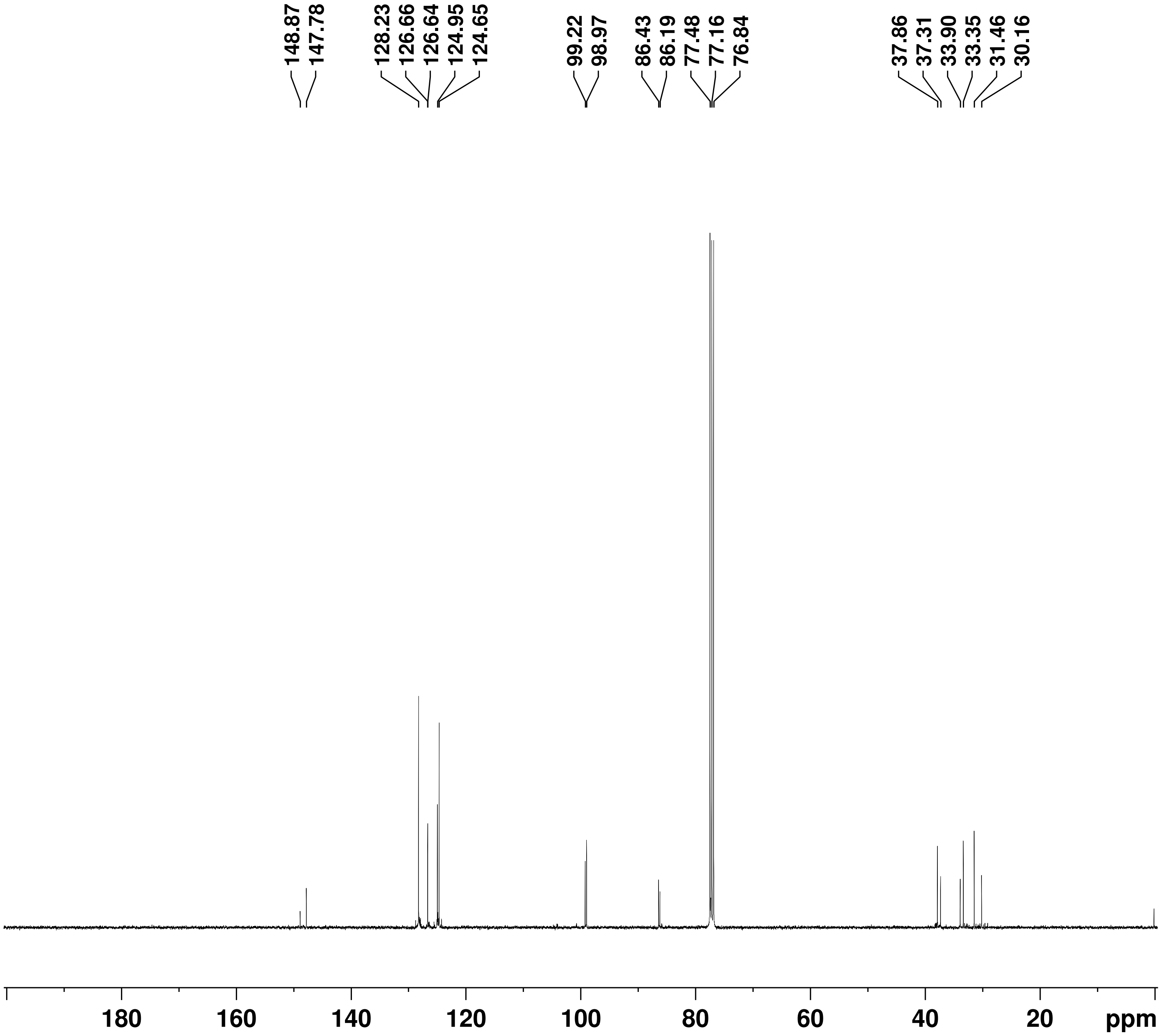

^{13}C spectrum of compounds **S3a** & **S3b**

400 MHz, $CDCl_3$

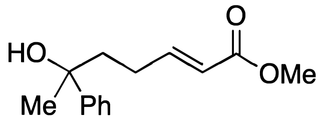

S4

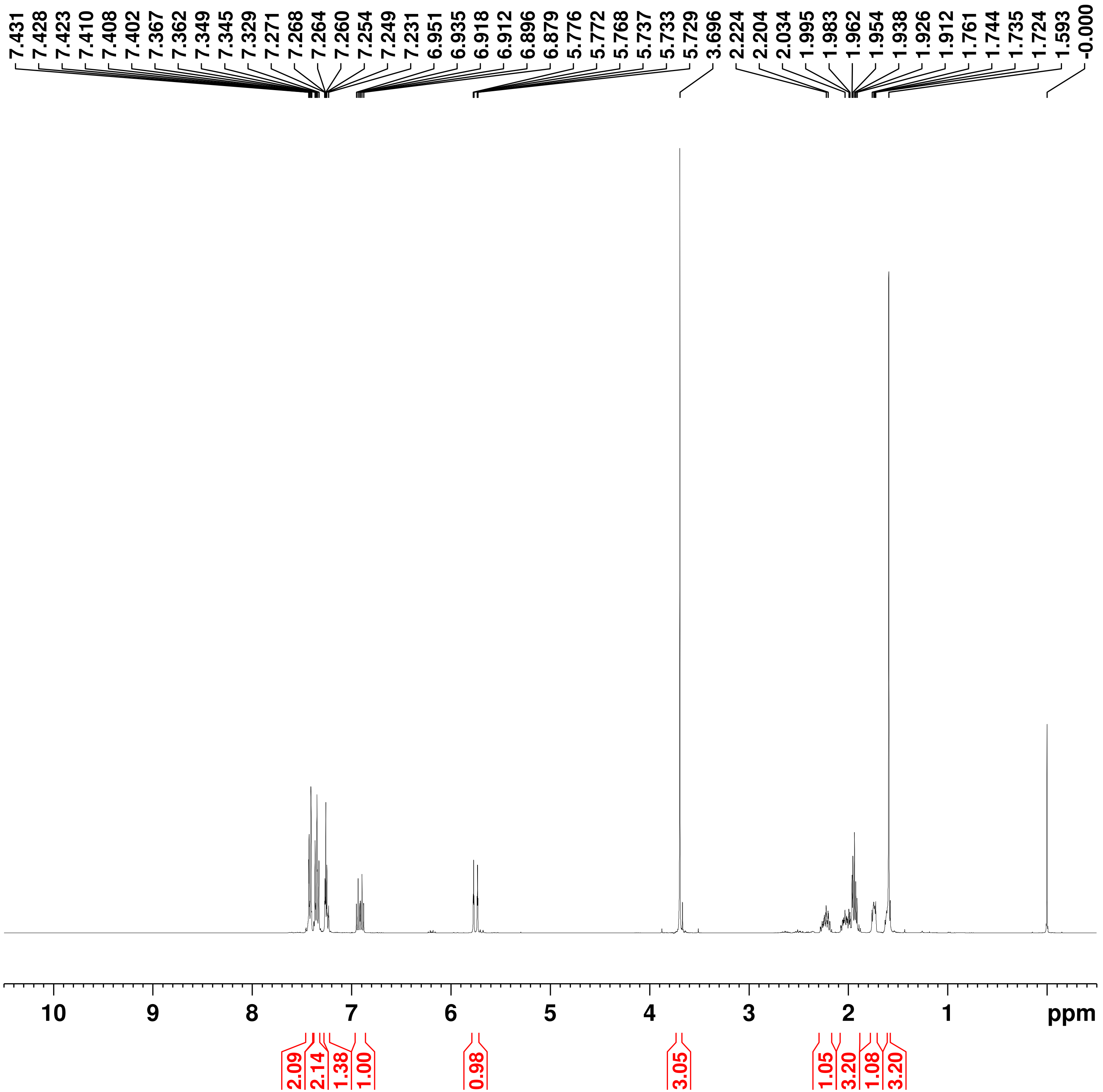

^{1}H spectrum of compound **S4**

100 MHz, $CDCl_3$

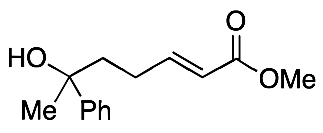

S4

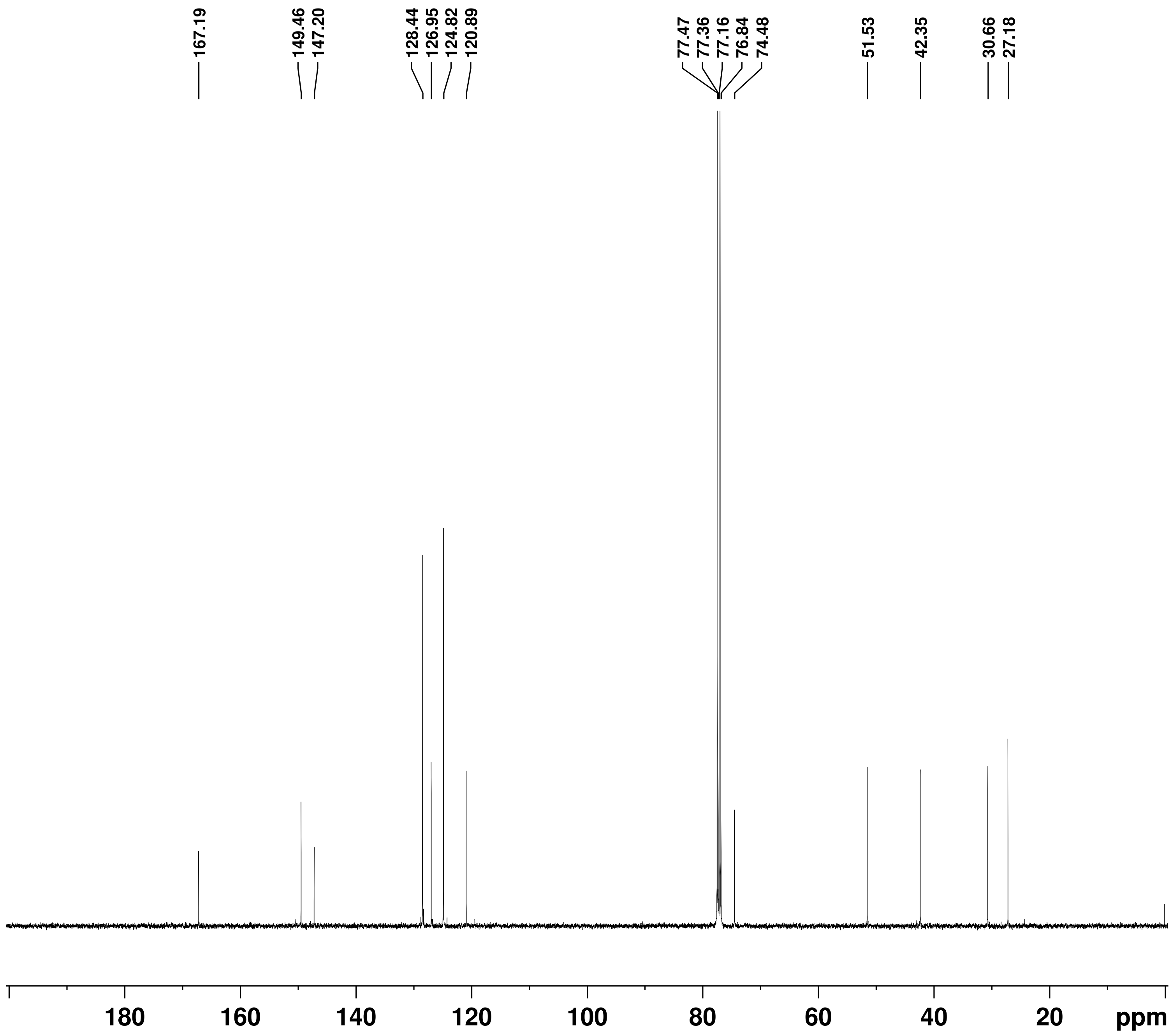

^{13}C spectrum of compound **S4**

100 MHz, $CDCl_3$

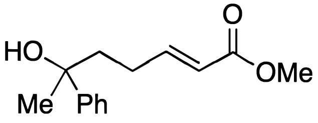

S4

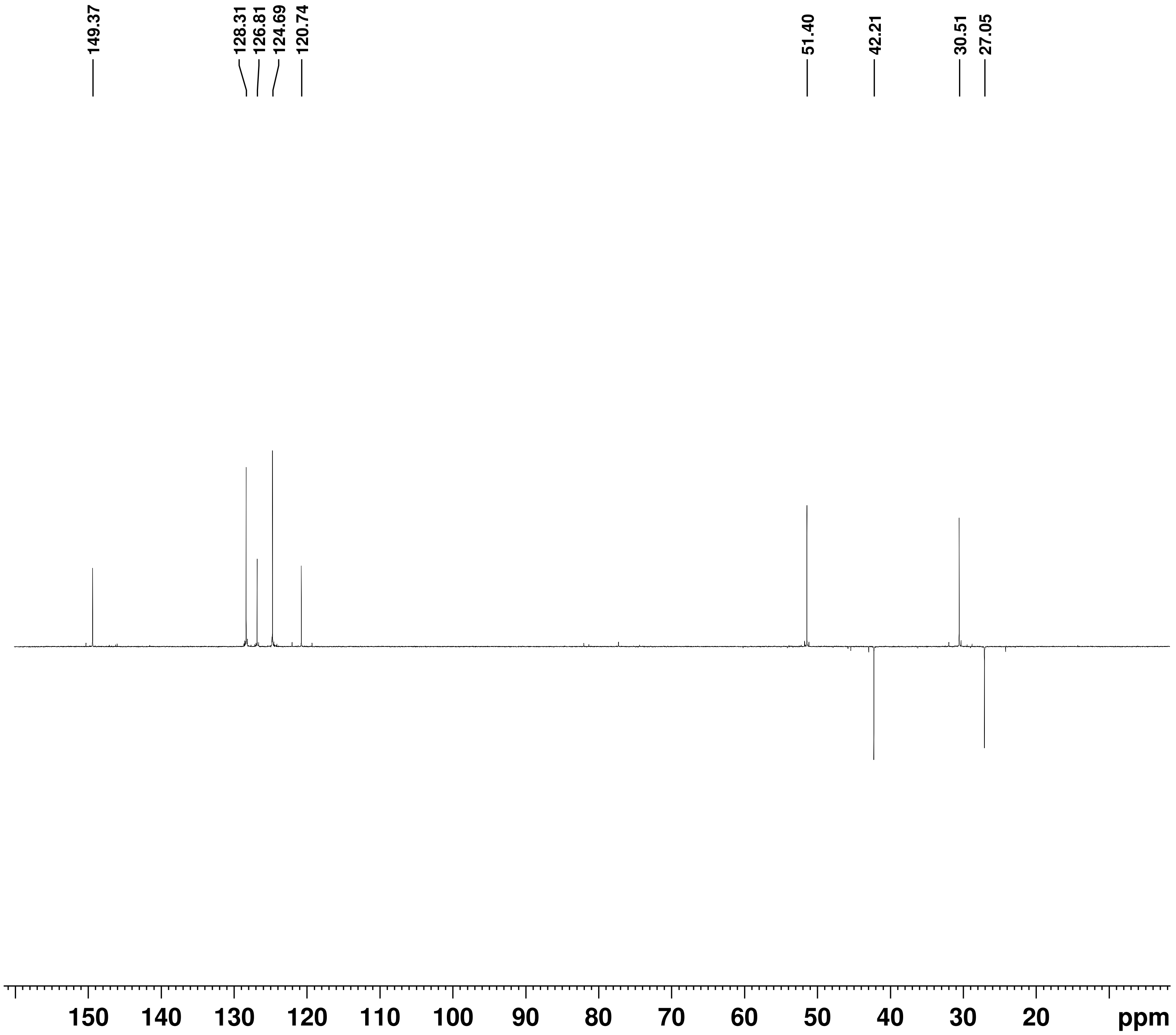

^{13}C DEPT spectrum of compound **S4**

400 MHz, $CDCl_3$

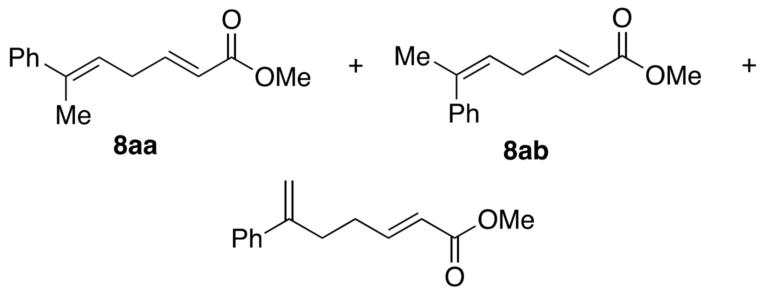

8aa

8ab

8ac

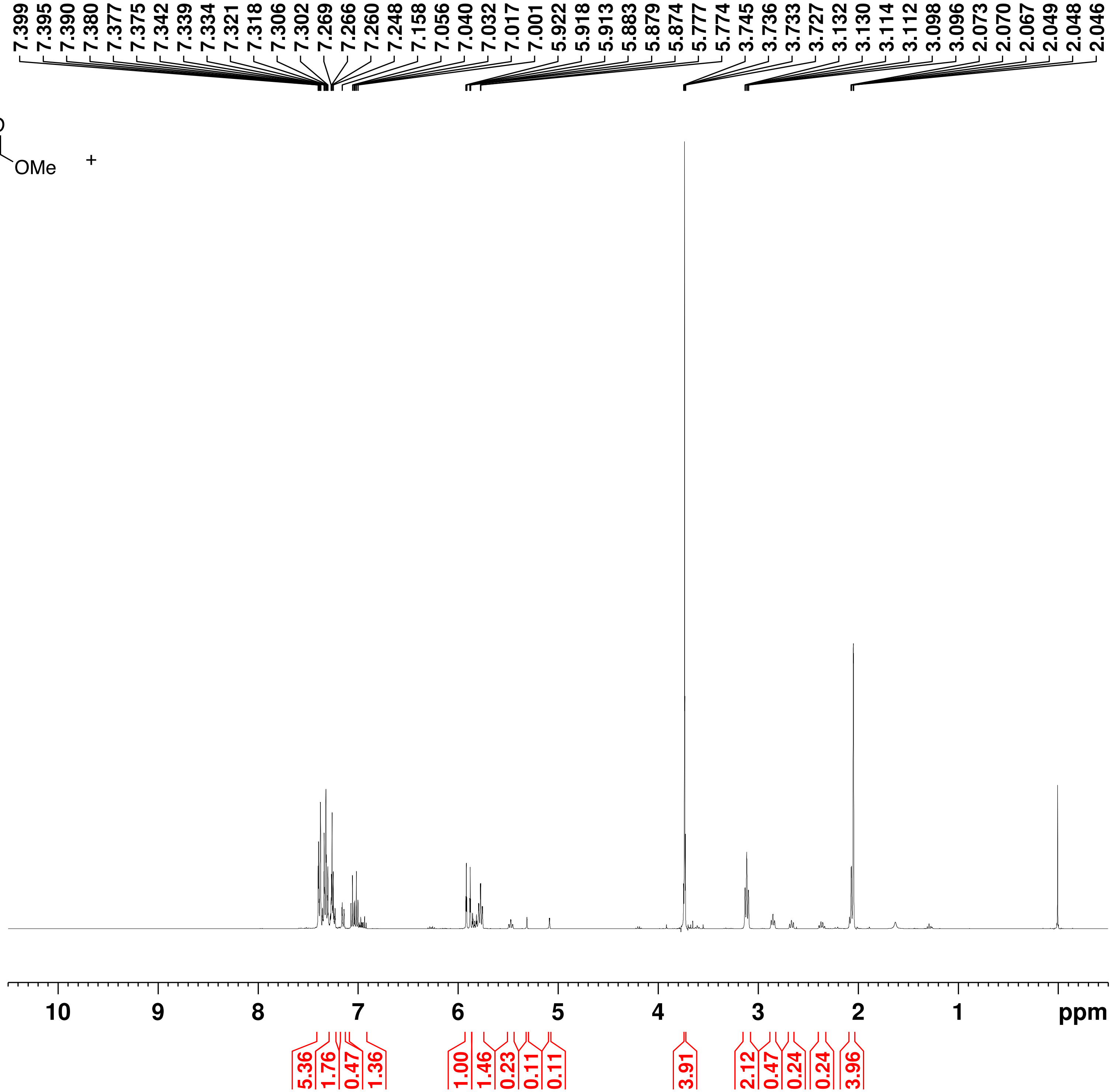

^{1}H spectrum of compounds **8aa**, **8ab**, & **8ac**

100 MHz, $CHCl_3$

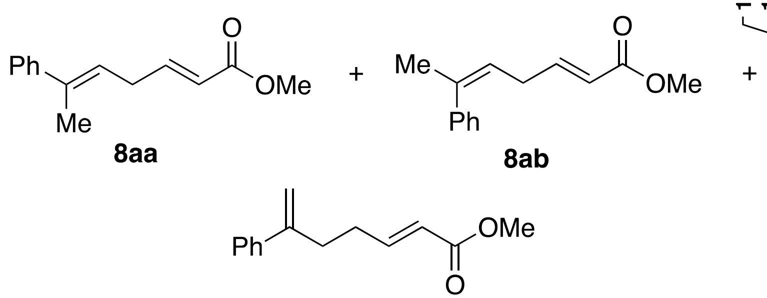

8aa **8ab** **8ac**

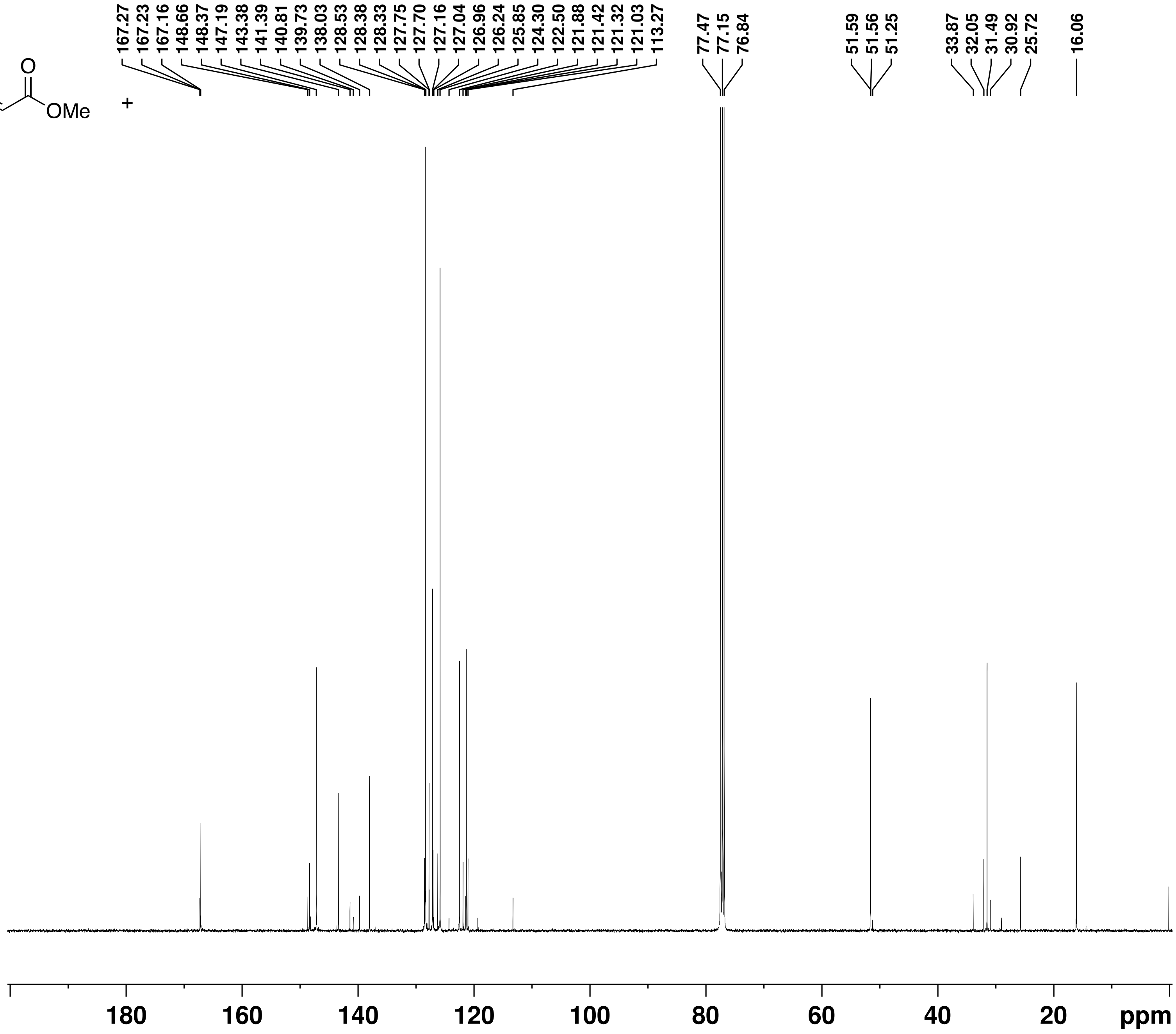

^{13}C spectrum of compounds **8aa**, **8ab** & **8ac**

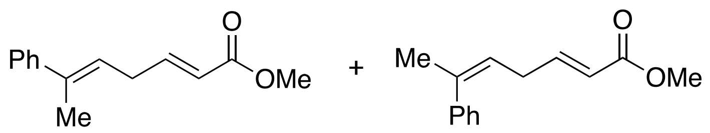

8aa + **8ab**

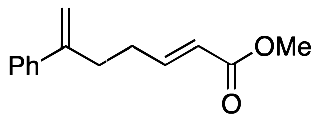

8ac

400 MHz, $CHCl_3$

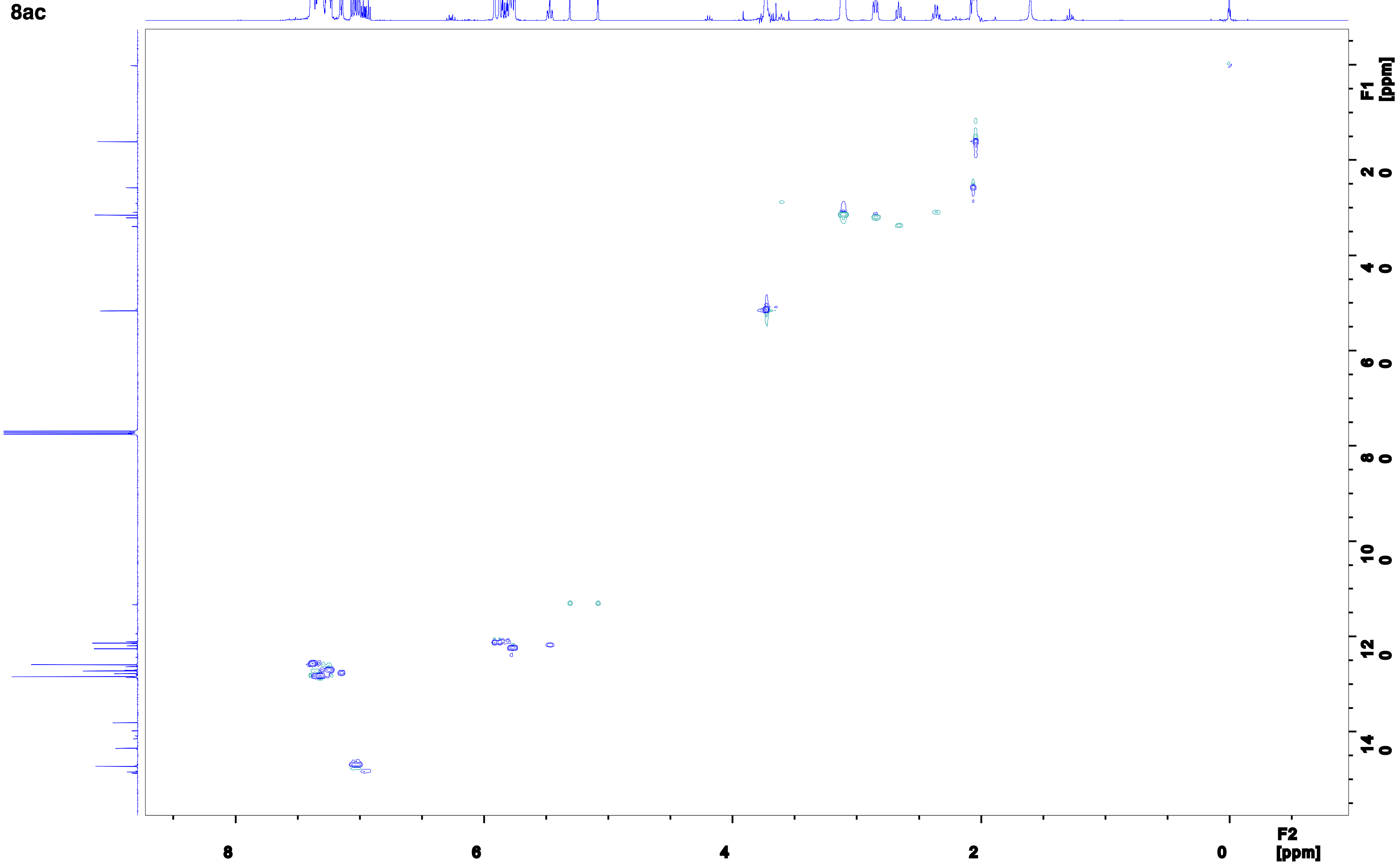

$^{1}H/^{13}C$ HSQC of compounds **8aa**, **8ab** & **8ac**

400 MHz, $CHCl_3$

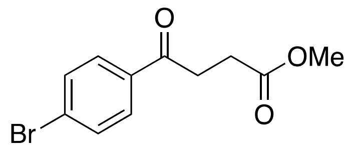

S6

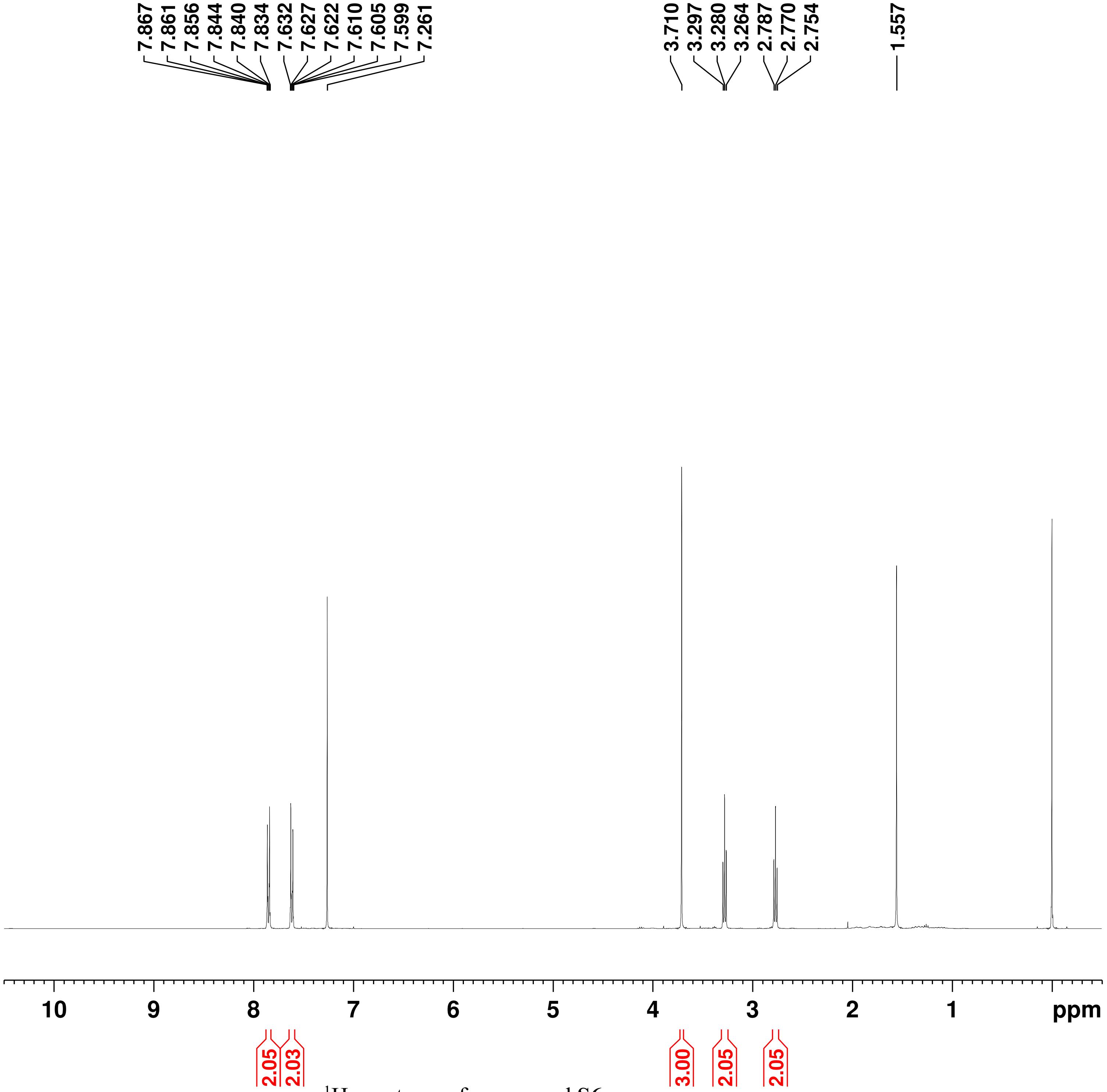

^{1}H spectrum of compound **S6**

100 MHz, $CHCl_3$

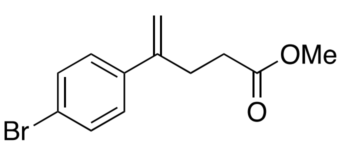

S7

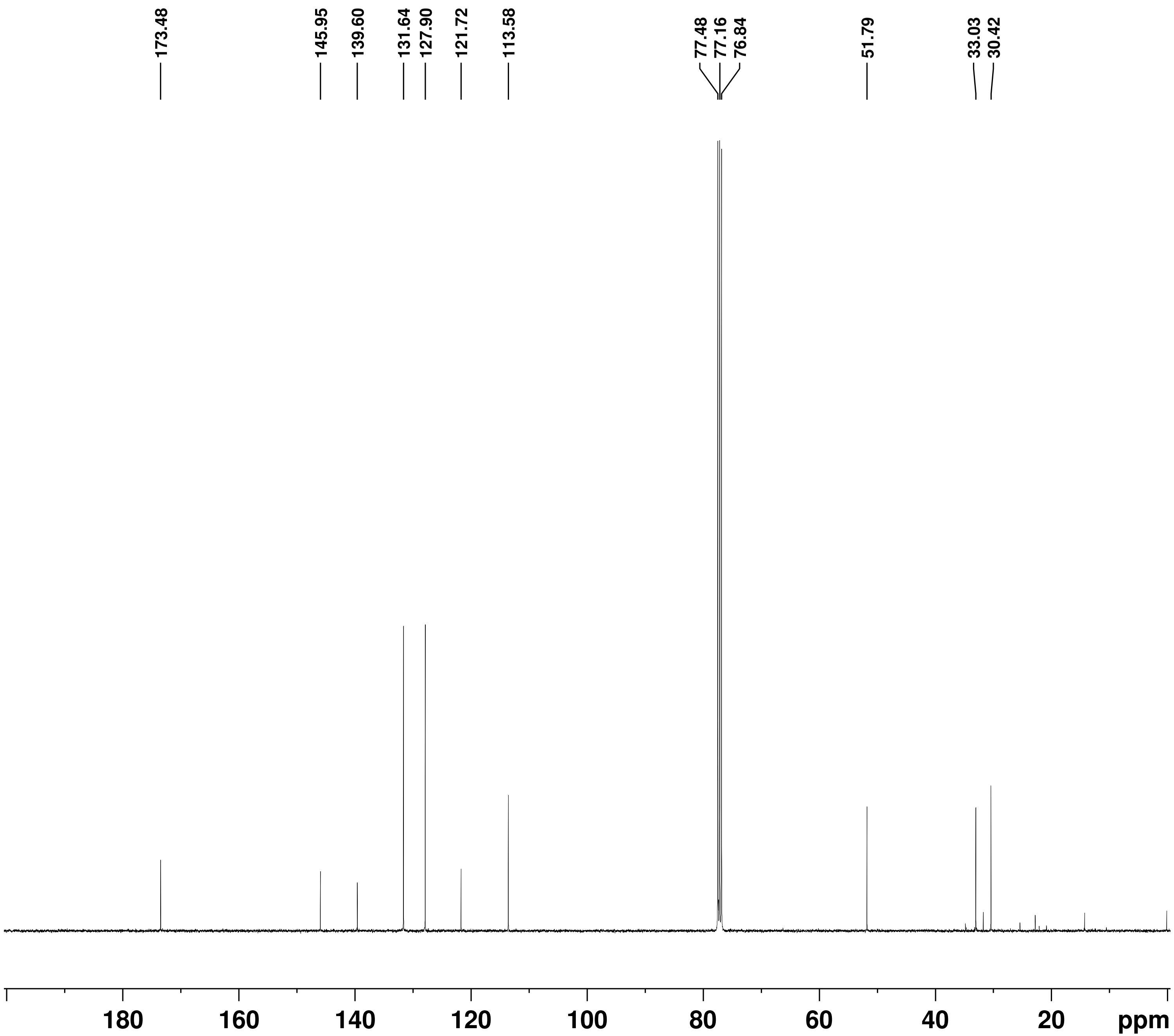

^{13}C spectrum of compound **S7**

400 MHz, $CHCl_3$

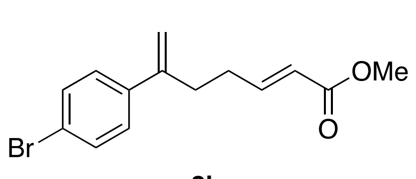

8ba

+

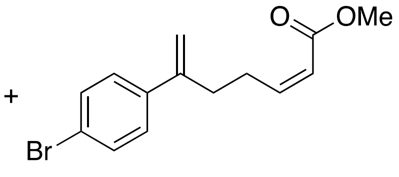

8bb

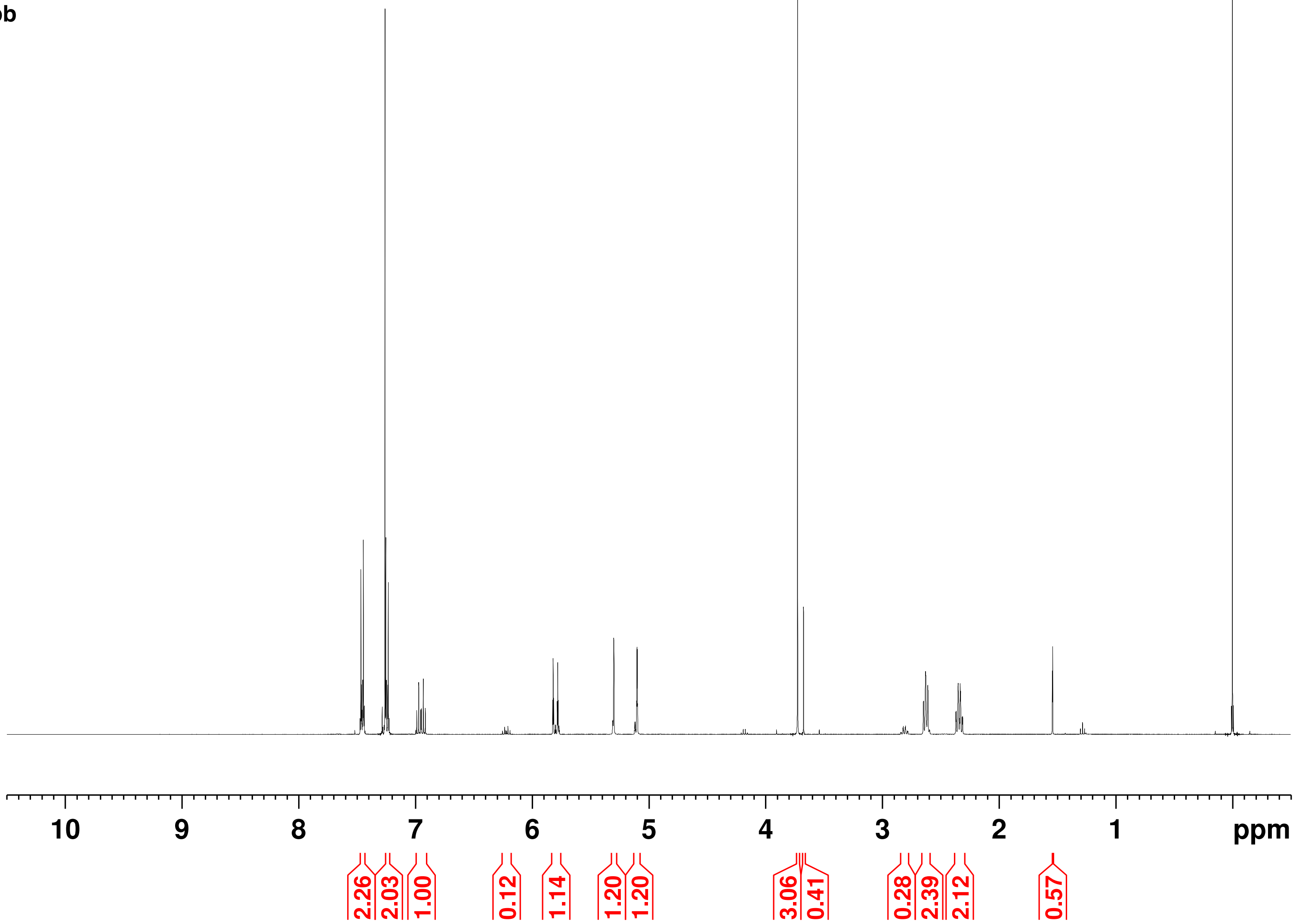

^1H spectrum of compounds **8ba & 8bb**

400 MHz, $CHCl_3$

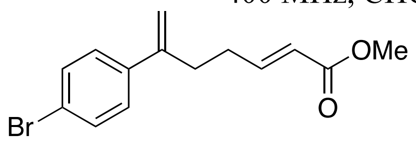

8ba

+

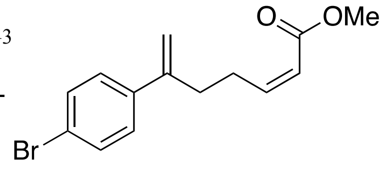

8bb

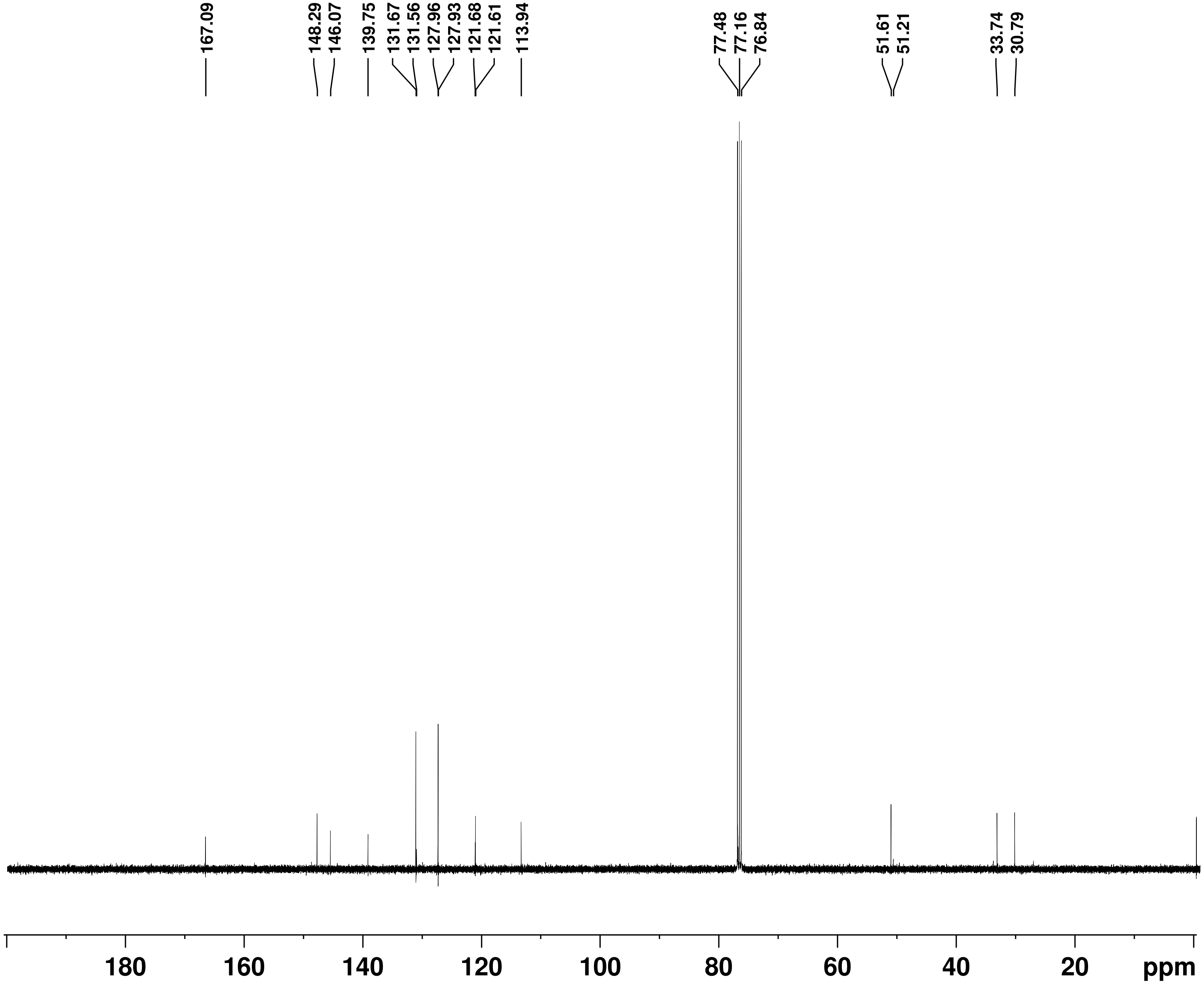

^{13}C spectrum of compounds of **8ba & 8bb**

400 MHz, $CHCl_3$

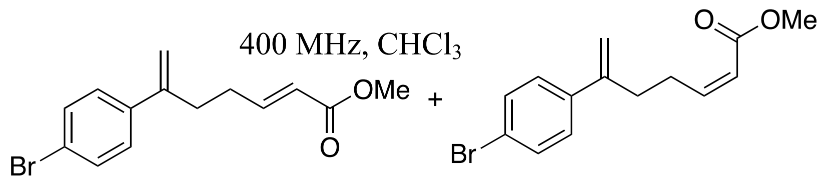

8ba **8bb**

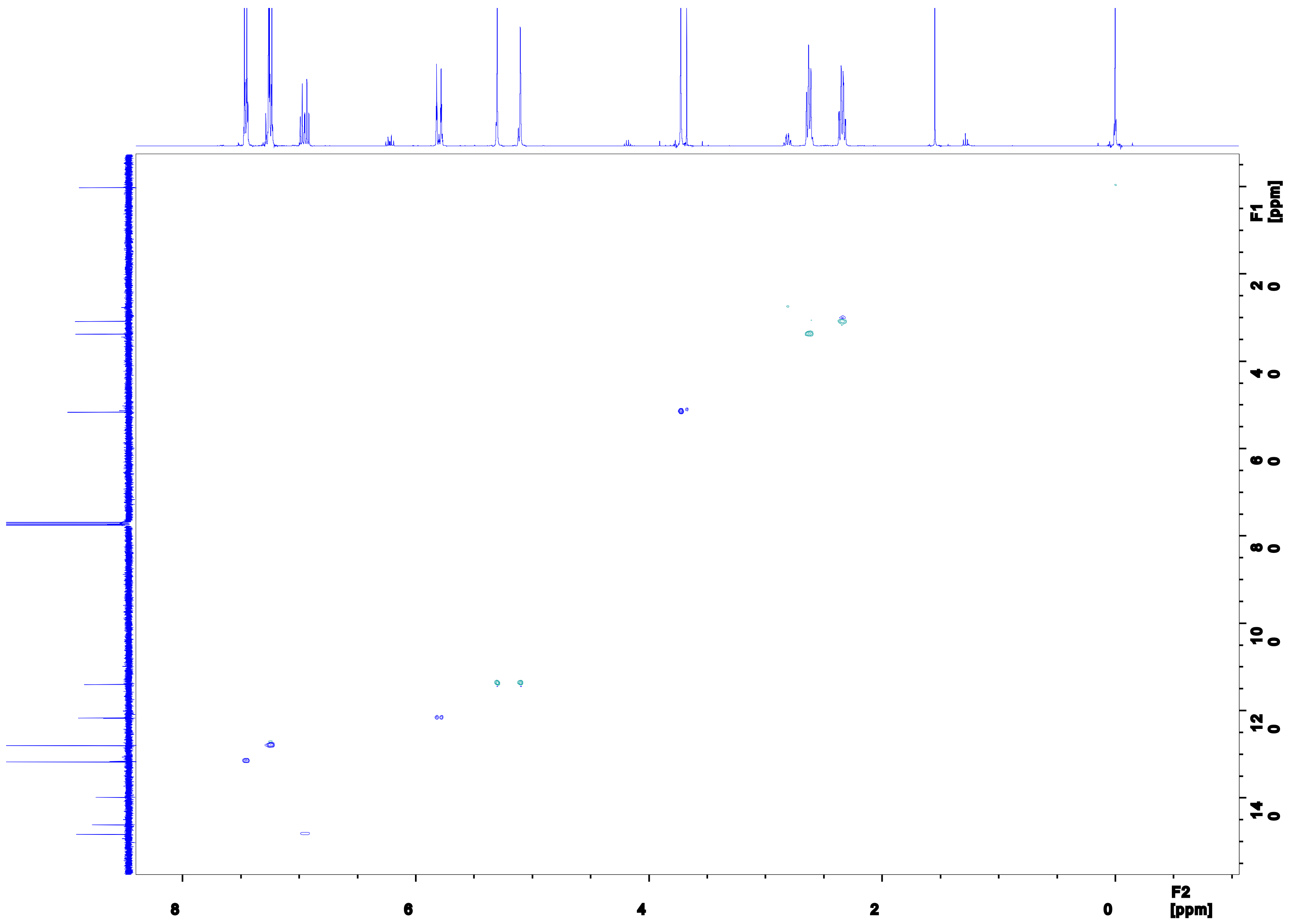

$^{1}H/^{13}C$ HSQC spectrum of compounds **8ba & 8bb**

100 MHz, $CHCl_3$

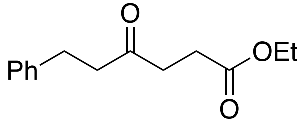

S9

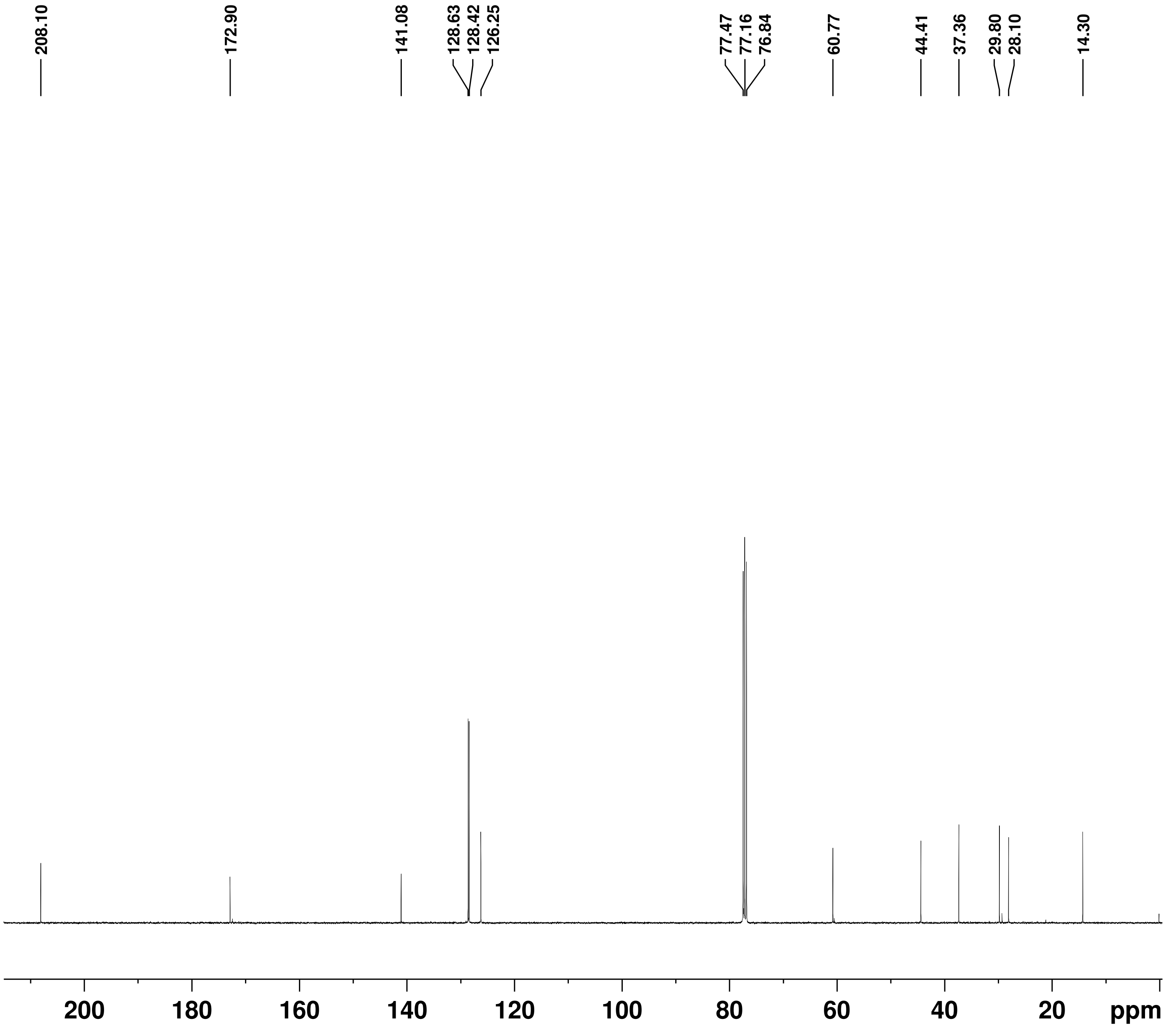

^{13}C spectrum of compound **S9**

400 MHz, $CHCl_3$

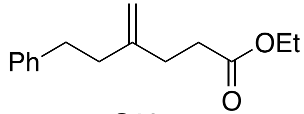

S10

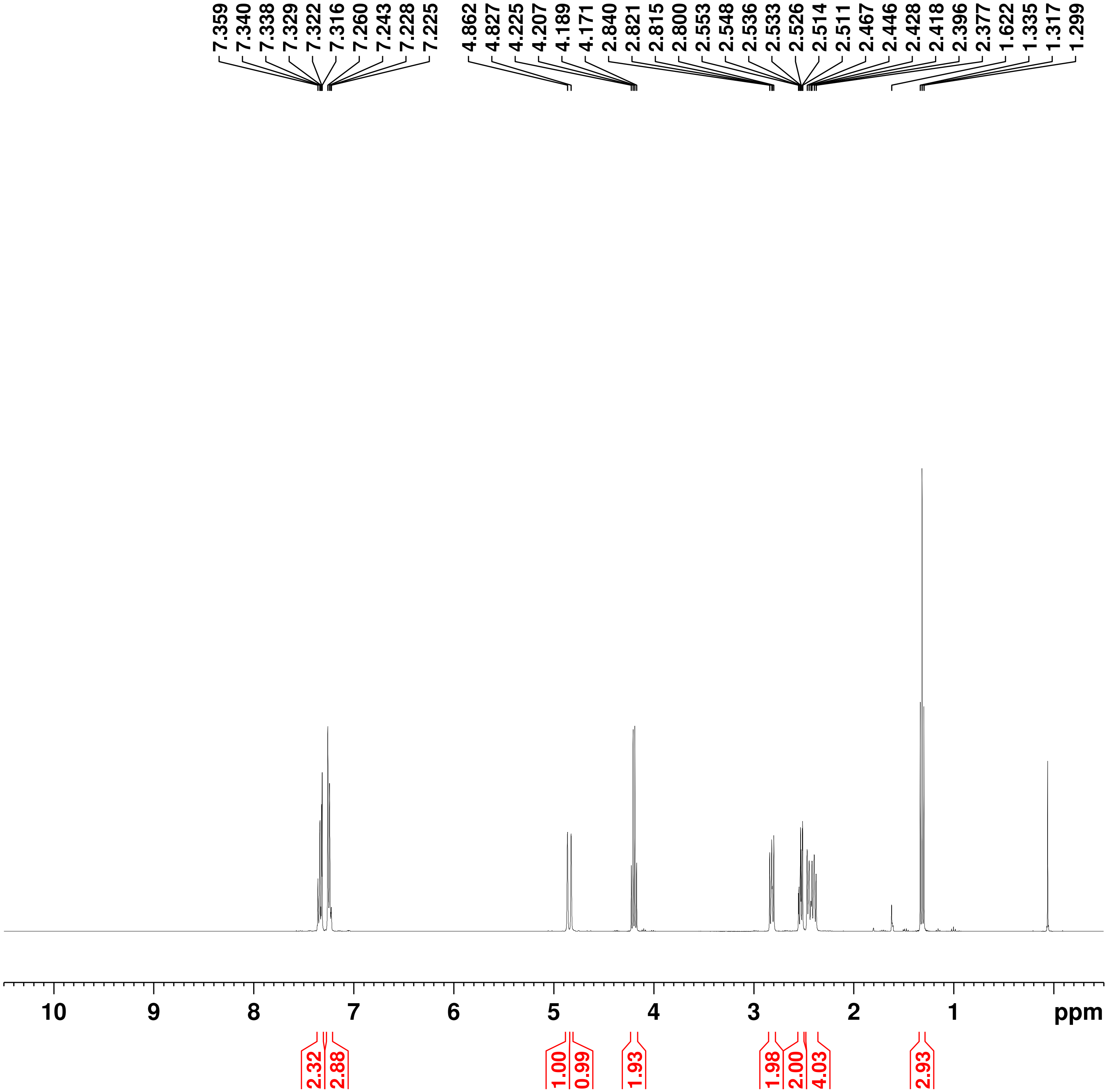

^1H spectrum of compound **S10**

400 MHz, $CHCl_3$

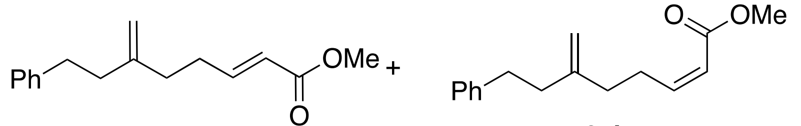

8ca **8cb**

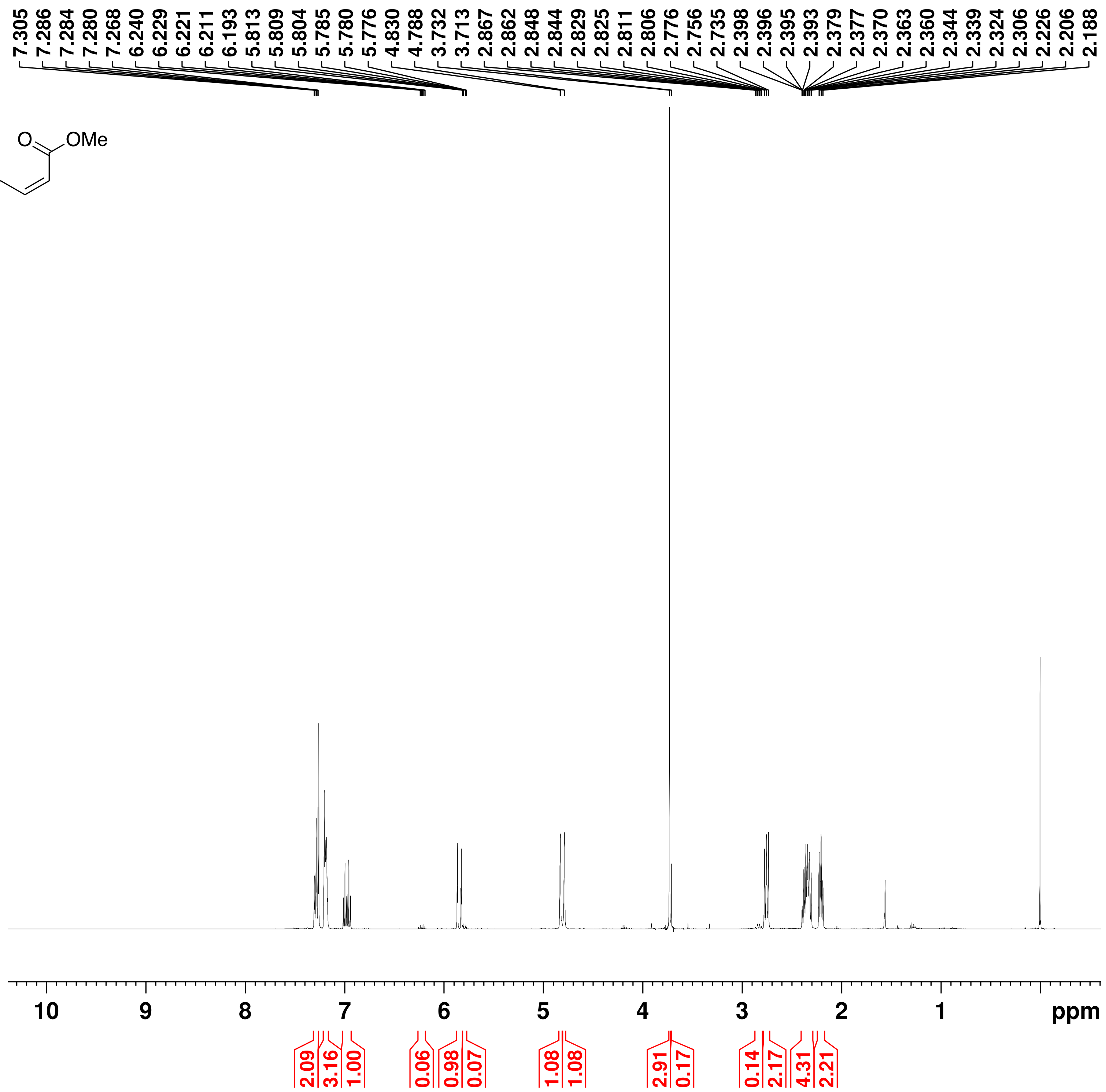

^{1}H spectrum of compounds **8ca & 8cb**

100 MHz, $CHCl_3$

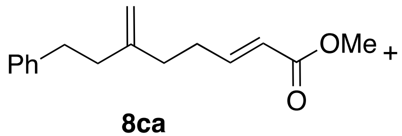

8ca

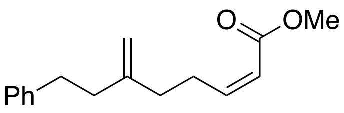

8cb

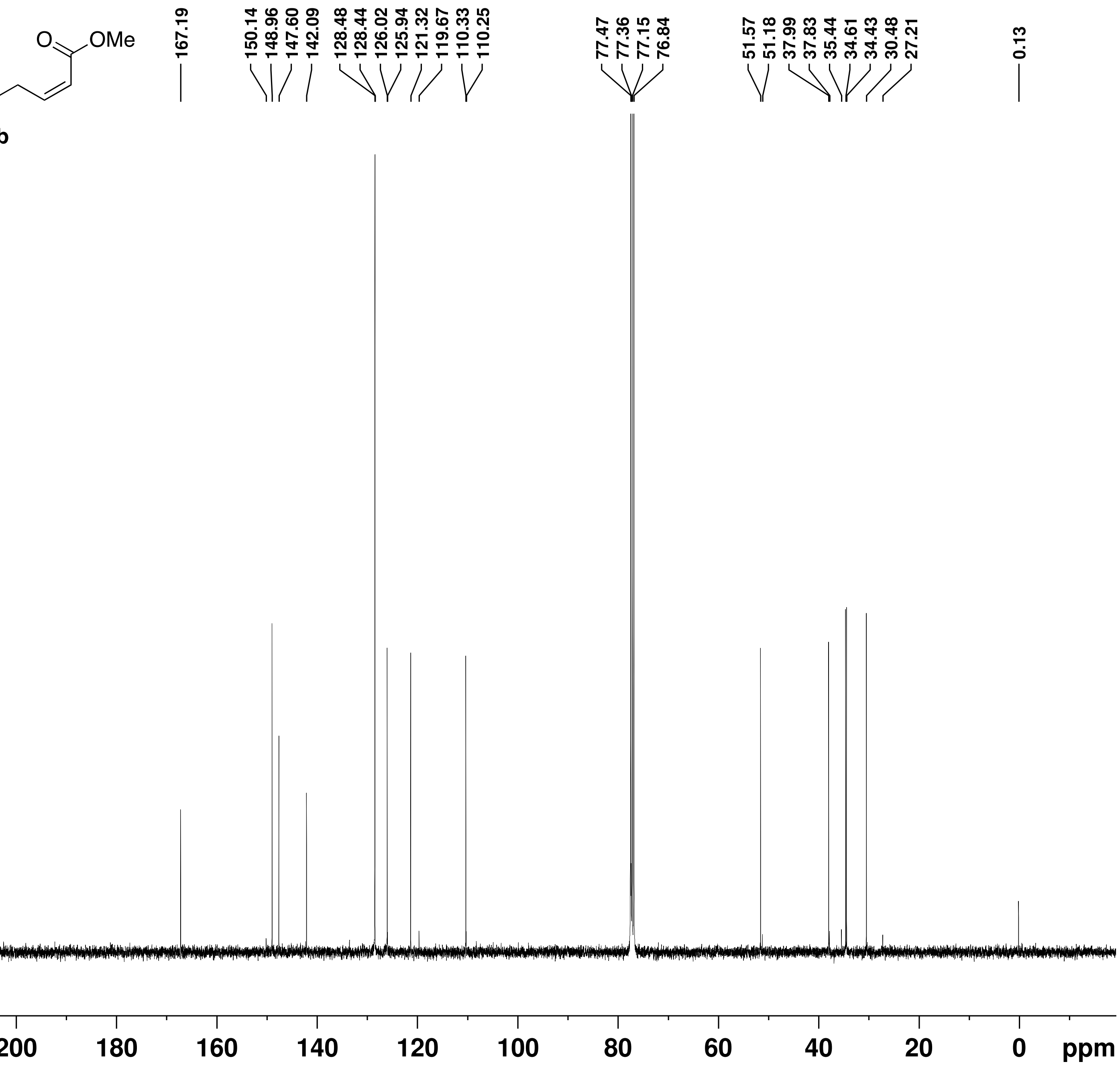

^{13}C spectrum of compounds of **8ca & 8cb**

400 MHz, $CHCl_3$

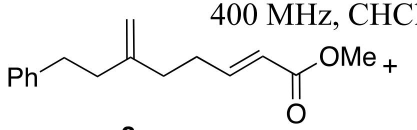

8ca

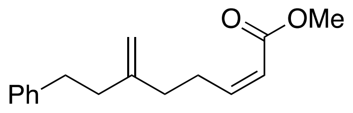

8cb

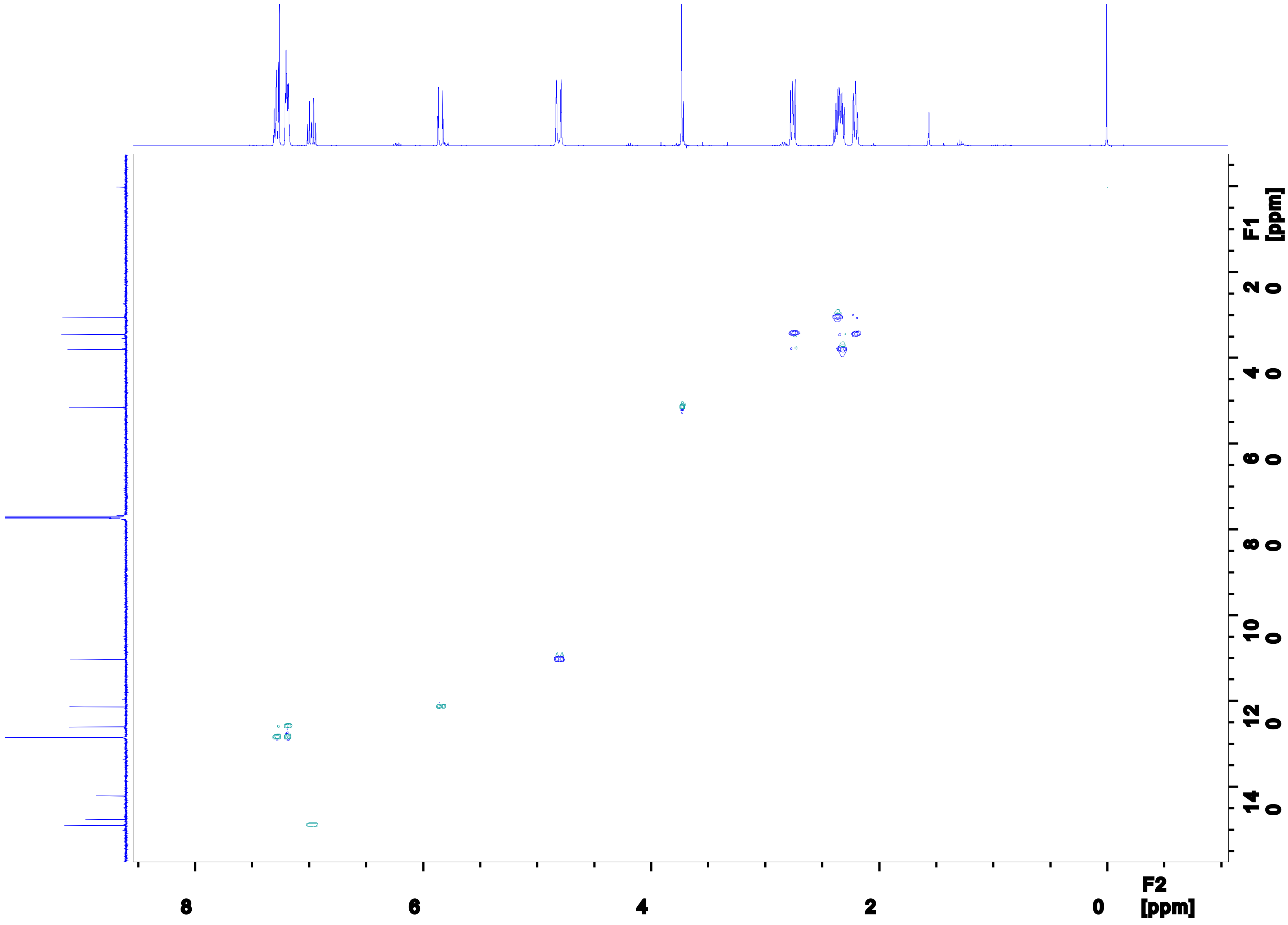

$^{1}H/^{13}C$ HSQC spectrum of **8ca & 8cb**

100 MHz, $CHCl_3$

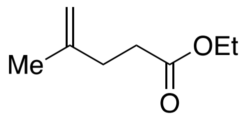

S12

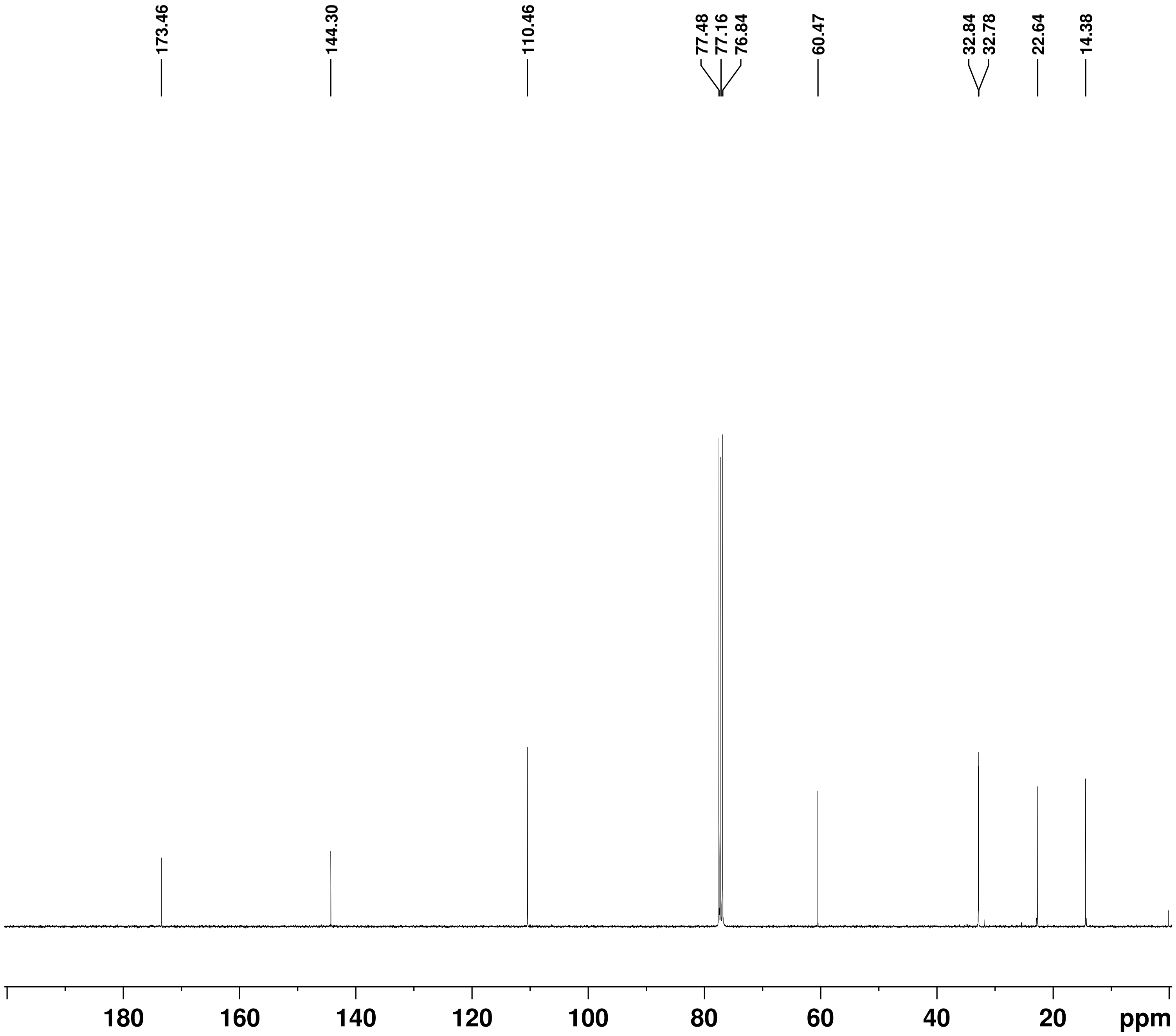

^{13}C spectrum of compound **S12**

100 MHz, $CHCl_3$

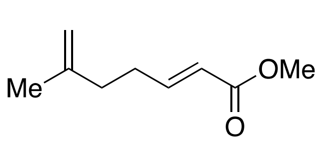

8d

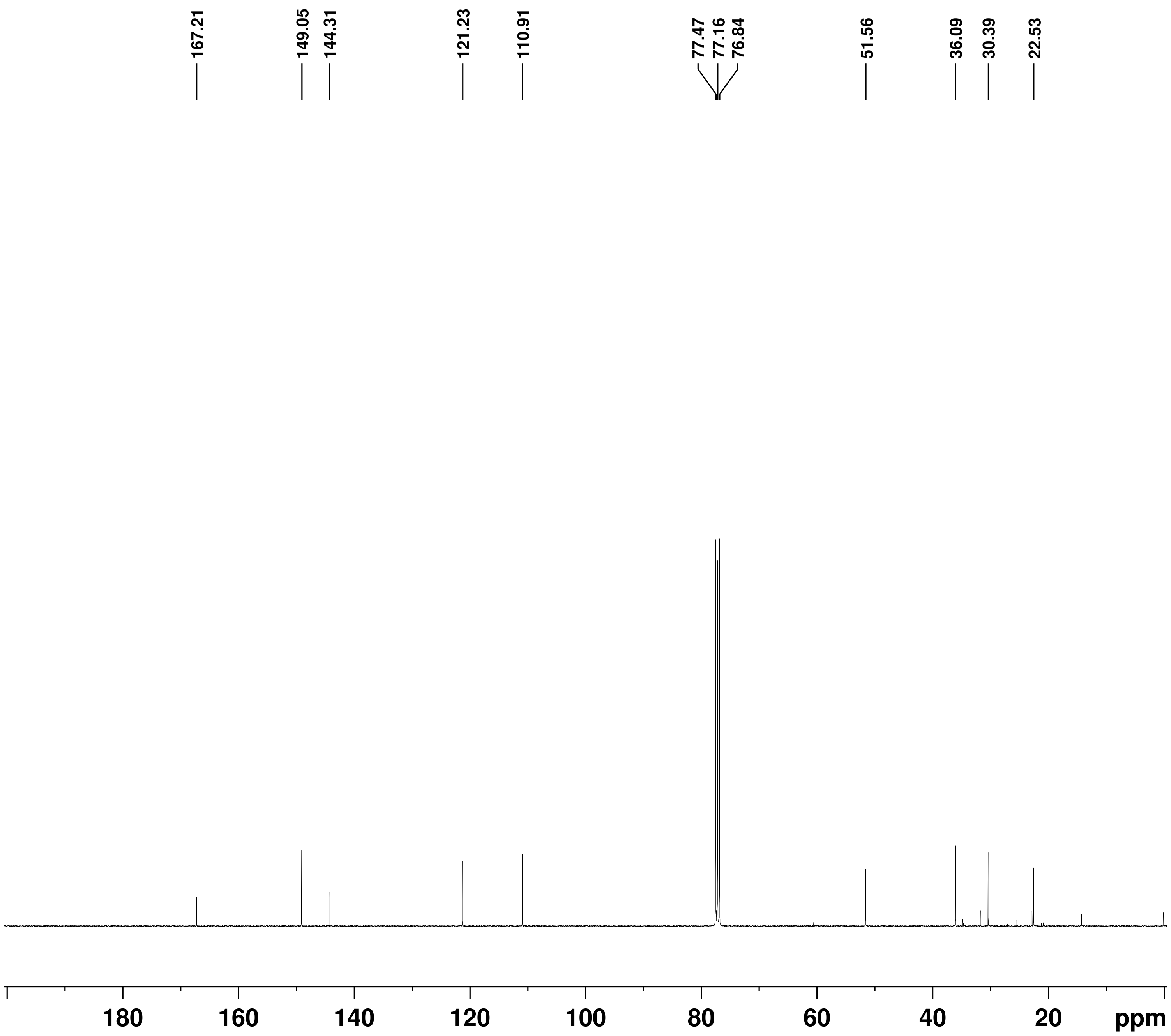

^{1}H spectrum of compound **8d**

100 MHz, $CHCl_3$

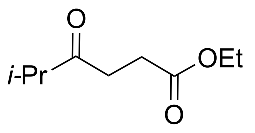

S15

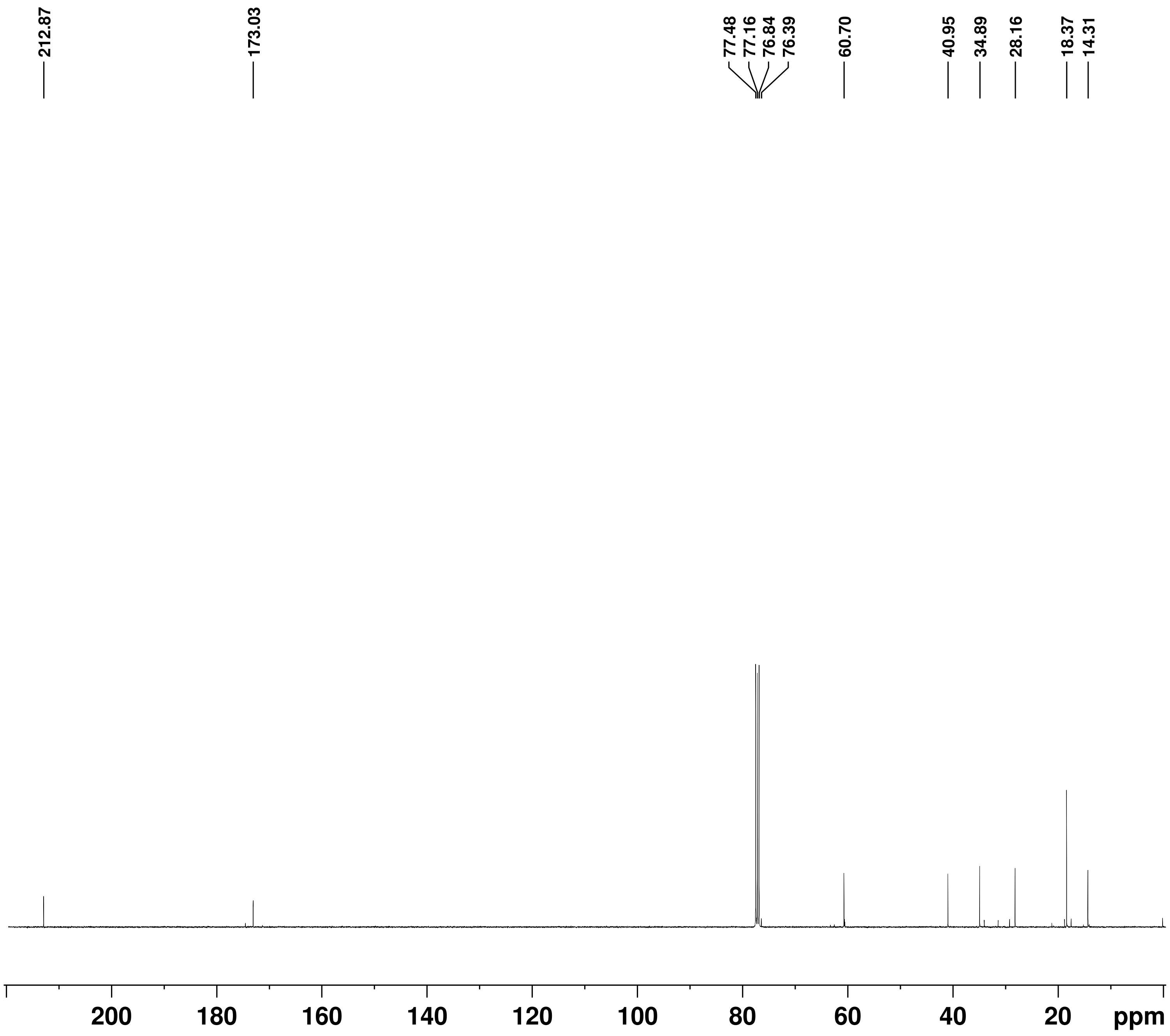

^{13}C spectrum of compound **S15**

100 MHz, $CHCl_3$

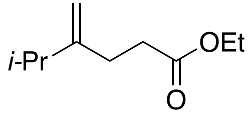

S16

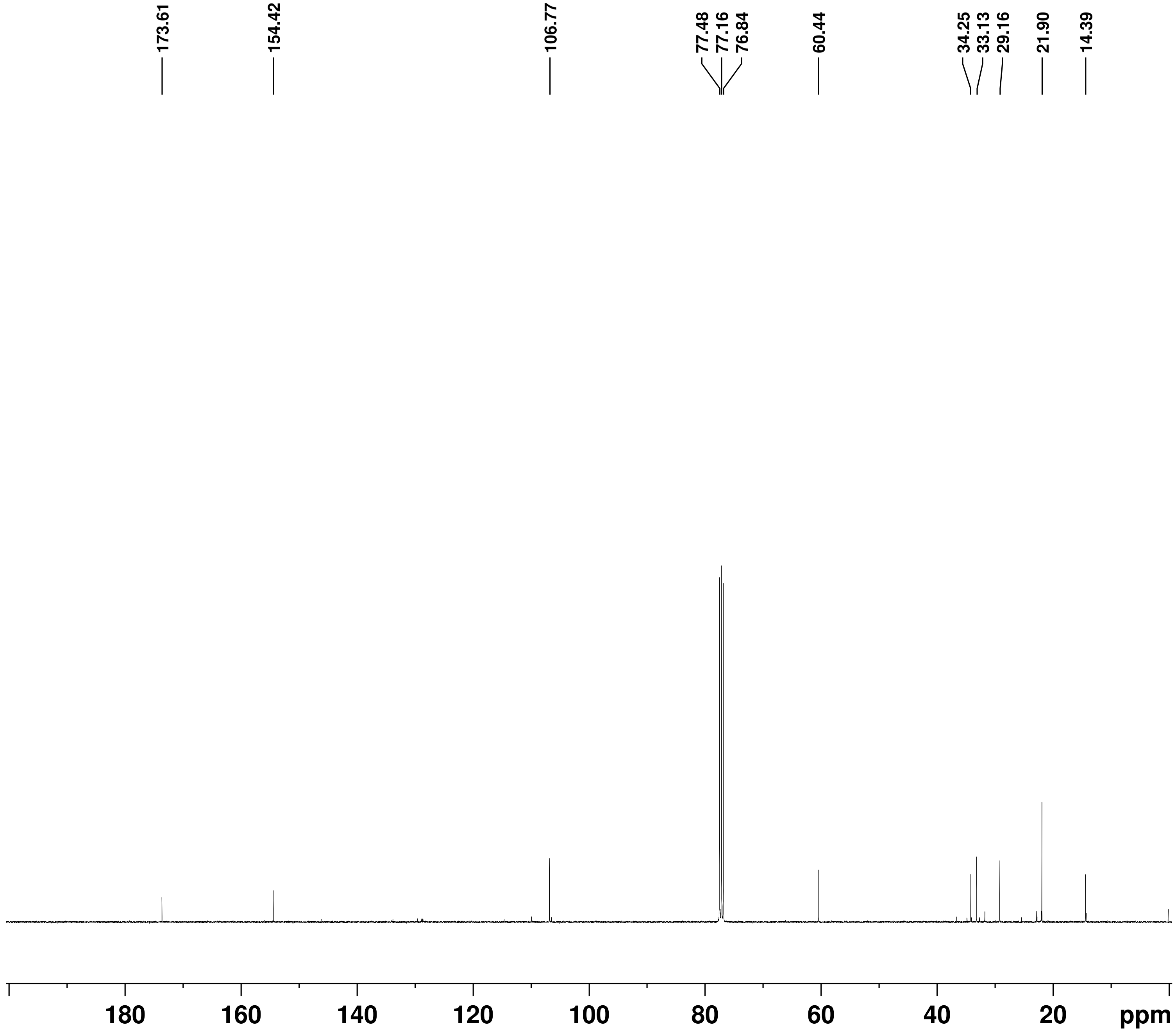

^{13}C spectrum of compound **S16**

400 MHz, $CHCl_3$

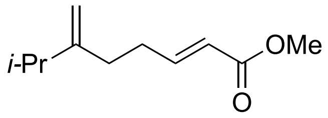

8ea

+

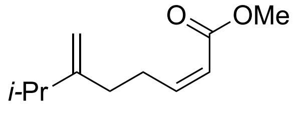

8eb

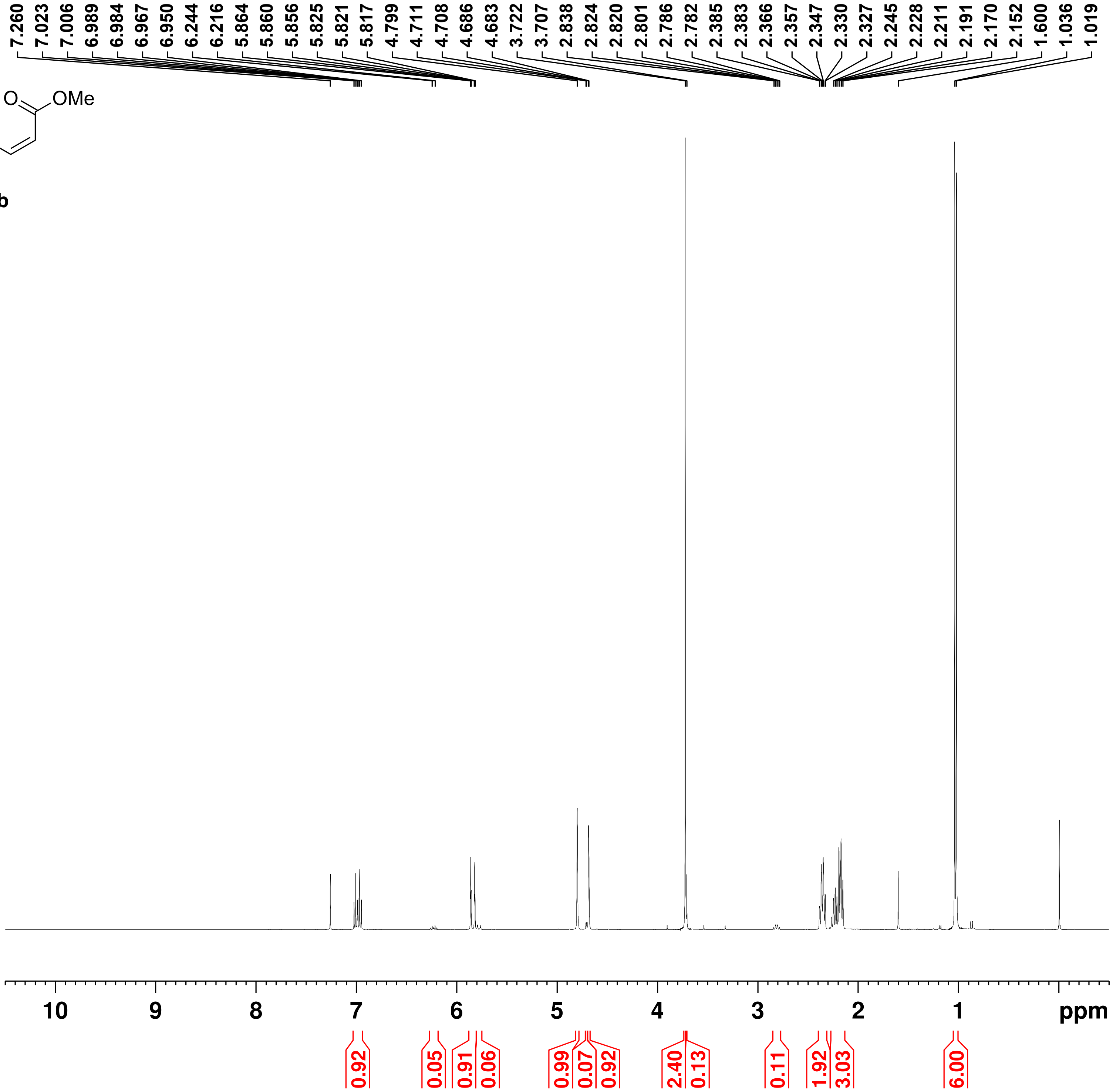

^{1}H spectrum of compounds **8ea & 8eb**

100 MHz, $CHCl_3$

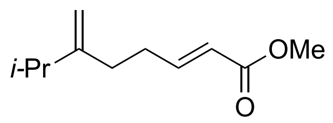

8ea

+

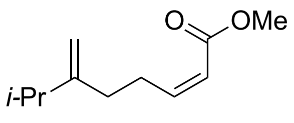

8eb

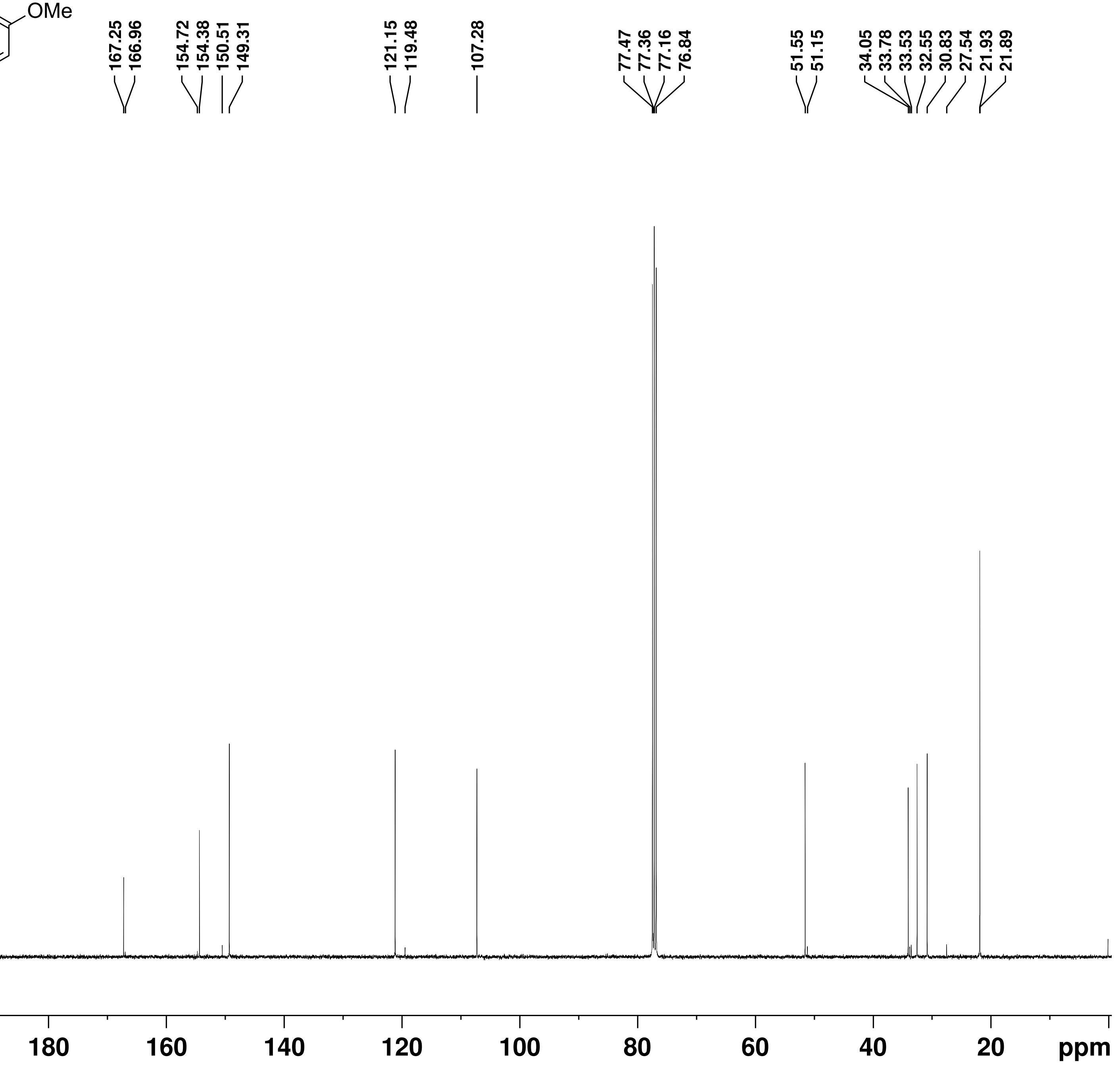

^{13}C spectrum of compound **8ea & 8eb**

100 MHz, $CHCl_3$

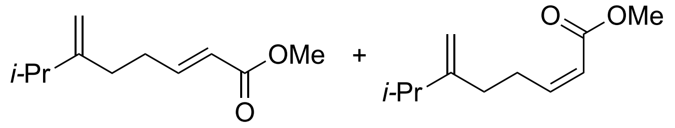

8ea **8eb**

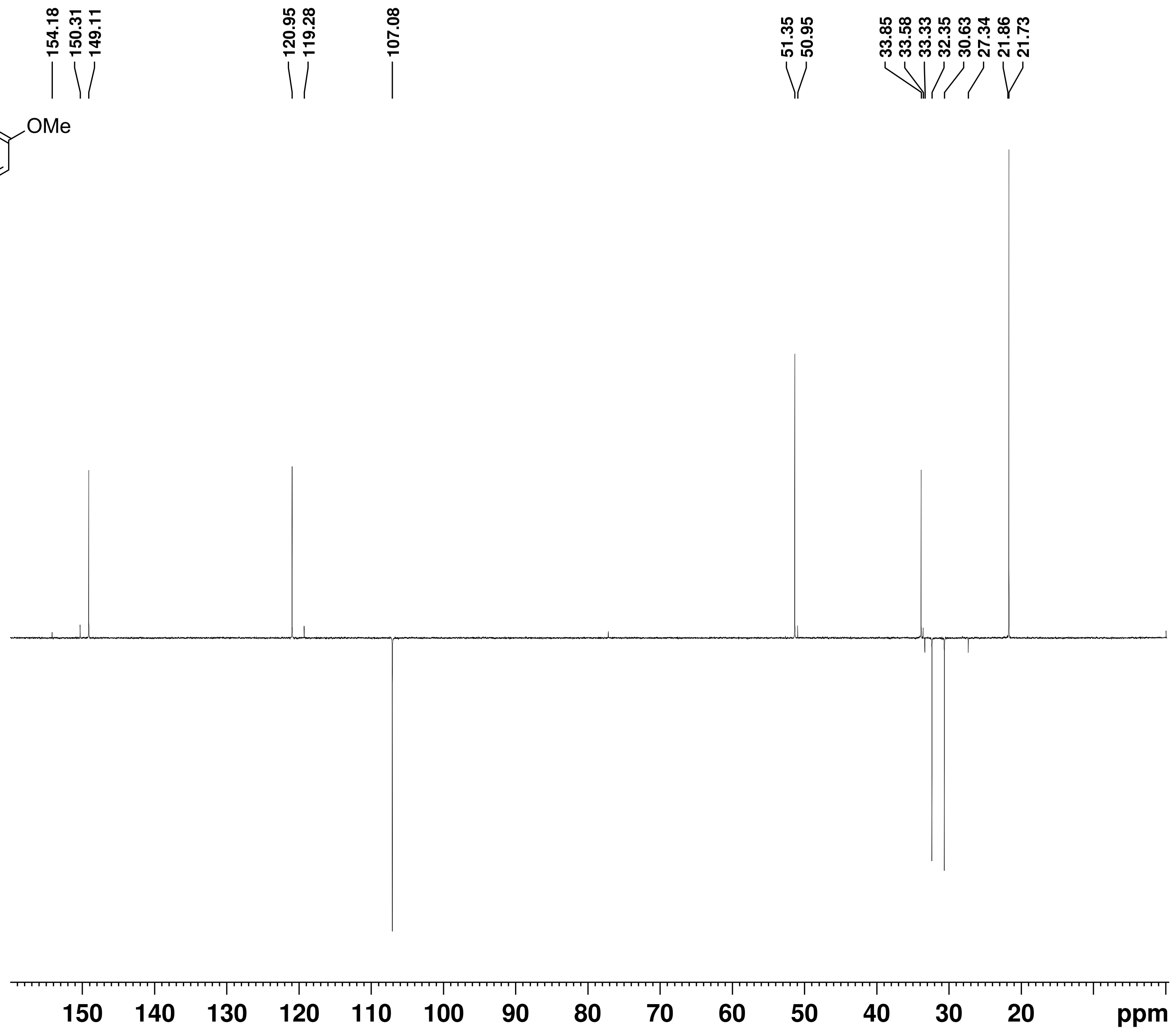

^{13}C DEPT of compounds **8ea & 8eb**

100 MHz, $CHCl_3$

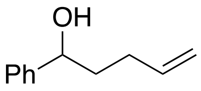

S18

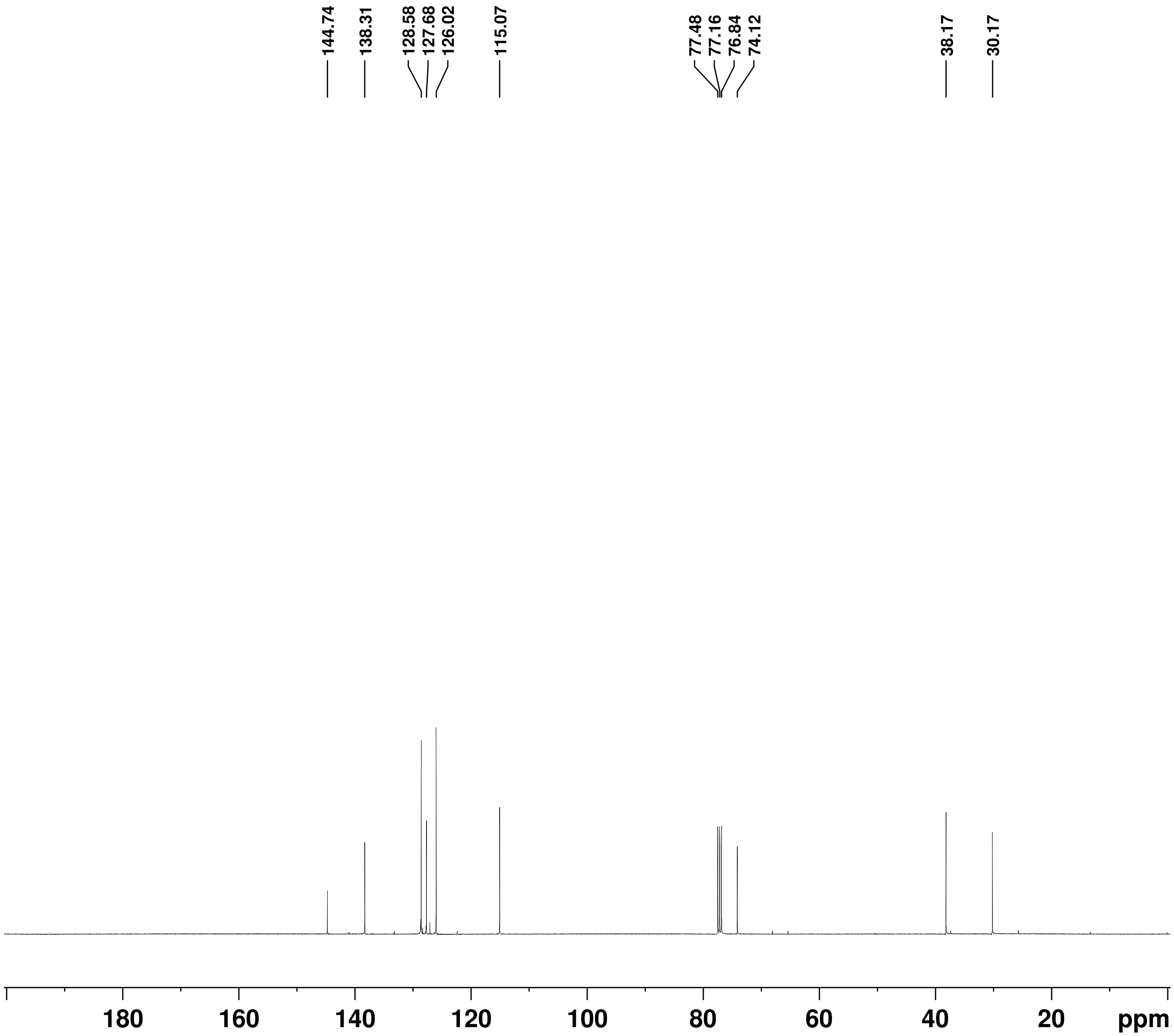

^{13}C spectrum of compound **S18**

100 MHz, $CHCl_3$

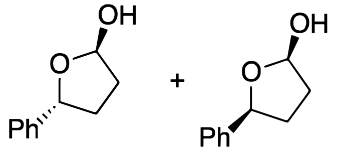

S19a + **S19b**

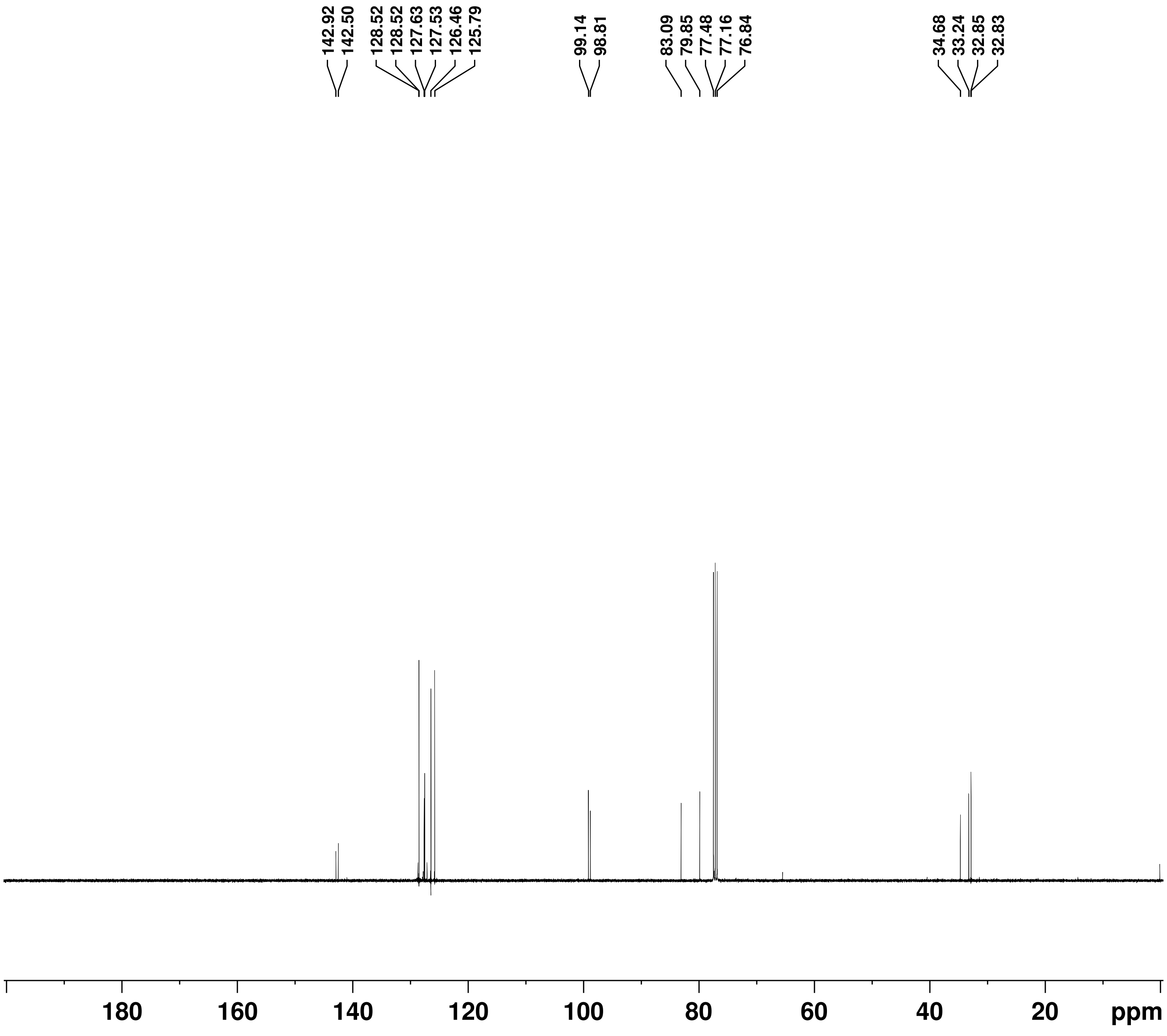

^{13}C spectrum of compounds **S19a & S19b**

100 MHz, $CHCl_3$

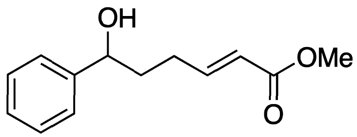

S20

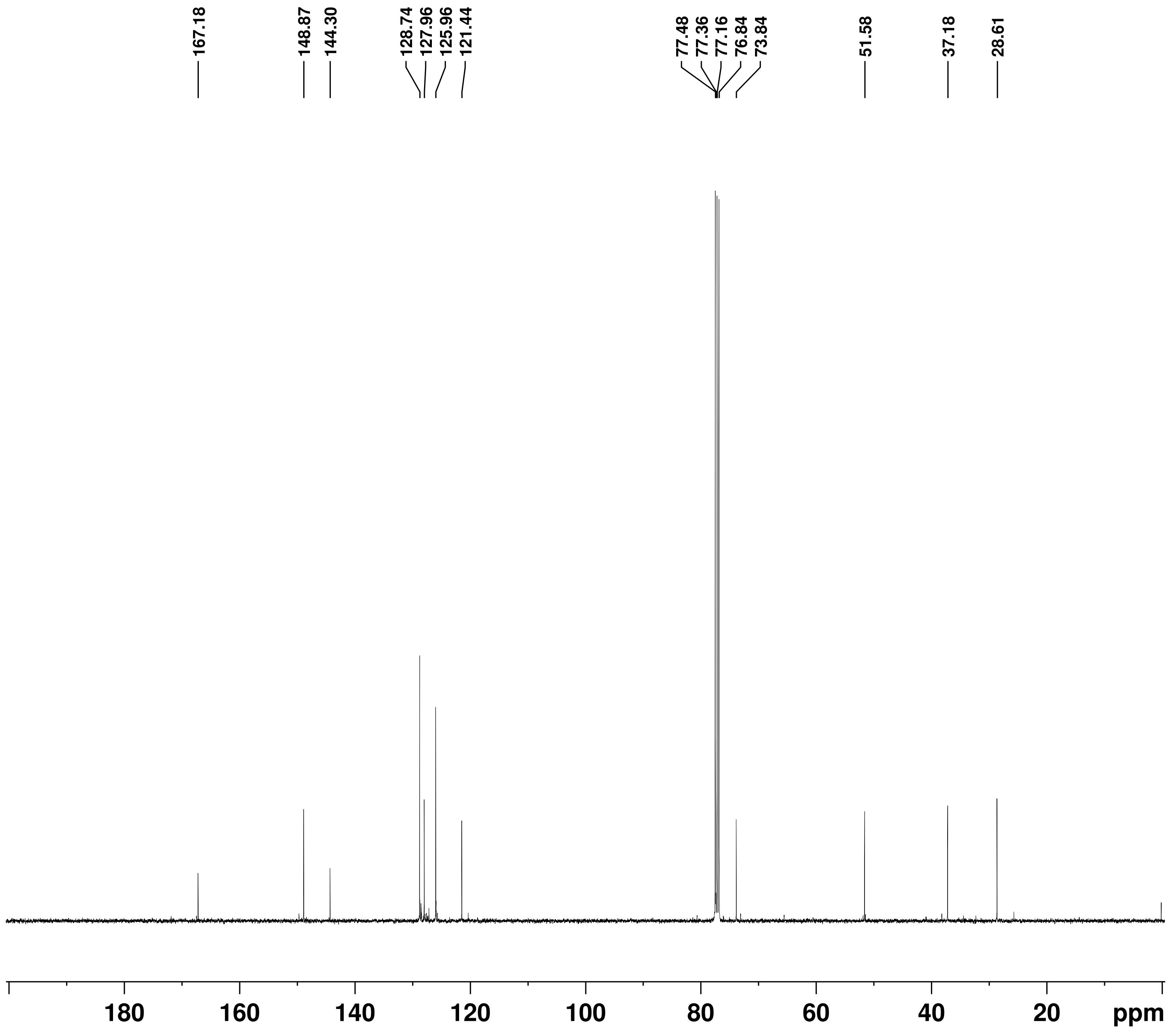

^{13}C spectrum of compound **S20**

400 MHz, $CHCl_3$

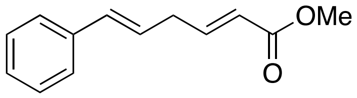

8f

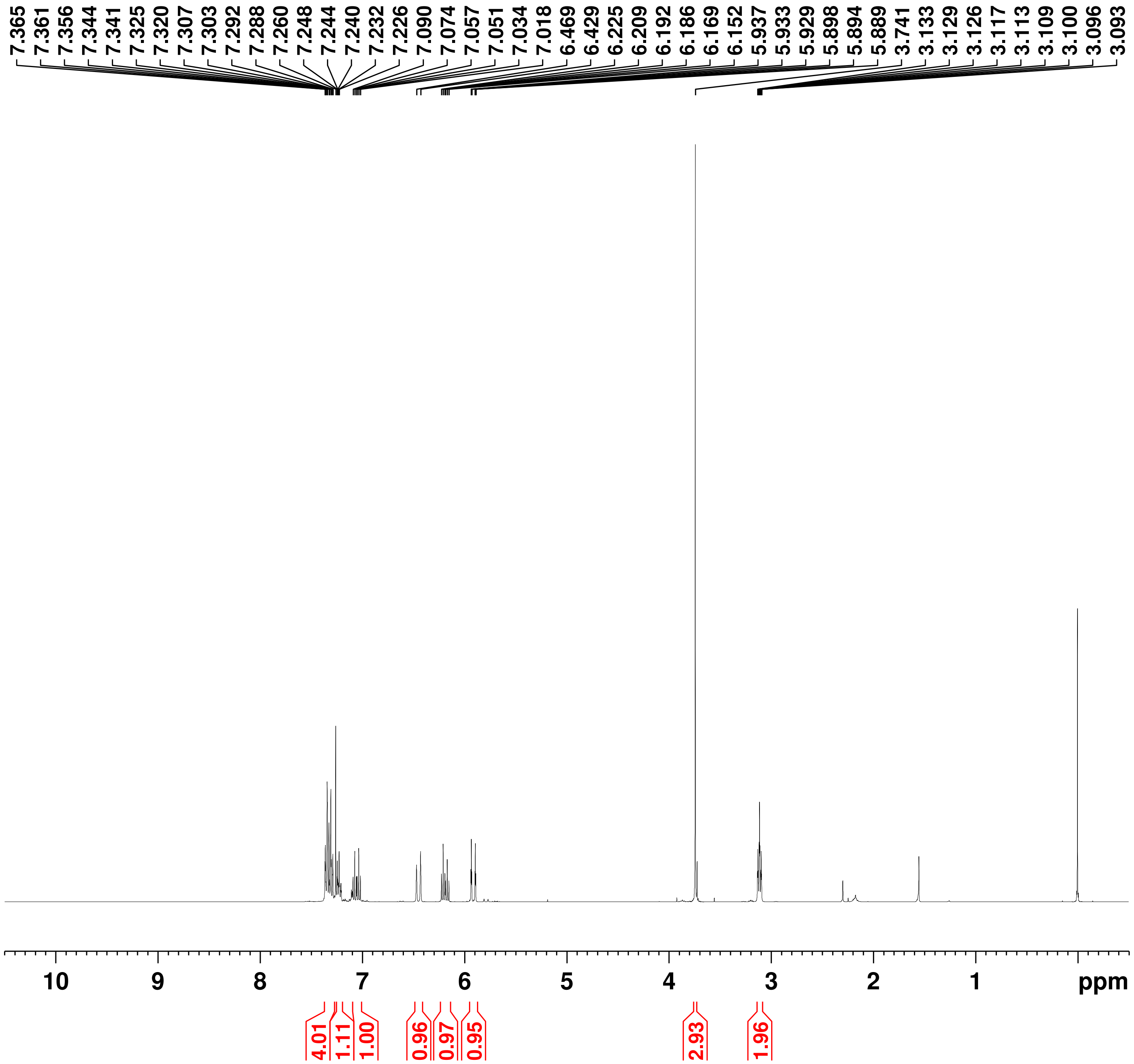

^1H spectrum of compound **8f**

100 MHz, $CHCl_3$

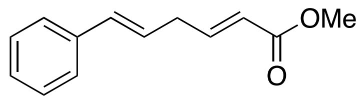

8f

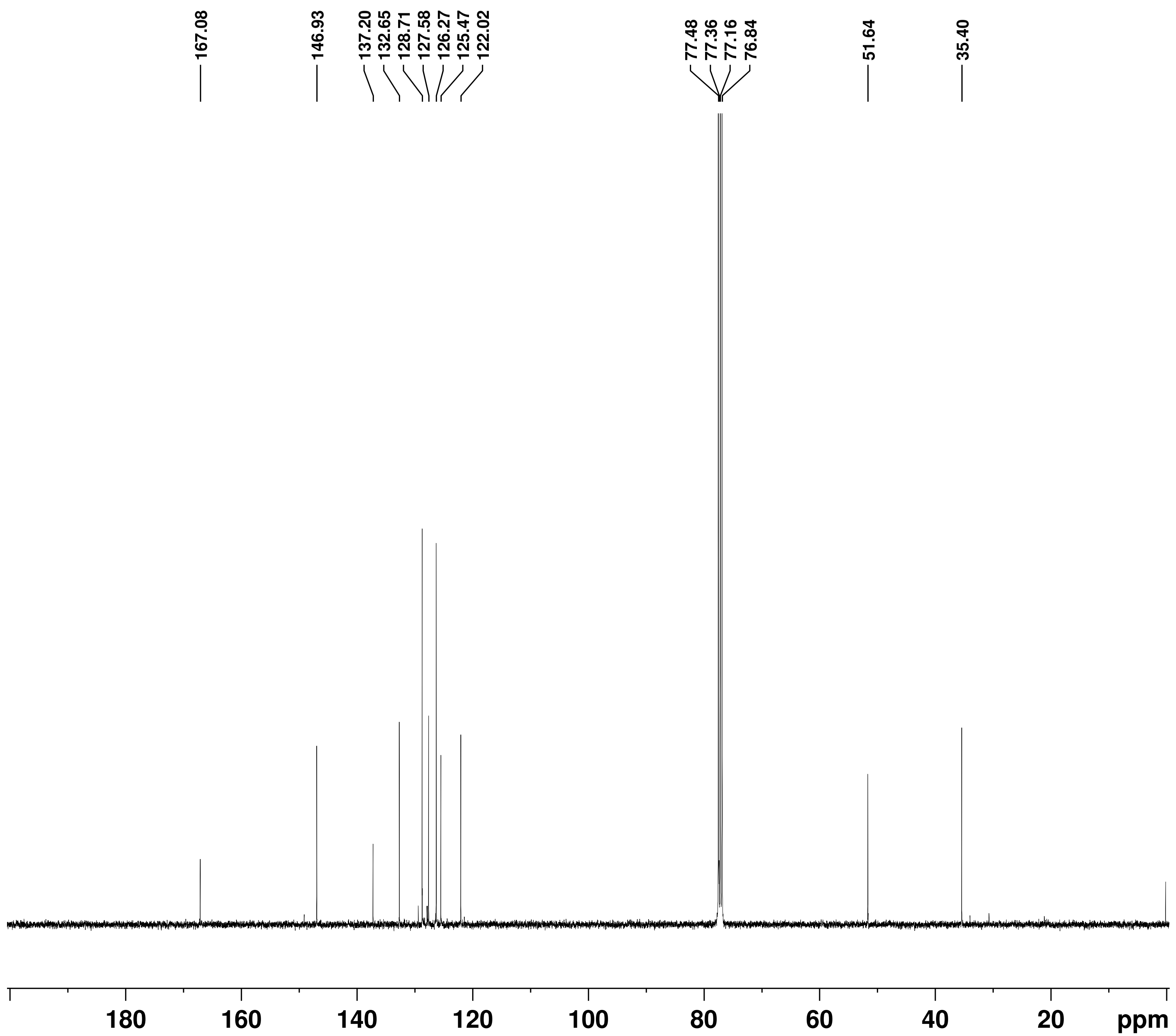

^{13}C spectrum of compound **8f**

400 MHz, $CHCl_3$

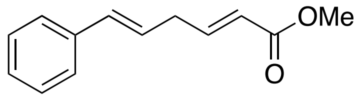

8f

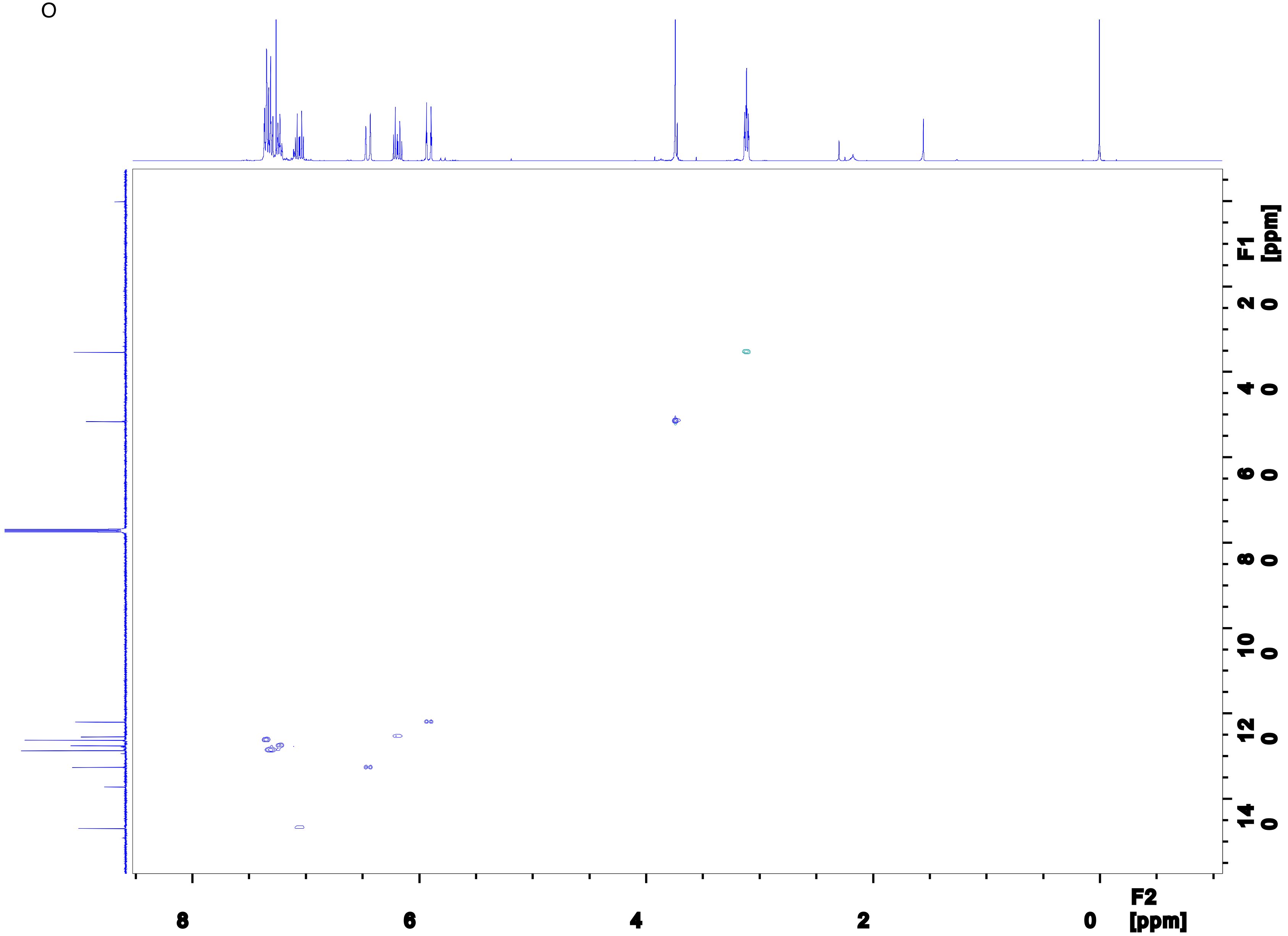

$^{1}H/^{13}C$ HSQC spectrum of compound **8f**

100 MHz, $CHCl_3$

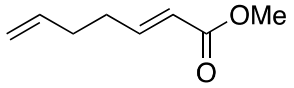

trans-**8g**

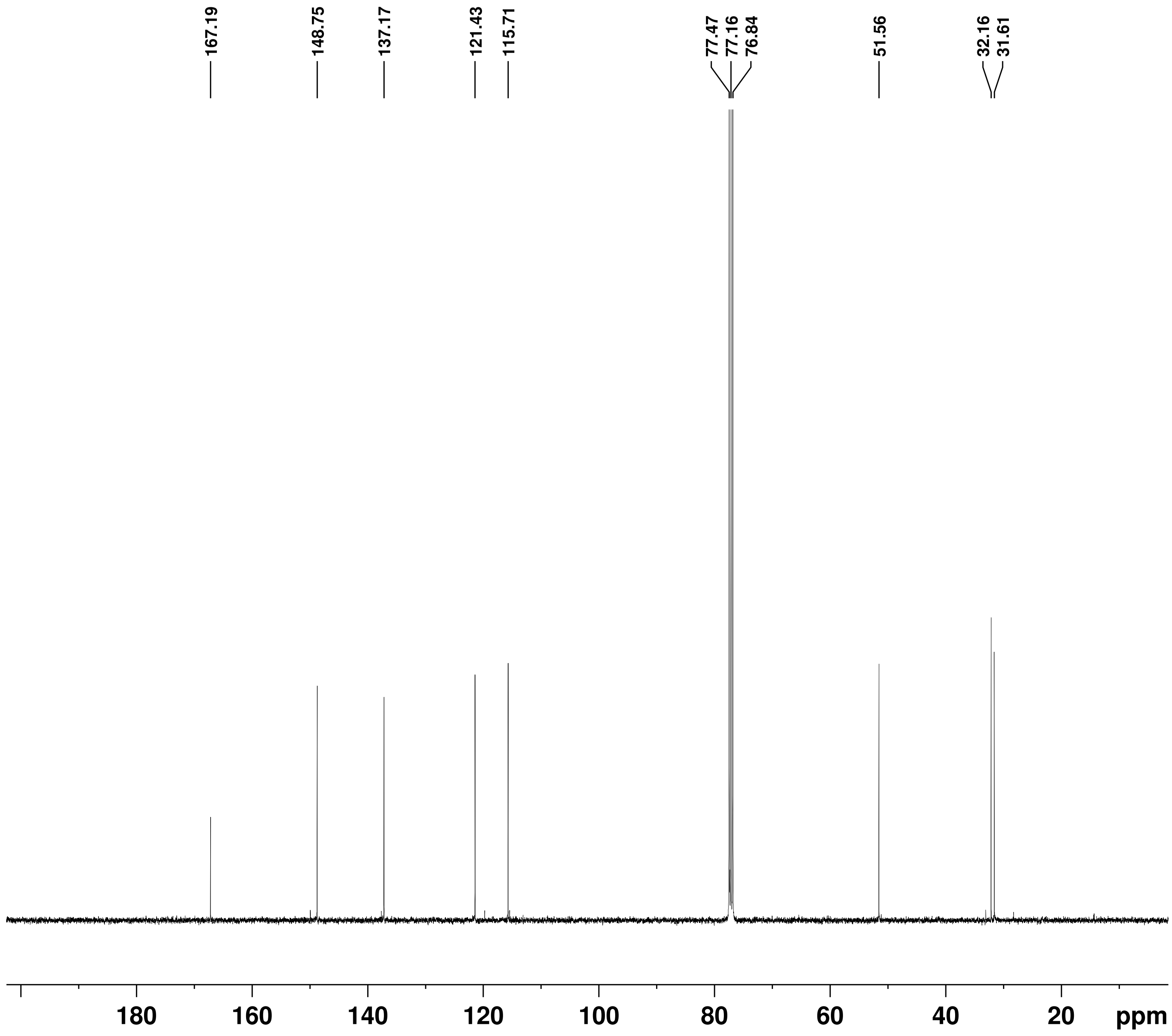

^{13}C spectrum of compound *trans*-**8g**

100 MHz, $CHCl_3$

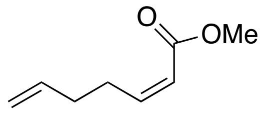

cis-**8g**

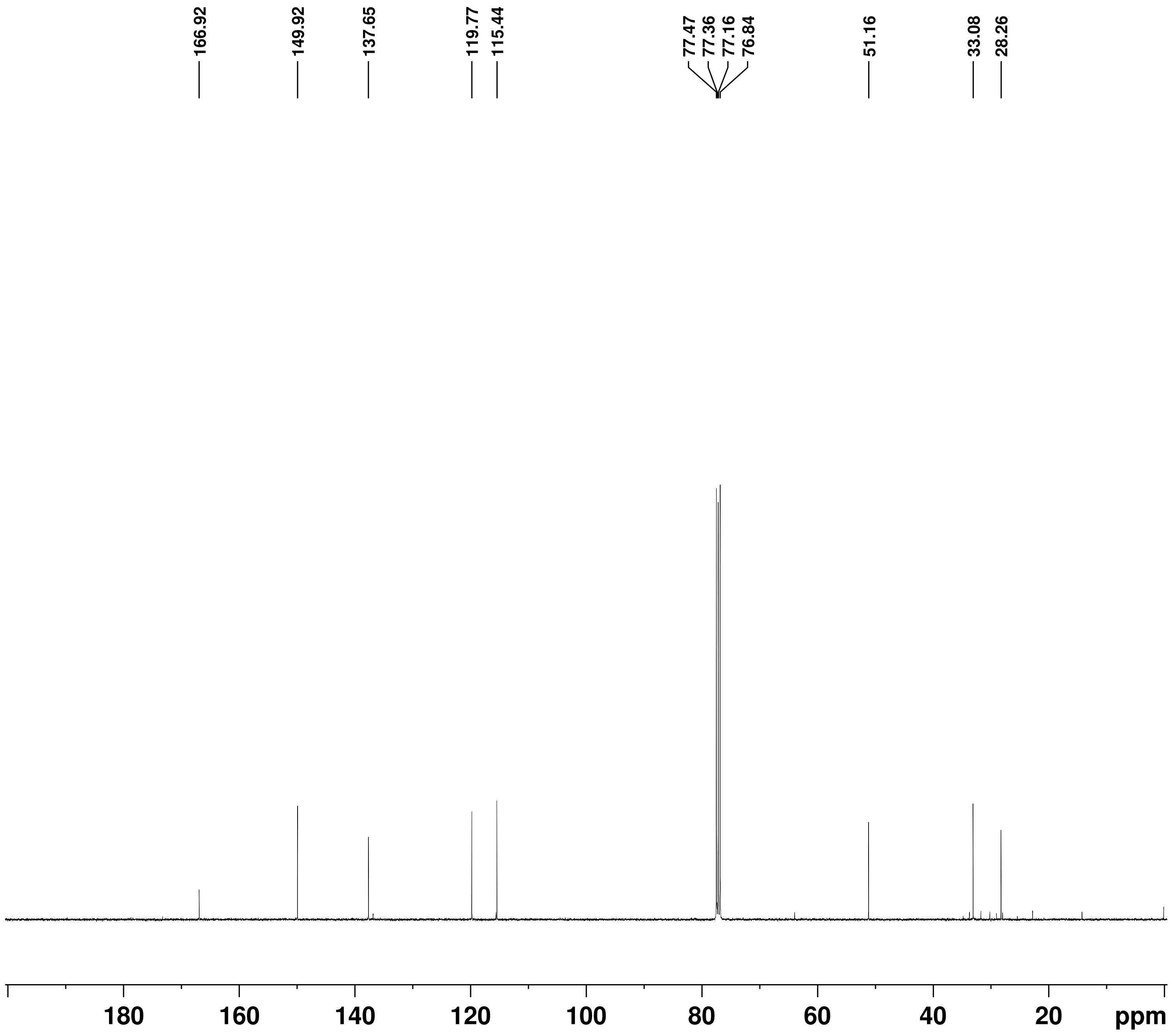

^{13}C spectrum of compound *cis*-**8g**

400 MHz, $CHCl_3$

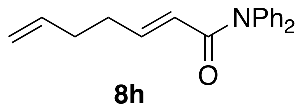

8h

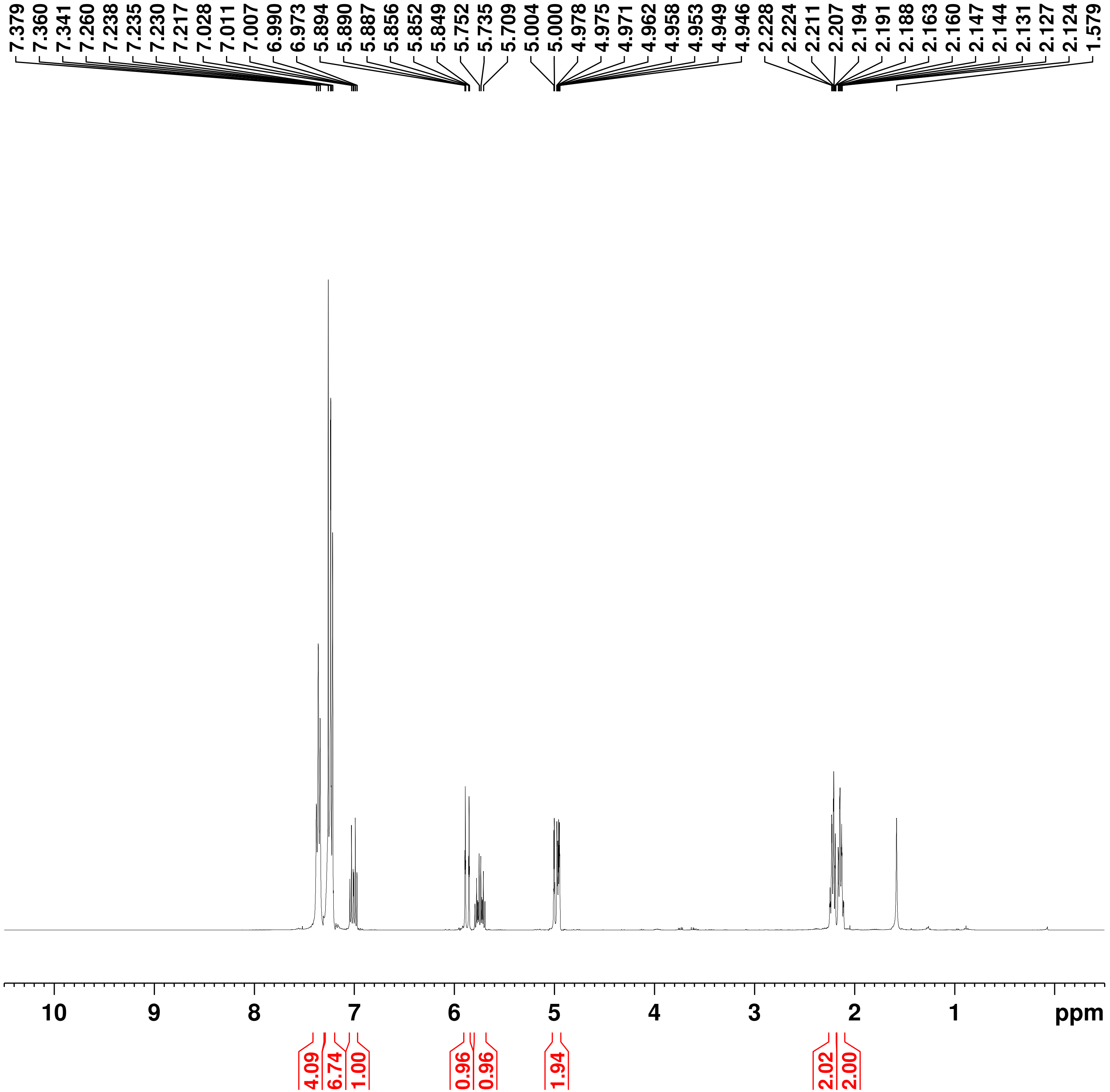

^1H spectrum of compound **8h**

100 MHz, $CHCl_3$

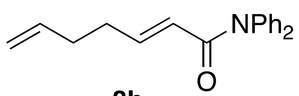

8h

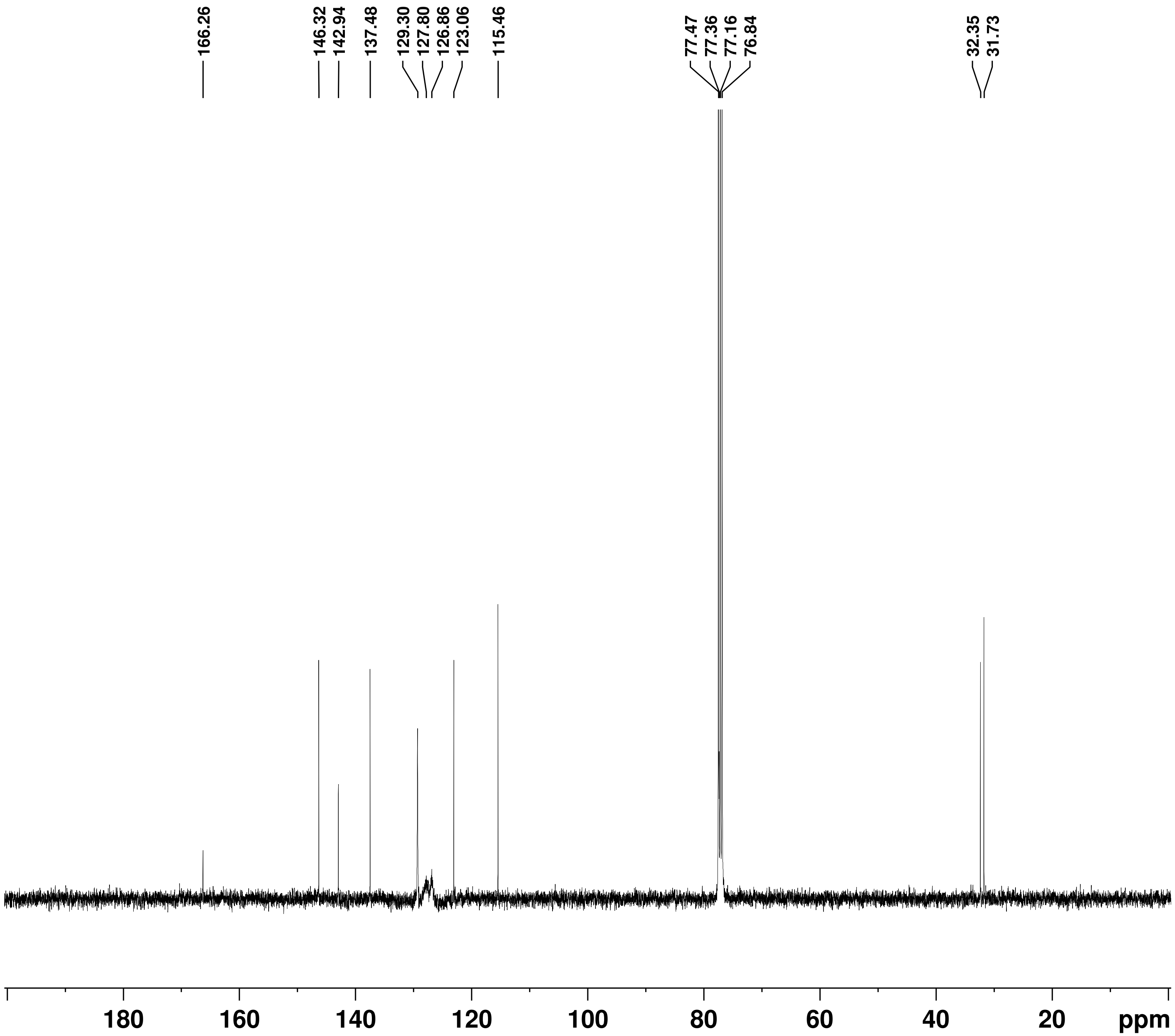

^{13}C spectrum of compound **8h**

400 MHz, $CHCl_3$

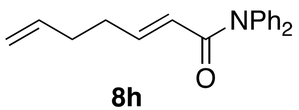

8h

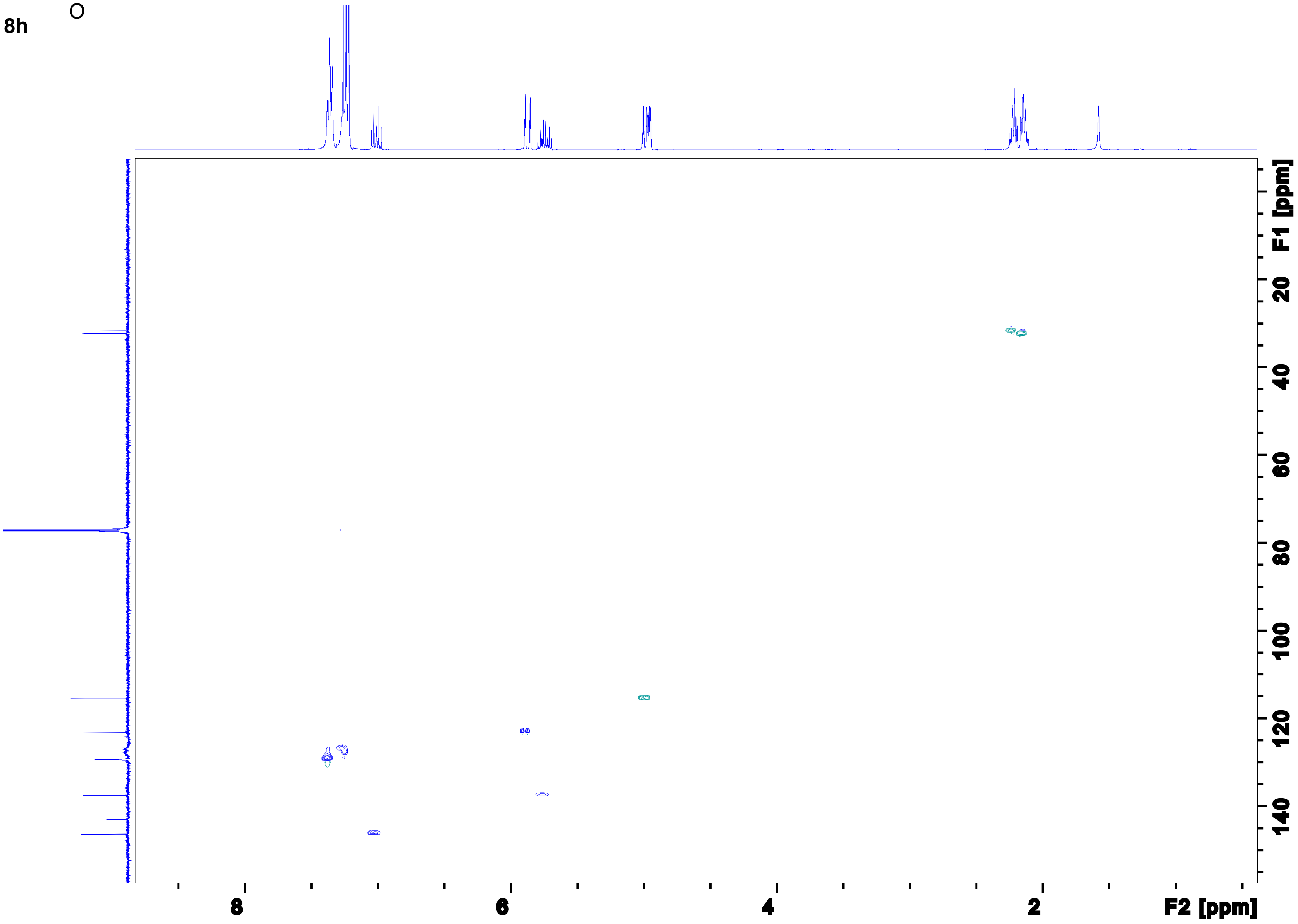

$^{1}H/^{13}C$ HSQC spectrum of compound **8h**

100 MHz, $CHCl_3$

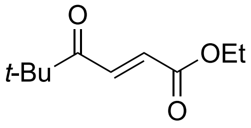

S22

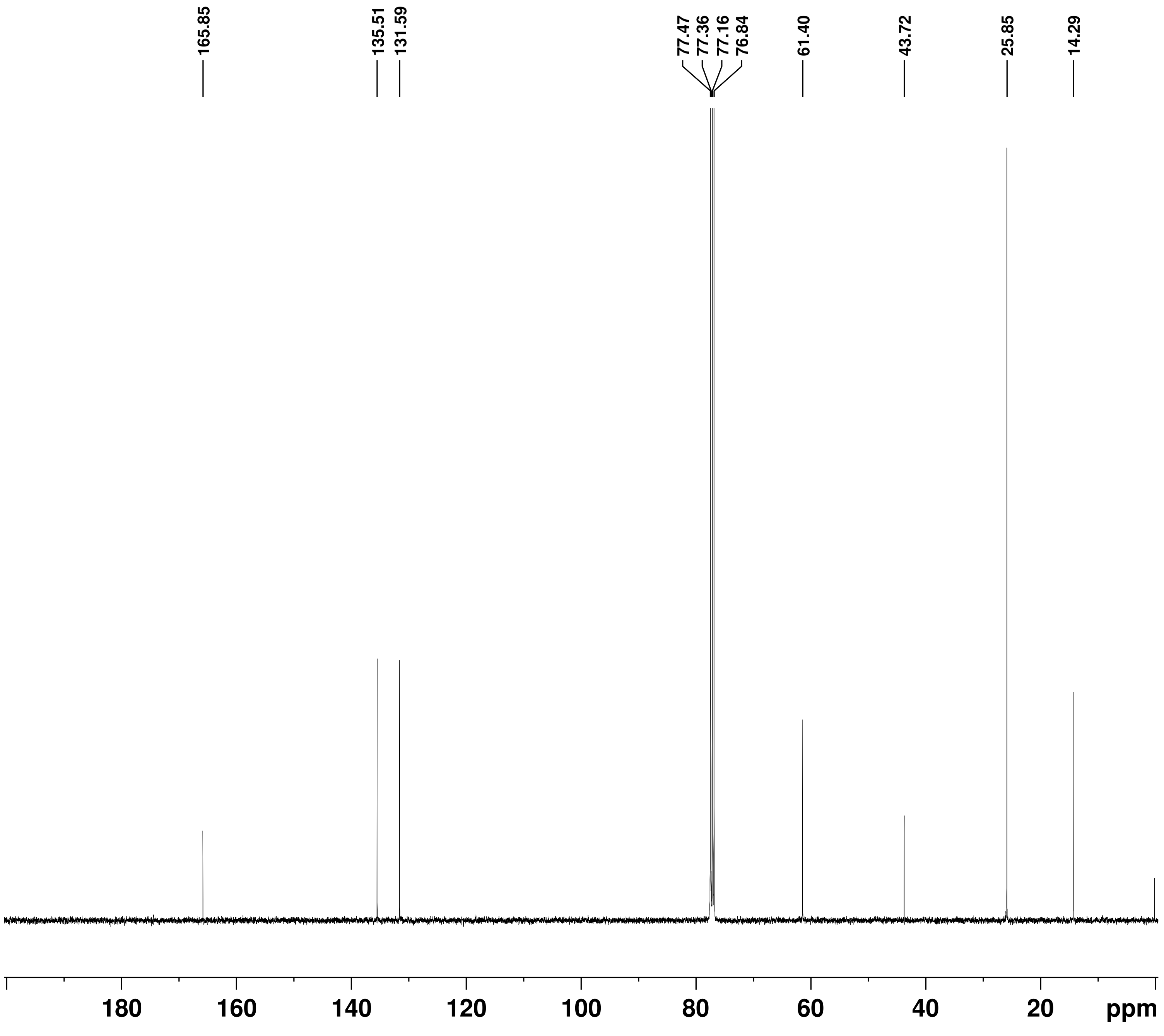

^{13}C spectrum of compound **S22**

100 MHz, $CHCl_3$

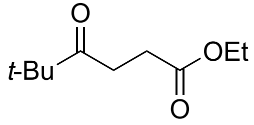

S23

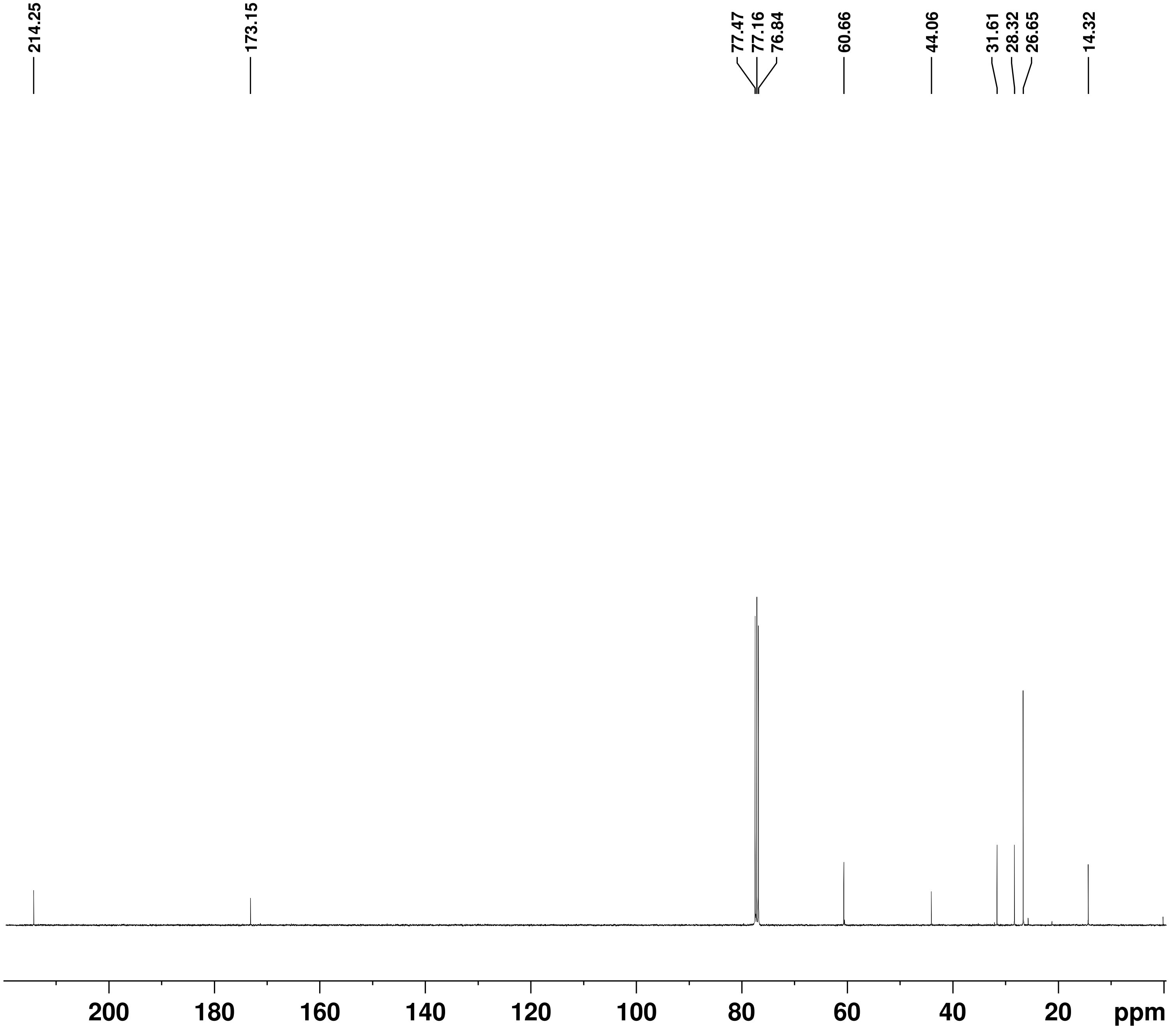

^{13}C spectrum of compound **S23**

100 MHz, $CHCl_3$

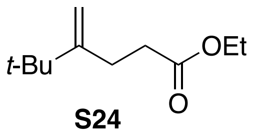

S24

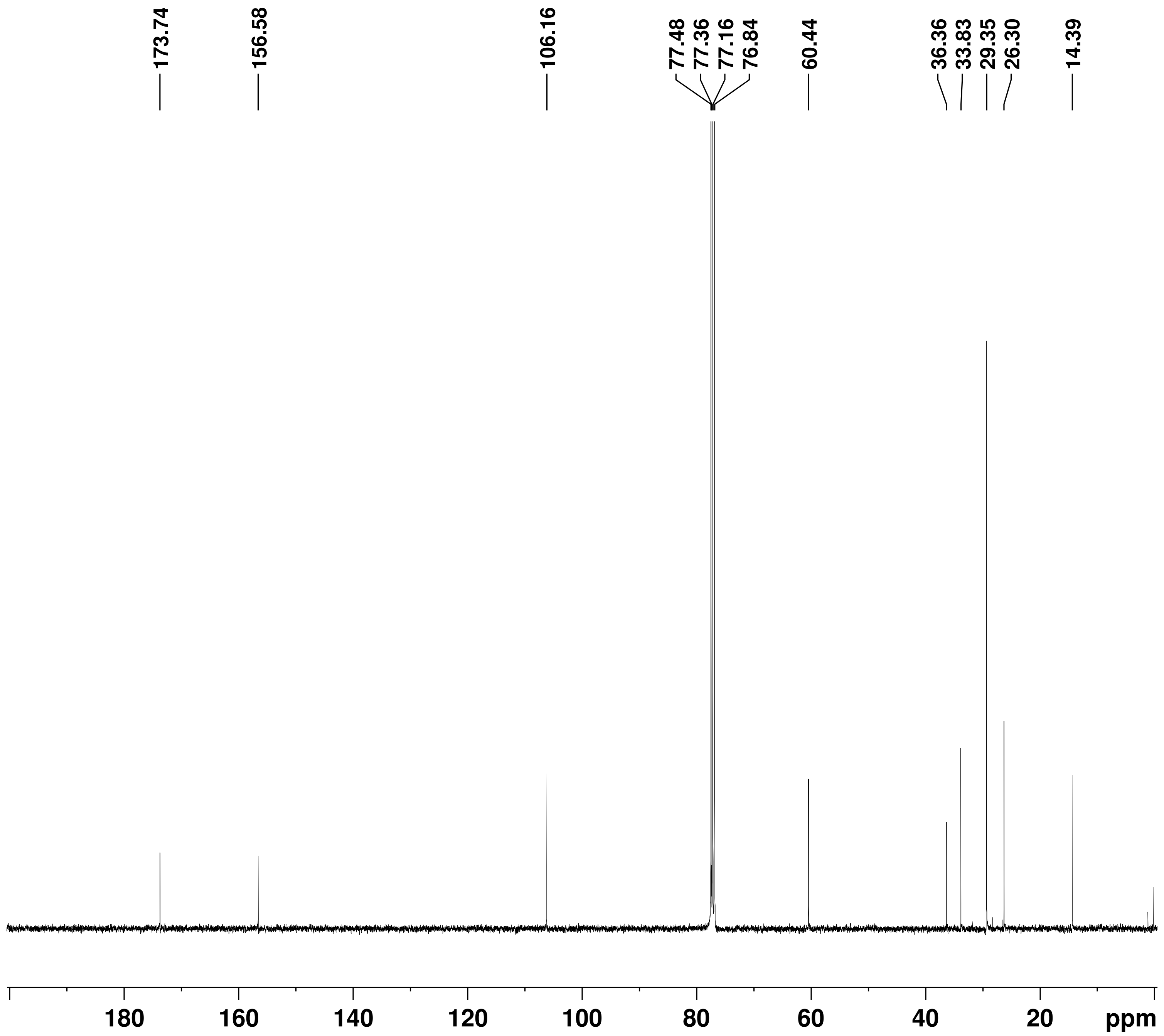

^{13}C spectrum of compound **S24**

400 MHz, $CDCl_3$

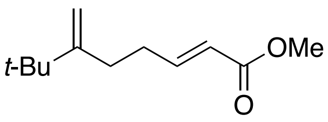

8i

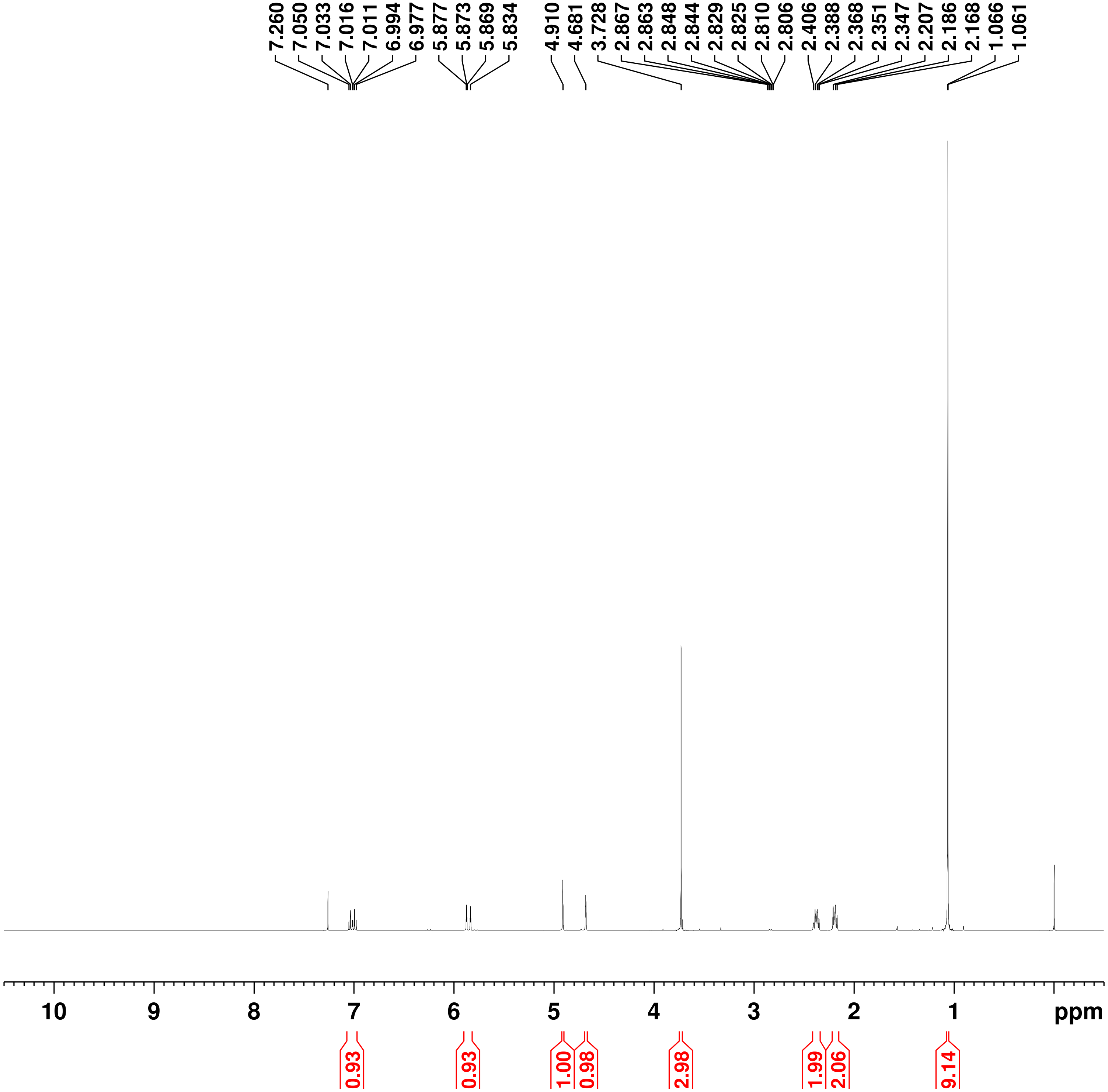

^{1}H spectrum of compound **8i**

100 MHz, $CDCl_3$

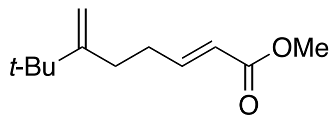

8i

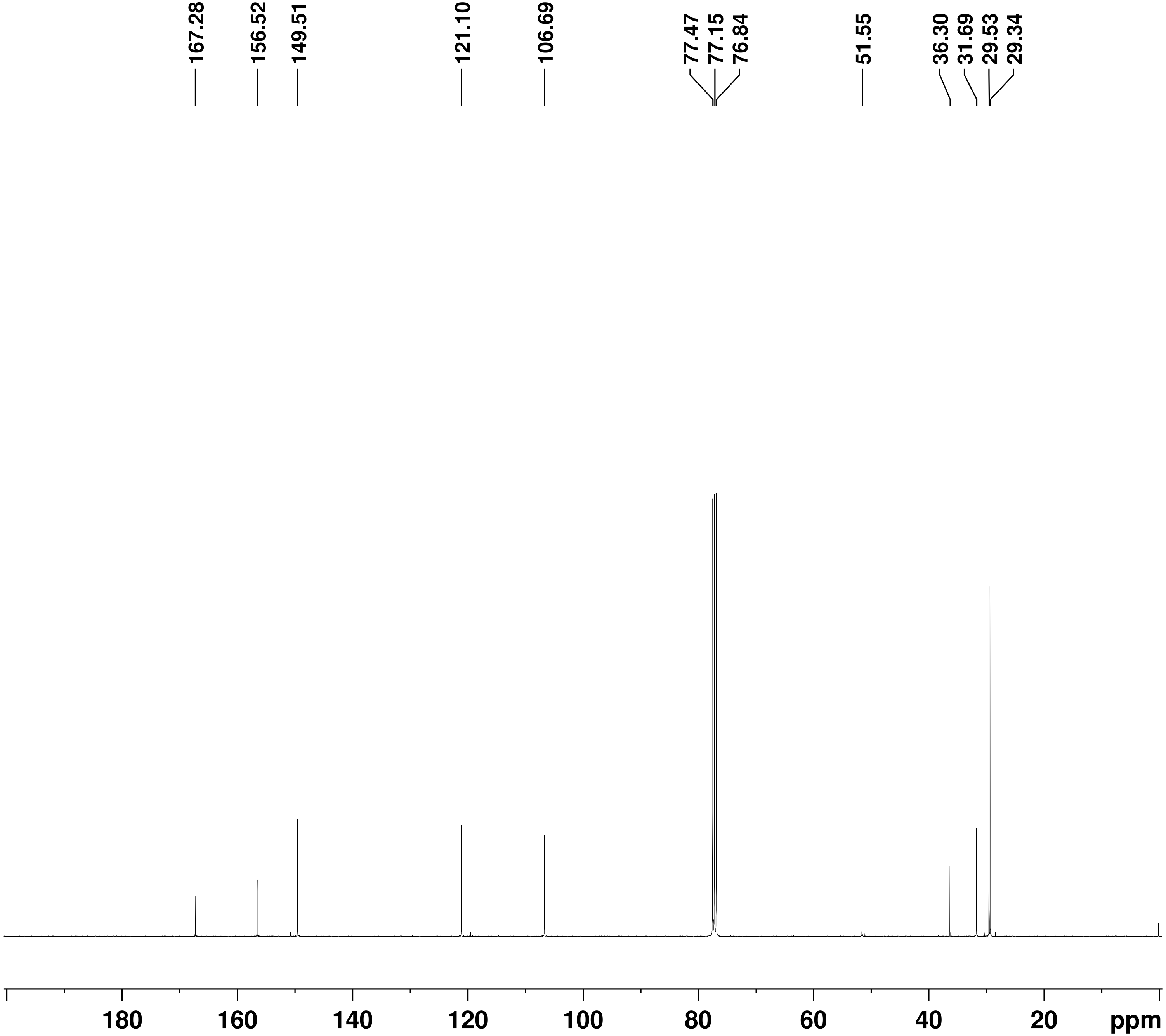

^{13}C spectrum of compound **8i**

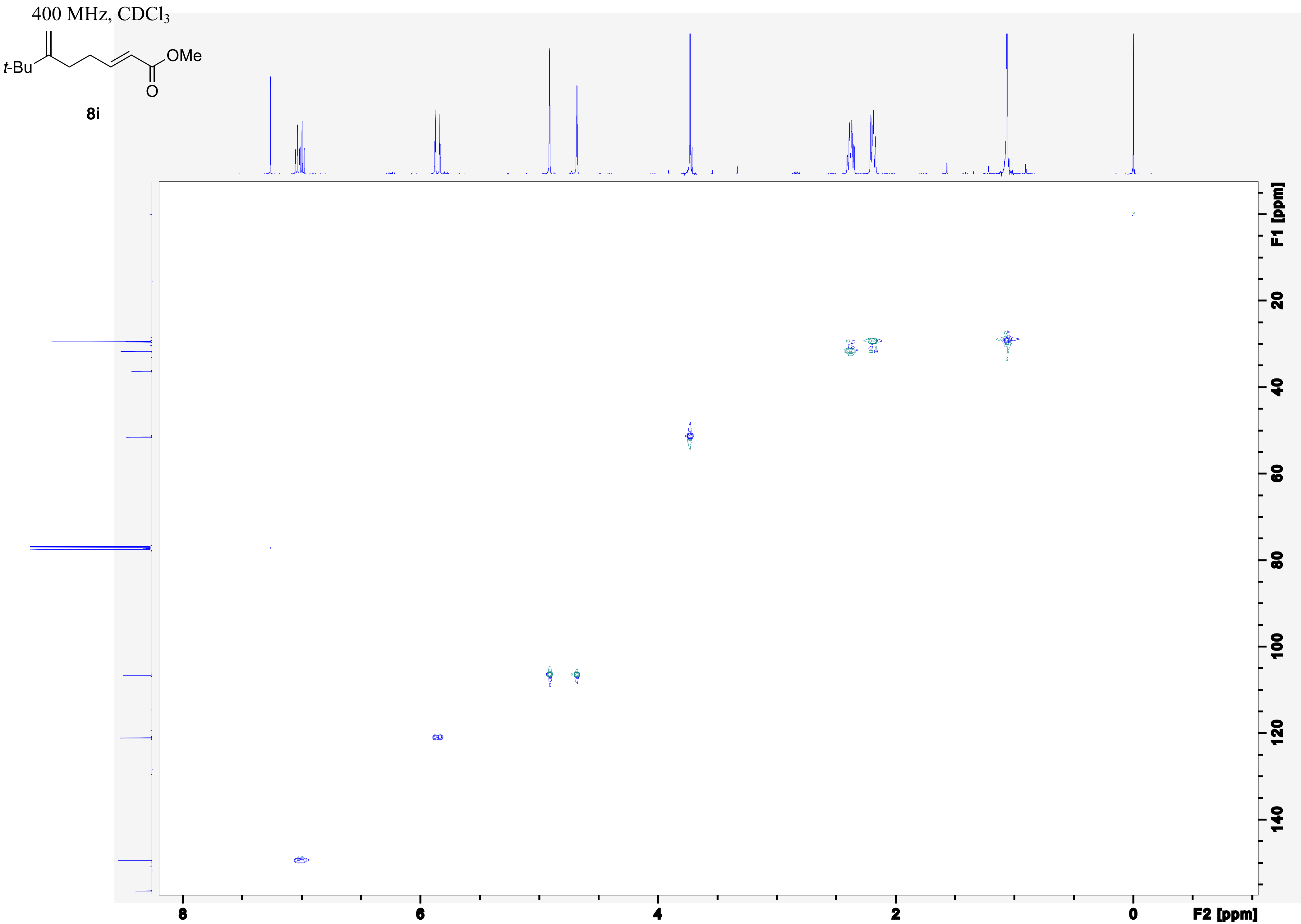

$^{1}H/^{13}C$ HSQC spectrum of compound **8i**

400 MHz, $CHCl_3$

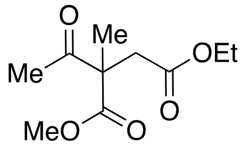

S27

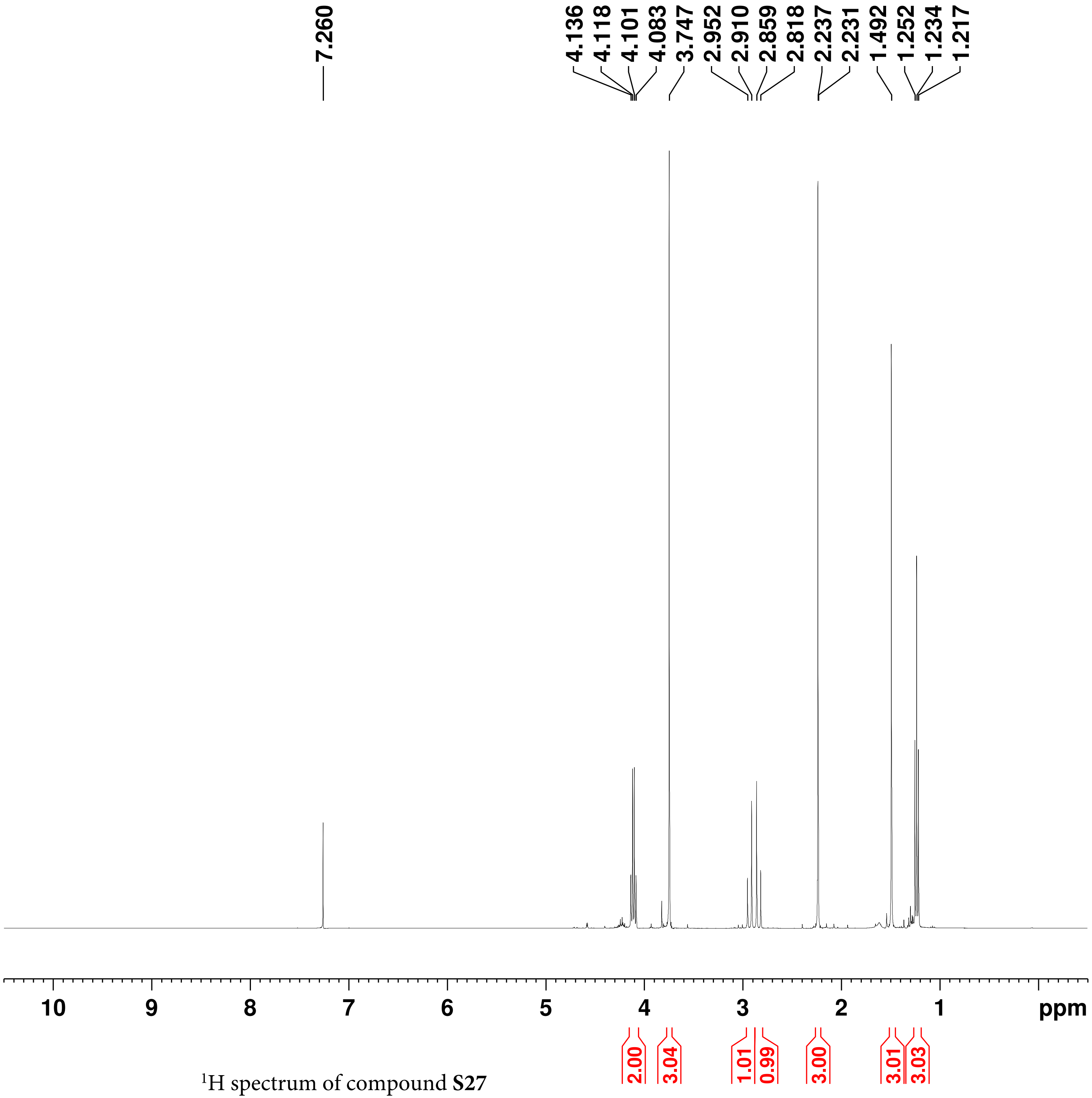

^{1}H spectrum of compound **S27**

100 MHz, $CHCl_3$

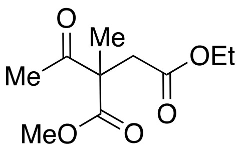

S27

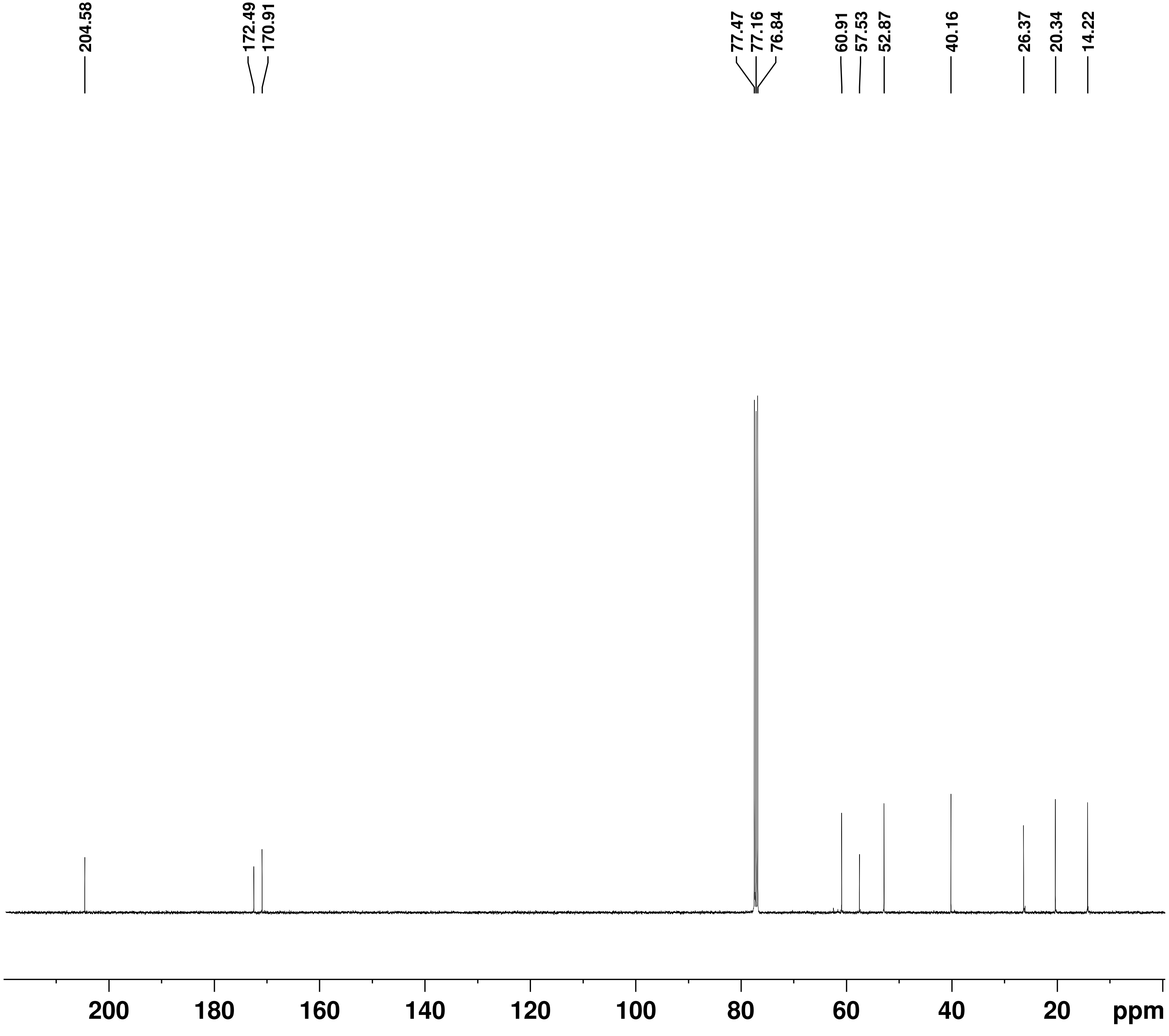

^{13}C spectrum of compound **S27**

400 MHz, $CHCl_3$

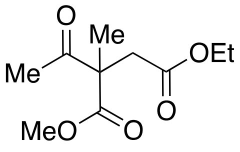

S27

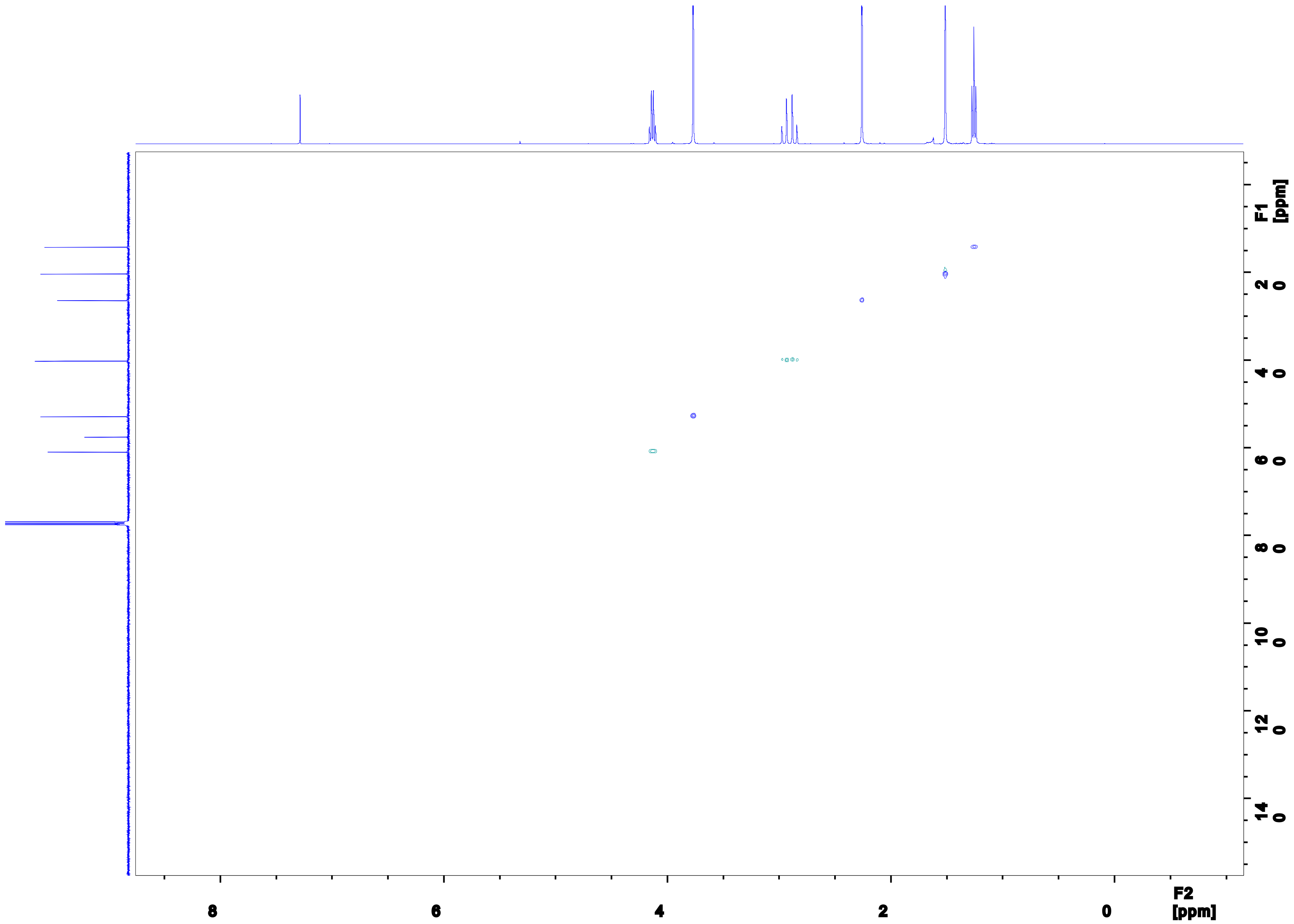

$^1H/^{13}C$ HSQC spectrum of compound **S2**

100 MHz, $CHCl_3$

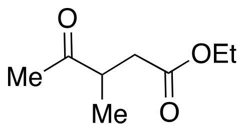

S28

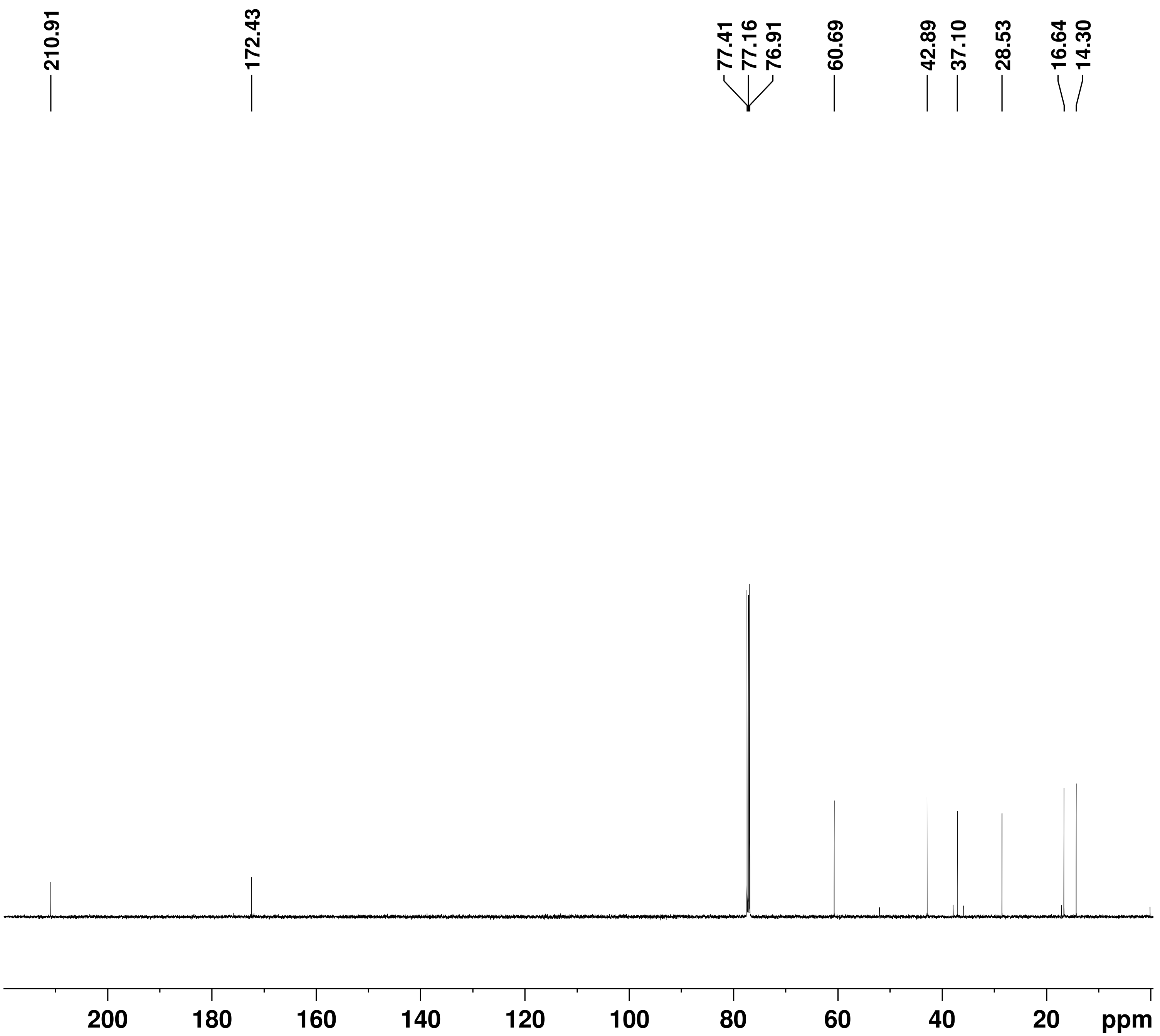

^{13}C spectrum of compound **S28**

100 MHz, $CHCl_3$

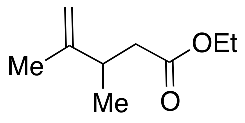

S29

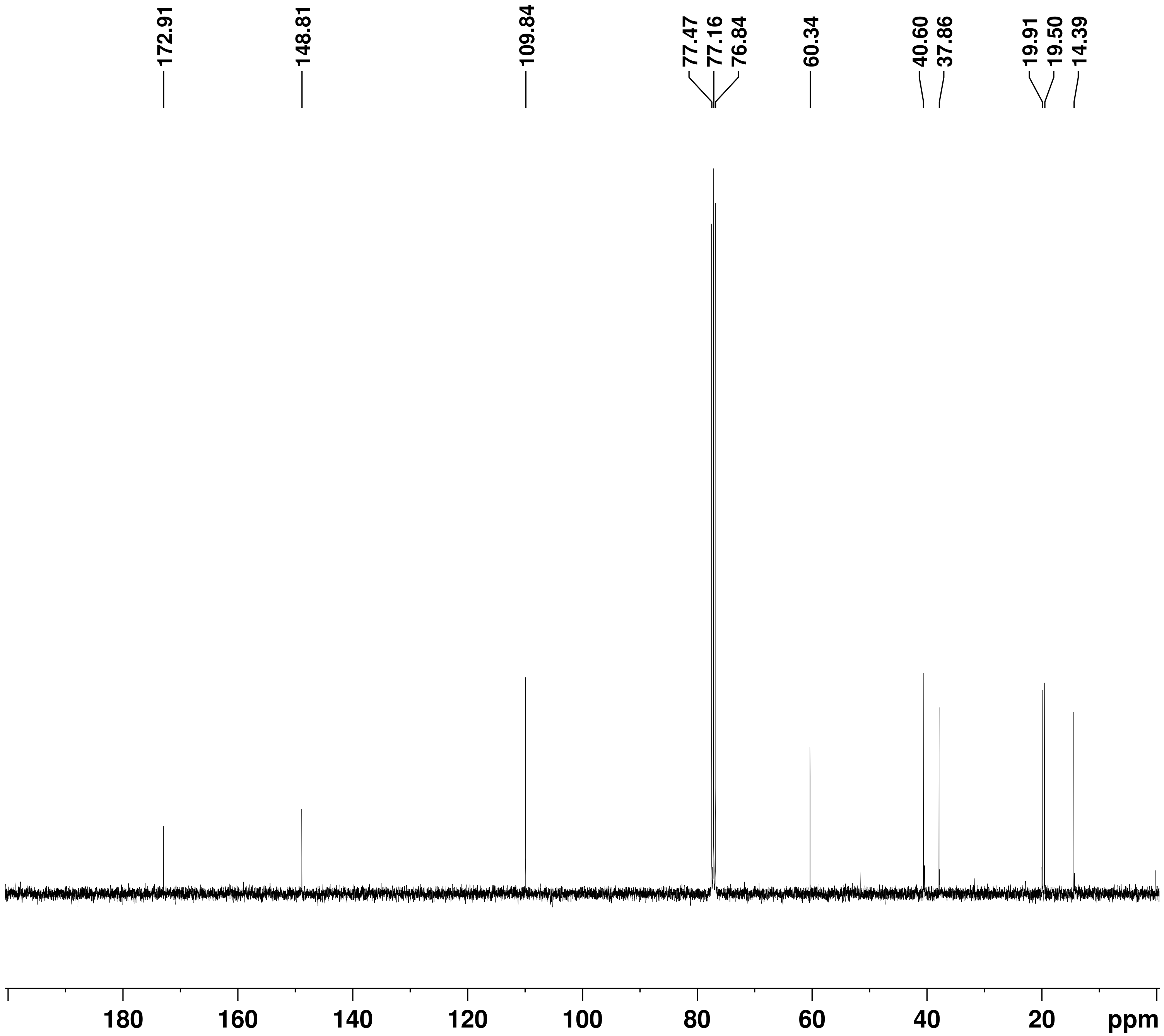

^{13}C spectrum of compound 2S299

400 MHz, $CHCl_3$

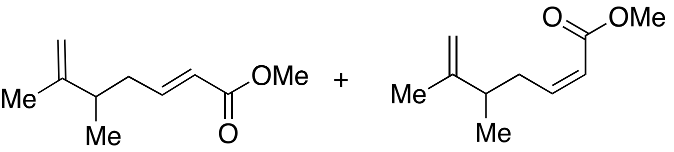

8ja **8jb**

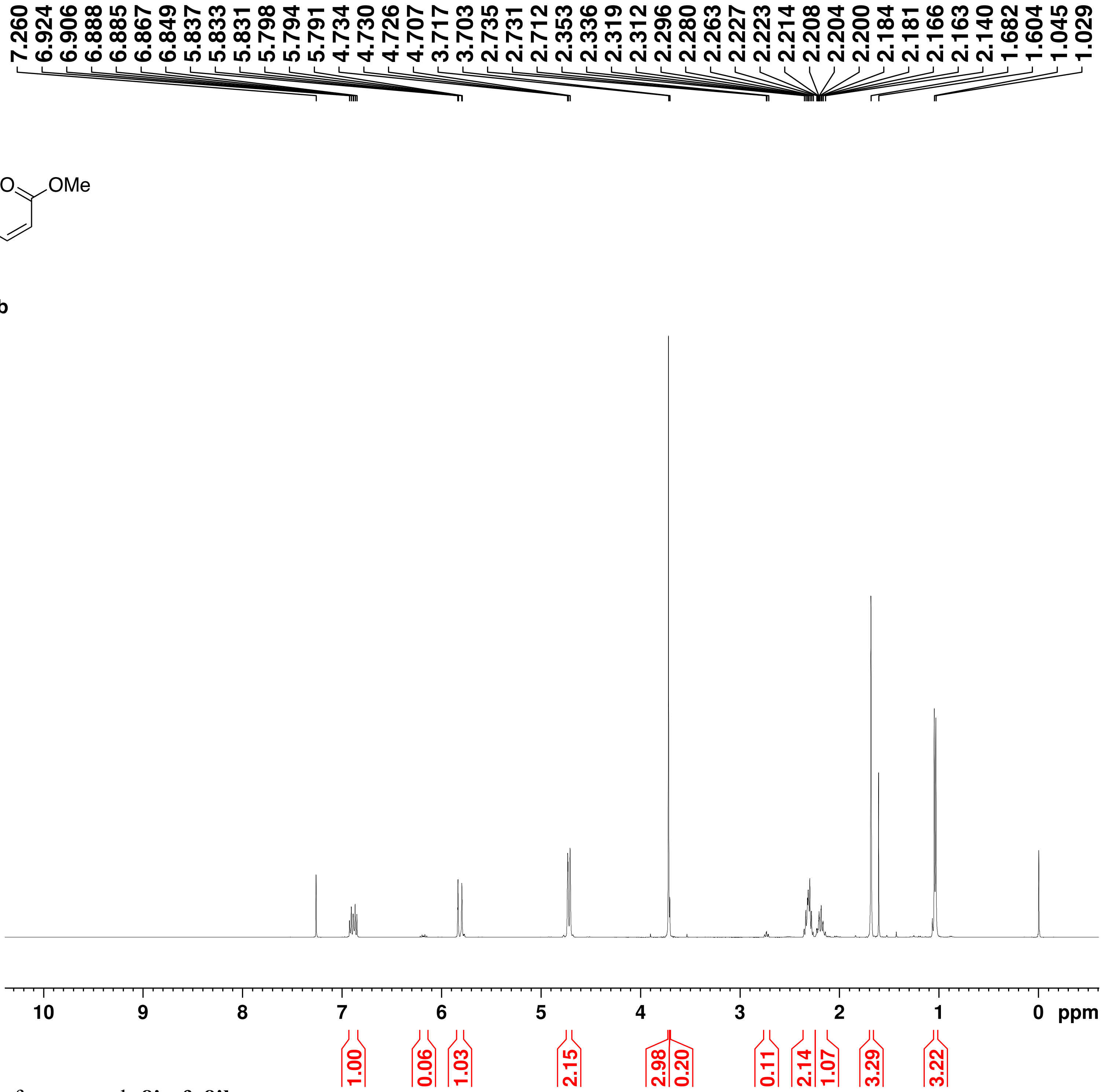

^{13}C spectrum of compounds **8ja & 8jb**

100 MHz, $CHCl_3$

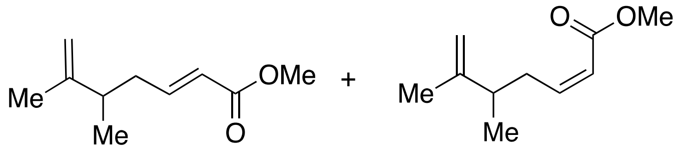

8ja **8jb**

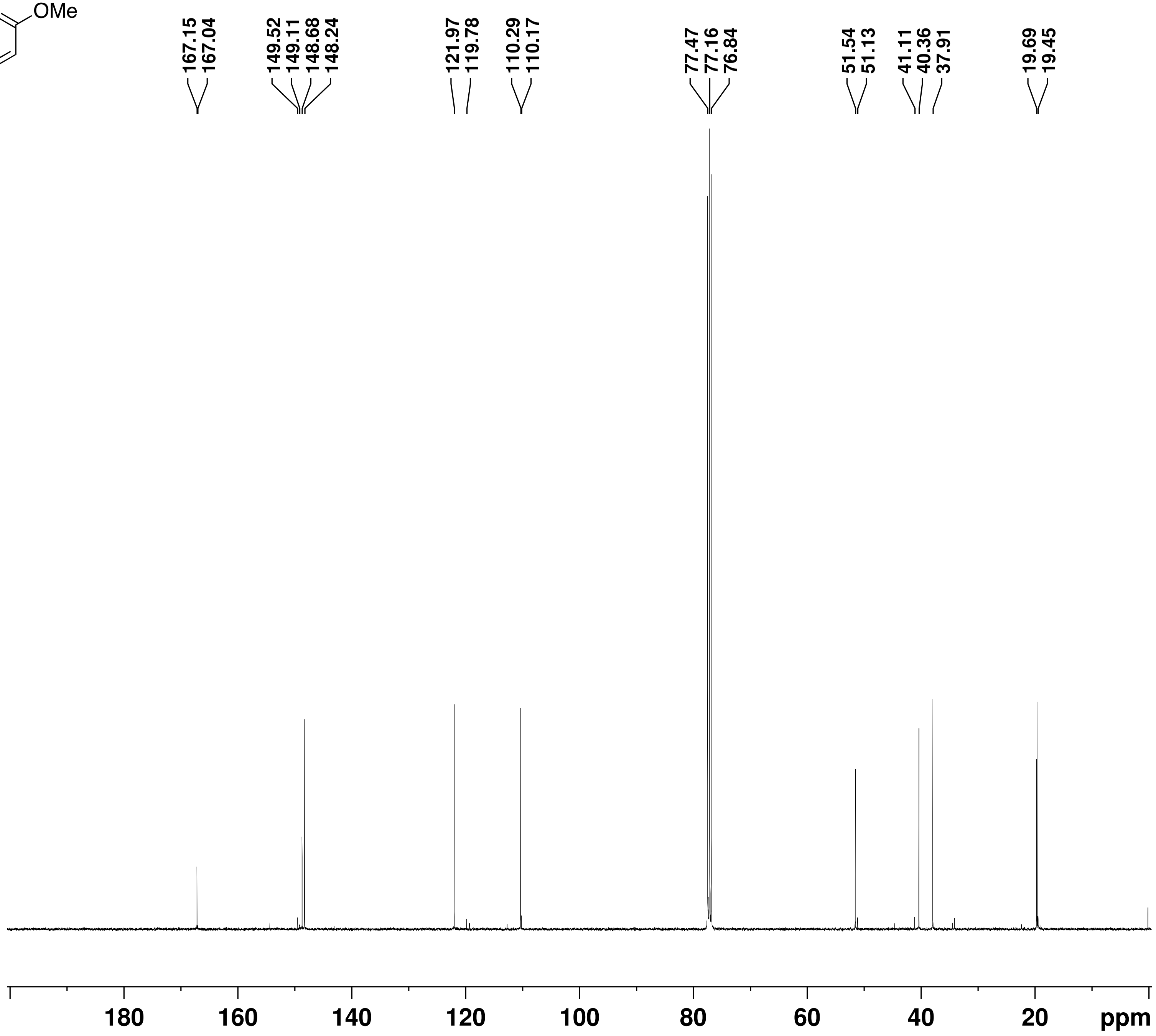

^{13}C spectrum of compounds **8ja & 8jb**

400 MHz, $CHCl_3$

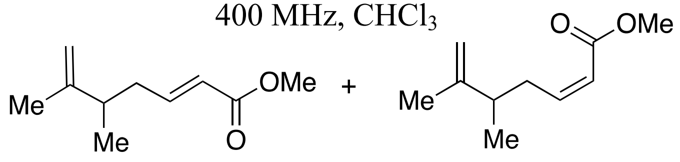

8ja **8jb**

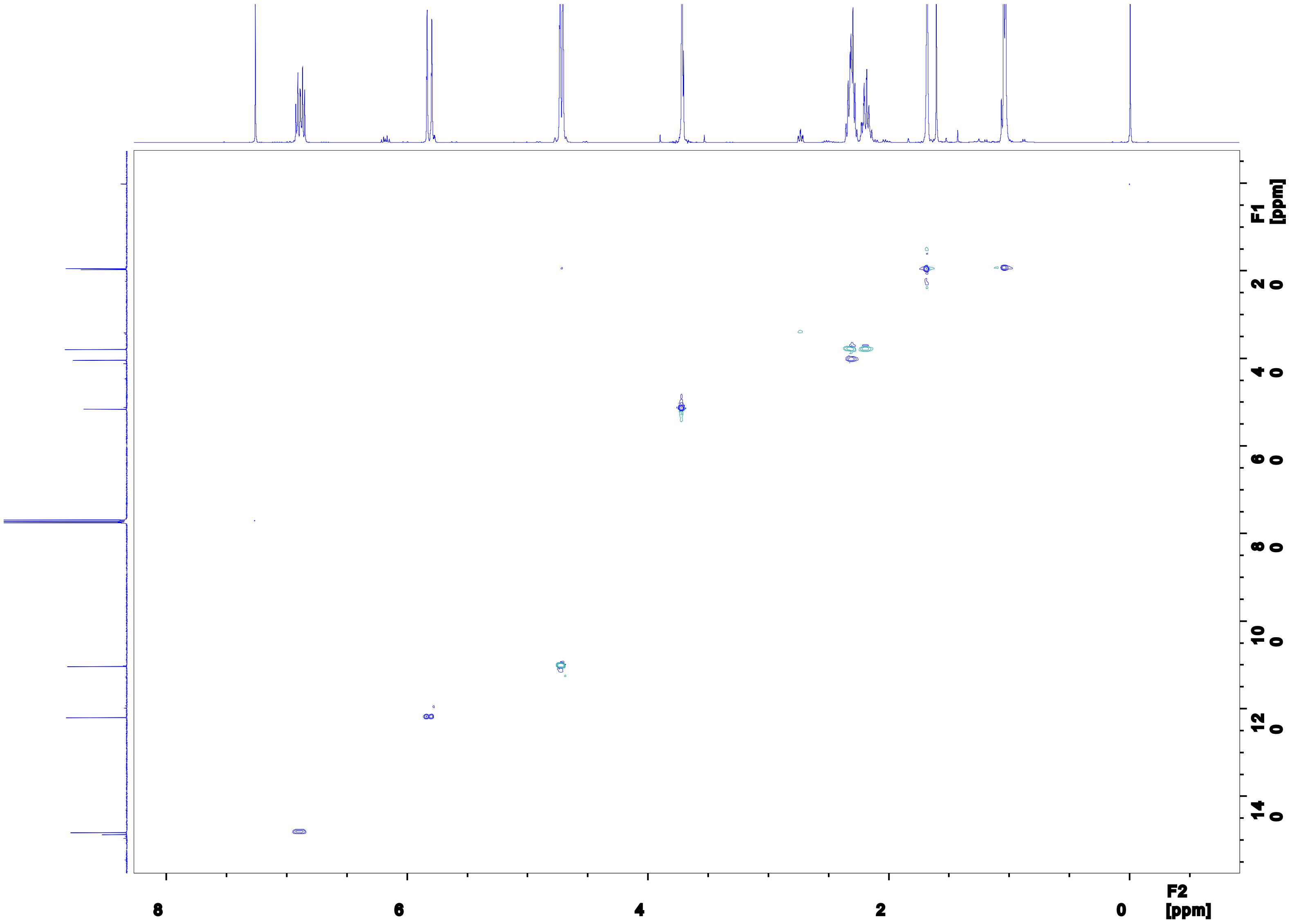

$^{1}H/^{13}C$ HSQC spectrum of compounds **8ja & 8jb**

400 MHz, $CHCl_3$

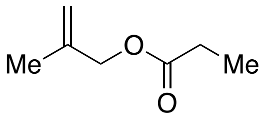

S31

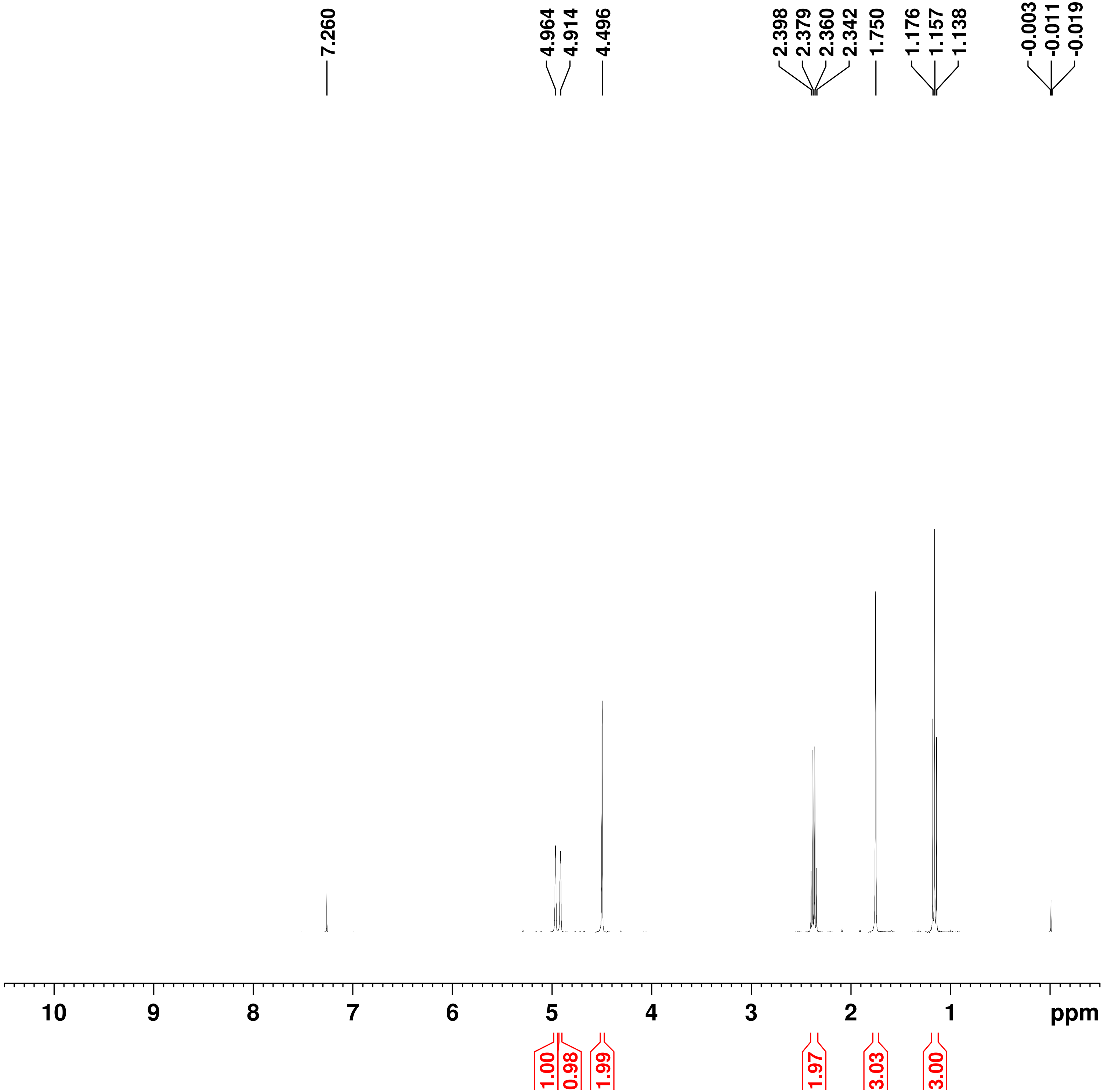

^{1}H spectrum of compound **S3131**

100 MHz, $CHCl_3$

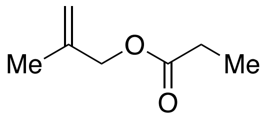

S31

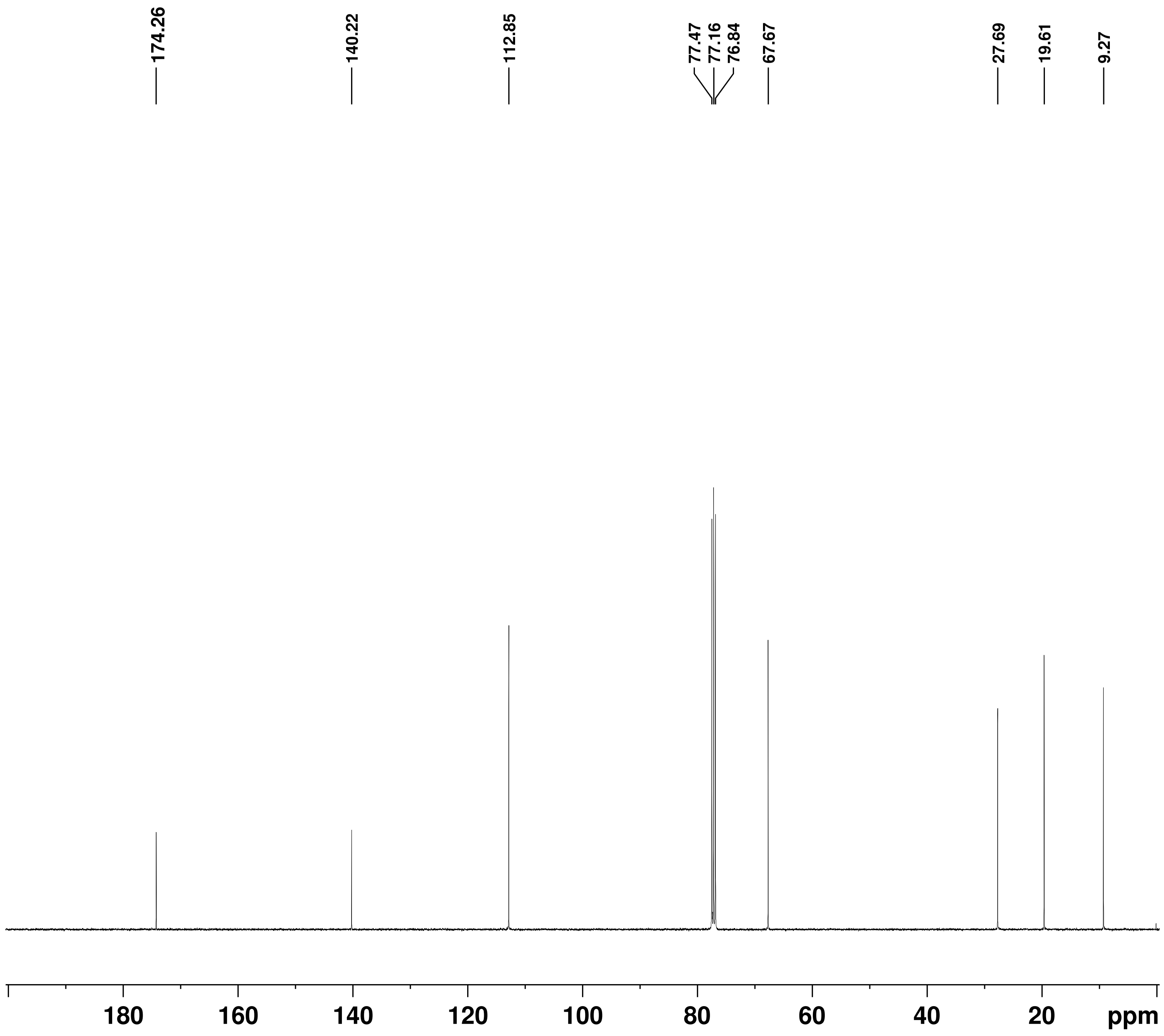

^{13}C spectrum of compound **S3131**

400 MHz, $CHCl_3$

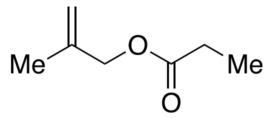

S31

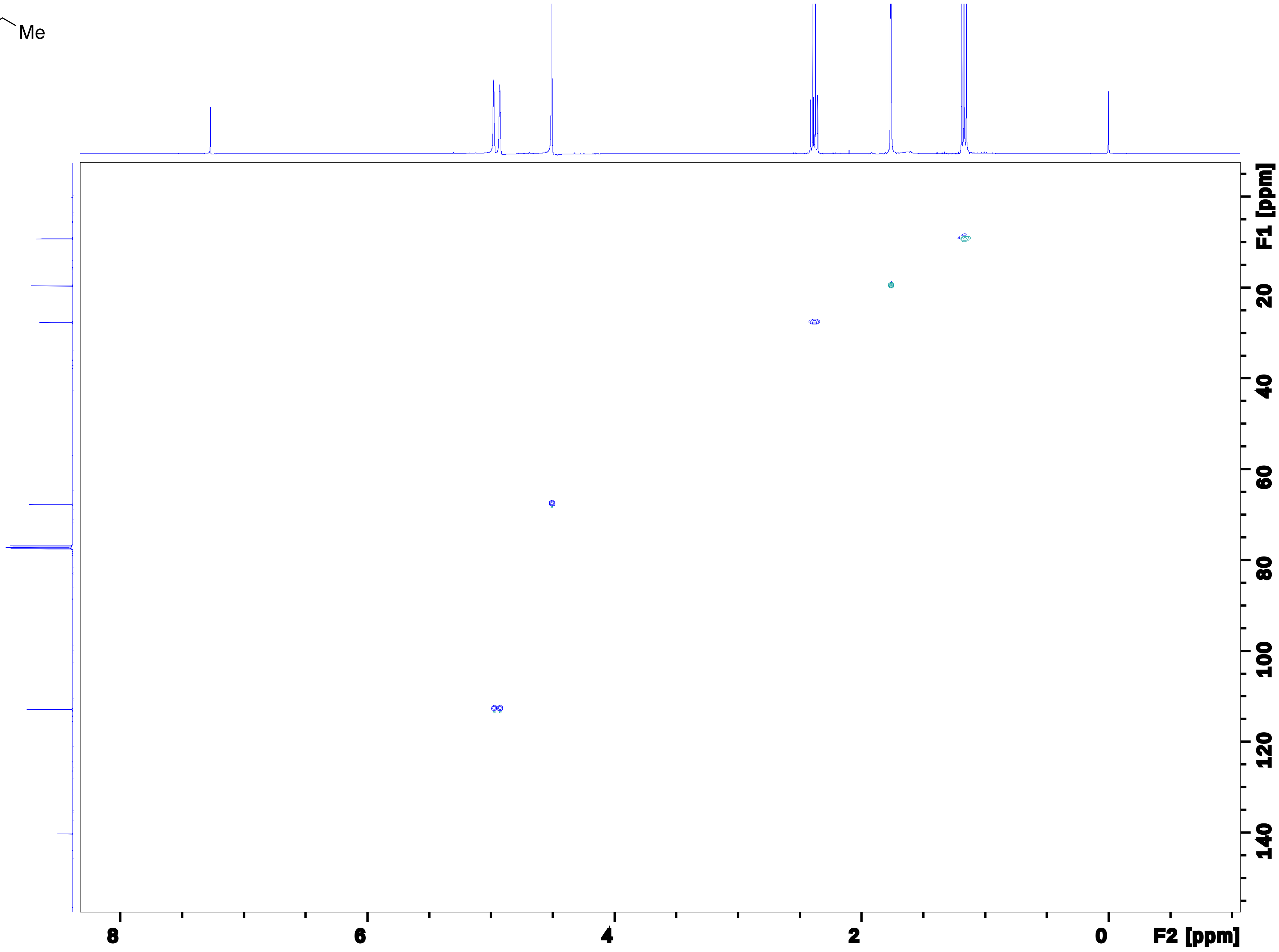

$^{1}H/^{13}C$ HSQC spectrum of compound **S311**

100 MHz, $CHCl_3$

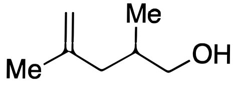

S32

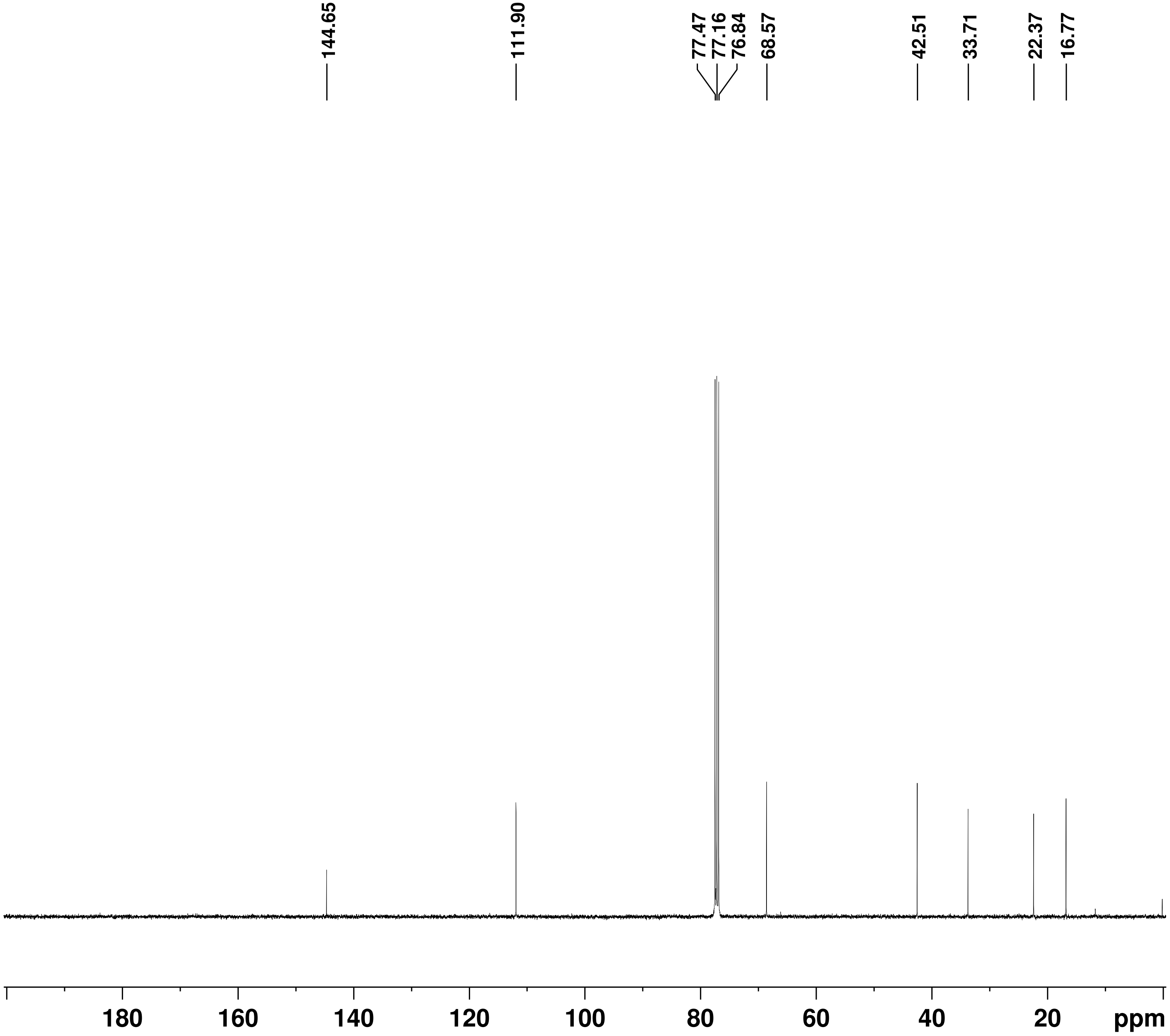

^{13}C spectrum of compound **S32 S**

400 MHz, $CHCl_3$

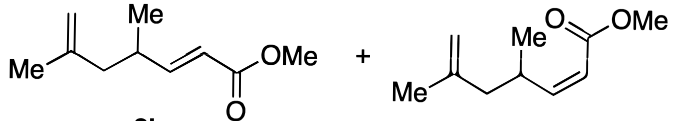

8ka + **8kb**

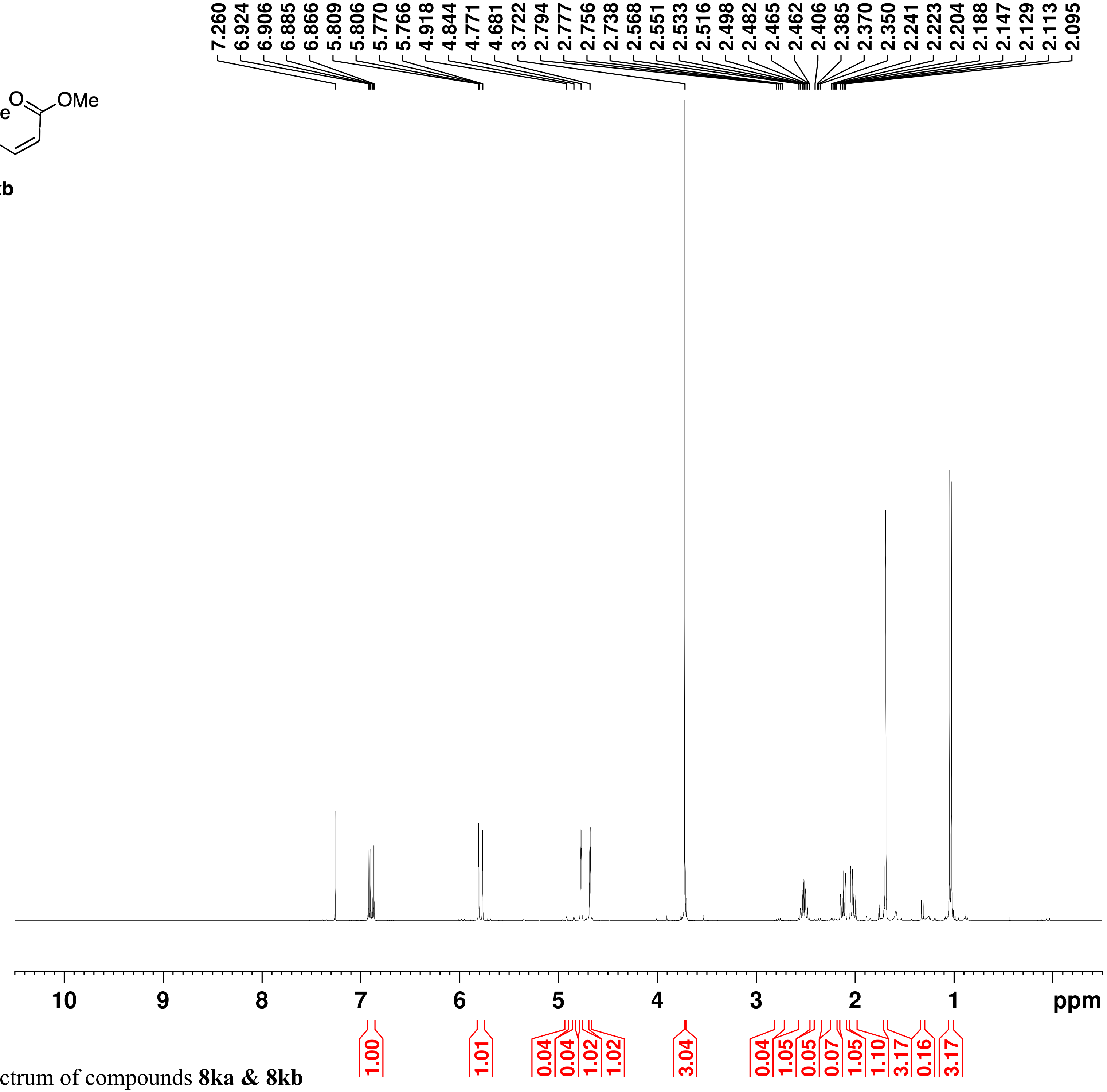

^{1}H spectrum of compounds **8ka & 8kb**

100 MHz, $CHCl_3$

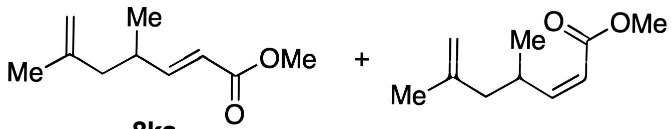

8ka **8kb**

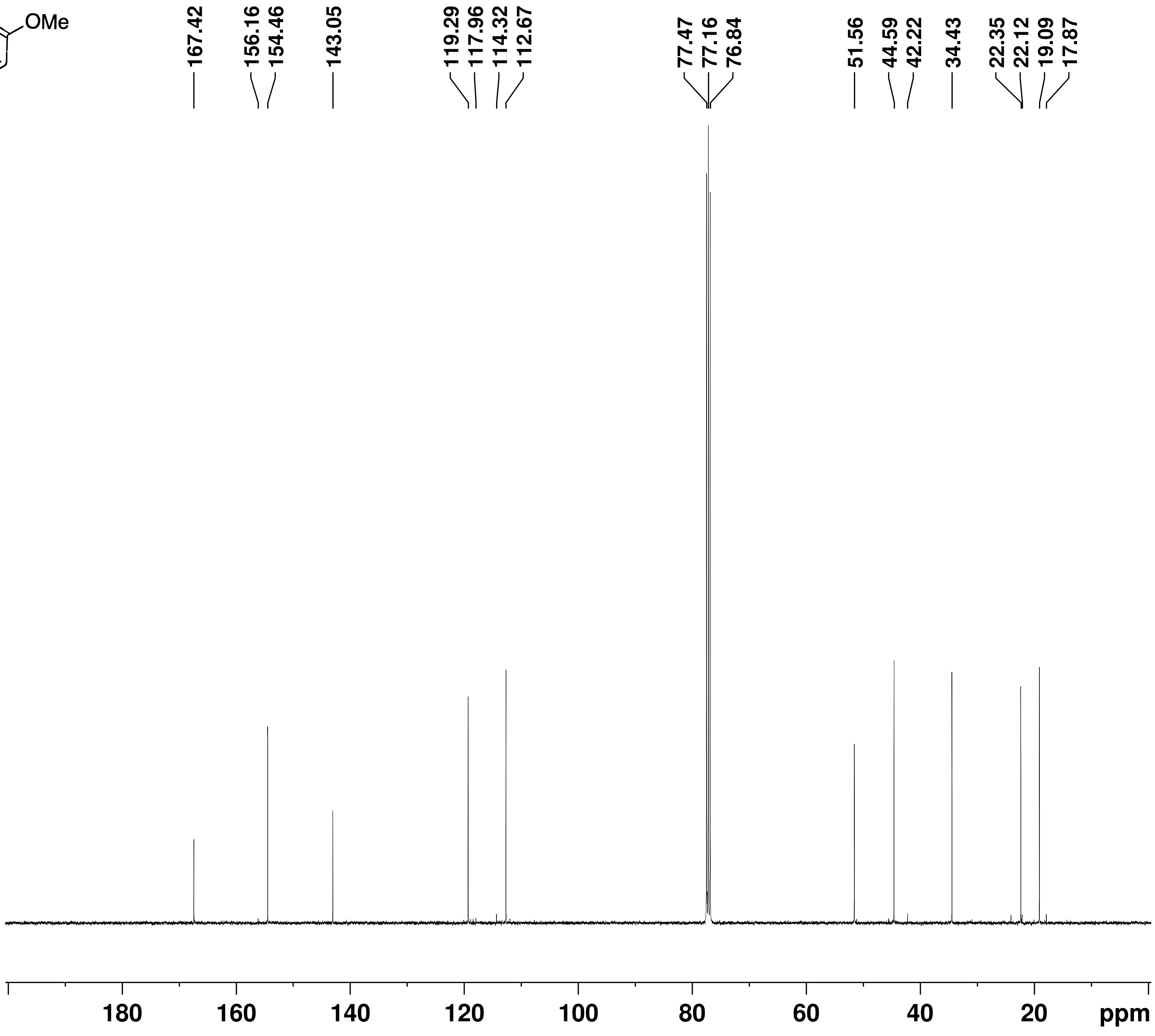

^{13}C spectrum of compound **8ka & 8kb**

400 MHz, $CHCl_3$

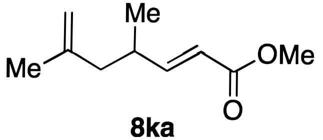

8ka

+

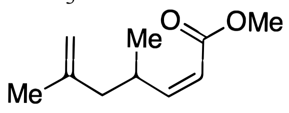

8kb

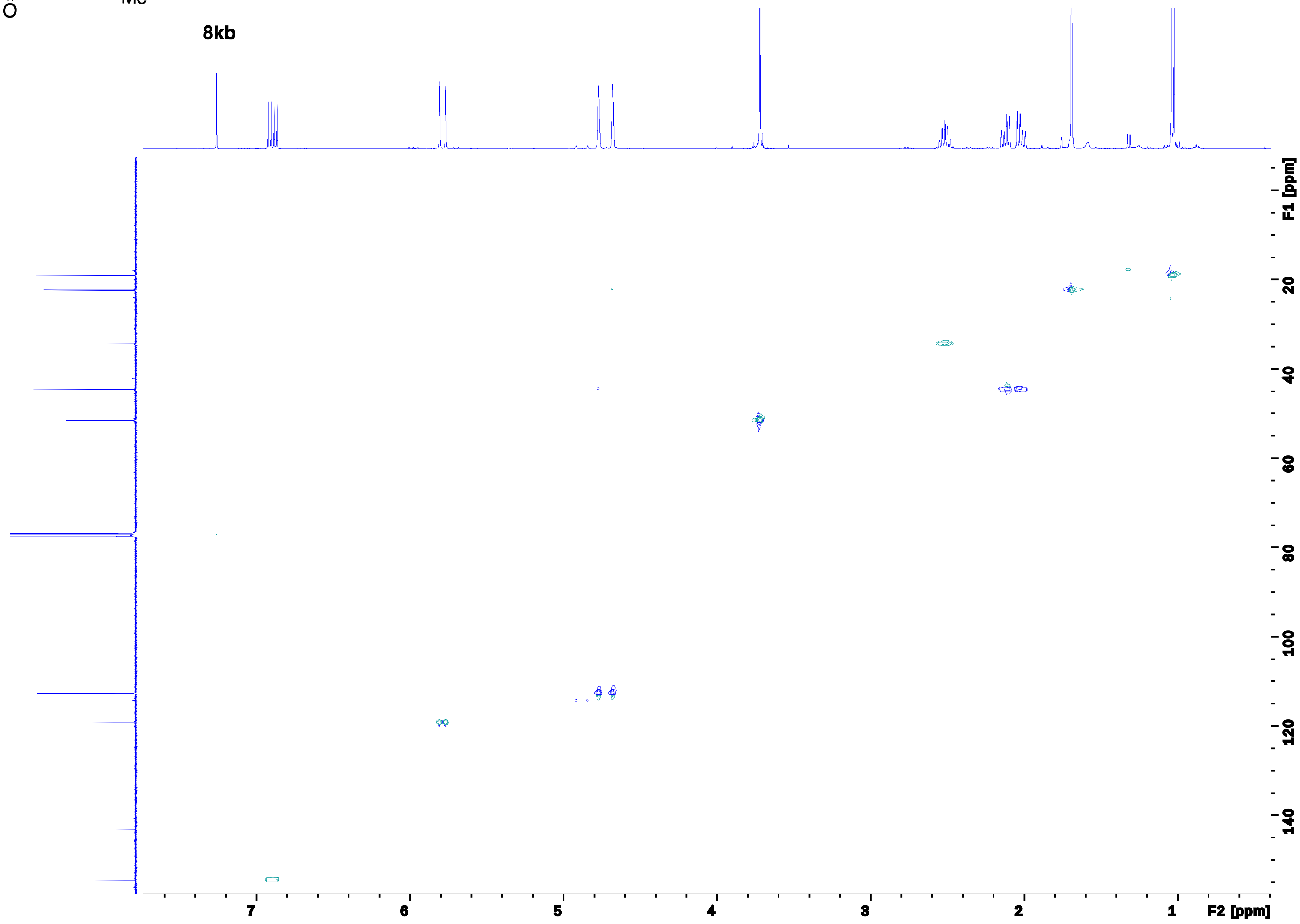

$^1H/^{13}C$ HSQC spectrum of compounds **8ka & 8kb**

400 MHz, $CDCl_3$

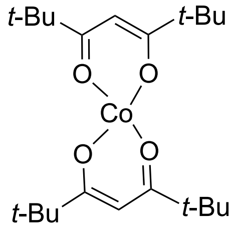

10

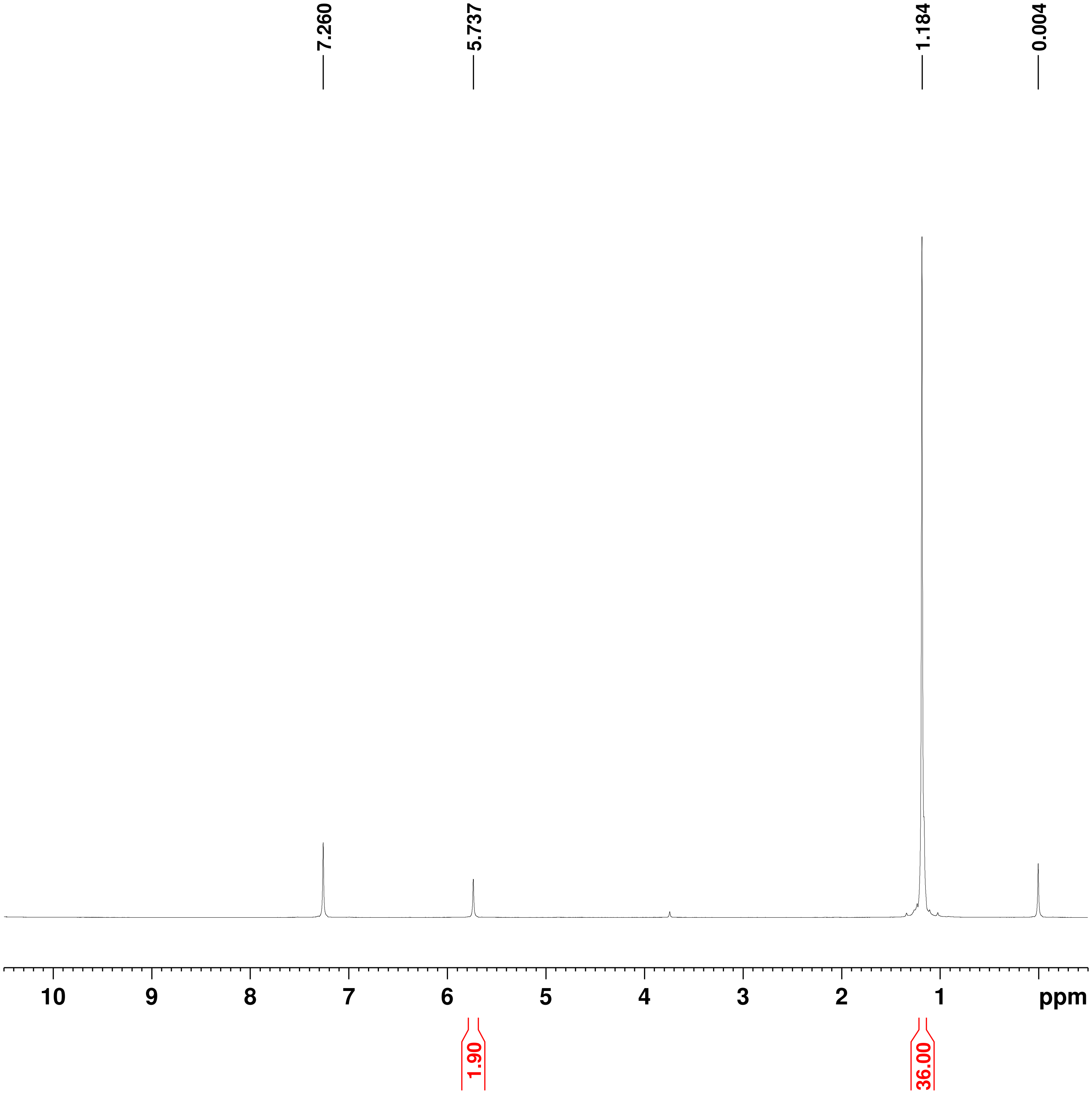

^1H spectrum of **10**

471 MHz, $(CD_3)_2CO$:$CDCl_3$ (1:1 v/v)

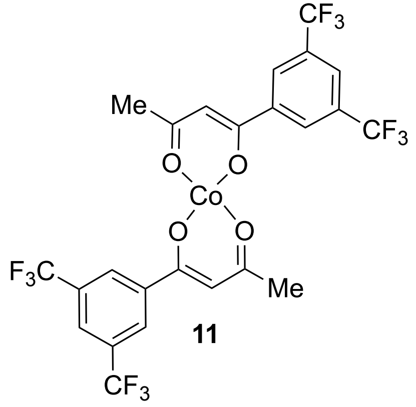

11

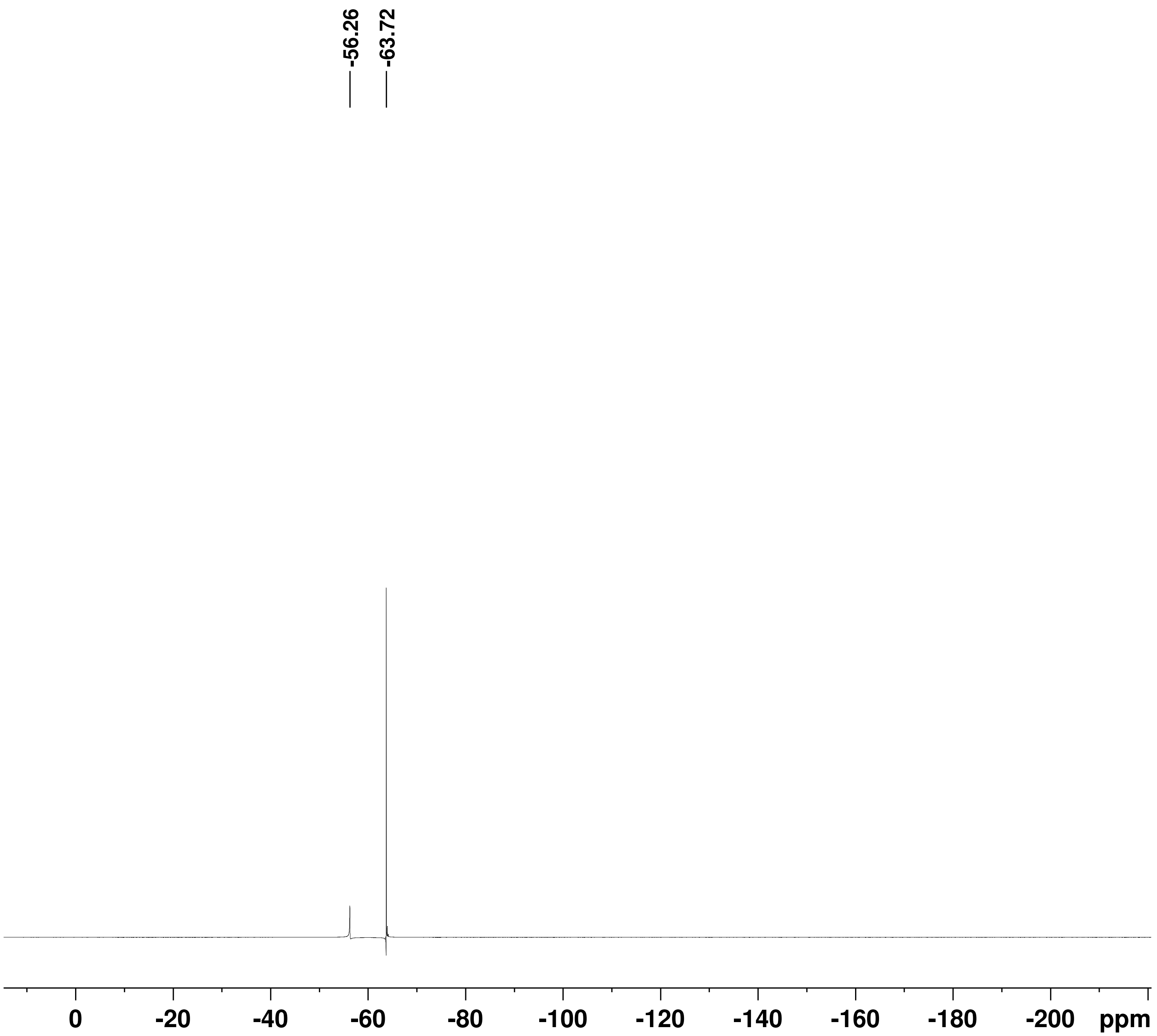

^{19}F spectrum of **11**

400 MHz, $CDCl_3$

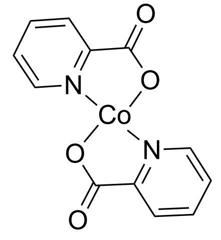

15

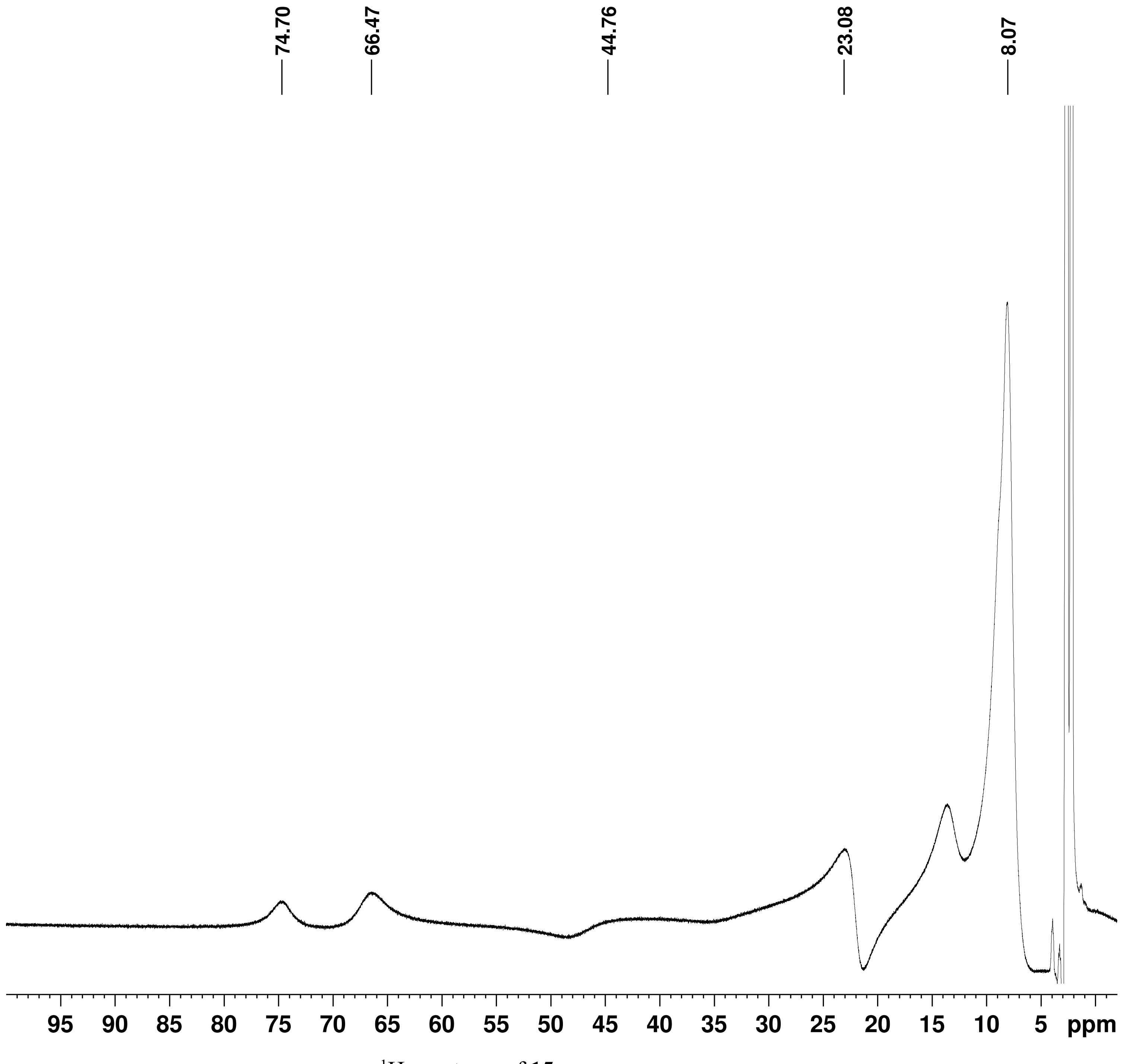

^{1}H spectrum of **15**

400 MHz, C_6D_6

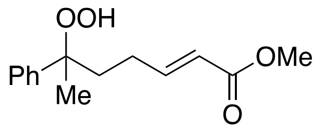

9a

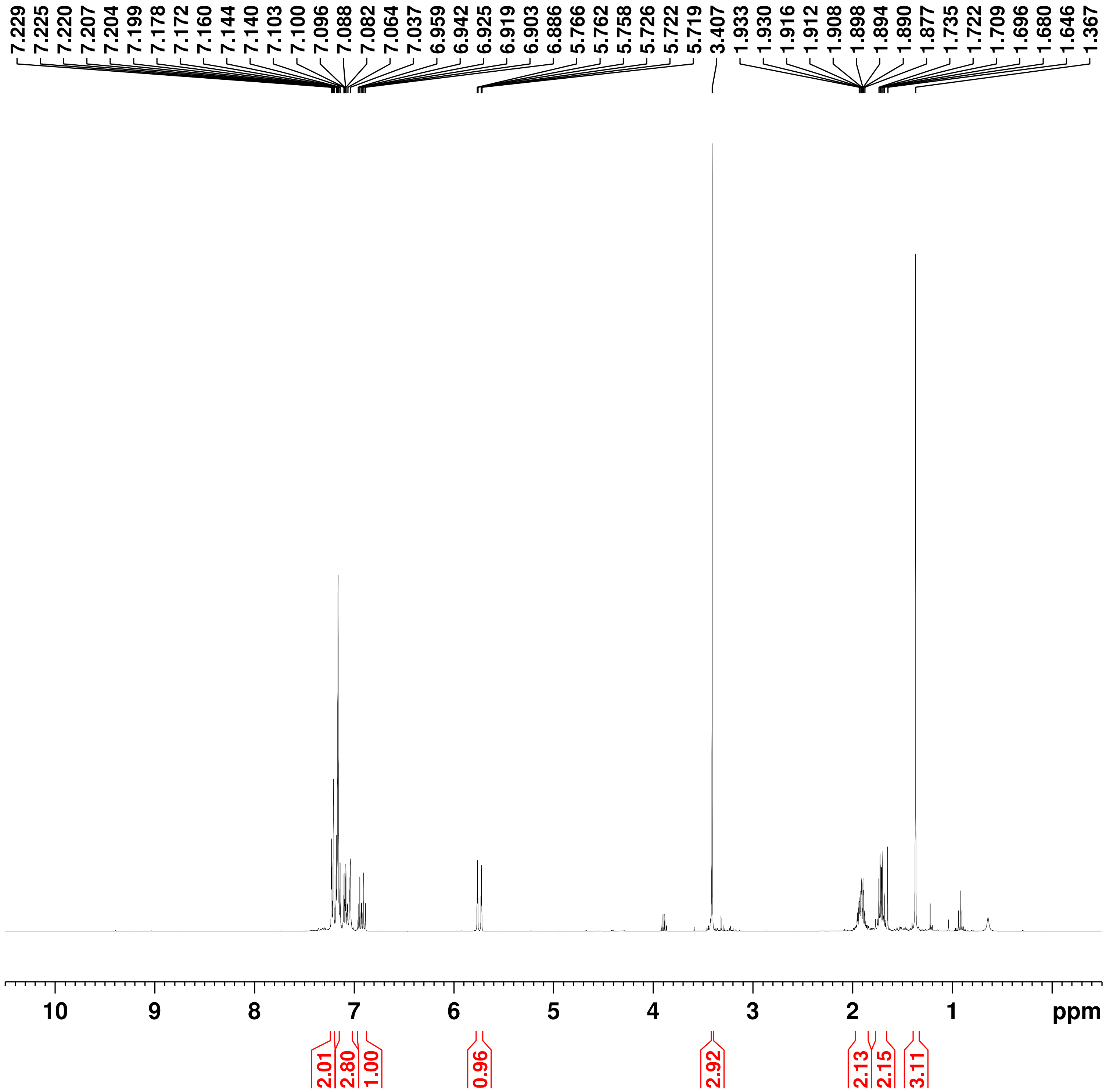

^{1}H spectrum of compound **9a**

100 MHz, C_6D_6

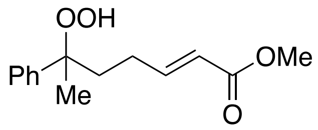

9a

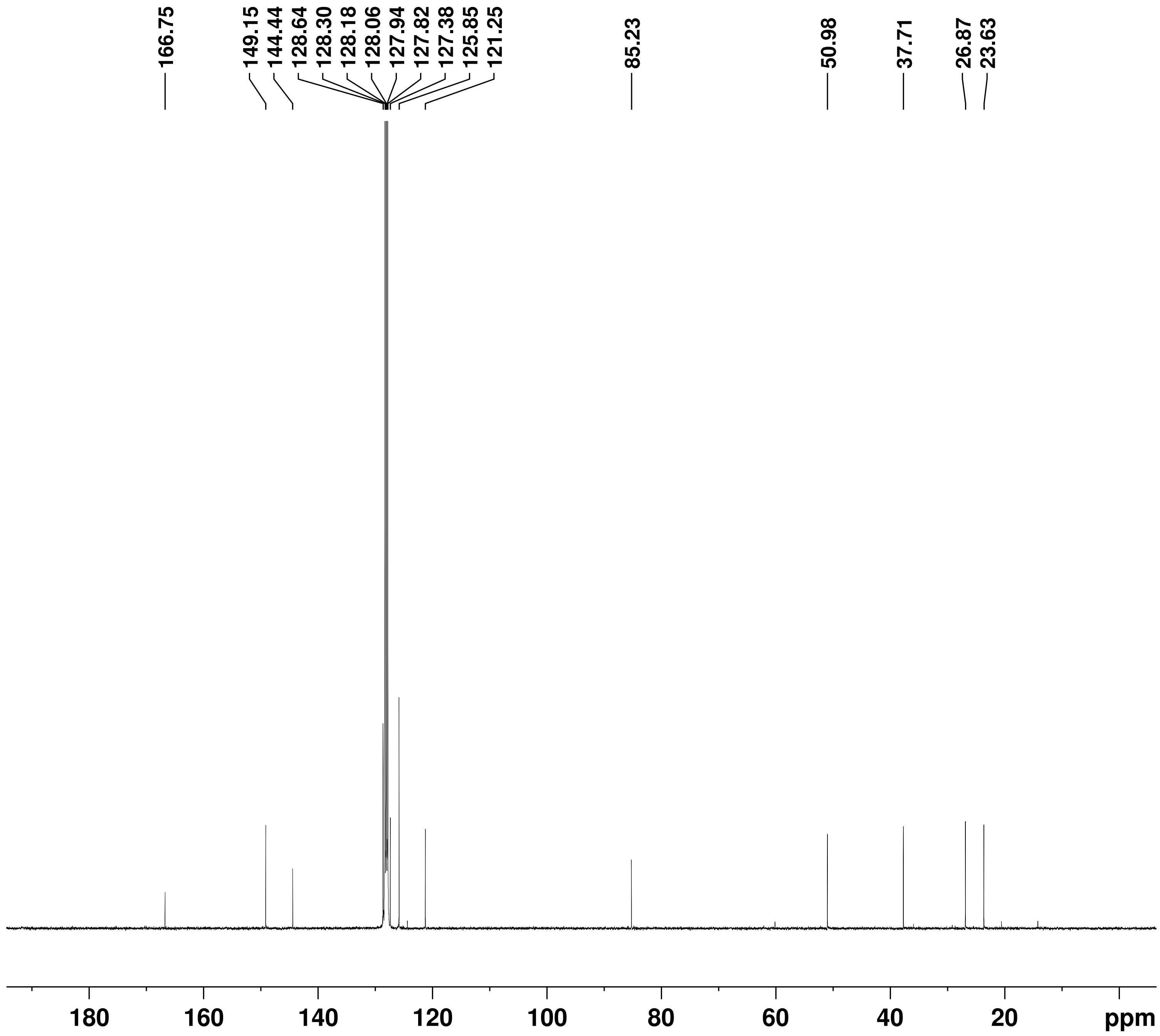

^{13}C spectrum of compound **22c**

400 MHz, C_6D_6

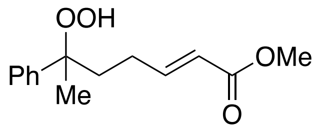

9a

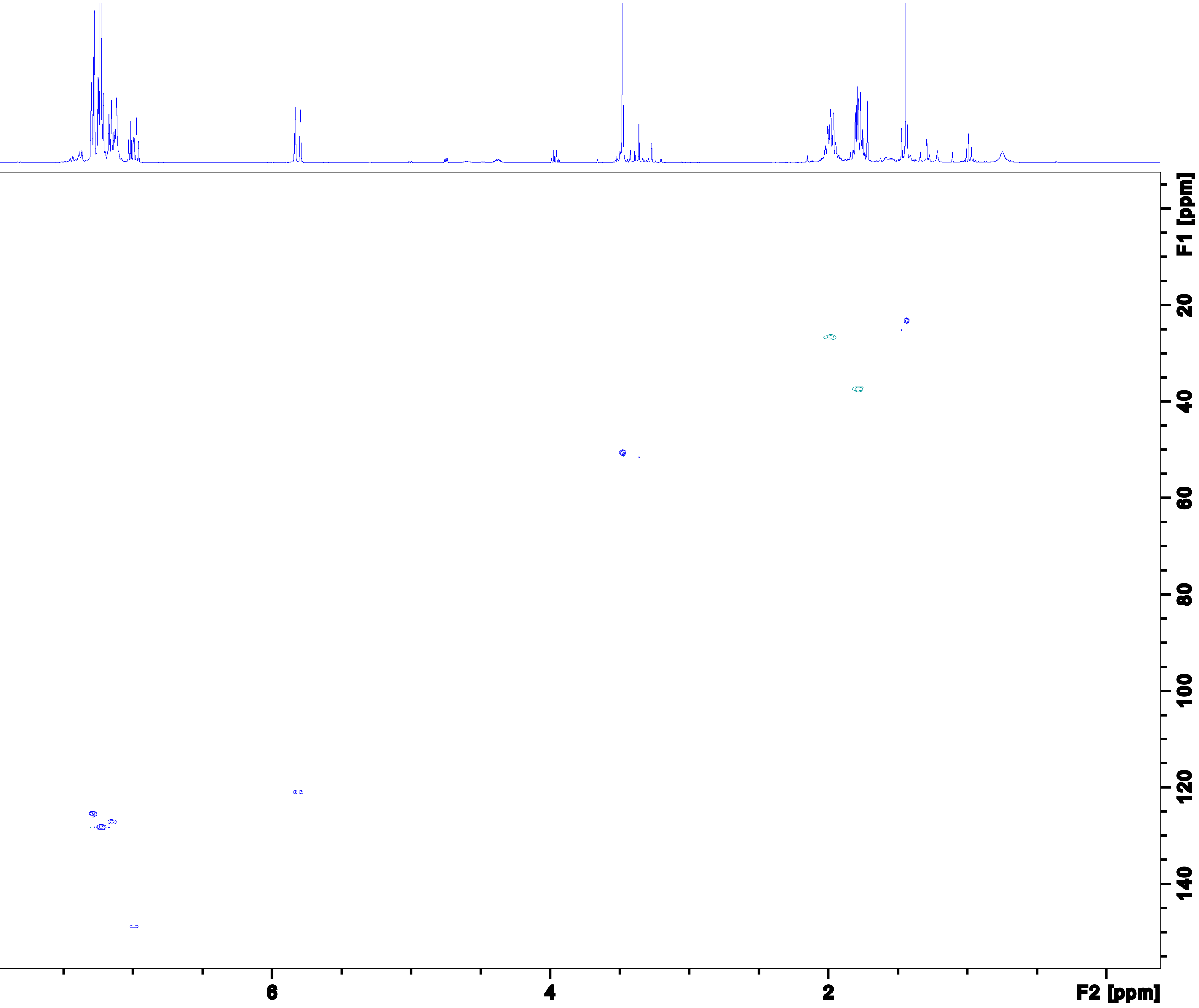

$^{1}H/^{13}C$ HSQC spectrum of compound **9a**

400 MHz, $CDCl_3$

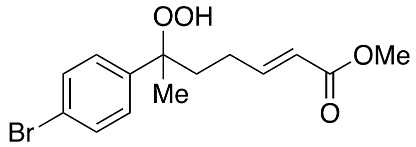

9b

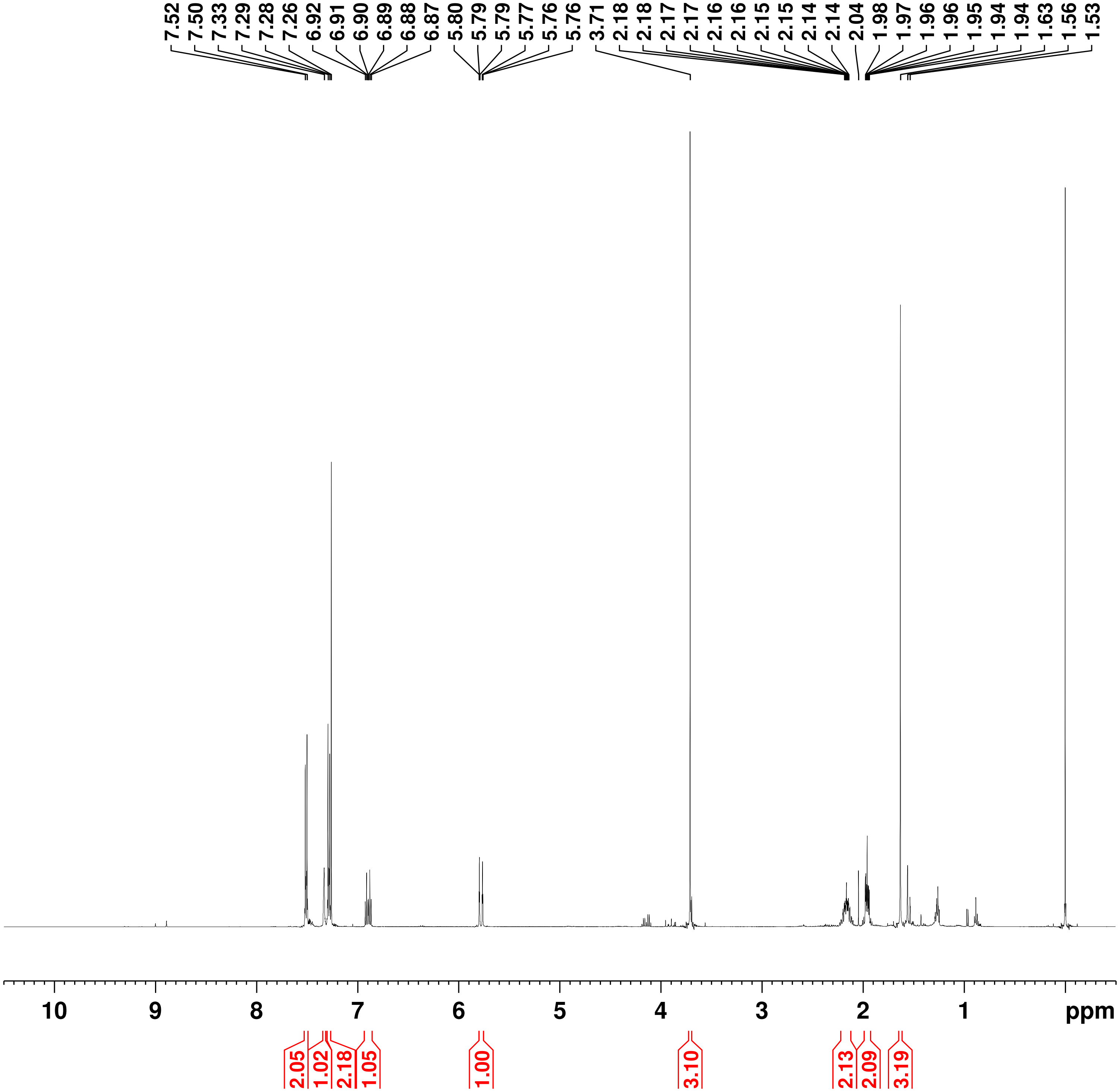

^{1}H spectrum of compound **9b**

100 MHz, $CDCl_3$

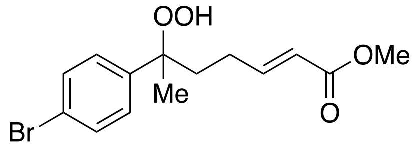

9b

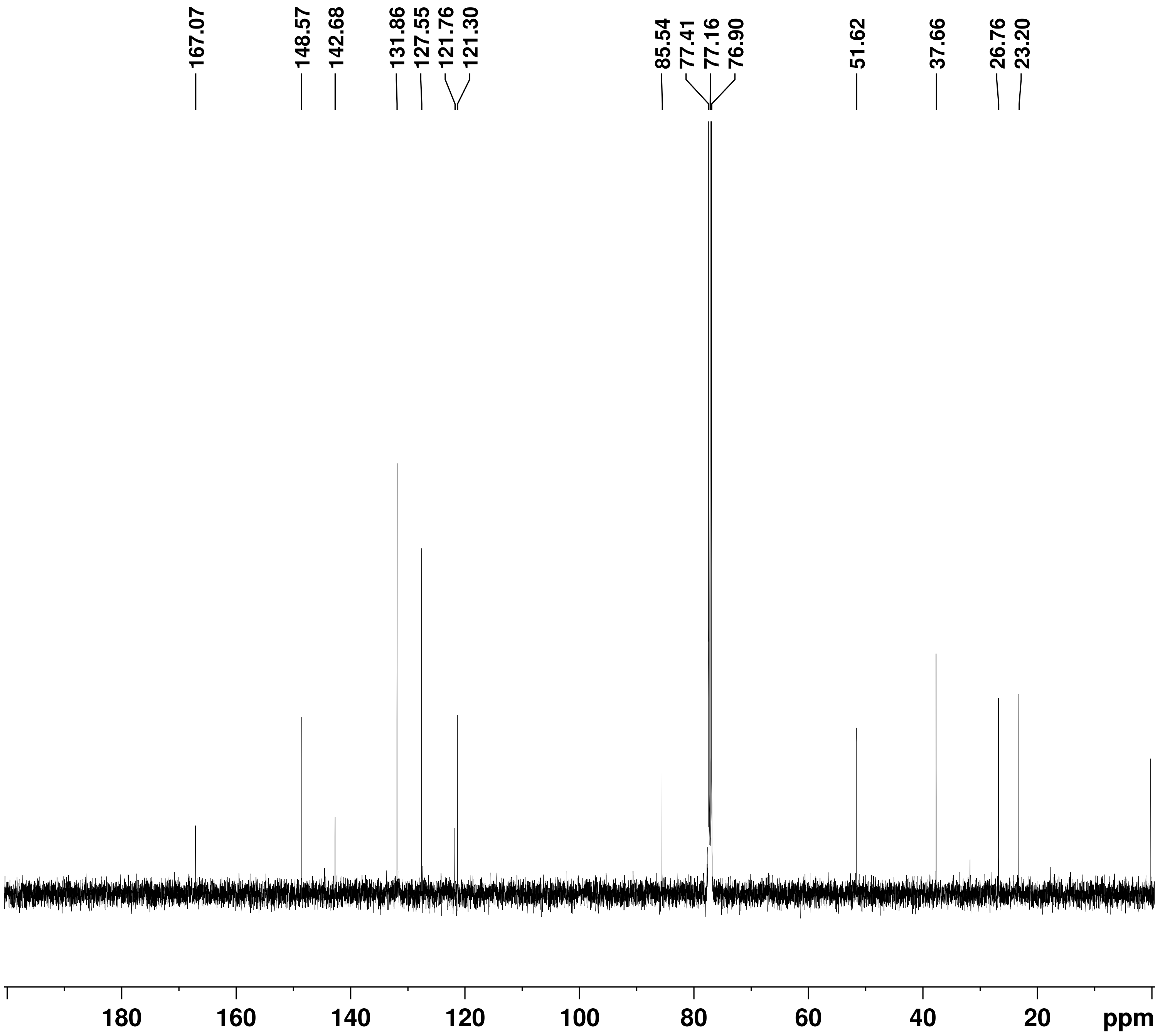

^{13}C spectrum of compound **9b**

400 MHz, $CDCl_3$

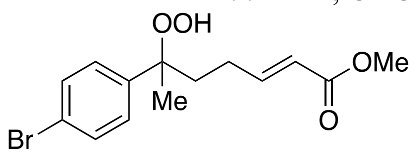

9b

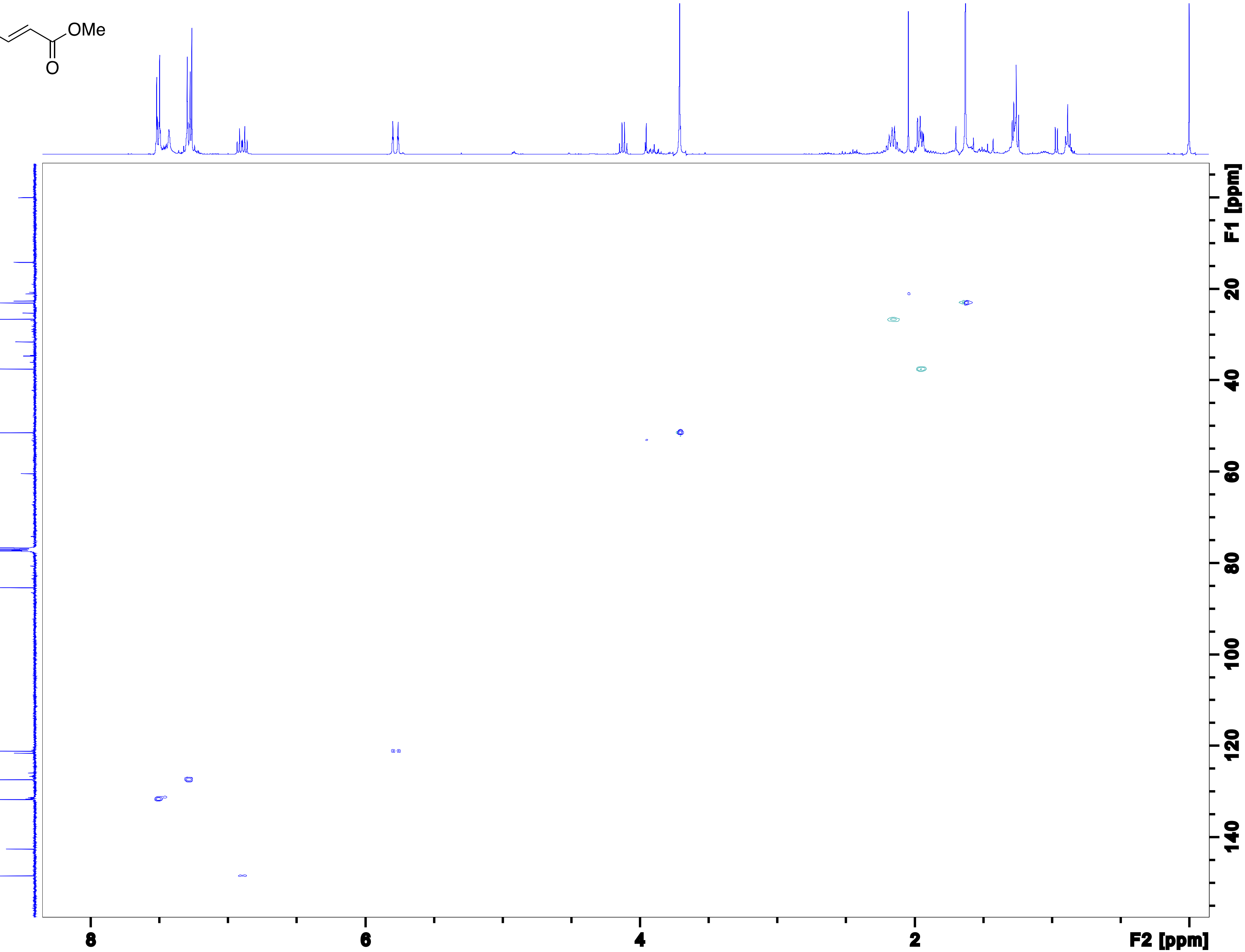

$^{1}H/^{13}C$ spectrum of compound **9b**

400 MHz, $CDCl_3$

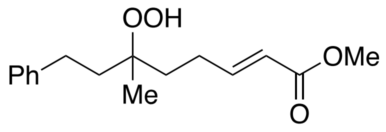

9c

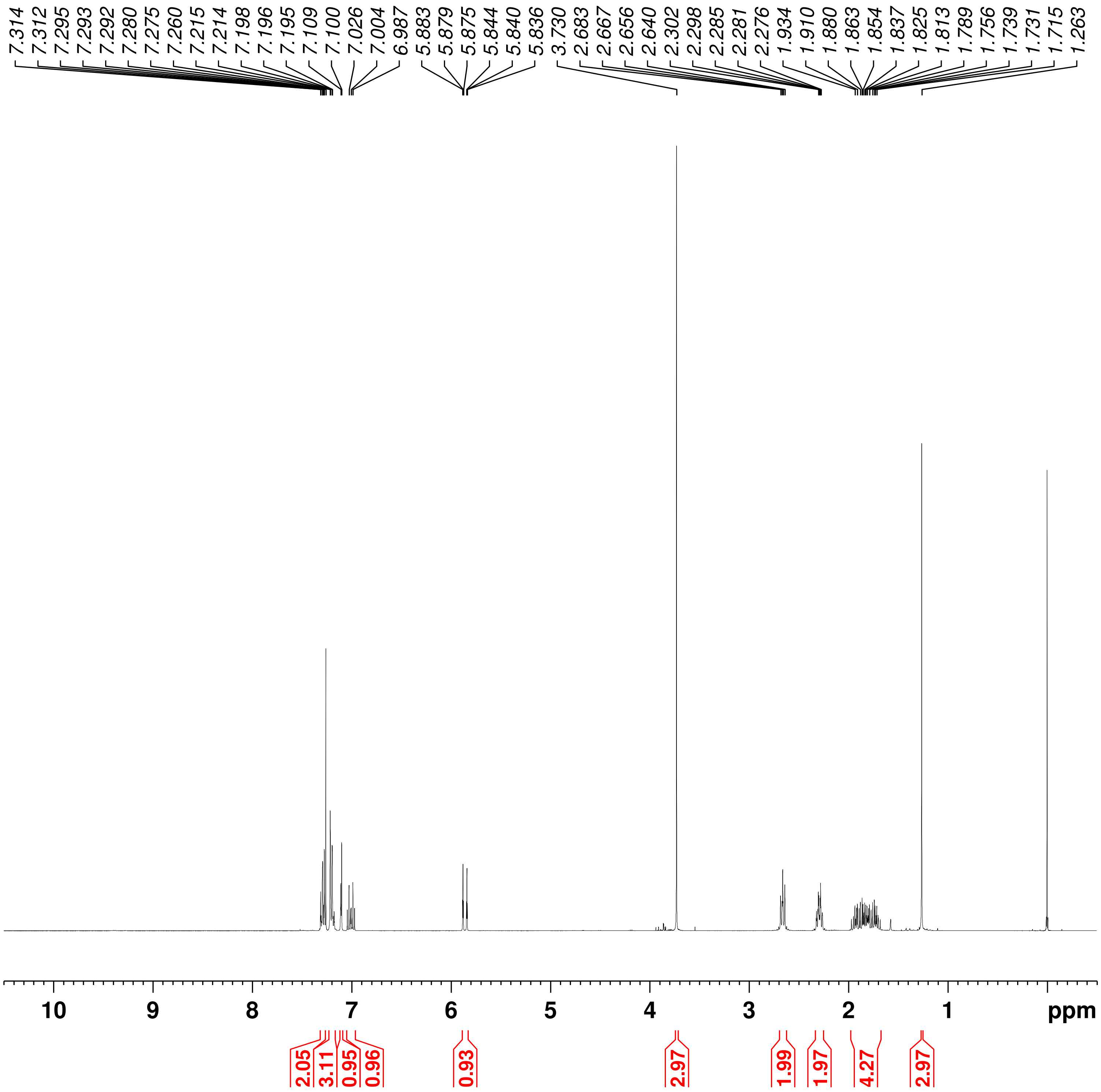

^{1}H spectrum of compound **9c**

100 MHz, $CDCl_3$

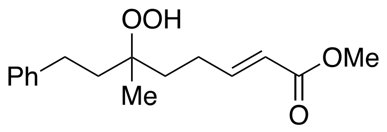

9c

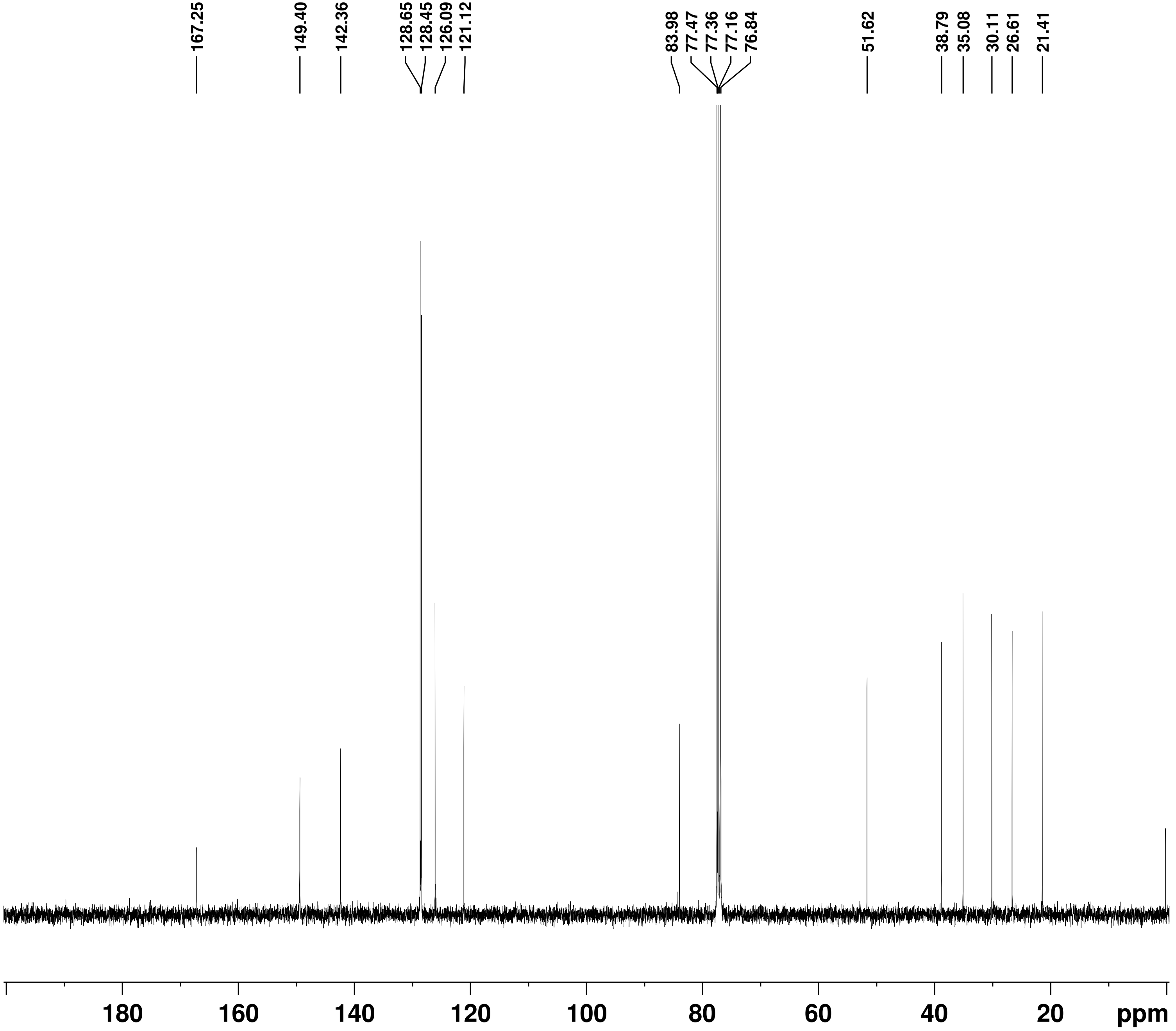

^{13}C spectrum of compound **9c**

400 MHz, $CDCl_3$

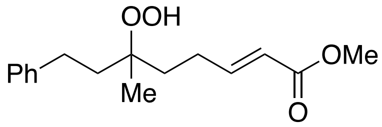

9c

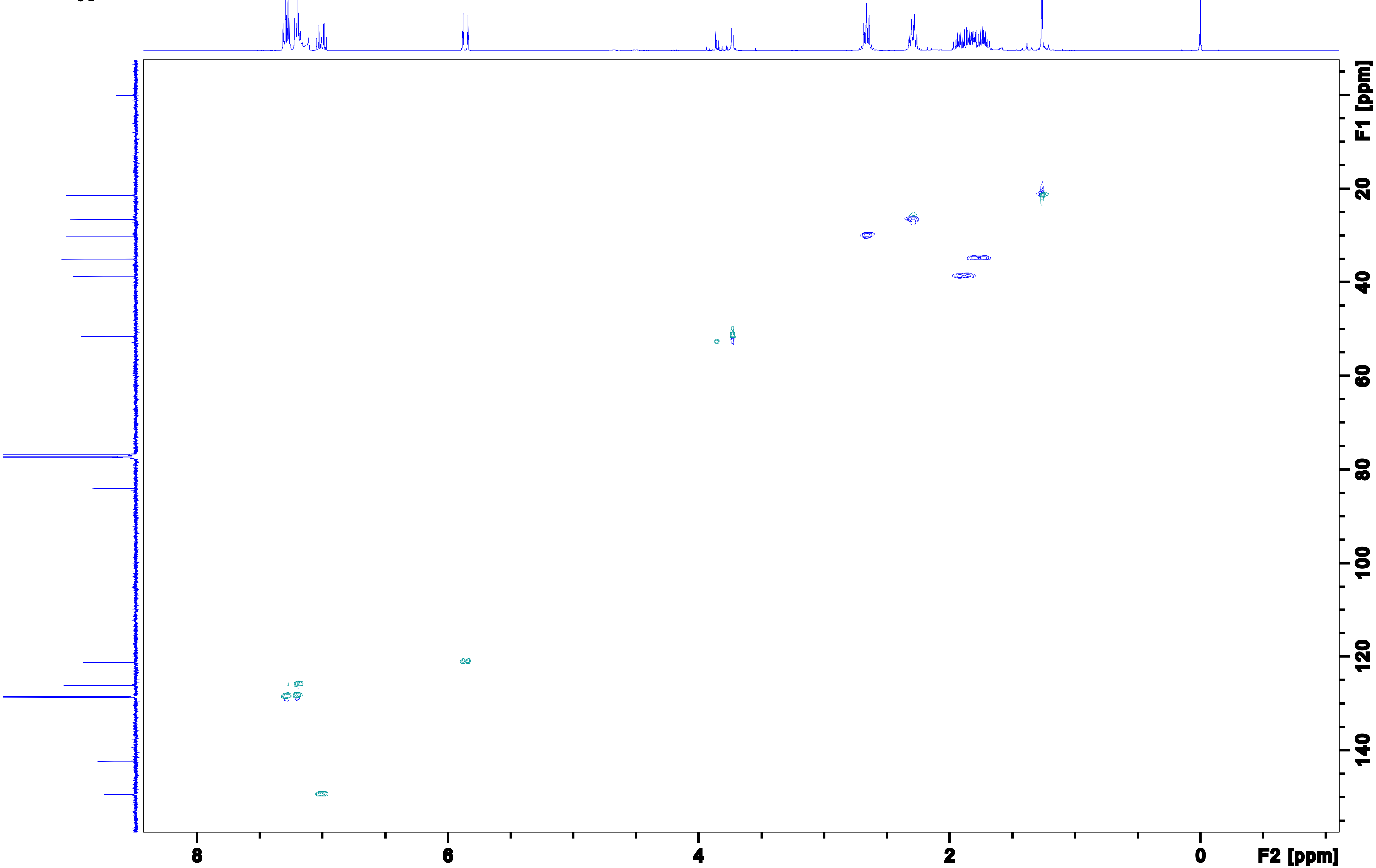

$^1H/^{13}C$ HSQC spectrum of compound **9c**

400 MHz, C_6D_6

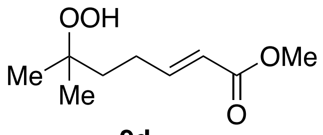

9d

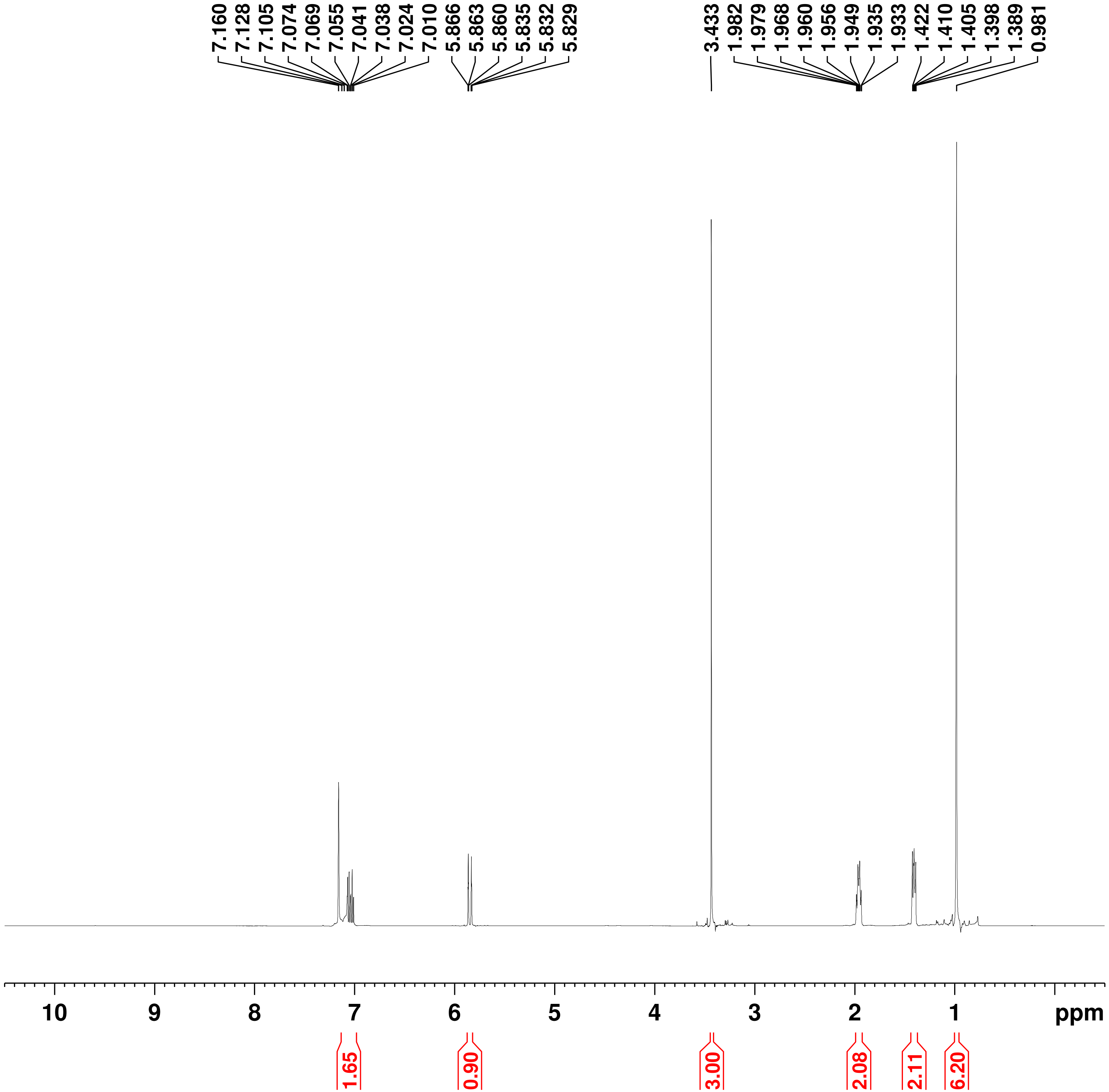

^1H spectrum of compound **9d**

100 MHz, C_6D_6

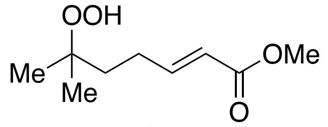

9d

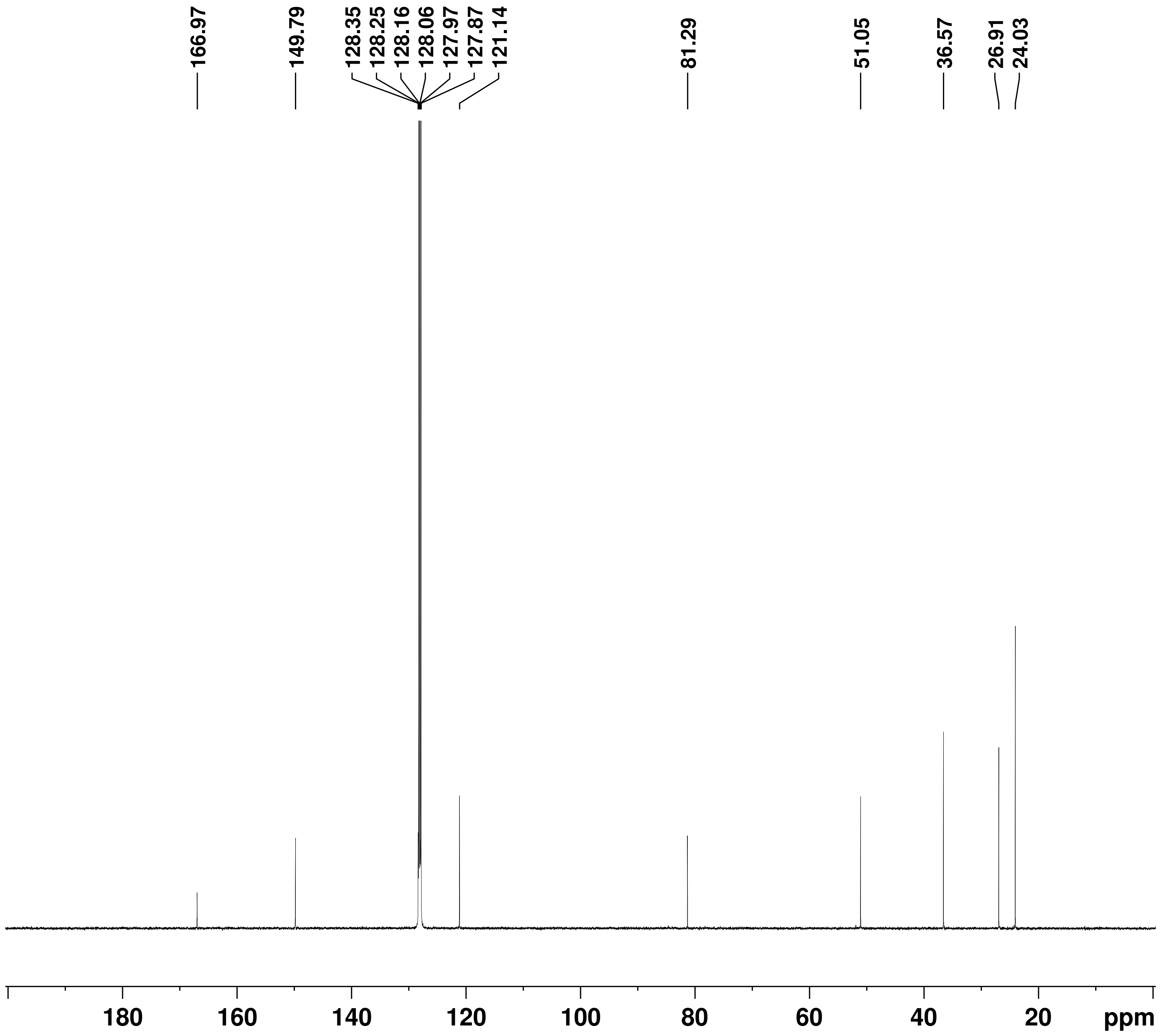

^{13}C spectrum of compound **9d**

100 MHz, C_6D_6

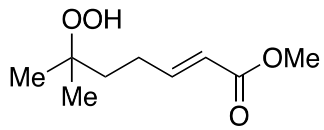

9d

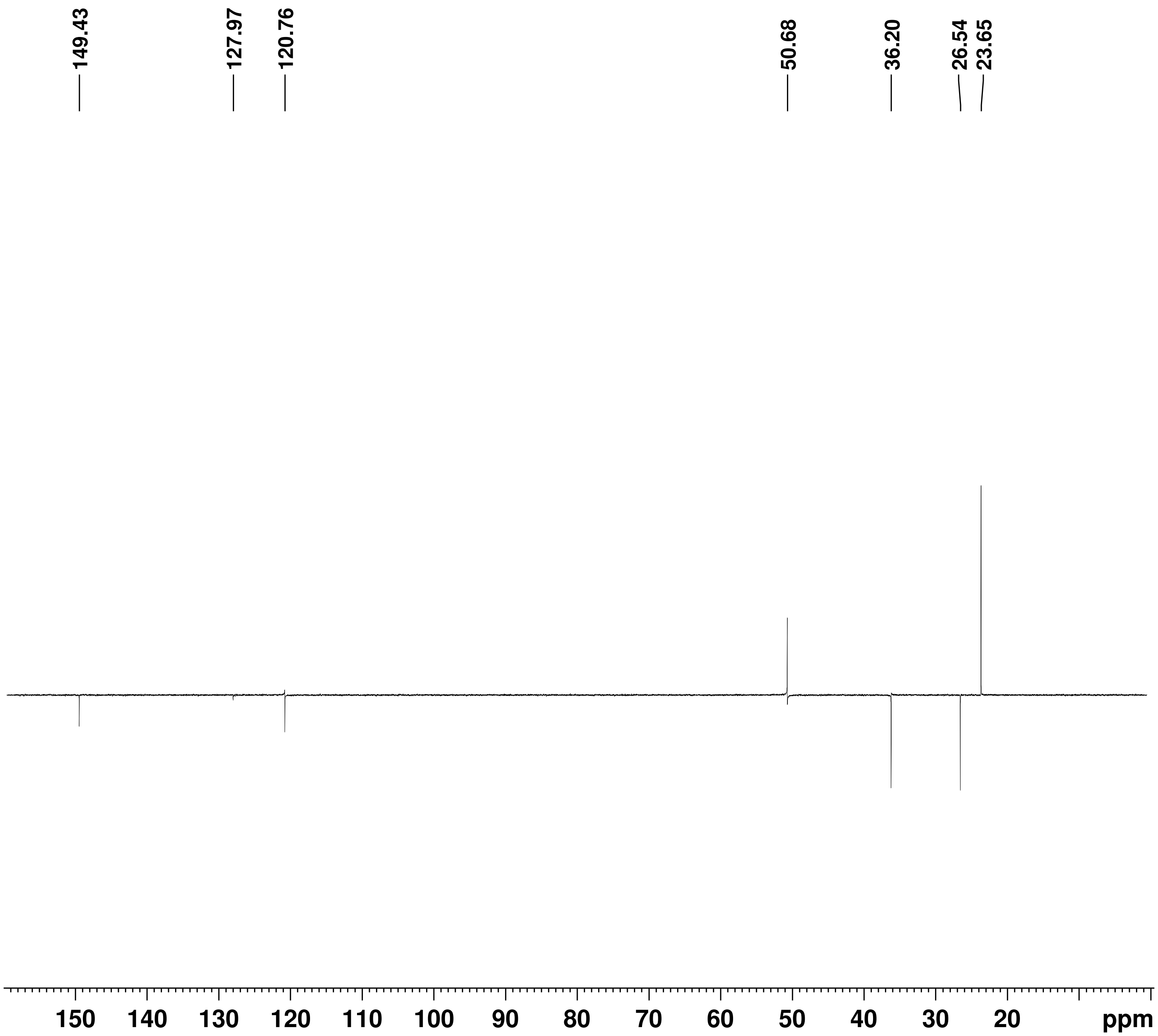

^{13}C DEPT spectrum of compound **9d**

400 MHz, C_6D_6

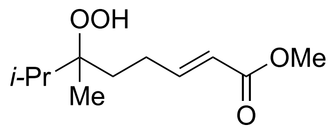

9e

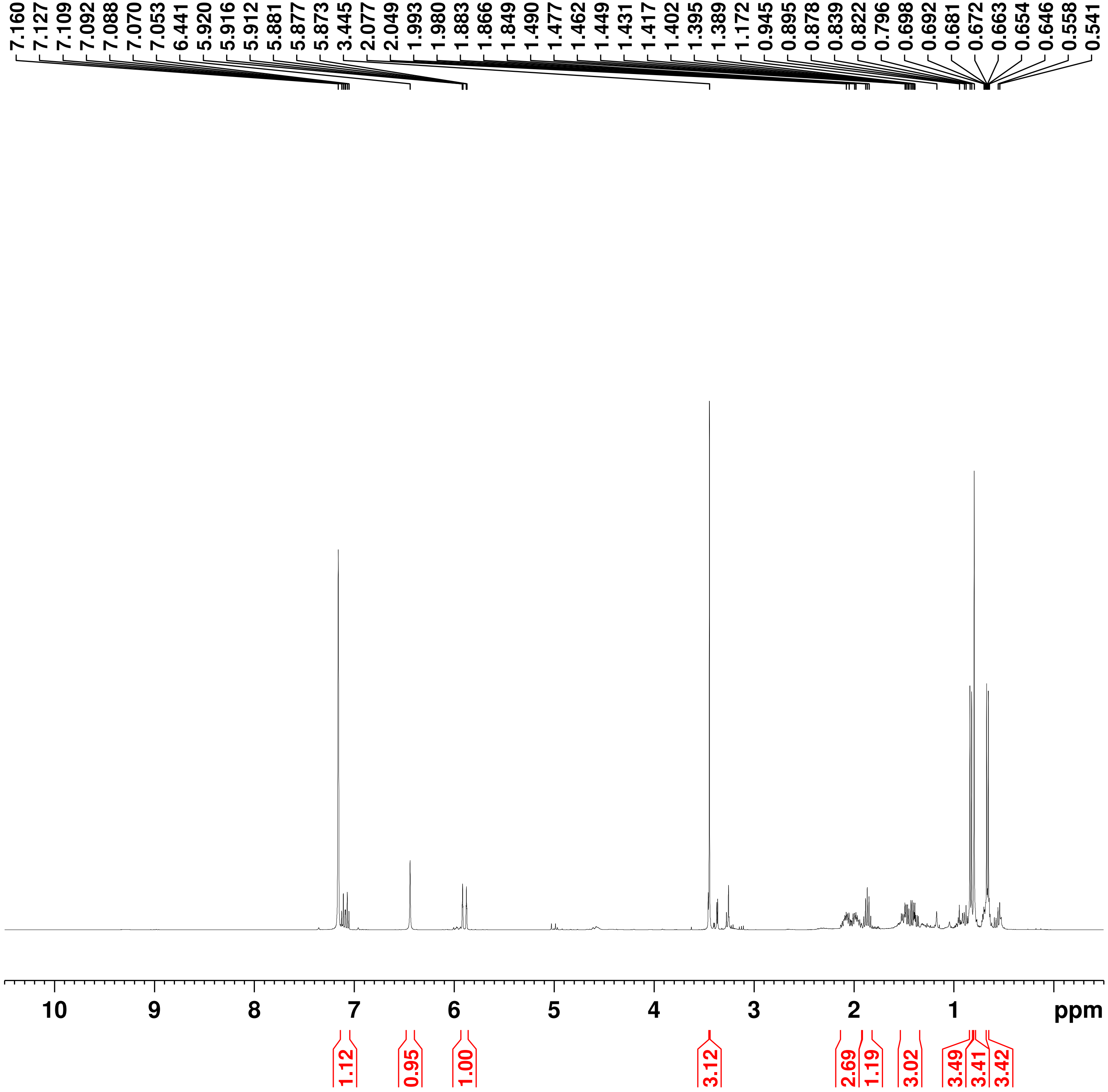

^{1}H spectrum of compound **9e**

100 MHz, C_6D_6

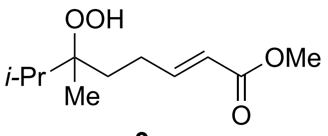

9e

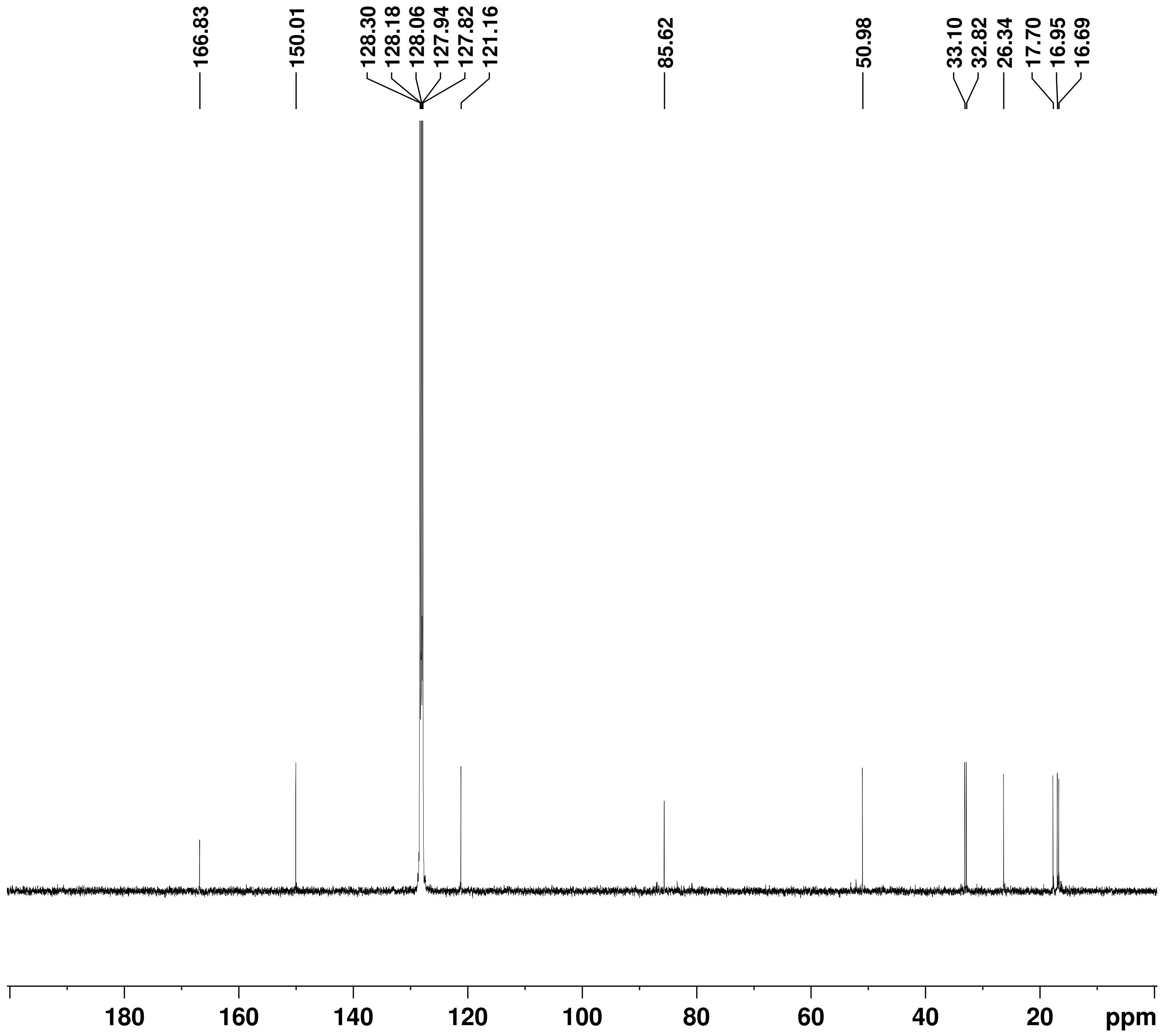

^{13}C spectrum of compound **9e**

400 MHz, C_6D_6

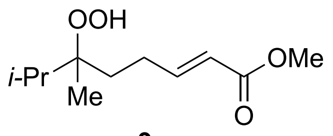

9e

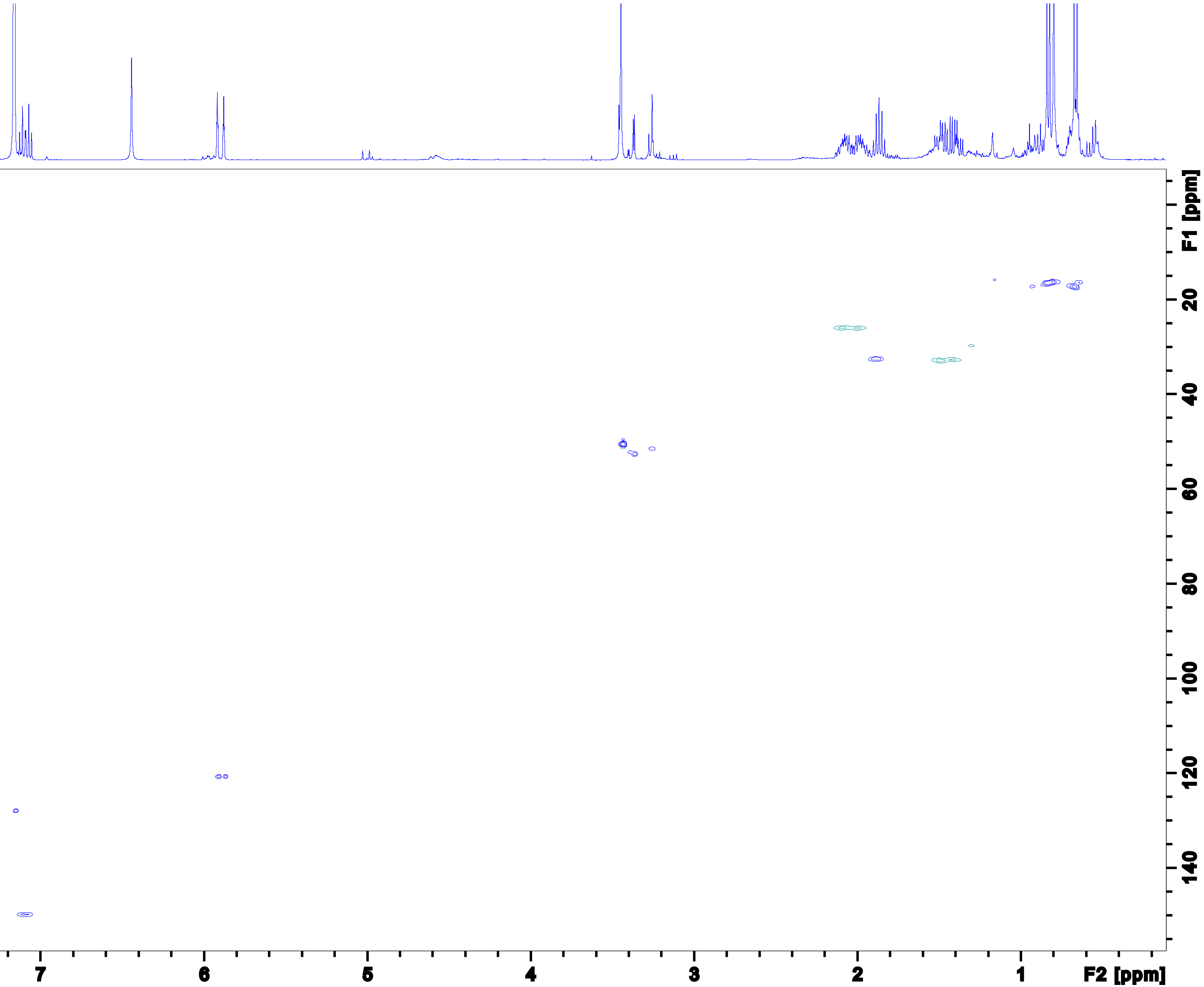

$^1H/^{13}C$ HSQC spectrum of compound **9e**

400 MHz, $CDCl_3$

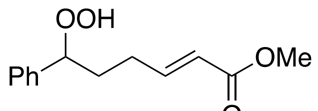

9f

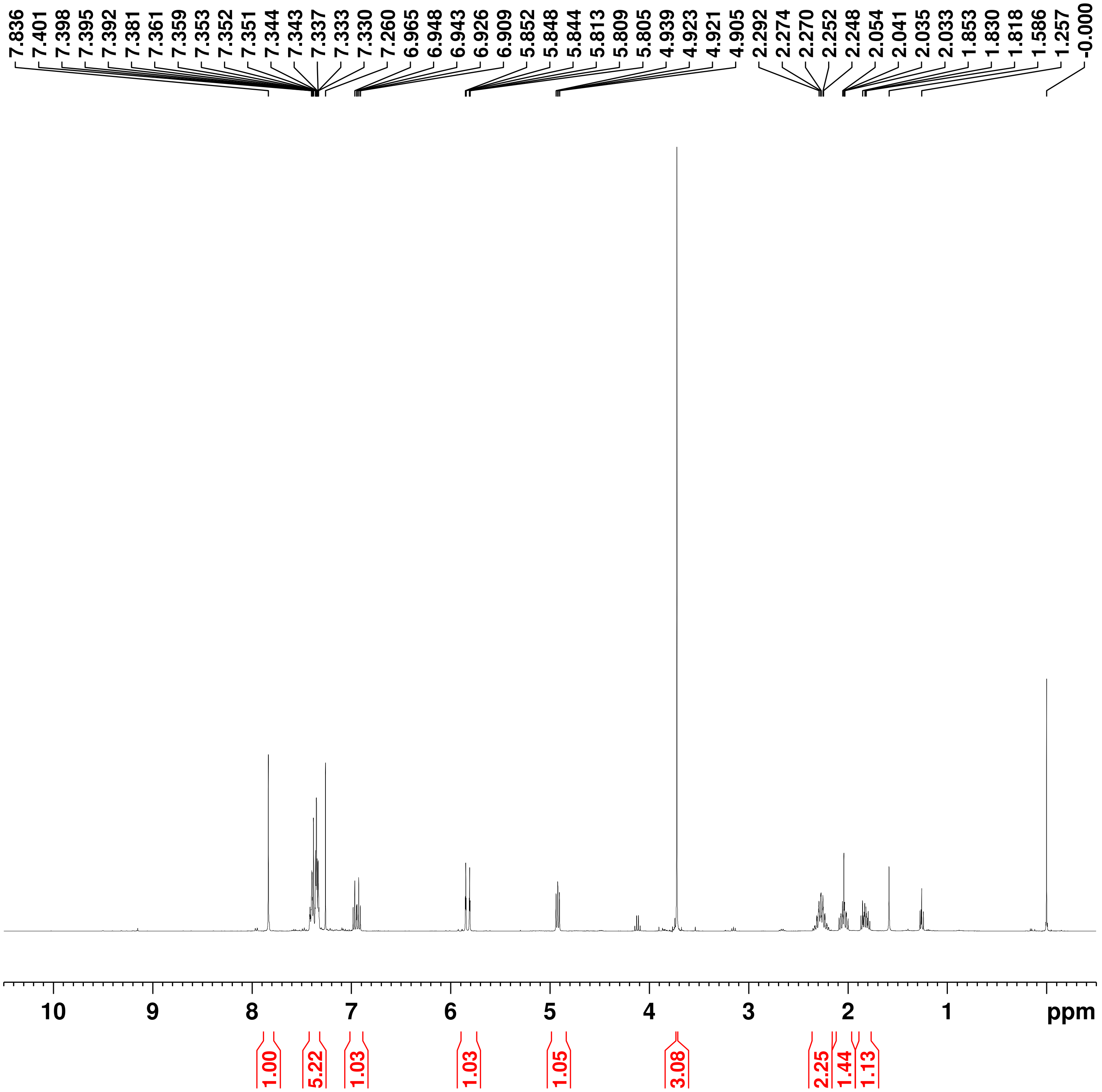

^1H spectrum of compound **9f**

100 MHz, $CDCl_3$

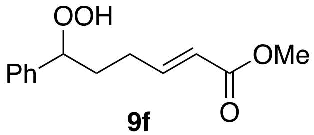

9f

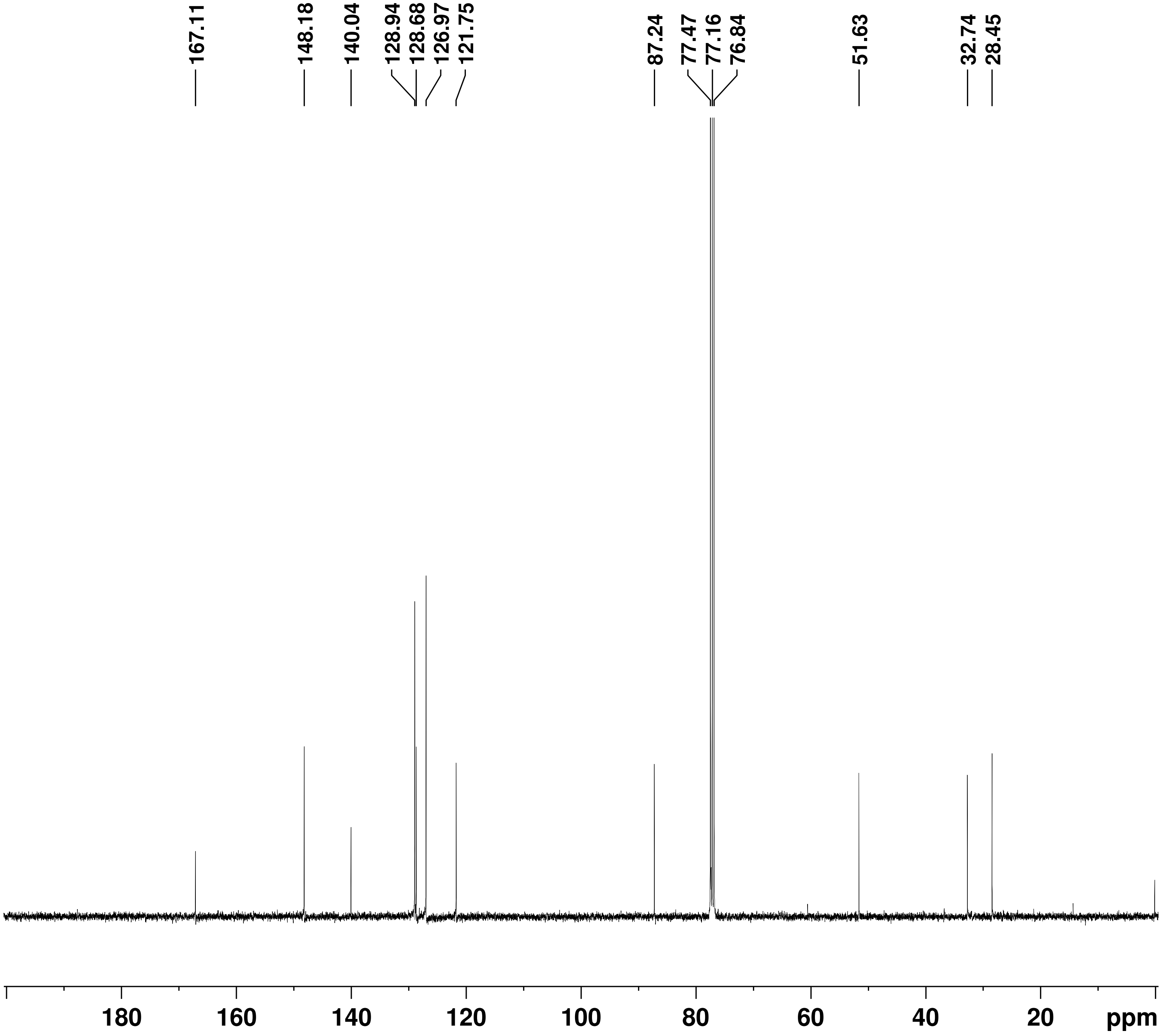

^{13}C HSQC spectrum of compound **9f**

400 MHz, $CDCl_3$

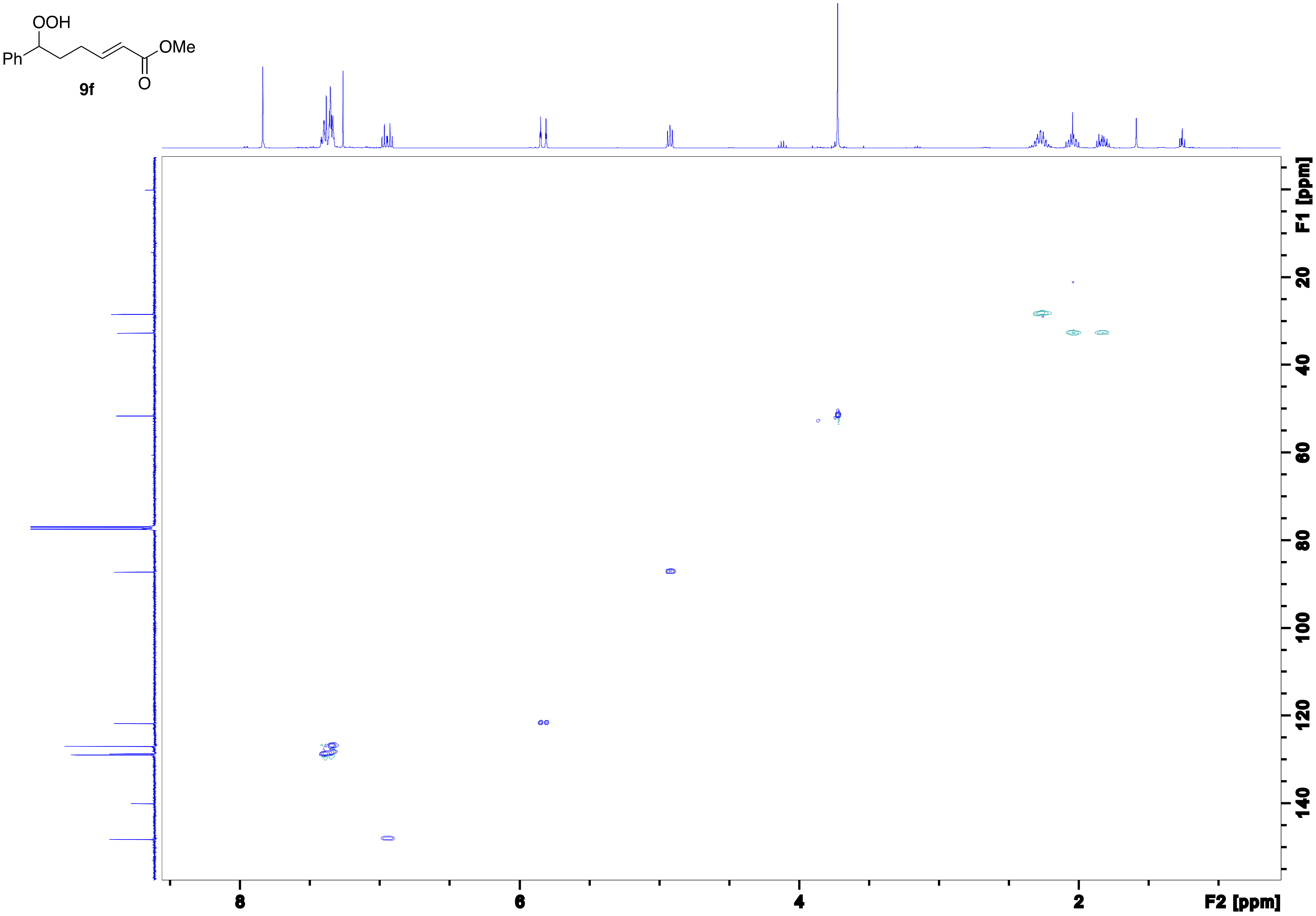

9f

$^1H/^{13}C$ HSQC spectrum of compound **9f**

400 MHz, $CDCl_3$

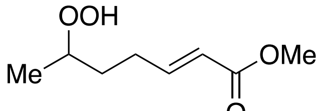

trans-**9g**

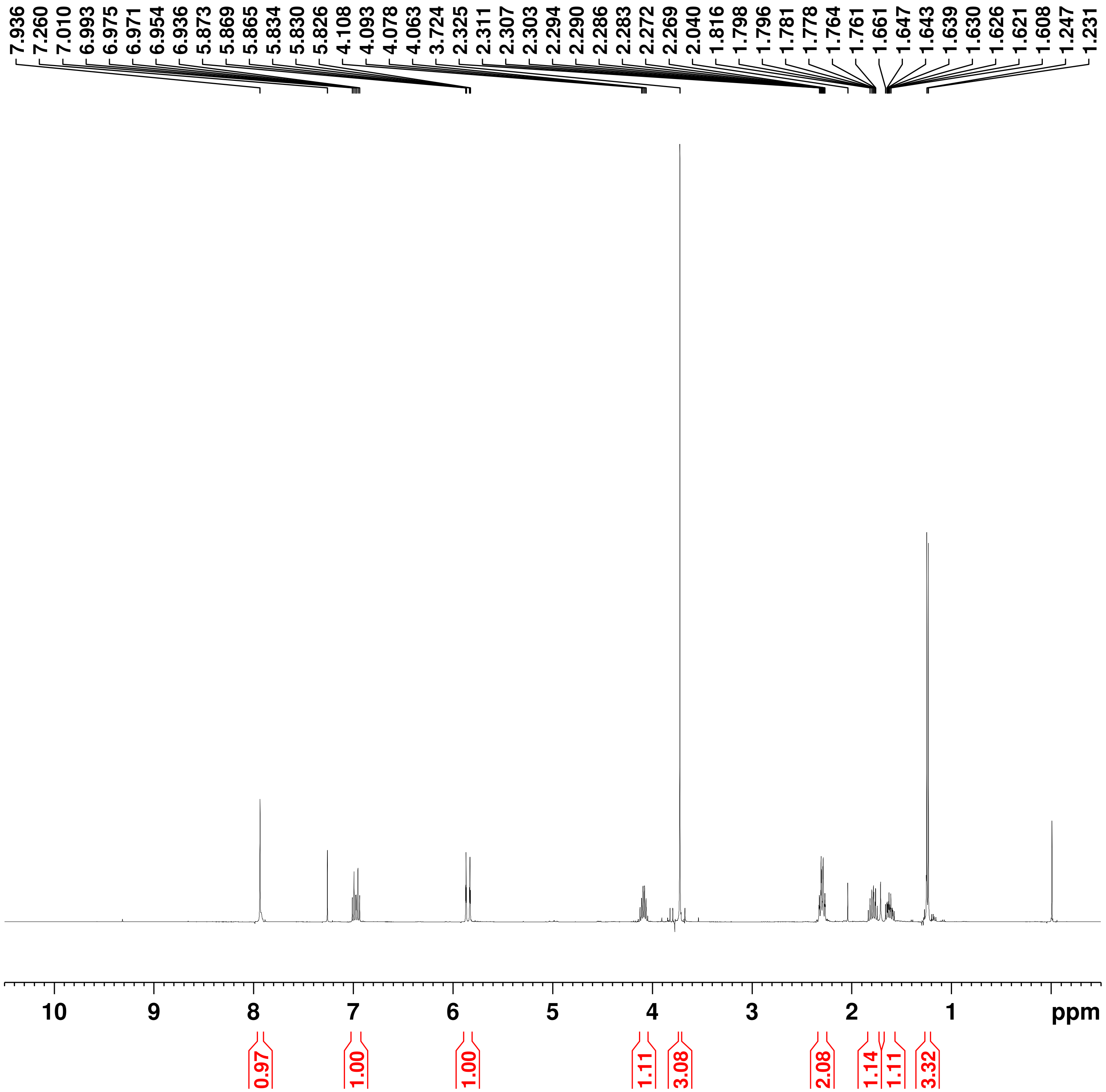

^{1}H spectrum of compound *trans*-**9g**

400 MHz, $CDCl_3$

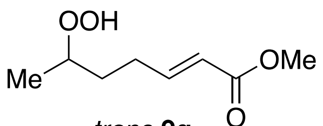

trans-**9g**

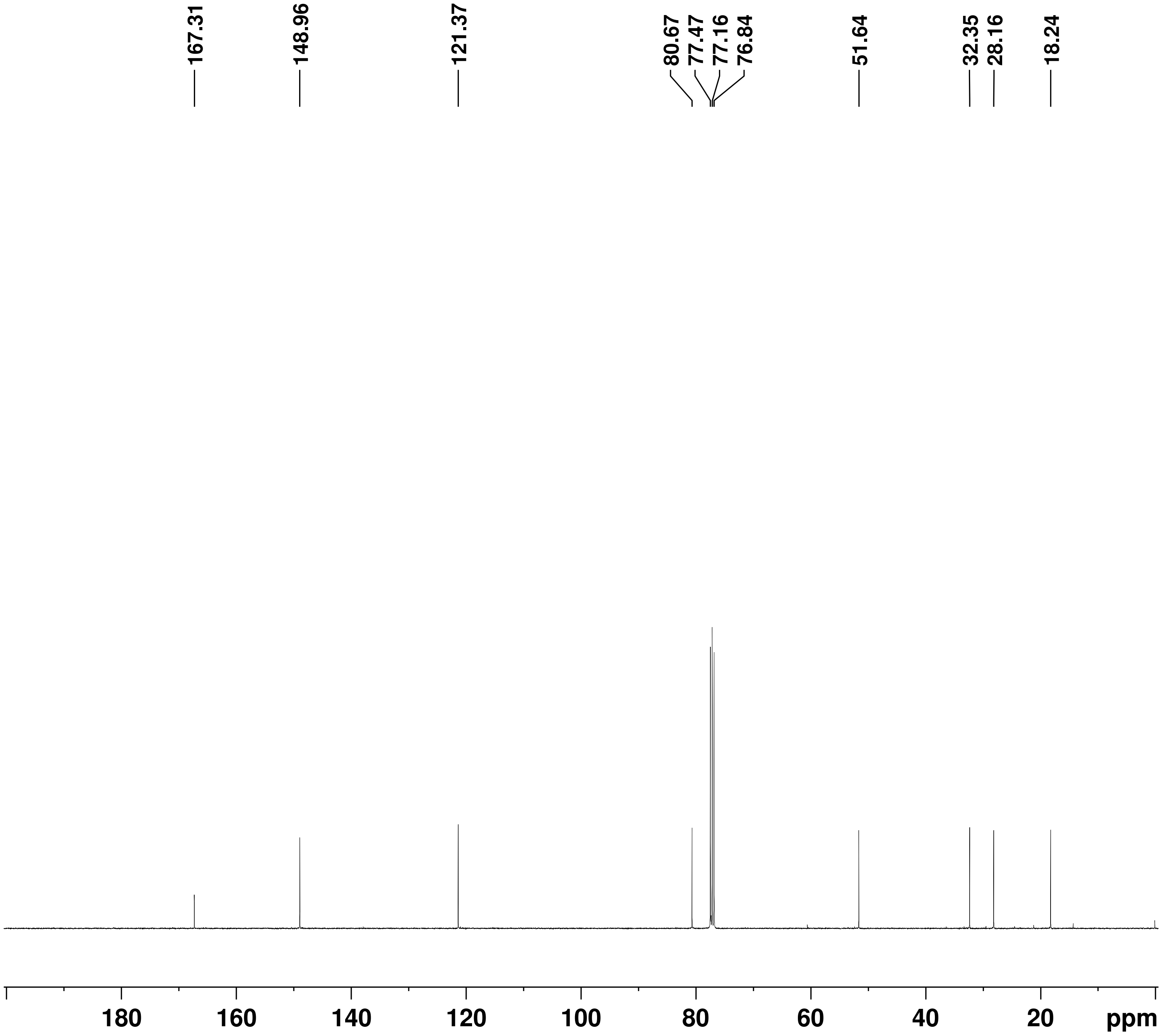

^{13}C HSQC spectrum of compound *trans*-**9g**

400 MHz, $CDCl_3$

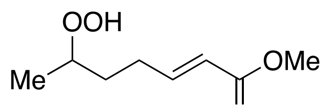

trans-**9g**

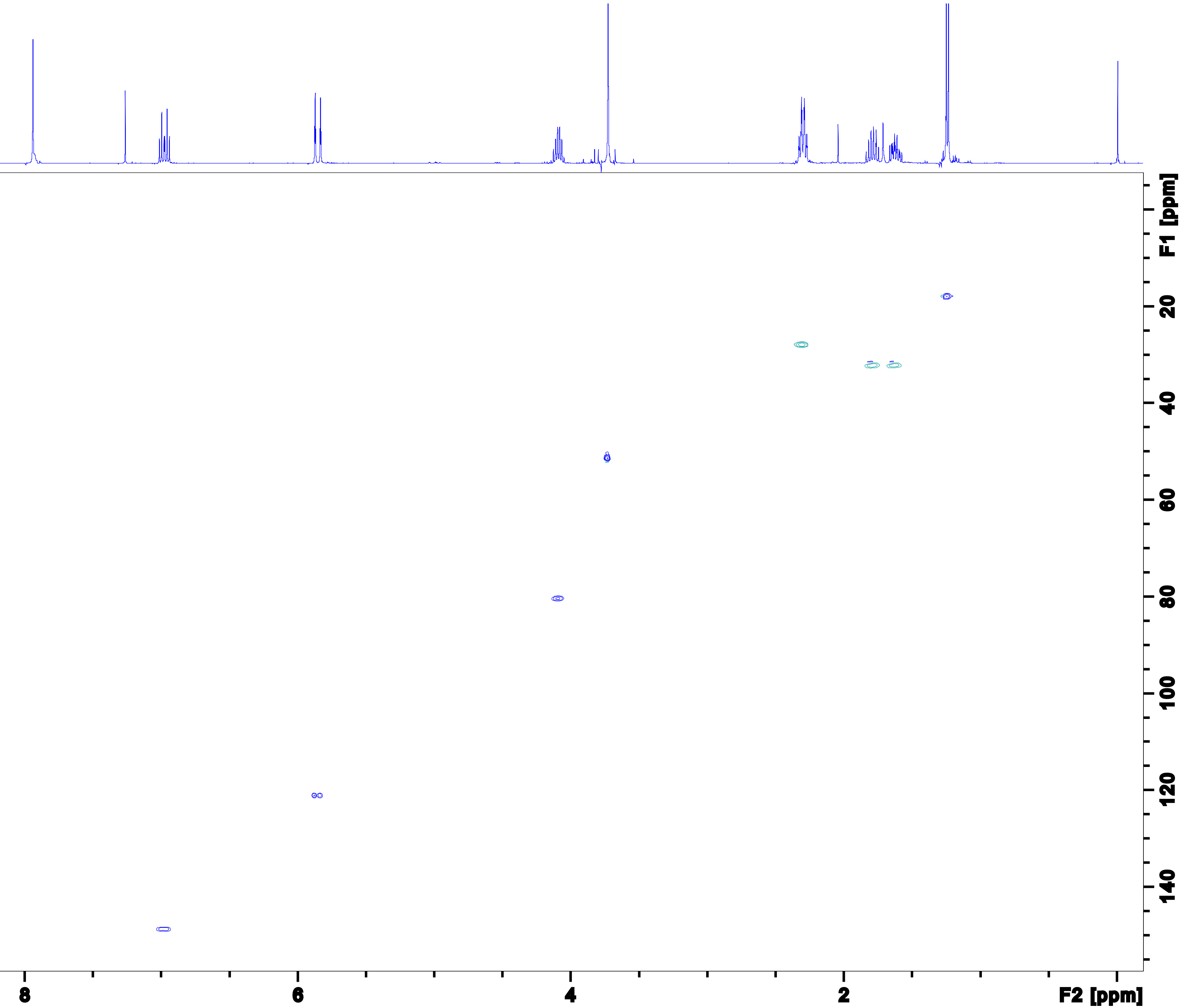

$^1H/^{13}C$ HSQC spectrum of compound *trans*-**9g**

400 MHz, C_6D_6

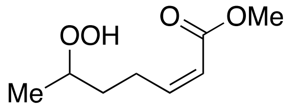

cis-**9g**

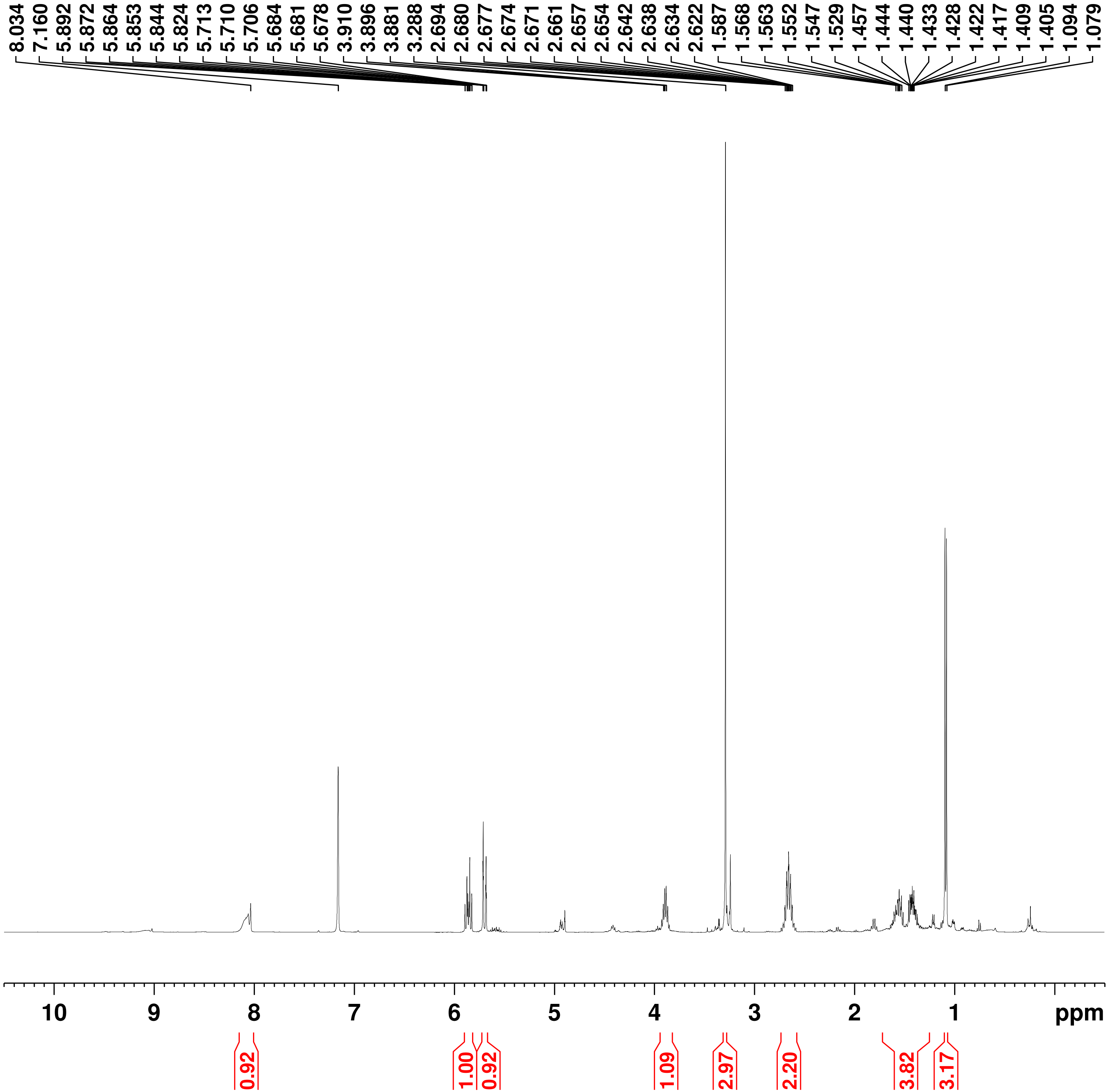

^1H spectrum of compound *cis*-**9g**

400 MHz, C_6D_6

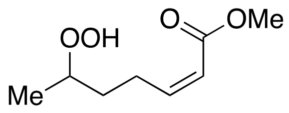

cis-**9g**

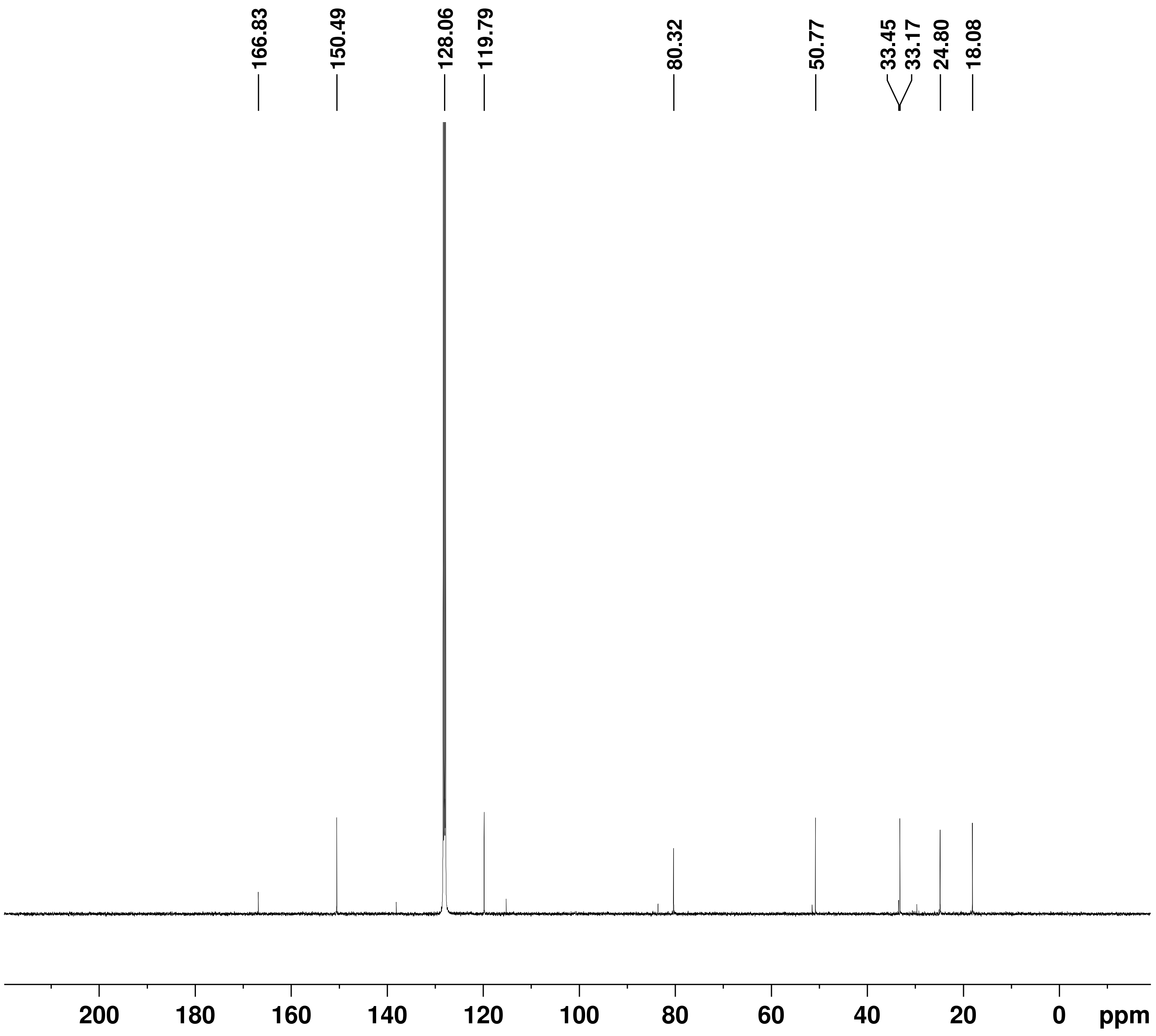

^{13}C spectrum of compound *cis*-**9g**

400 MHz, C_6D_6

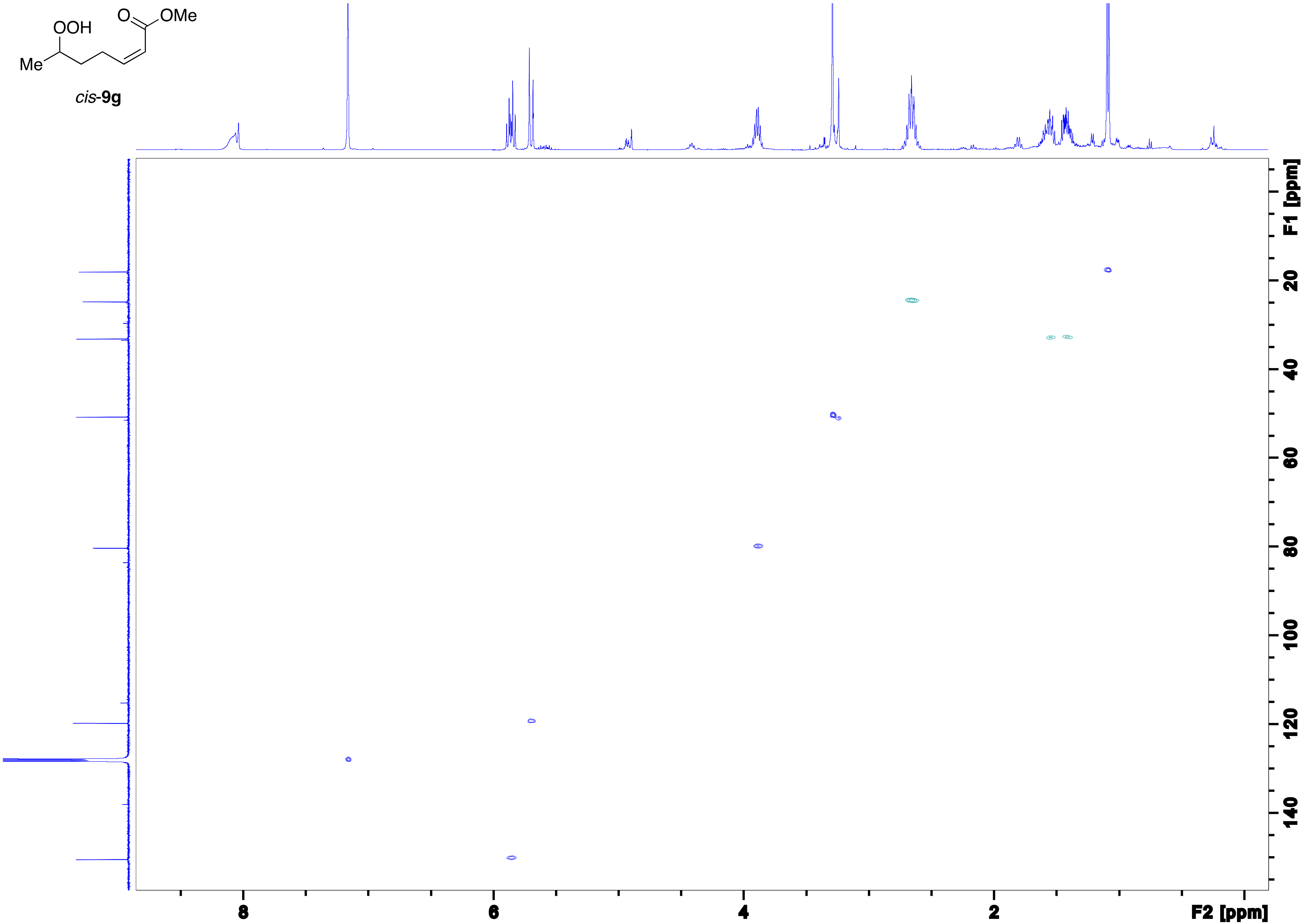

cis-**9g**

$^1H/^{13}C$ HSQC spectrum of compound *cis*-**9g**

400 MHz, C_6D_6

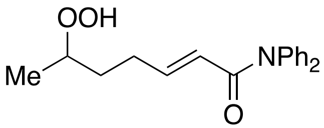

9h

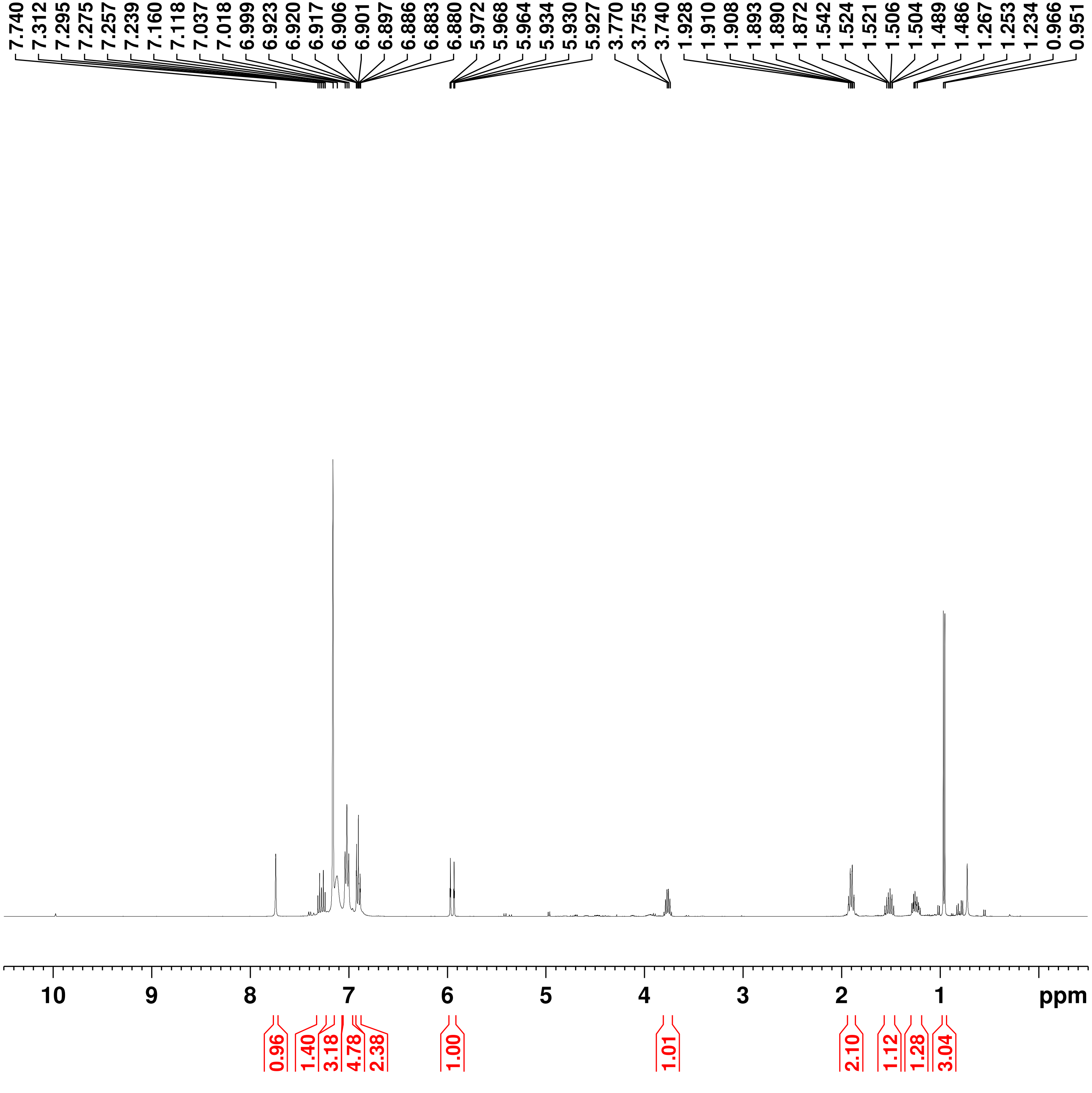

^{1}H spectrum of compound **9h**

100 MHz, C_6D_6

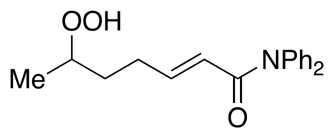

9h

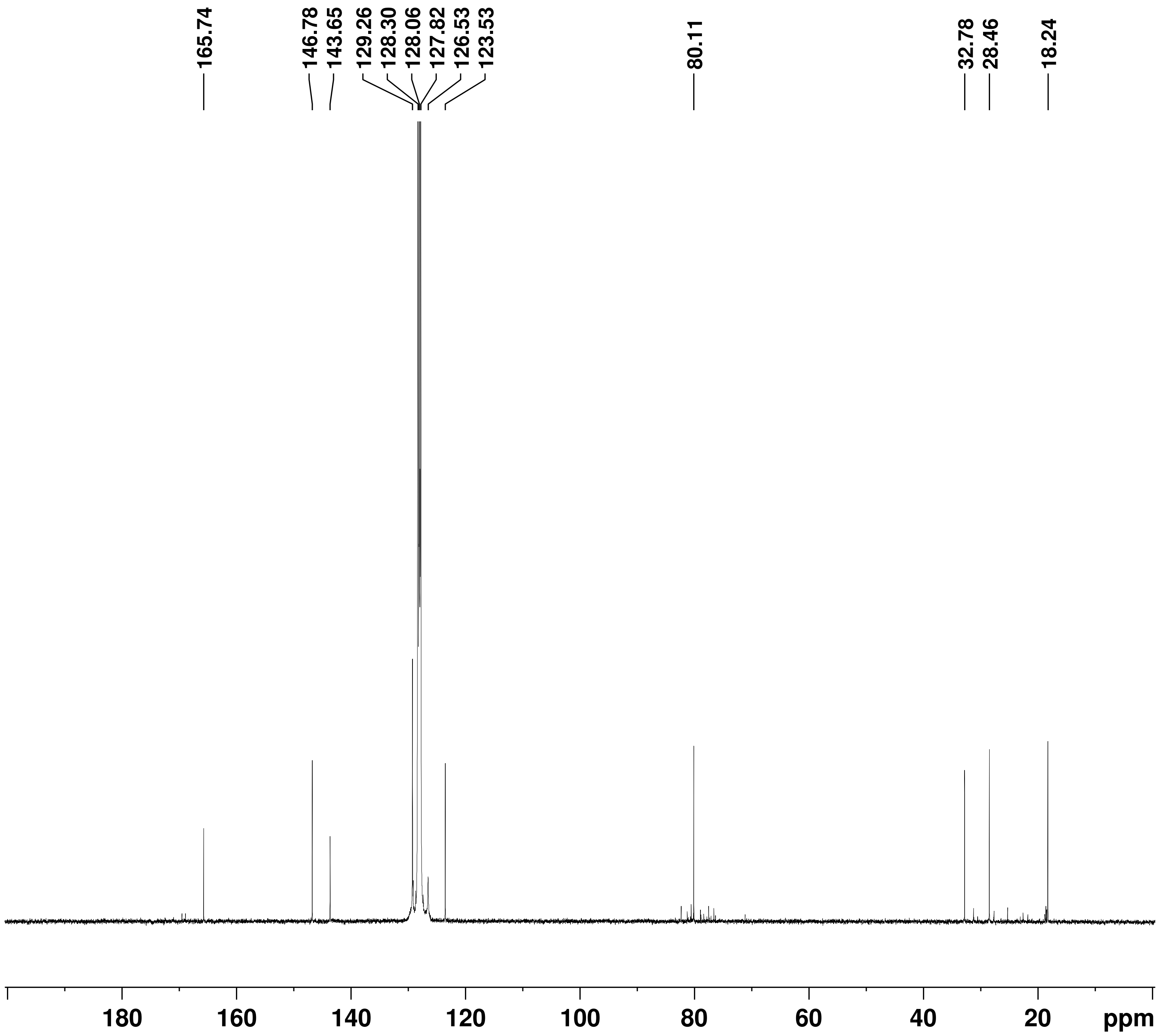

^{13}C spectrum of compound **9h**

400 MHz, C_6D_6

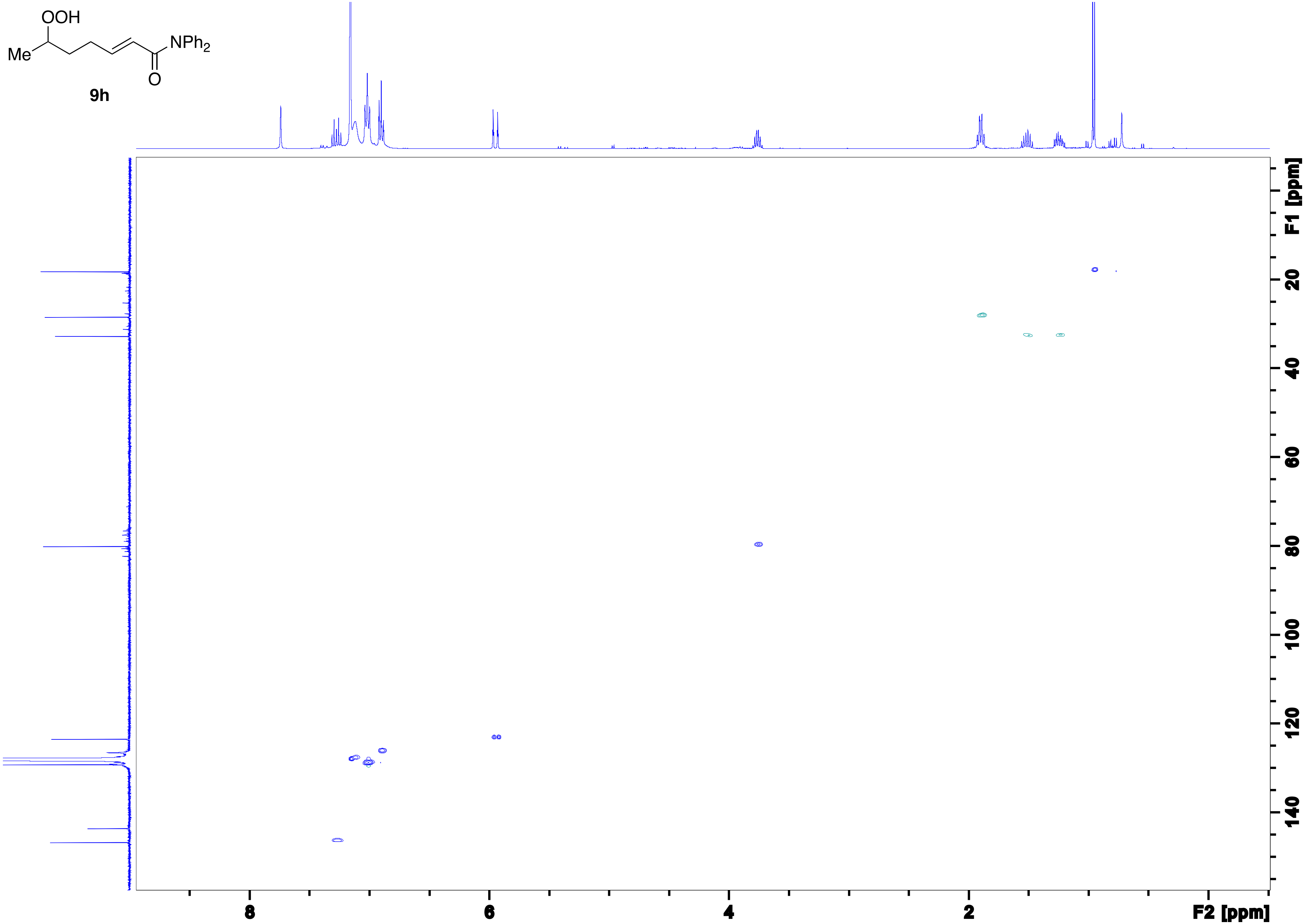

$^1H/^{13}C$ HSQC spectrum of compound **9h**

400 MHz, C_6D_6

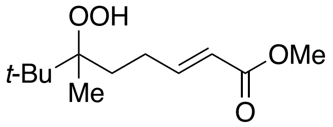

9i

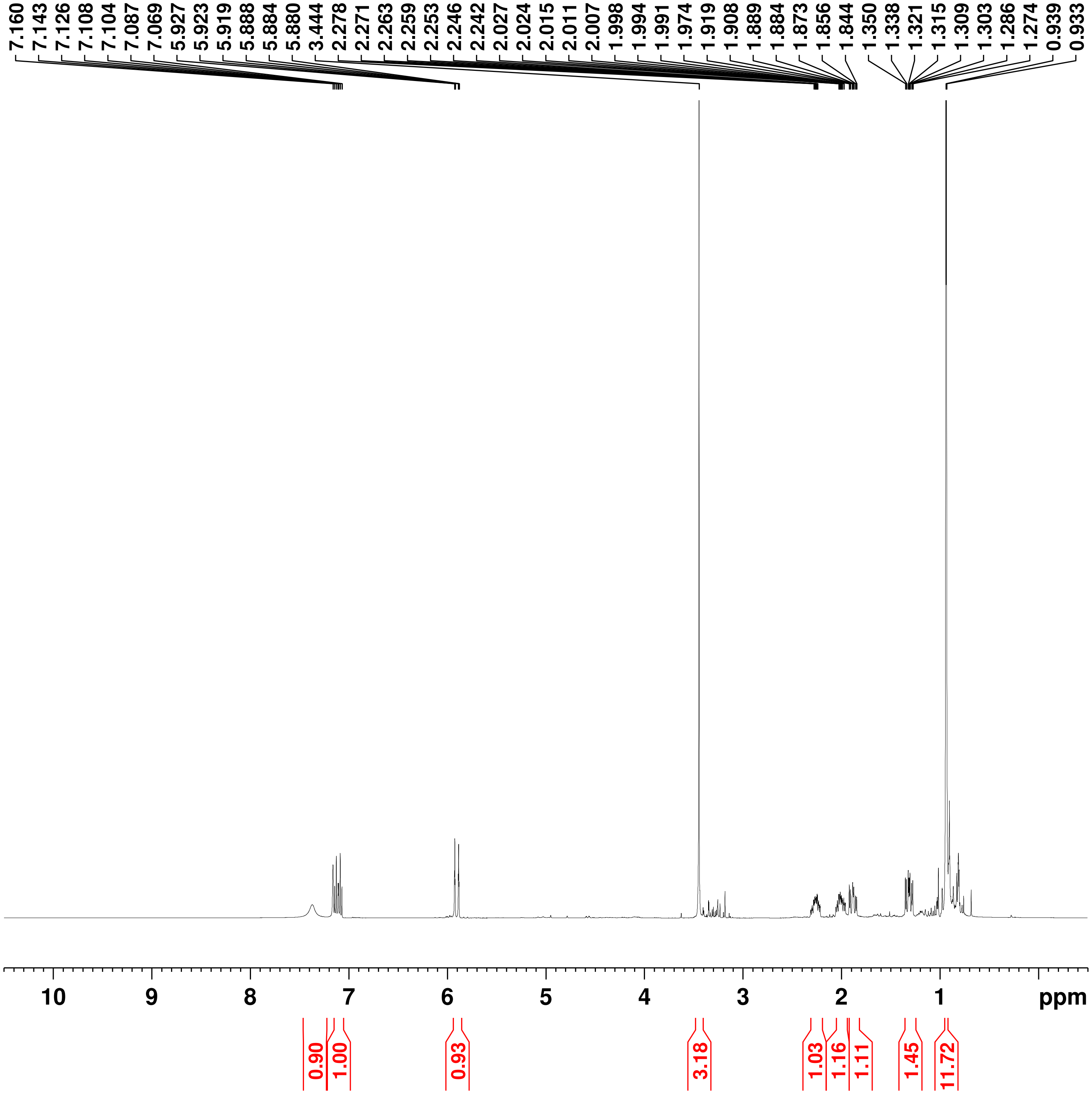

^{1}H spectrum of compound **9i**

100 MHz, C_6D_6

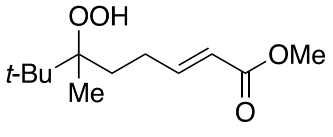

9i

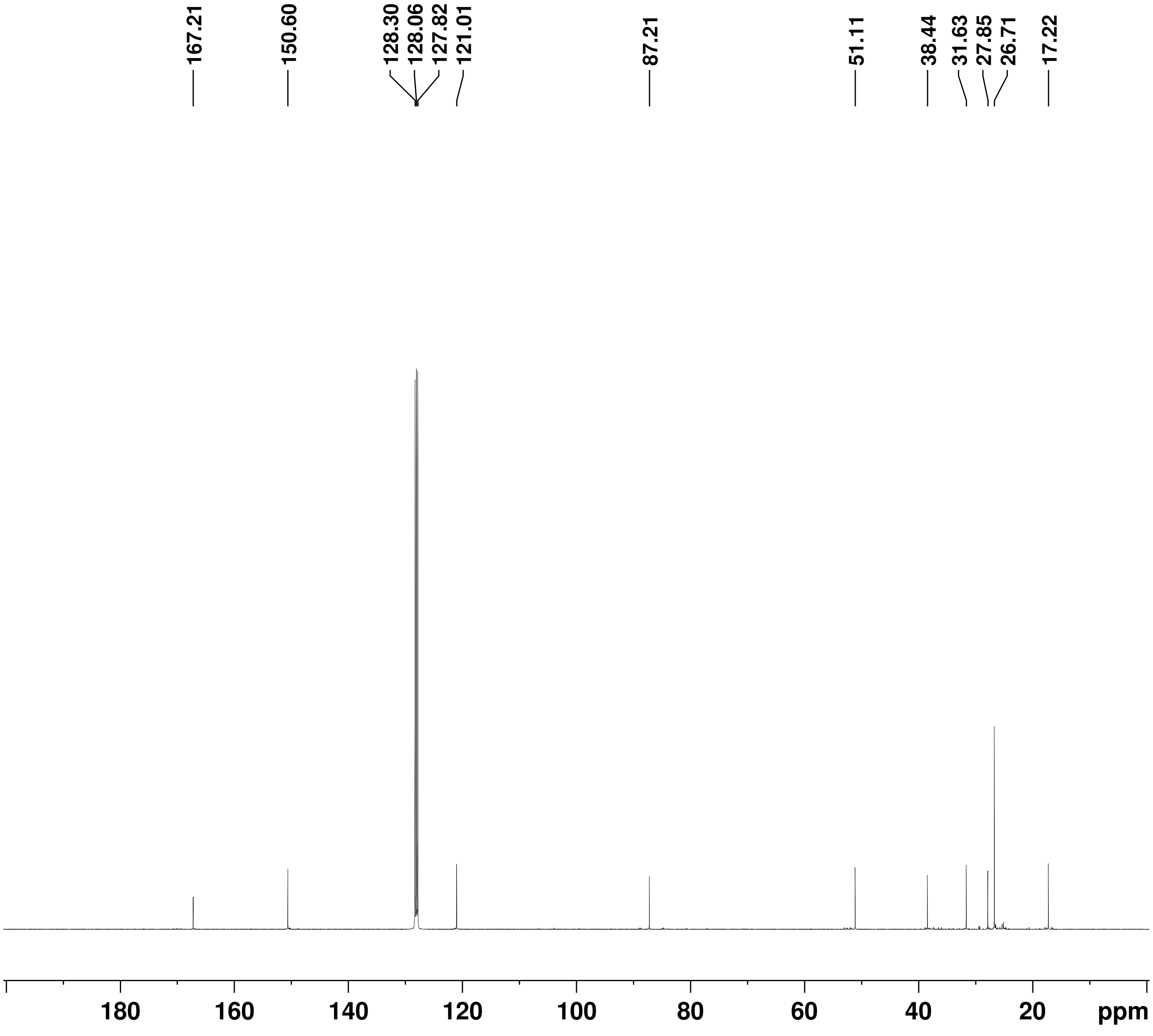

^{13}C spectrum of compound **9i**

400 MHz, C_6D_6

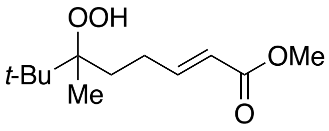

9i

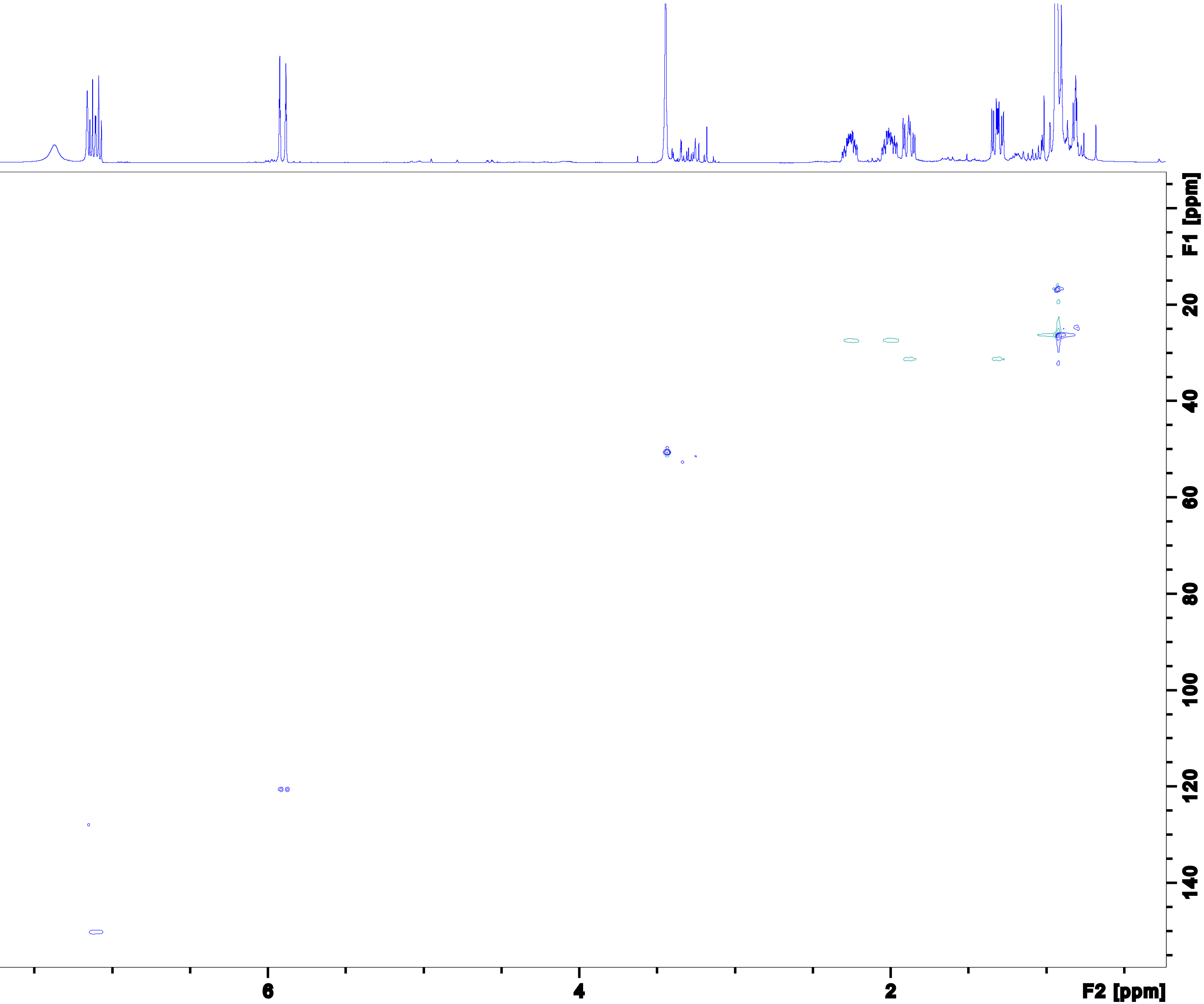

$^{1}H/^{13}C$ HSQC spectrum of compound **9i**

400 MHz, C_6D_6

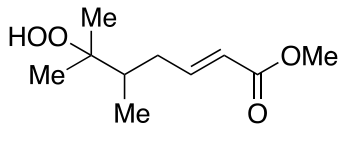

9j

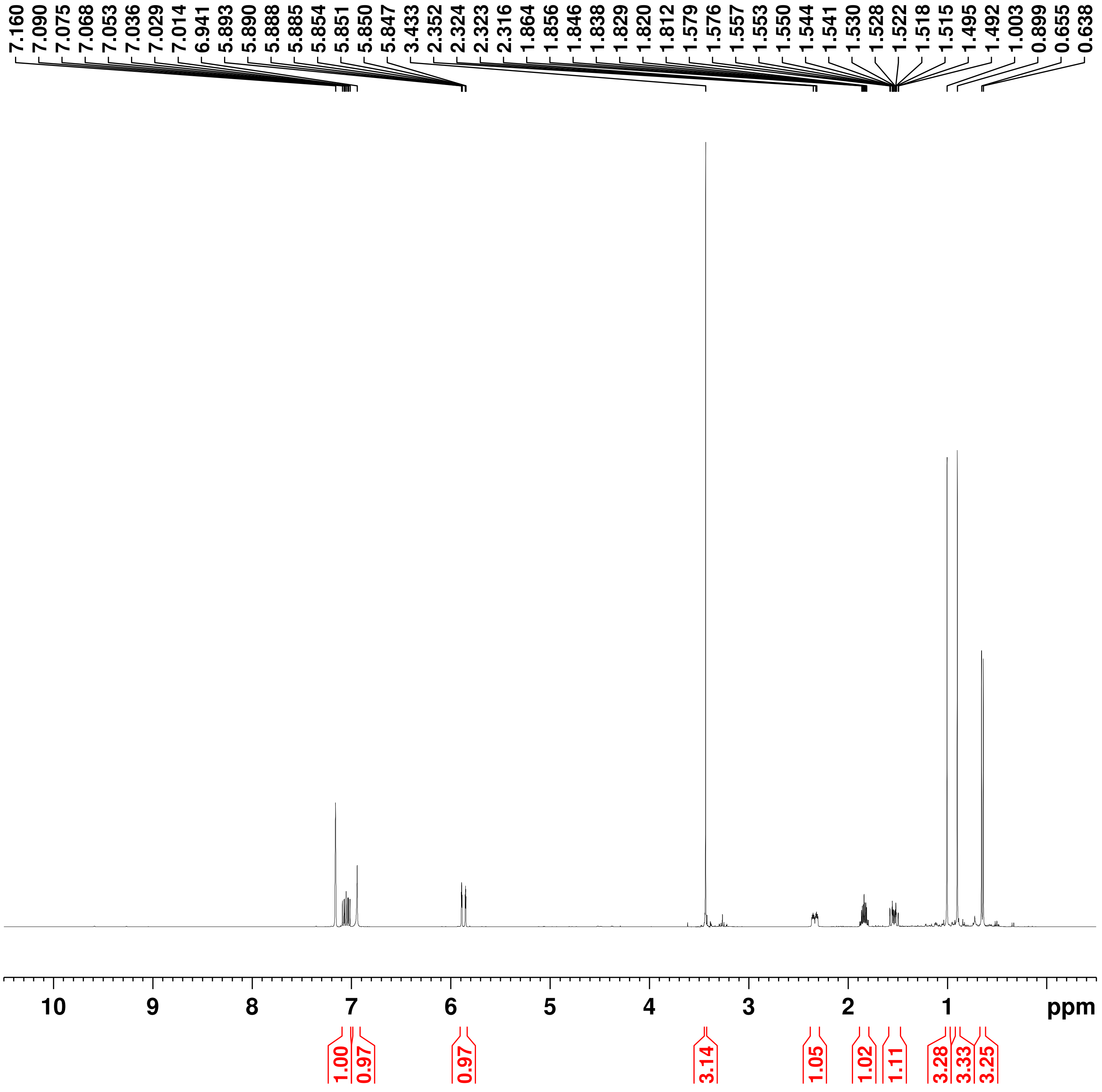

^{1}H spectrum of compound **9j**

100 MHz, C_6D_6

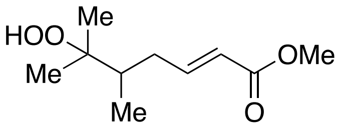

9j

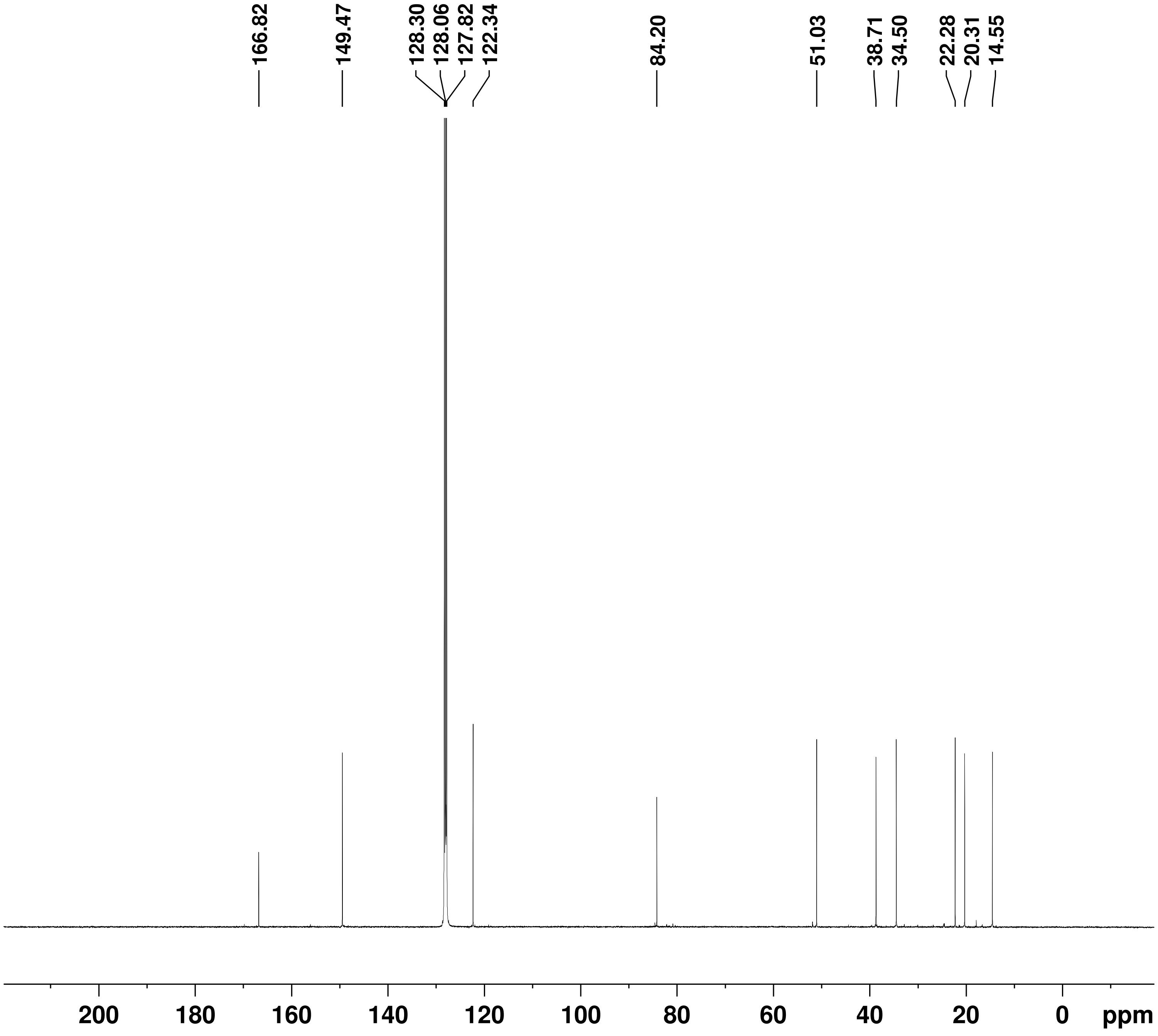

^{13}C spectrum of compound **9j**

400 MHz, C_6D_6

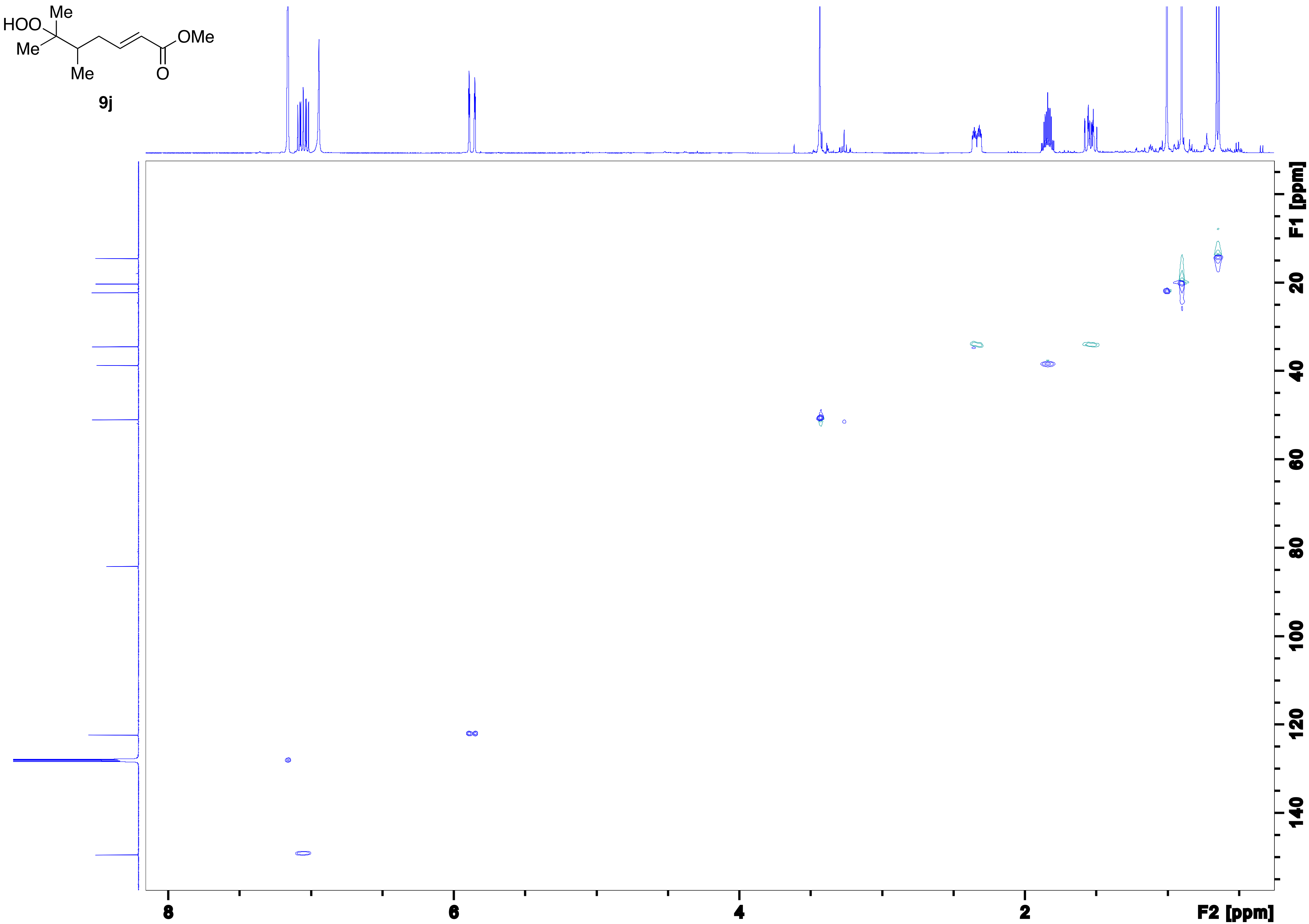

$^{1}H/^{13}C$ HSQC spectrum of compound **9j**

400 MHz, C_6D_6

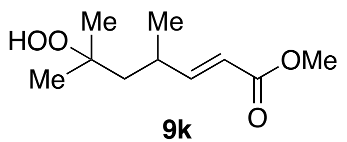

9k

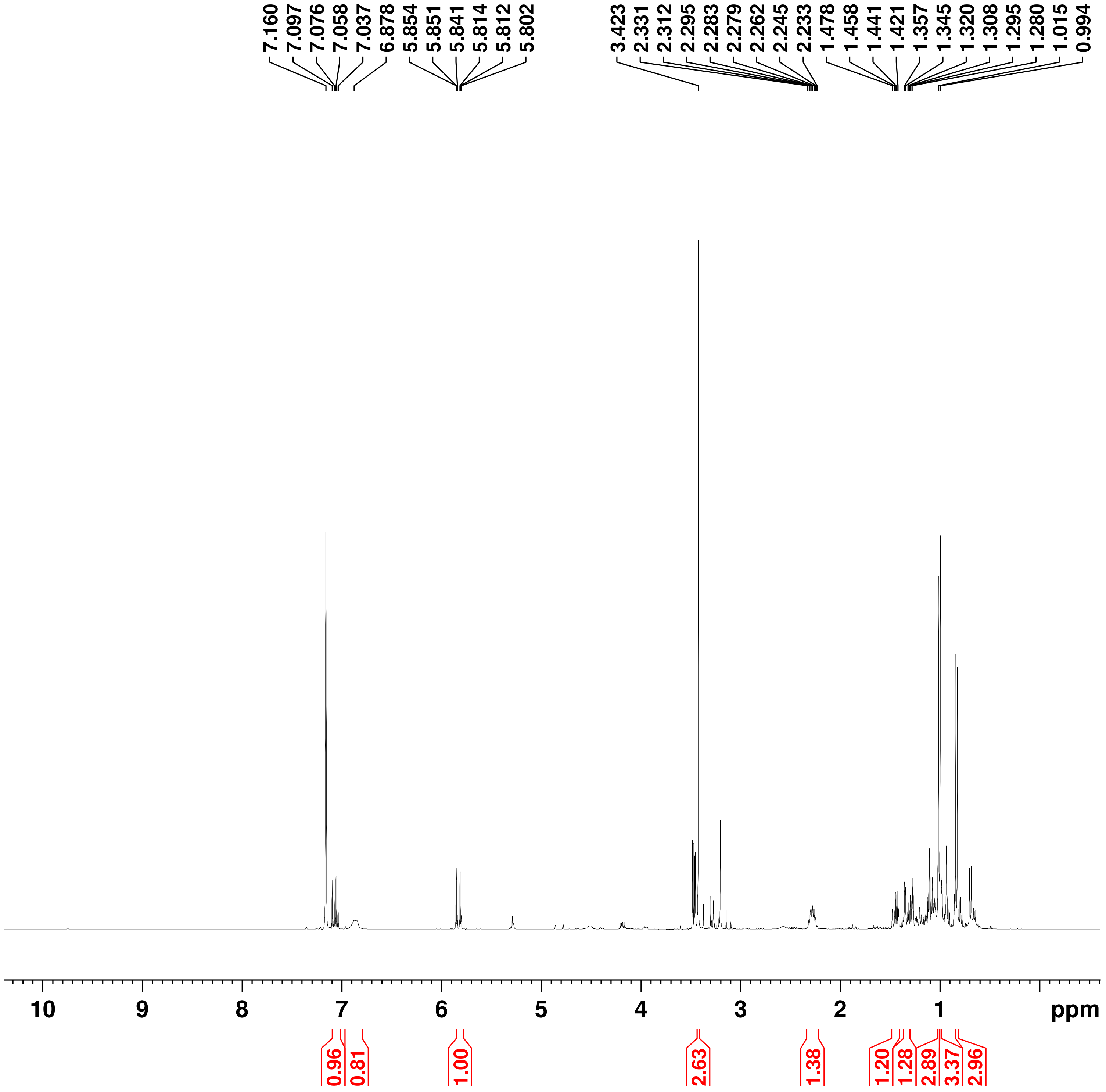

^{1}H spectrum of compound **9k**

100 MHz, C_6D_6

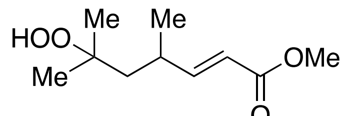

9k

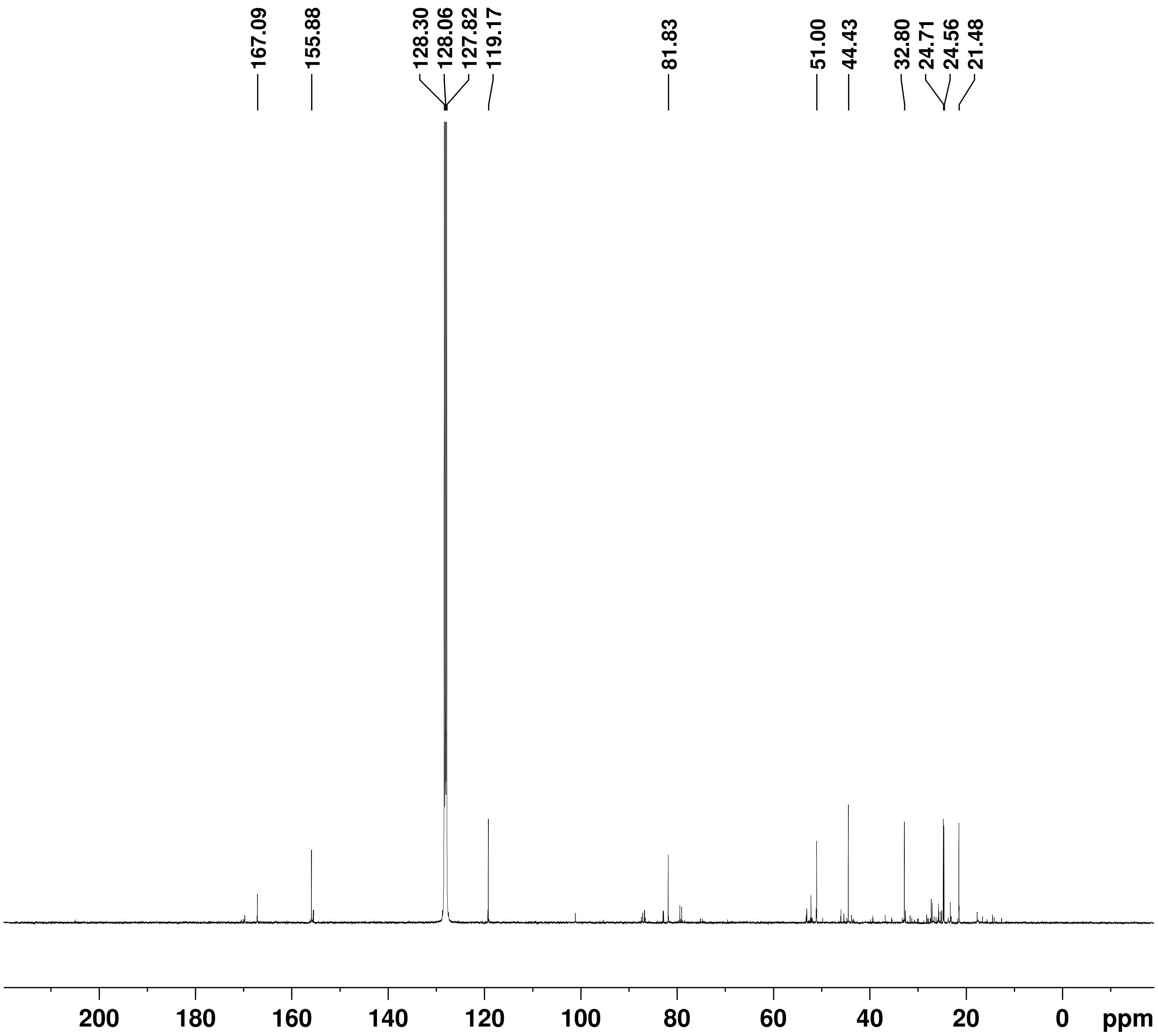

^{13}C spectrum of compound **9k**

100 MHz, C_6D_6

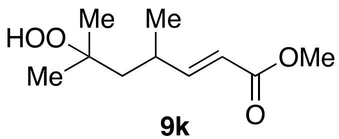

9k

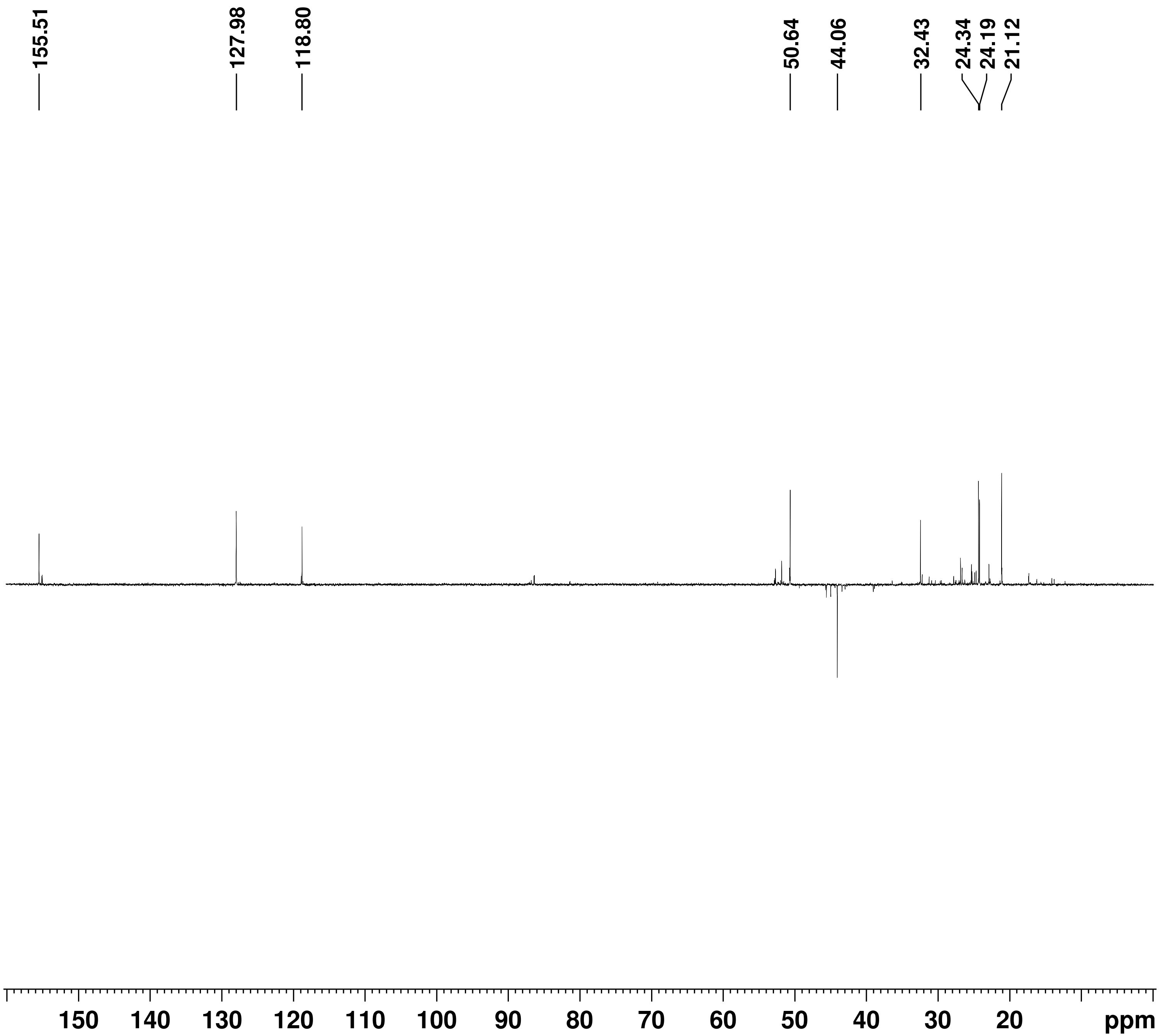

^{13}C DEPT spectrum of compound **9k**

400 MHz, $CDCl_3$

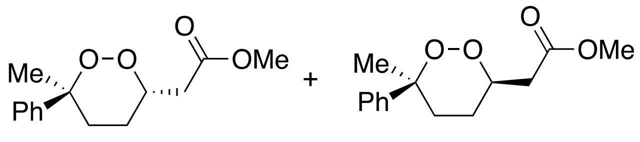

16a **16a’**

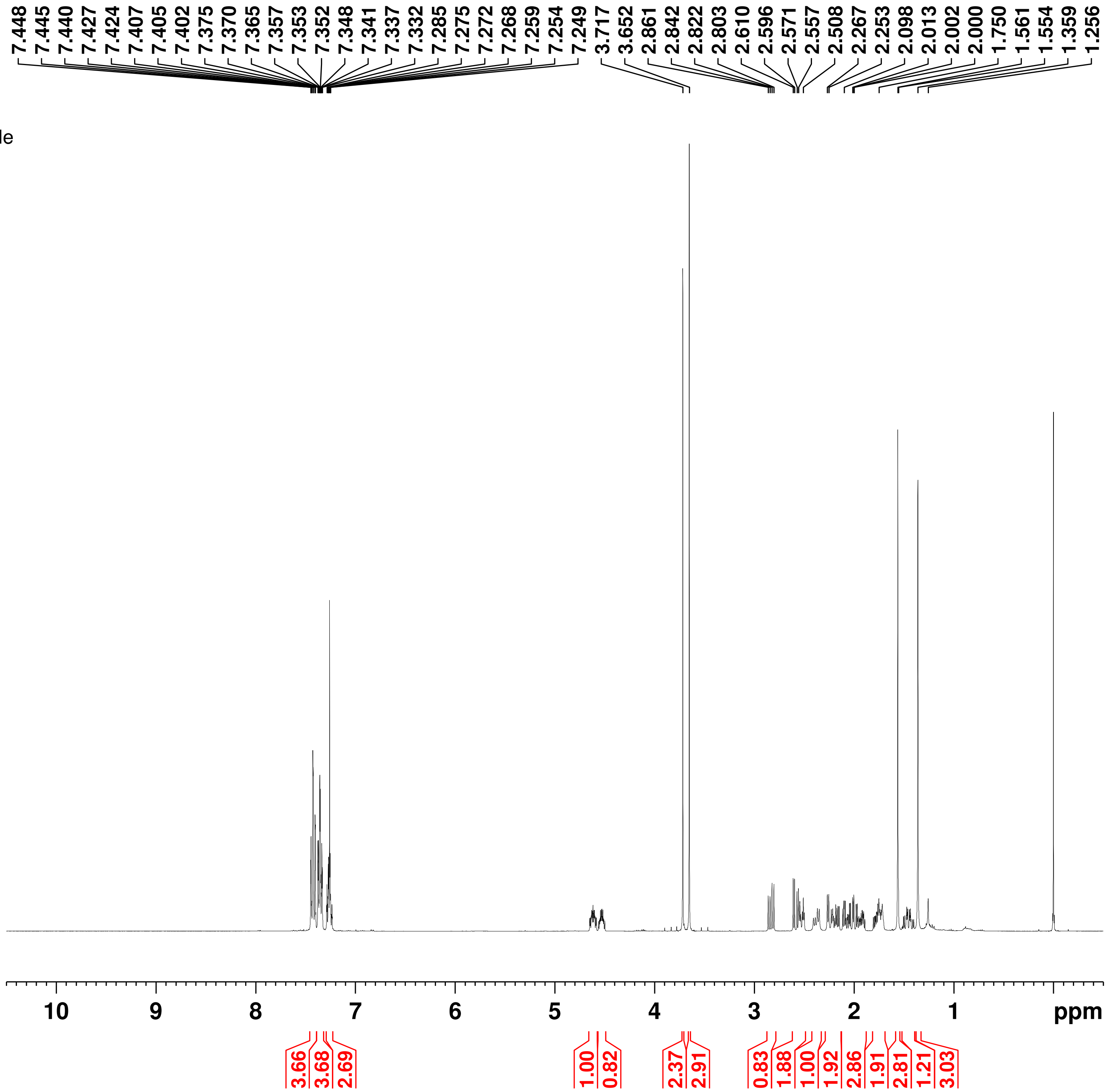

^{1}H spectrum of compounds **16a & 16a'**

100 MHz, $CDCl_3$

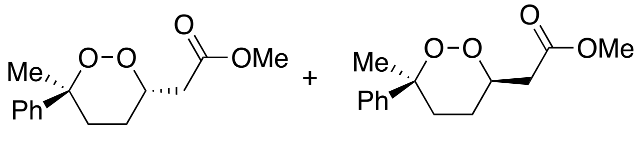

16a + 16a’

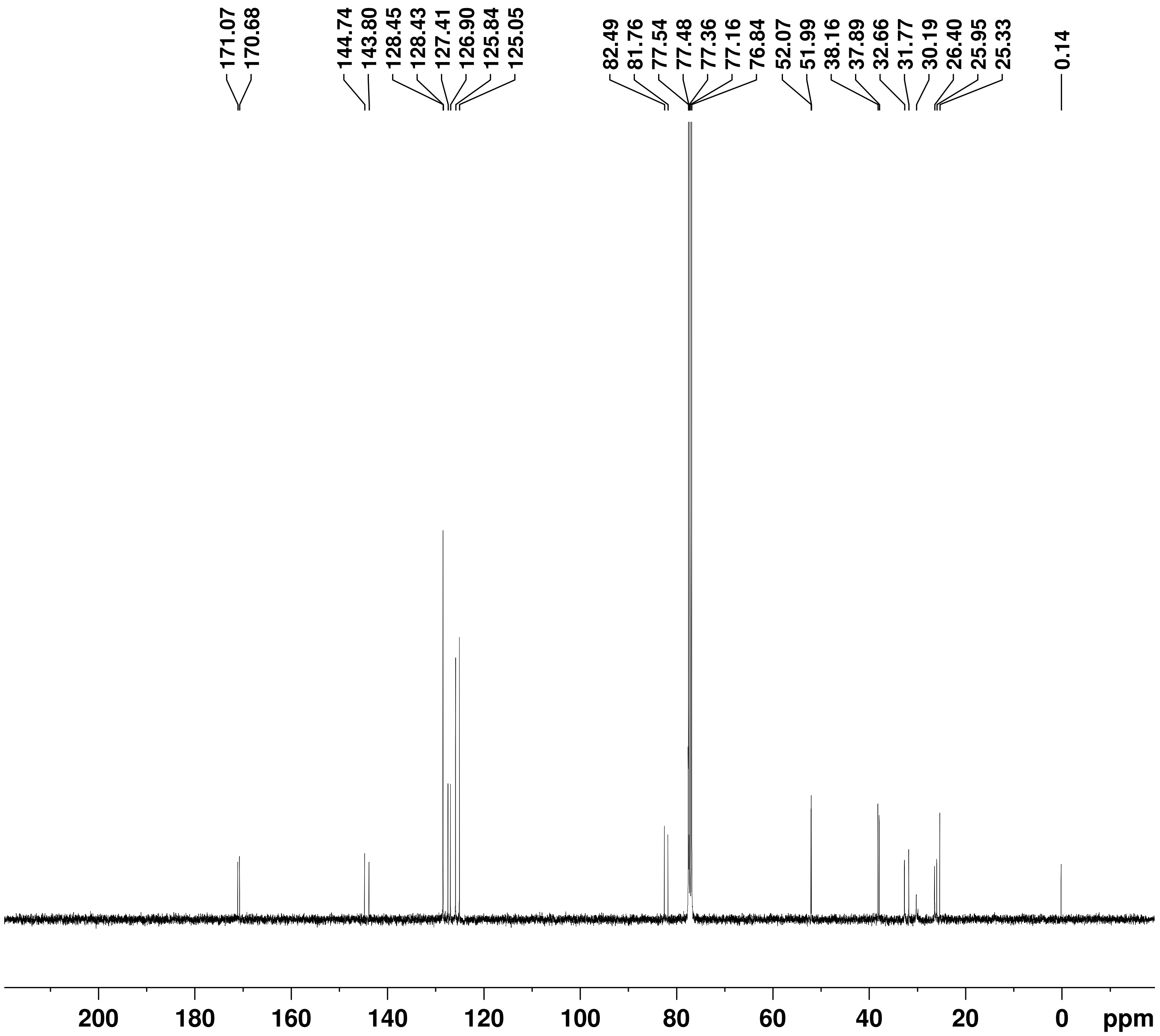

^{13}C spectrum of compound **16a & 16a'**

400 MHz, $CDCl_3$

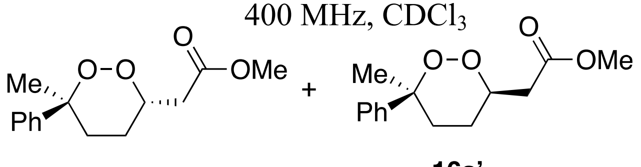

16a + 16a'

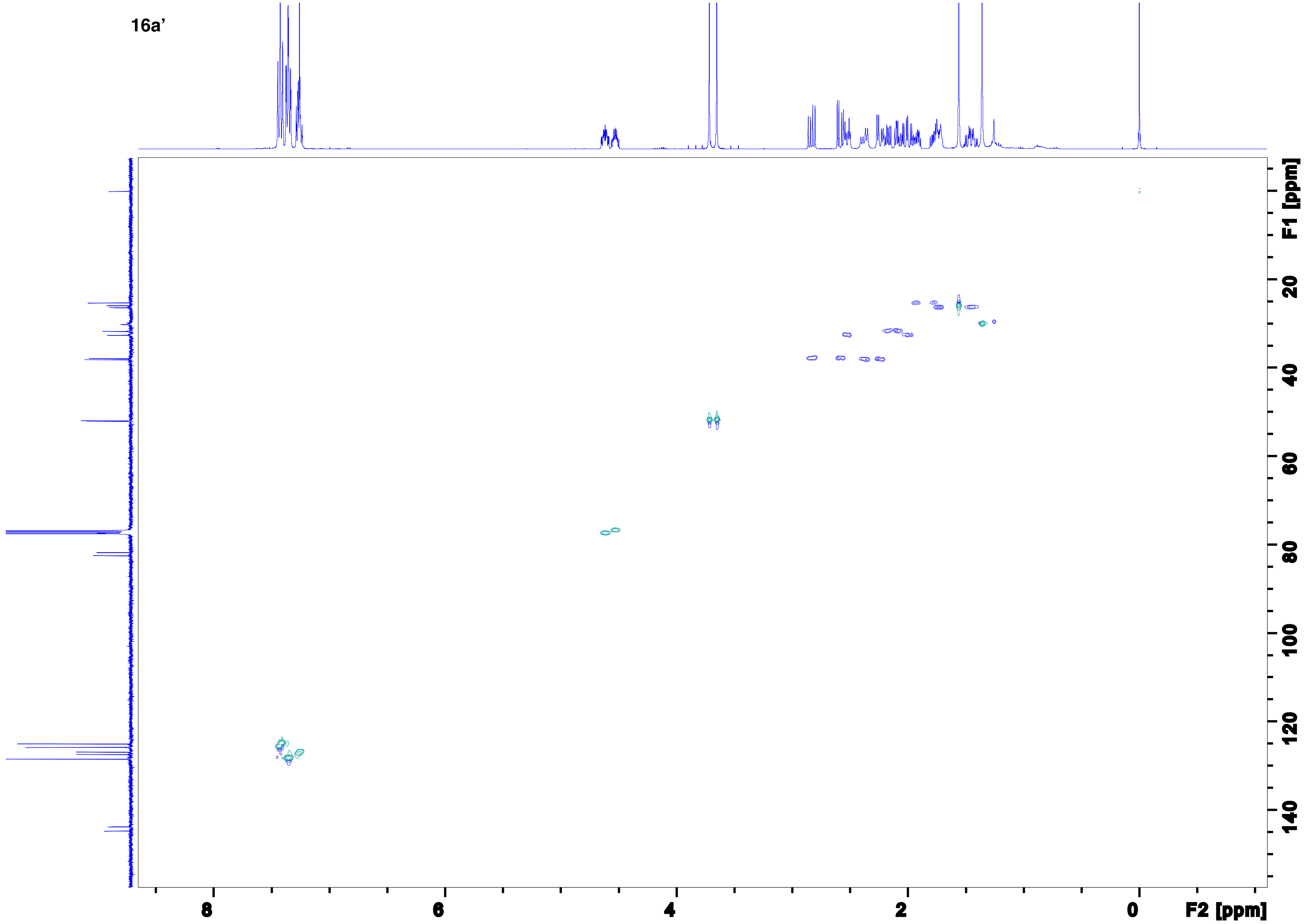

$^1H/^{13}C$ HSQC spectrum of compounds **16a & 16a'**

400 MHz, $CDCl_3$

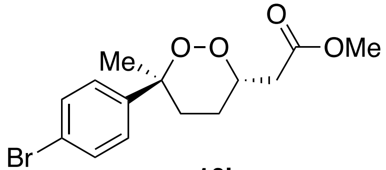

16b

+

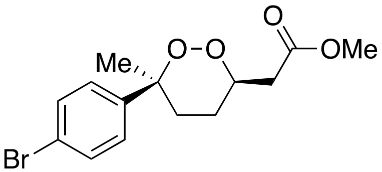

16b’

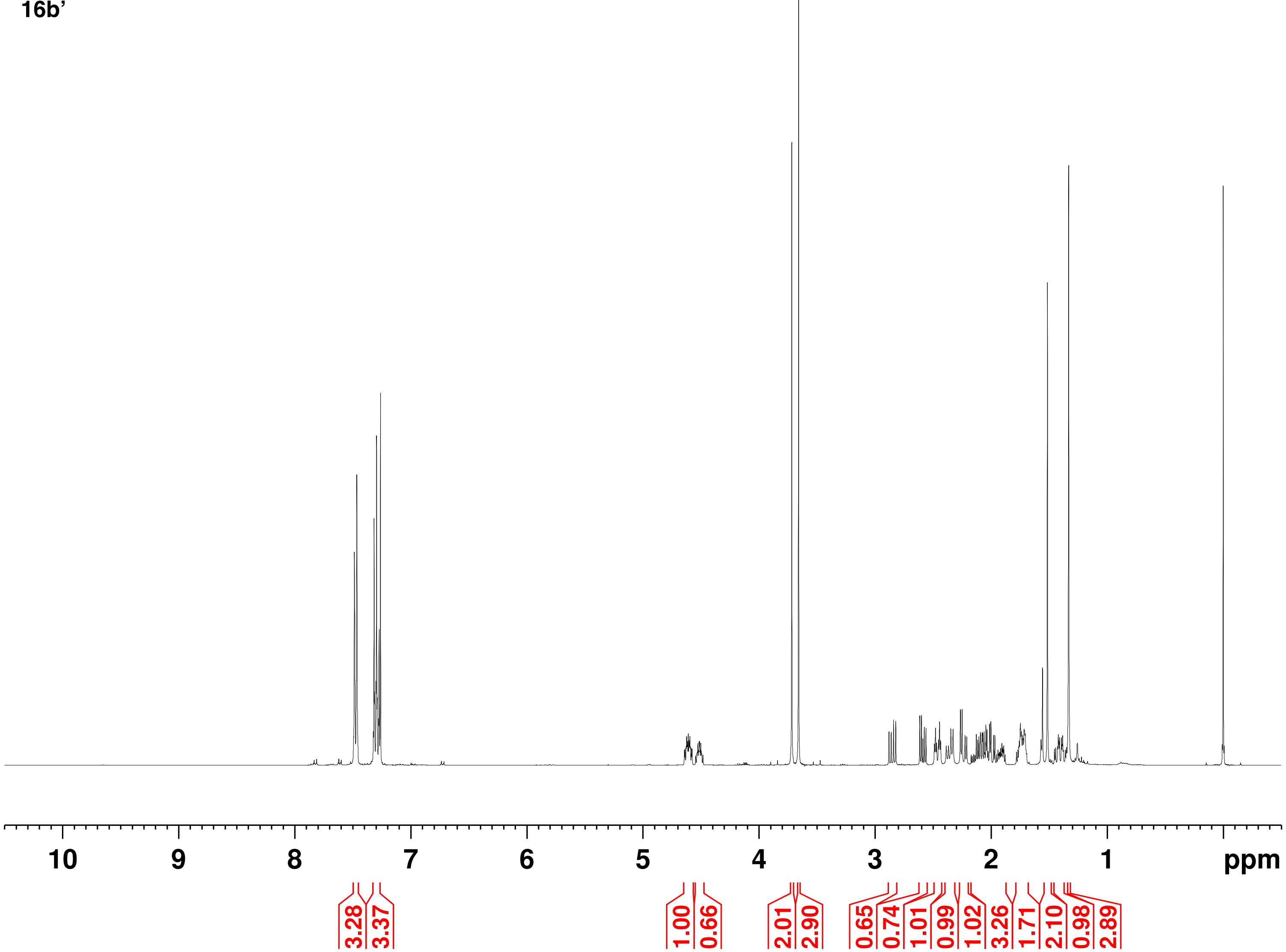

^{1}H spectrum of compounds **16b & 16b'**

100 MHz, $CDCl_3$

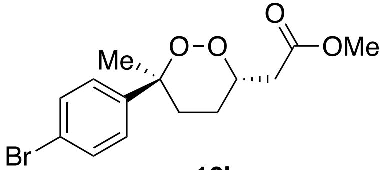

16b

+

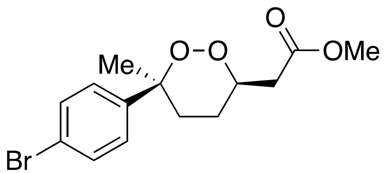

16b’

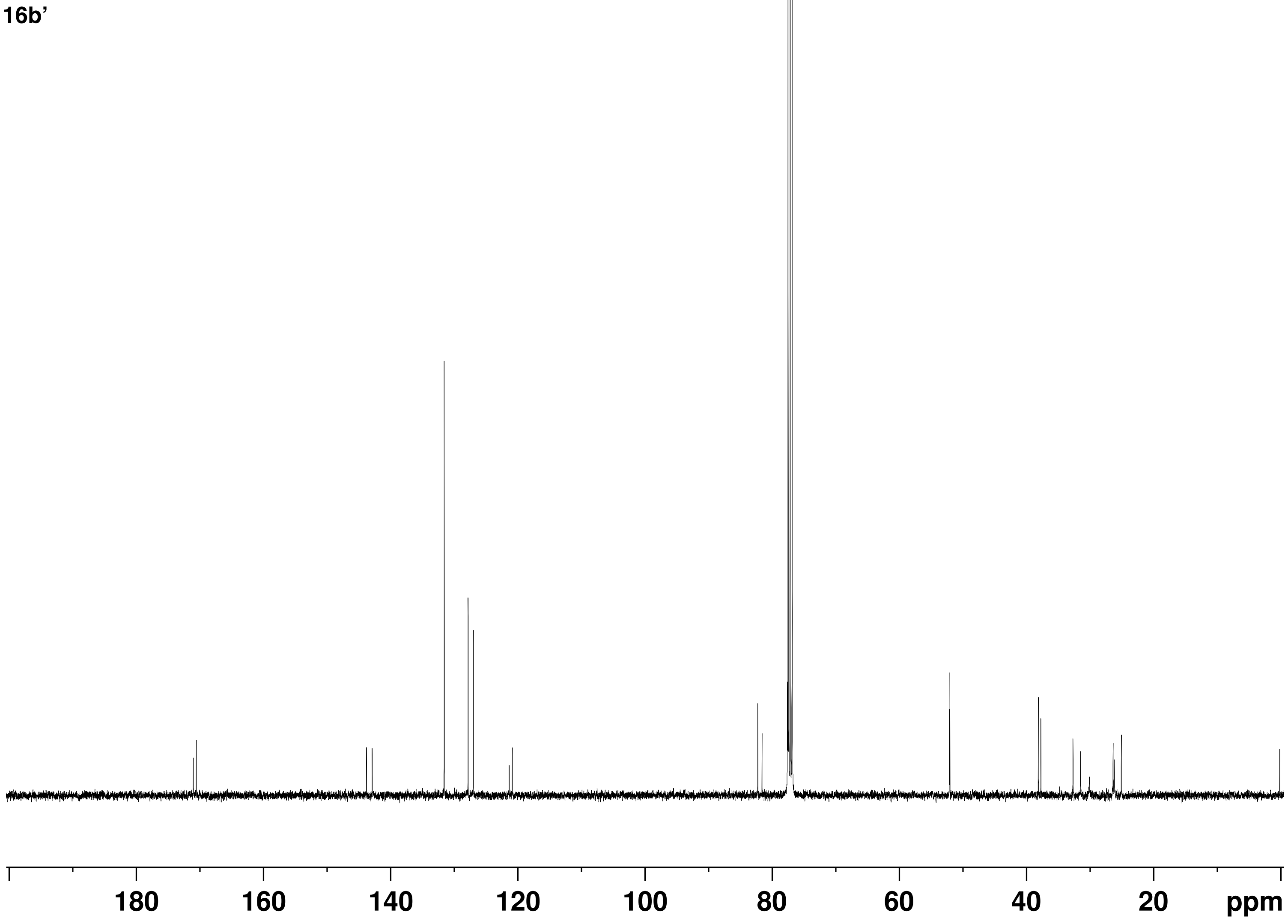

^{13}C spectrum of compounds **16b & 16b'**

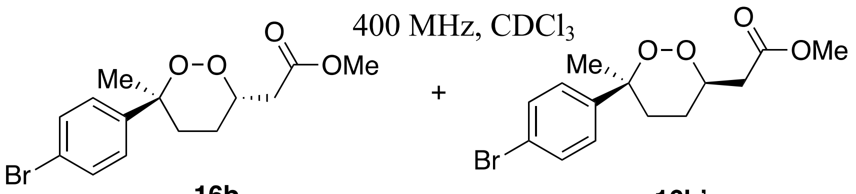

400 MHz, $CDCl_3$

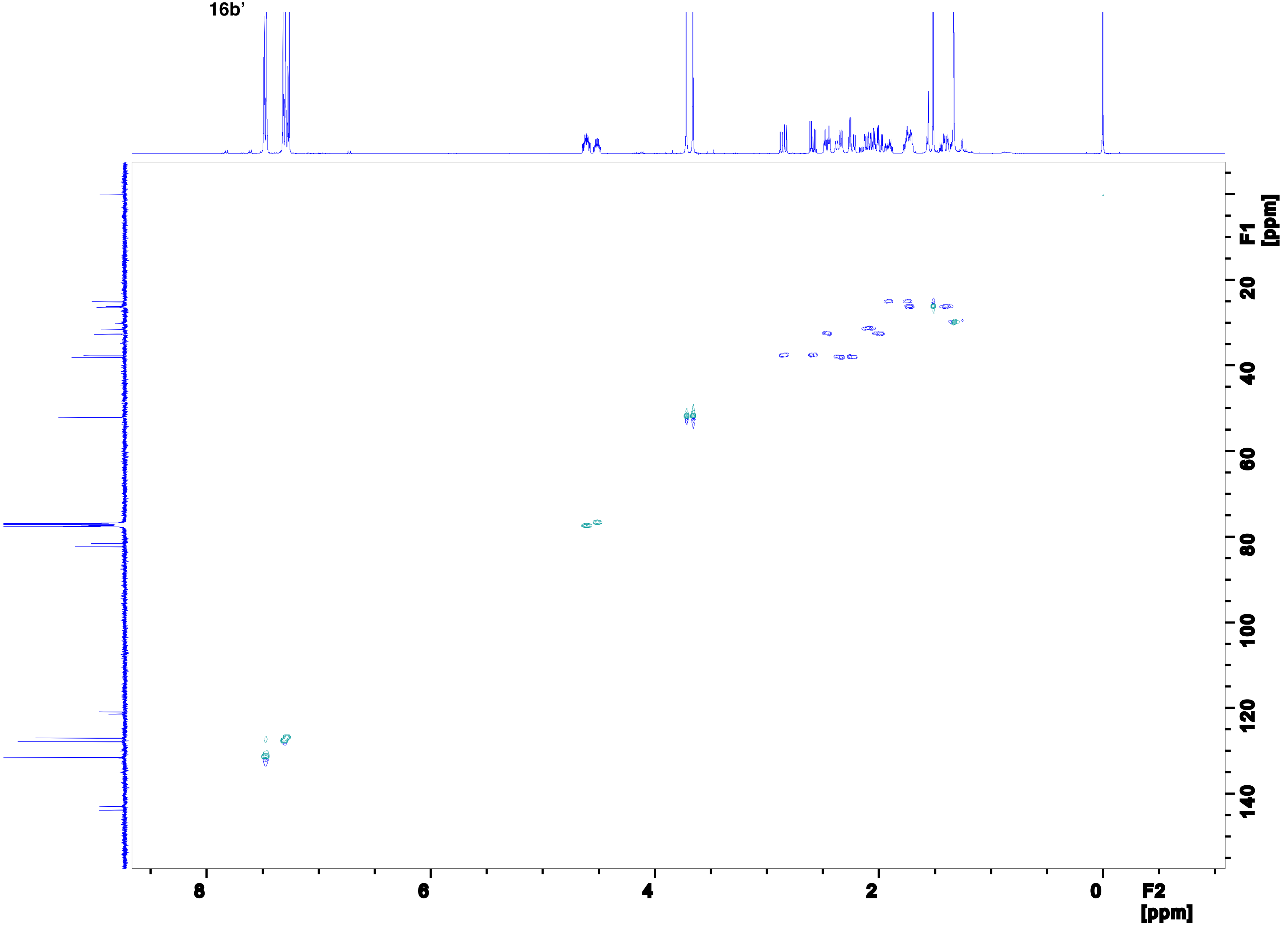

$^{1}H/^{13}C$ HSQC spectrum of compounds **16b & 16b'**

400 MHz, $CDCl_3$

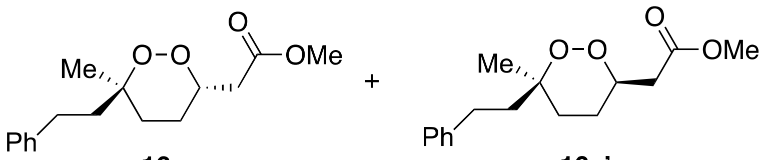

16c **16c'**

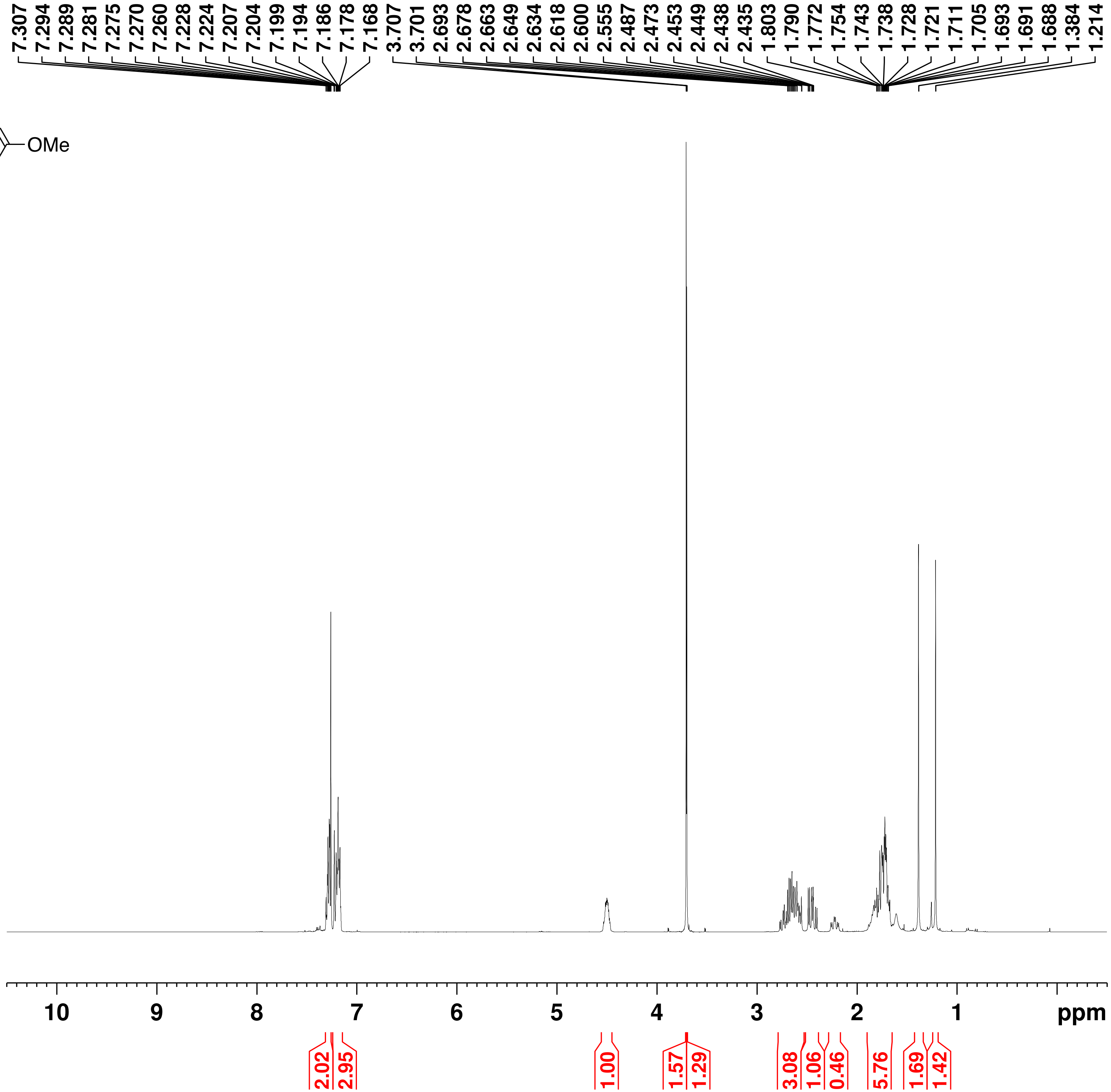

^{1}H spectrum of compounds **16c & 16c'**

100 MHz, $CDCl_3$

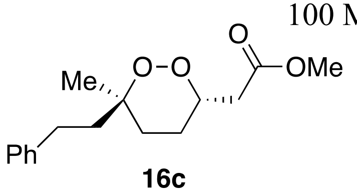

16c

+

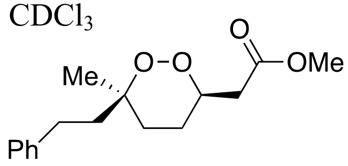

16c'

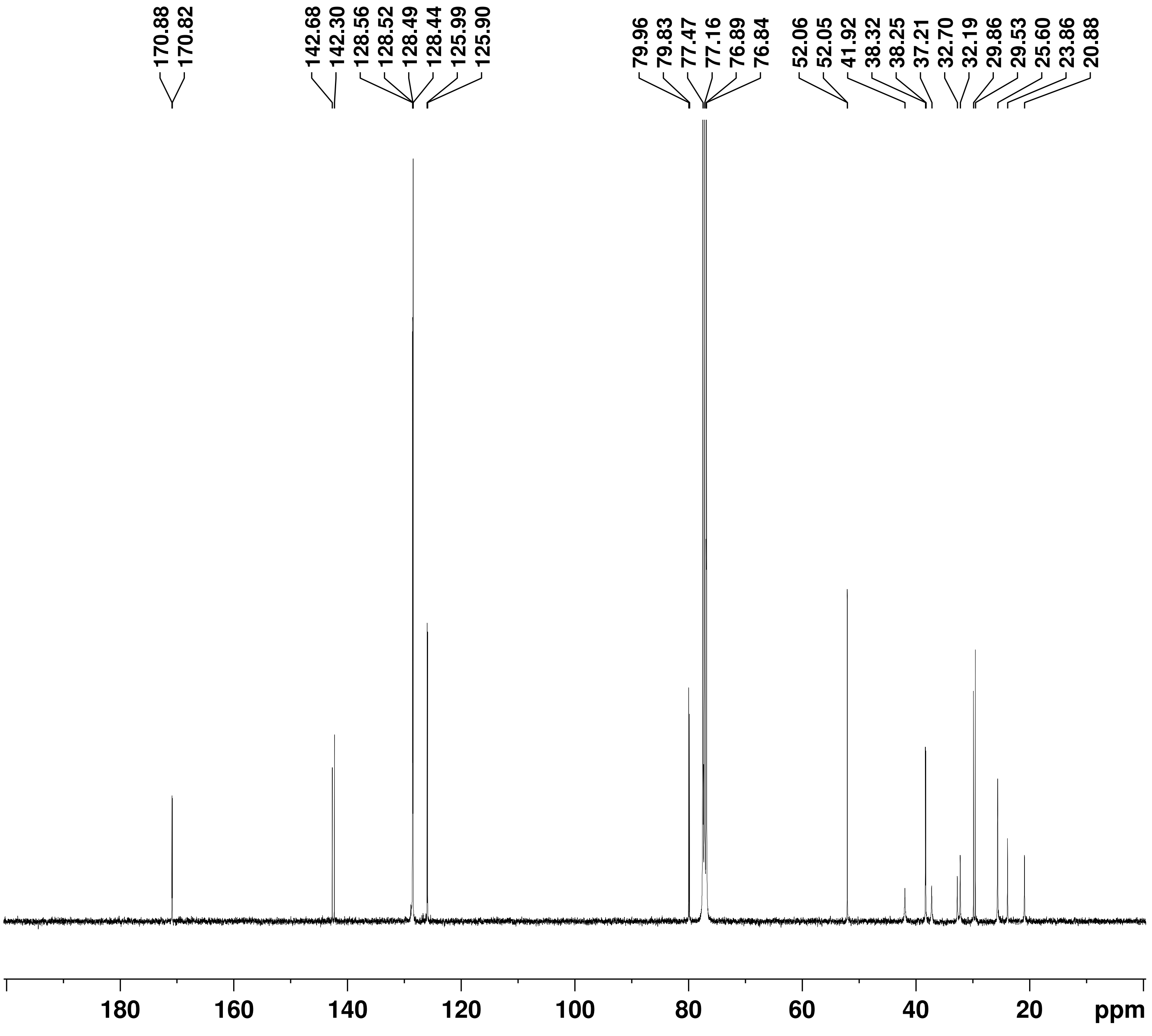

^{13}C spectrum of compounds **16c & 16c'**

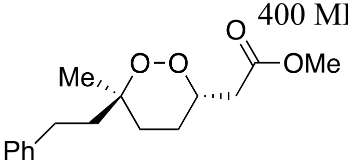

16c

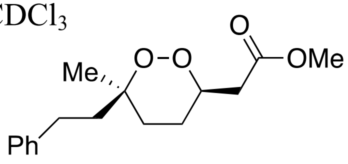

16c’

400 MHz, $CDCl_3$

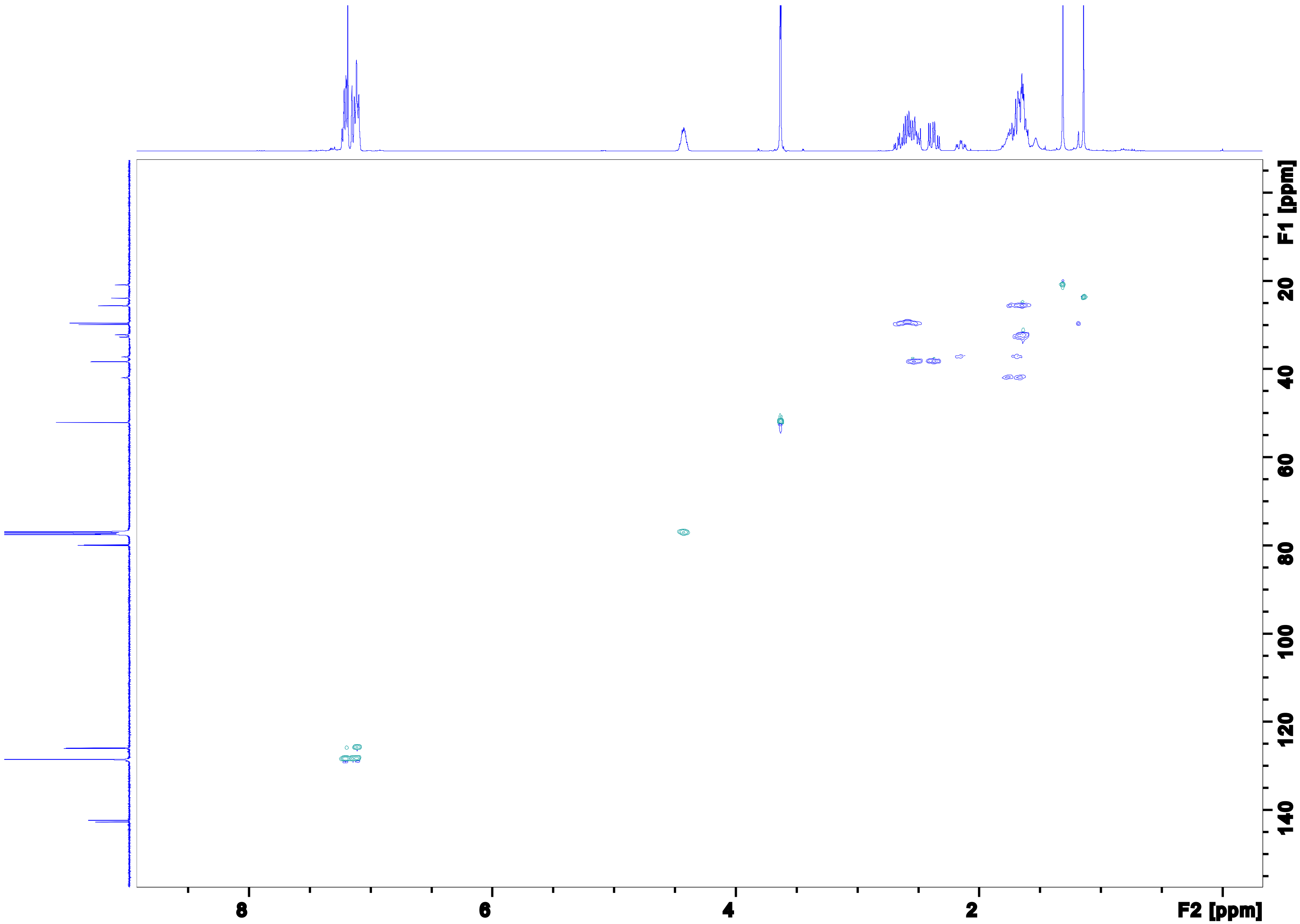

$^{1}H/^{13}C$ HSQC spectrum of compounds **16c & 16c'**

400 MHz, $CDCl_3$

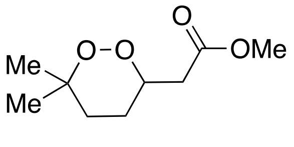

16d

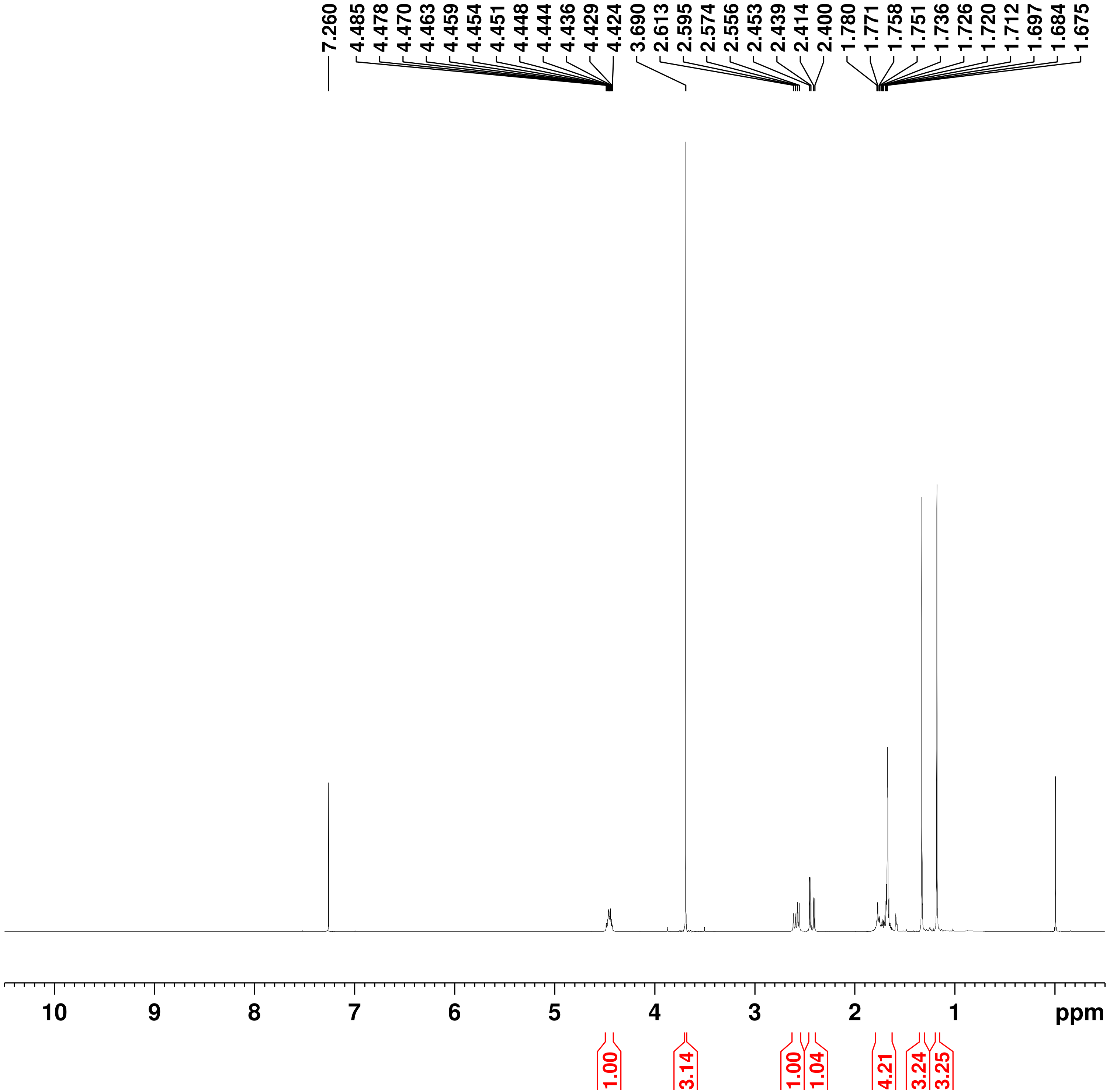

^1H spectrum of compound **16d**

100 MHz, C_6D_6

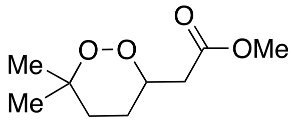

16d

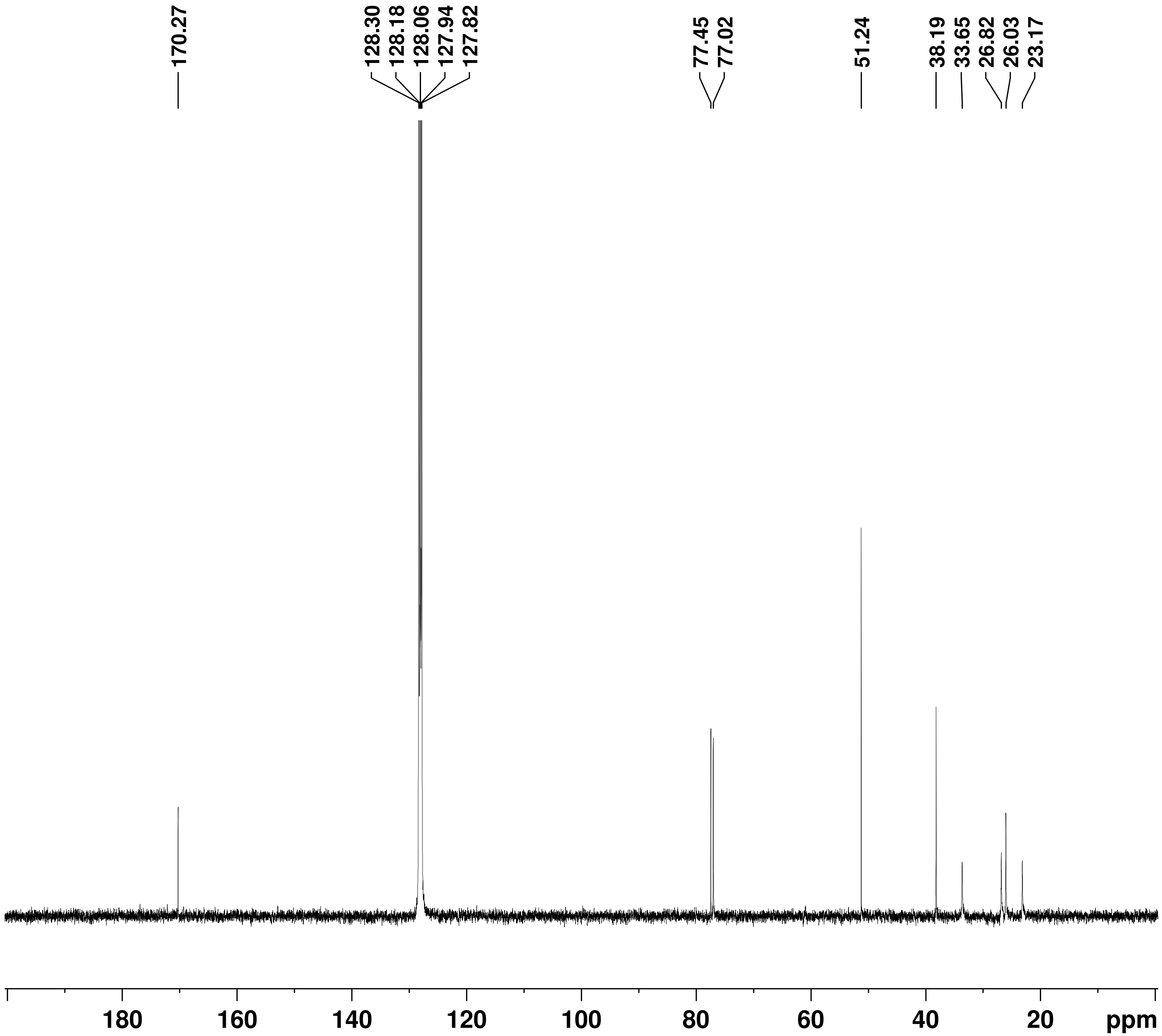

^{13}C spectrum of compound **16d**

400 MHz, $CDCl_3$

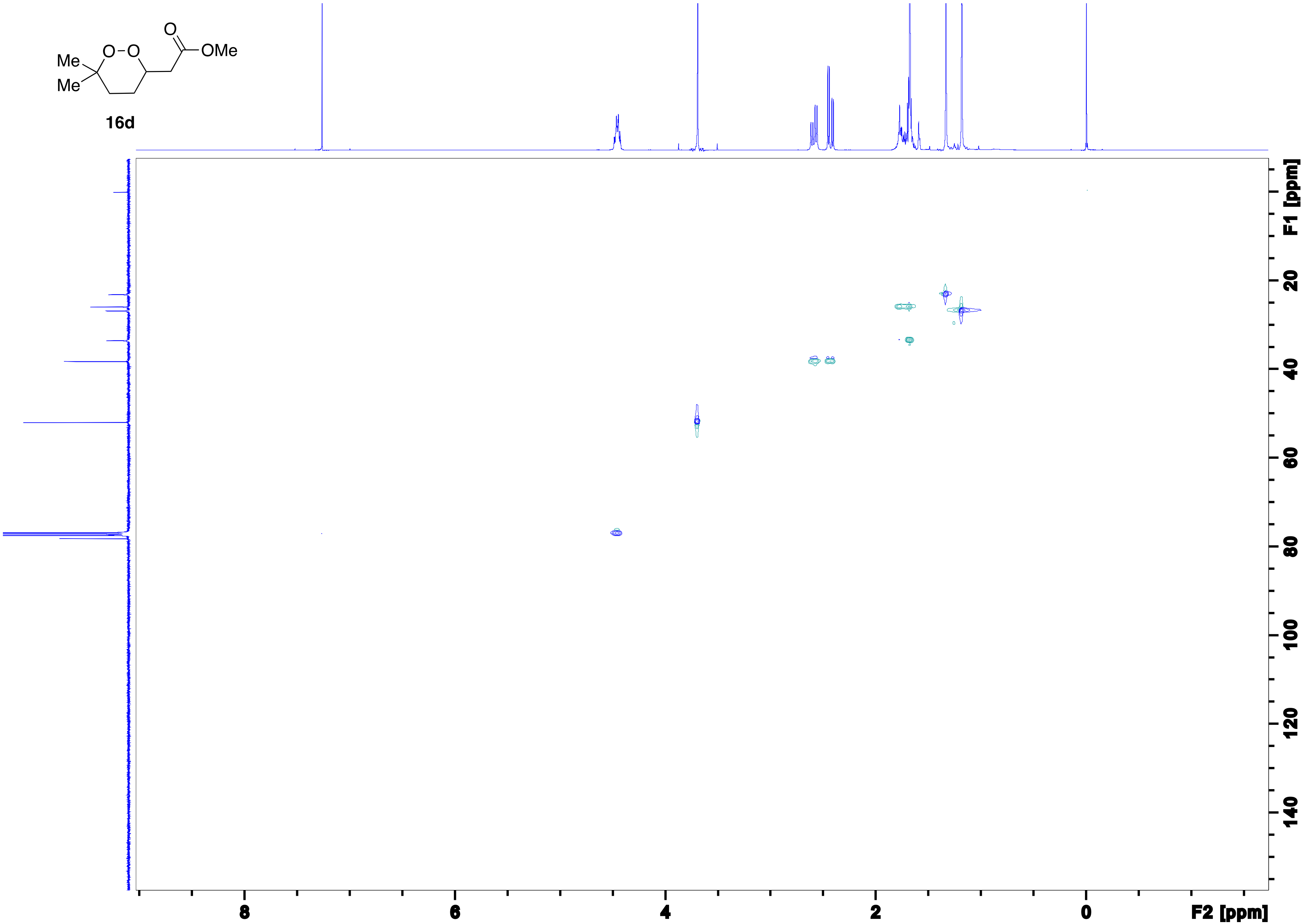

$^1H/^{13}C$ HSQC spectrum of compound **16d**

400 MHz, $(CD_3)_2SO$

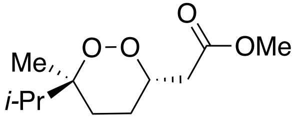

16e

+

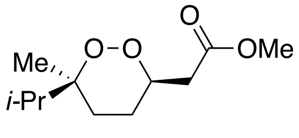

16e'

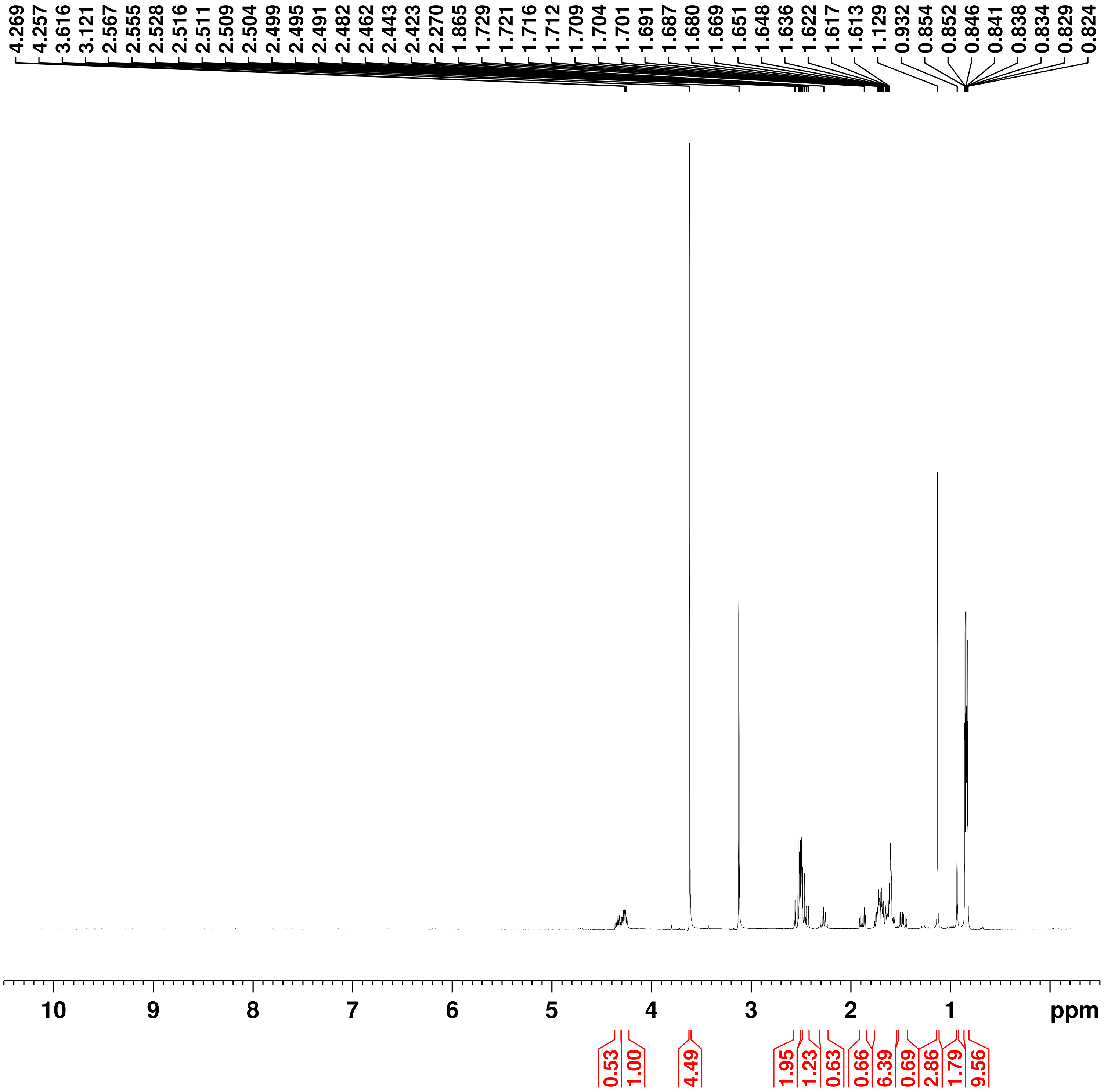

^1H spectrum of compounds **16e & 16e'**

400 MHz, $(CD_3)_2SO$

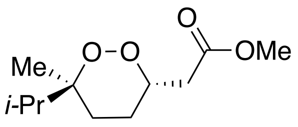

16e

+

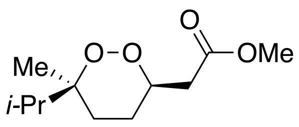

16e'

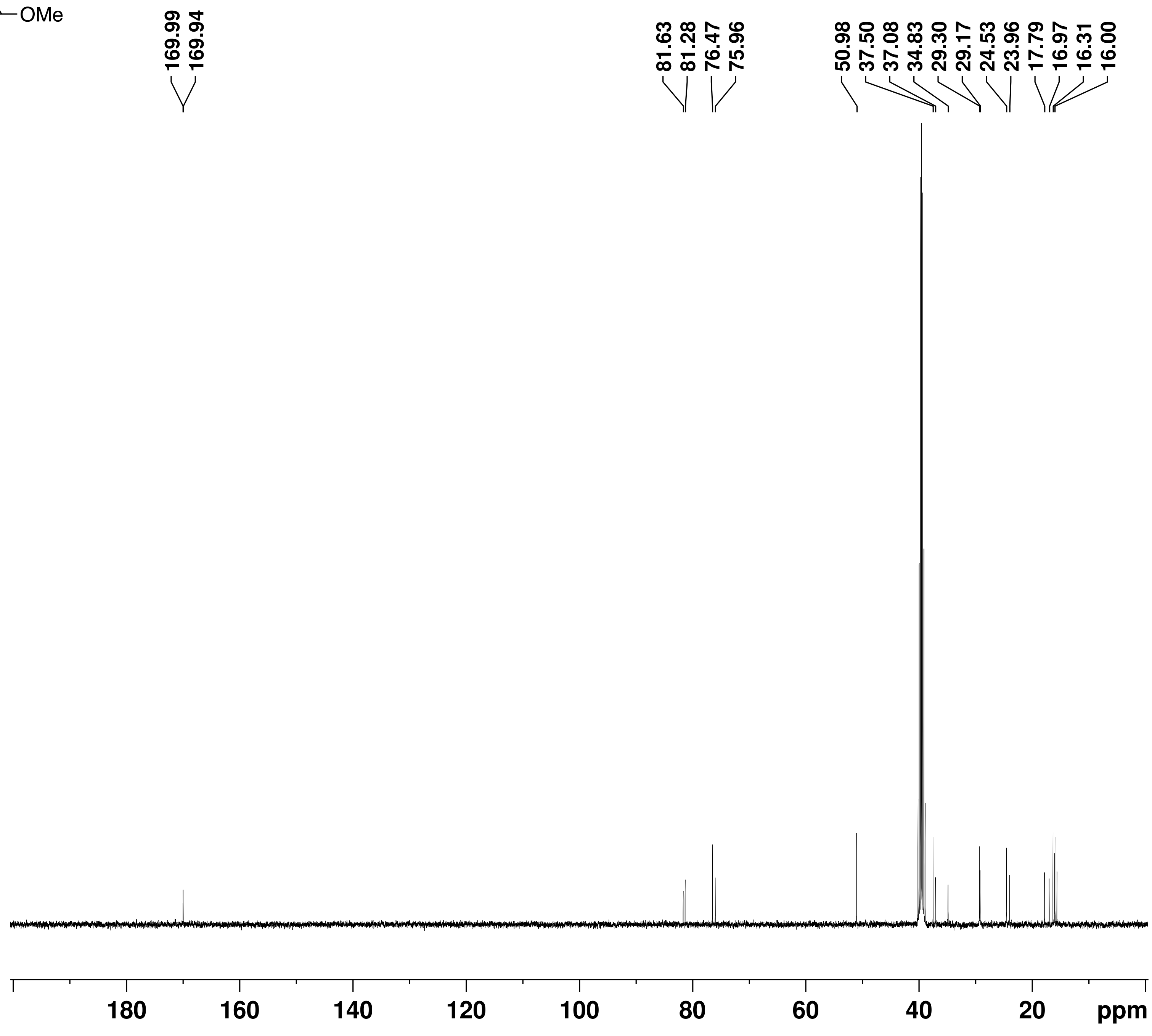

^{13}C spectrum of compounds **16e & 16e'**

400 MHz, $(CD_3)_2SO$

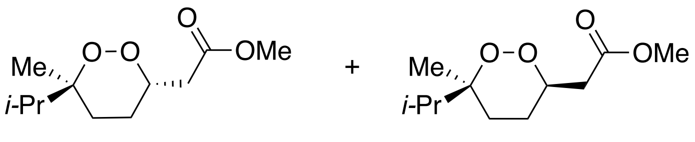

16e 16e’

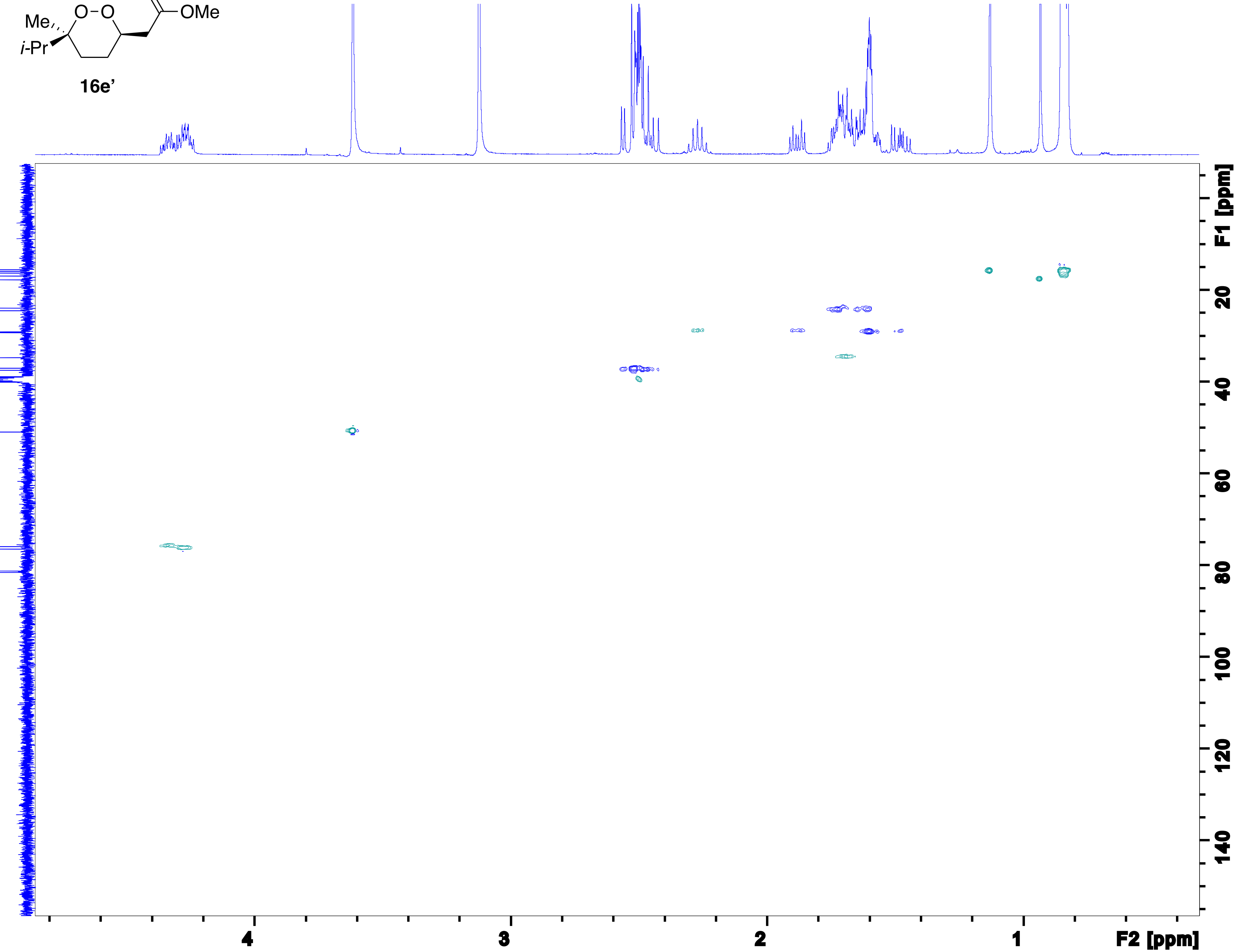

$^{1}H/^{13}C$ HSQC spectrum of compounds **16e & 16e'**

400 MHz, $CDCl_3$

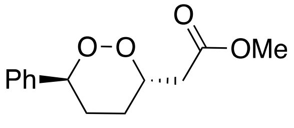

16f

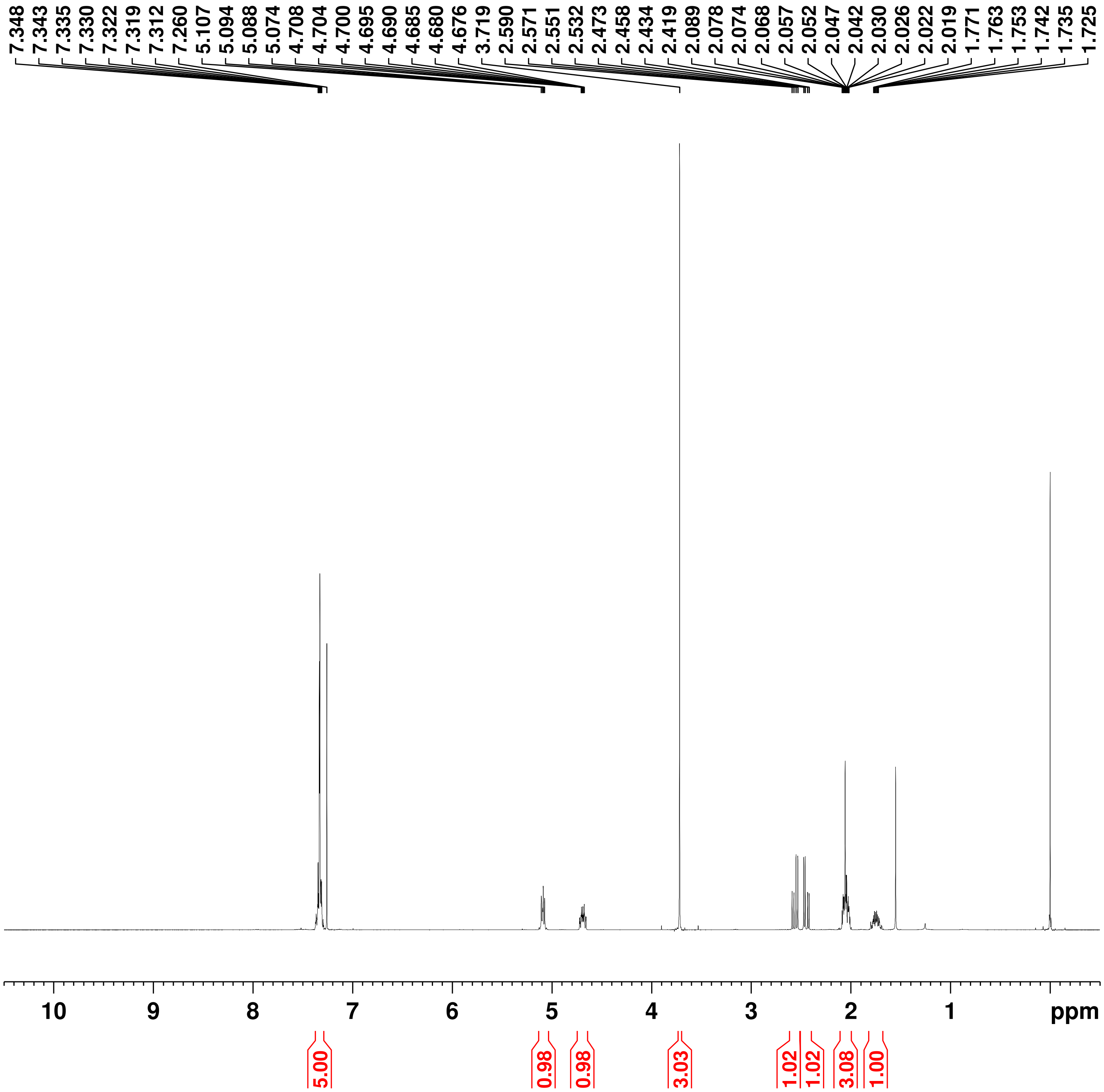

^1H spectrum of compound **16f**

100 MHz, $CDCl_3$

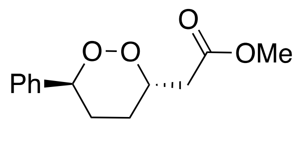

16f

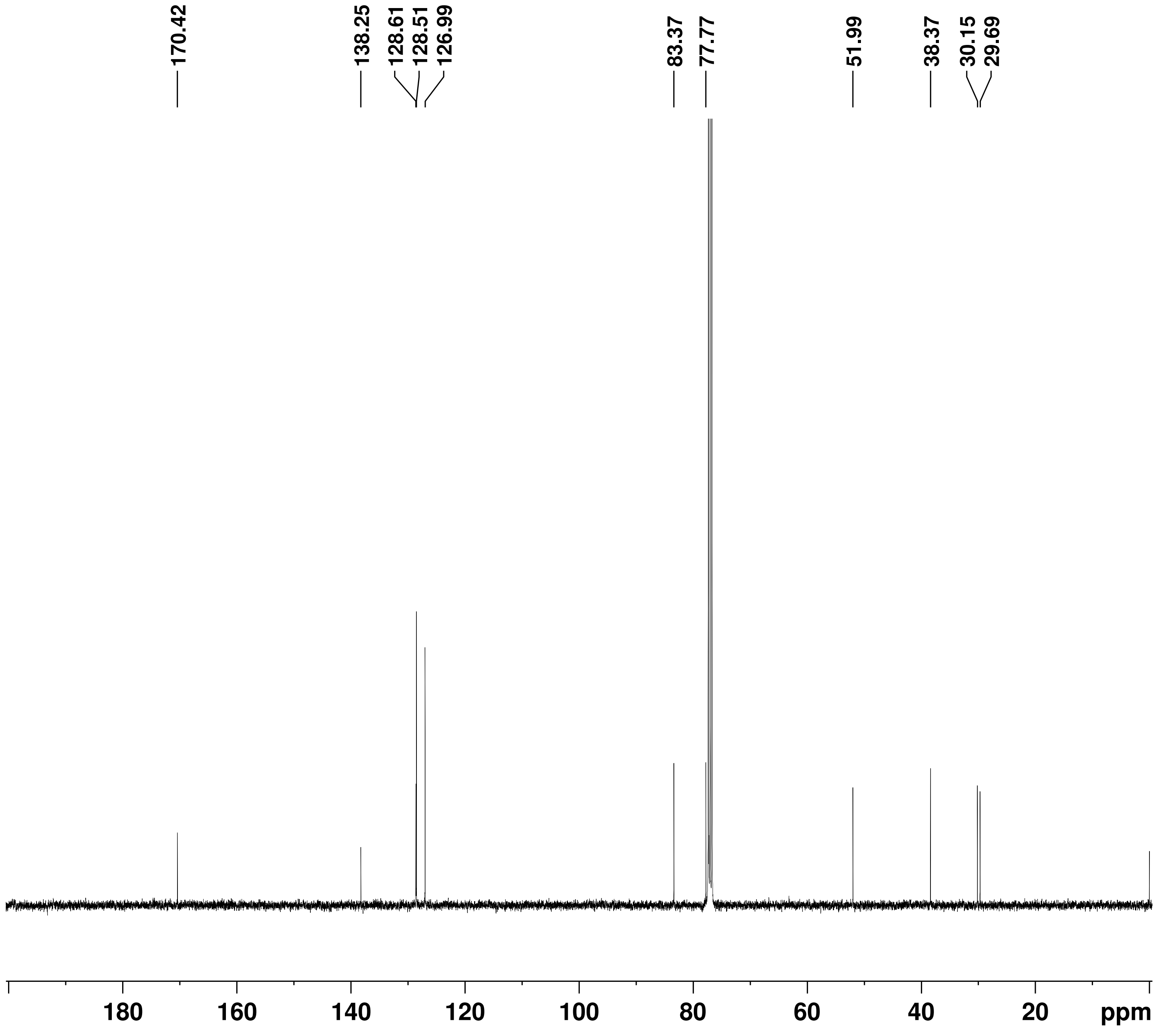

^{13}C spectrum of compound **16f**

400 MHz, $CDCl_3$

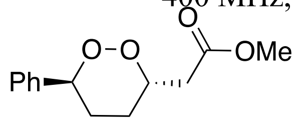

16f

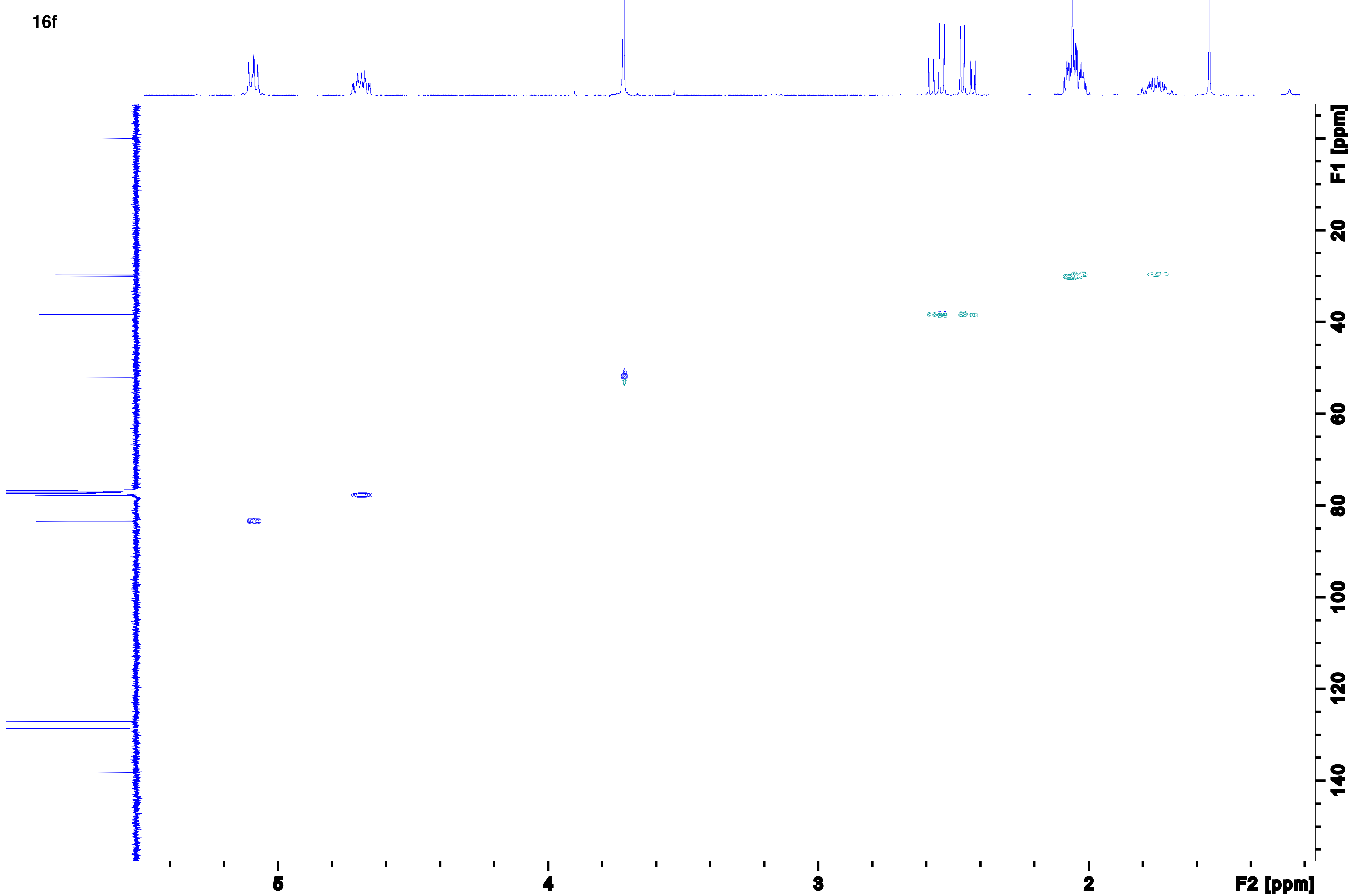

$^{1}H/^{13}C$ HSQC spectrum of compound **16f**

400 MHz, C_6D_6

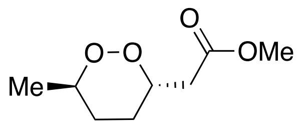

16g

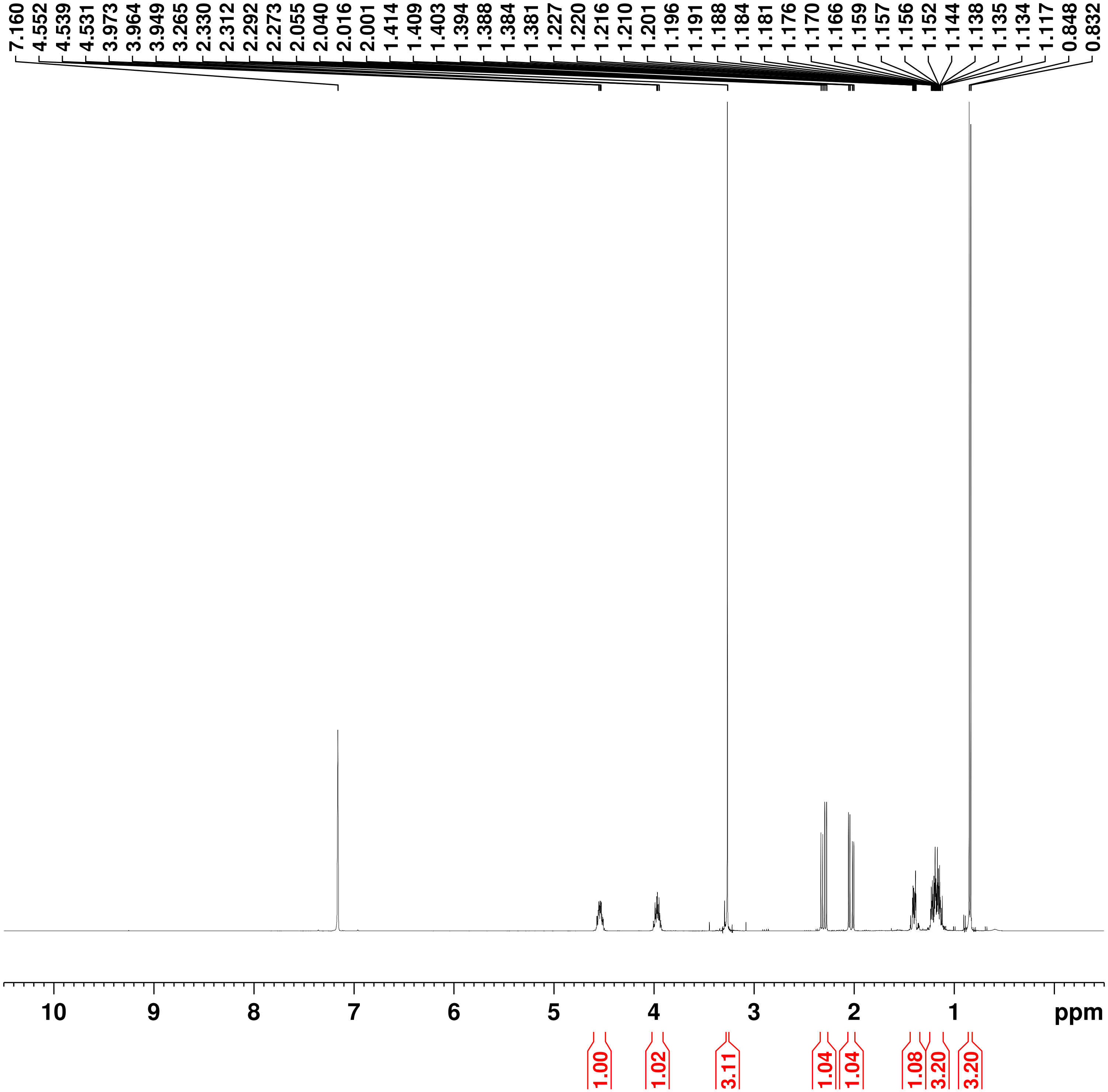

^{1}H spectrum of compound **16g**

100 MHz, C_6D_6

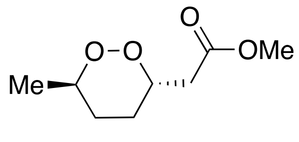

16g

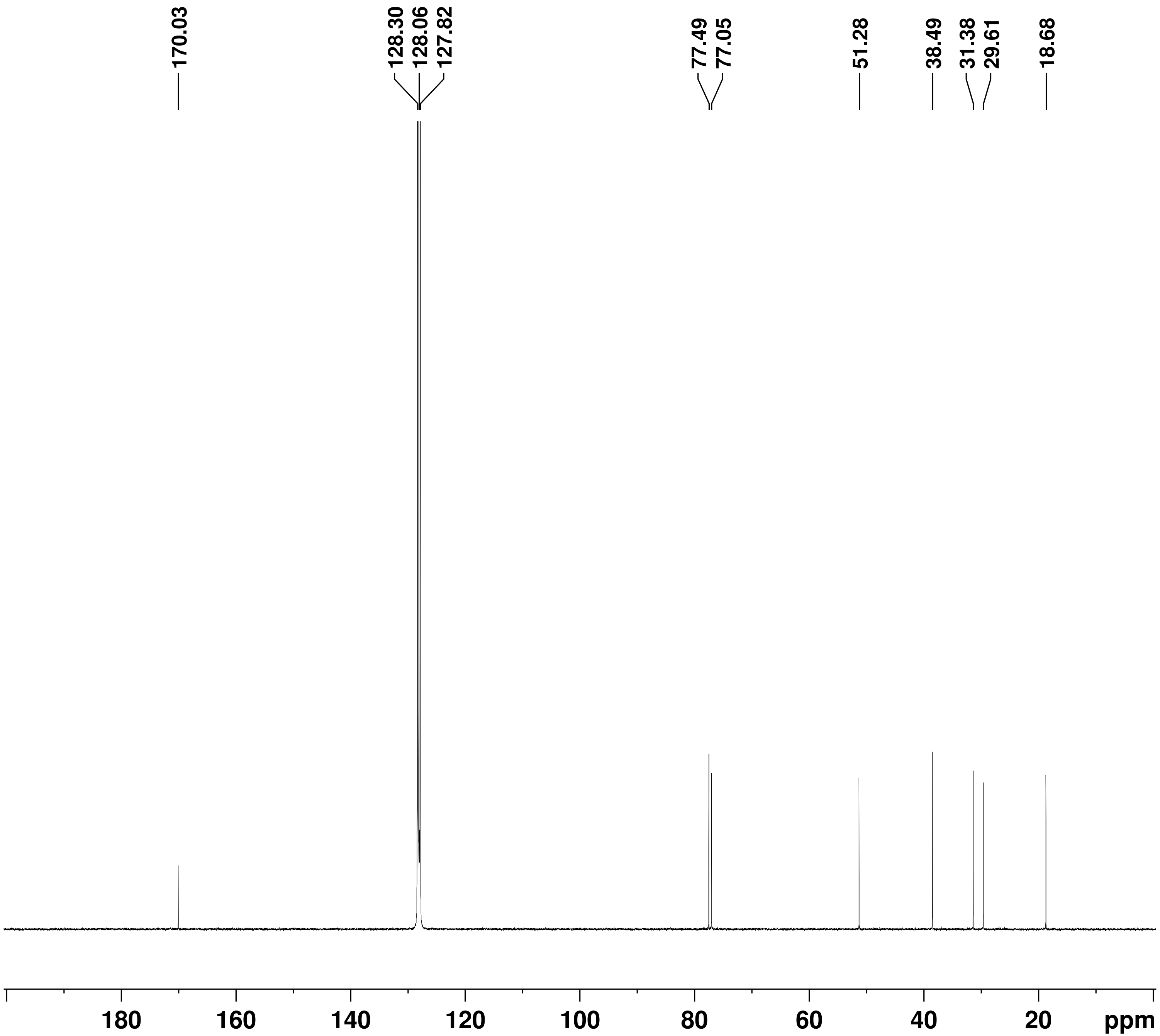

^{13}C spectrum of compound **16g**

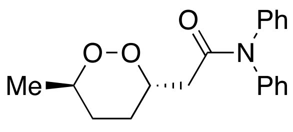

16h

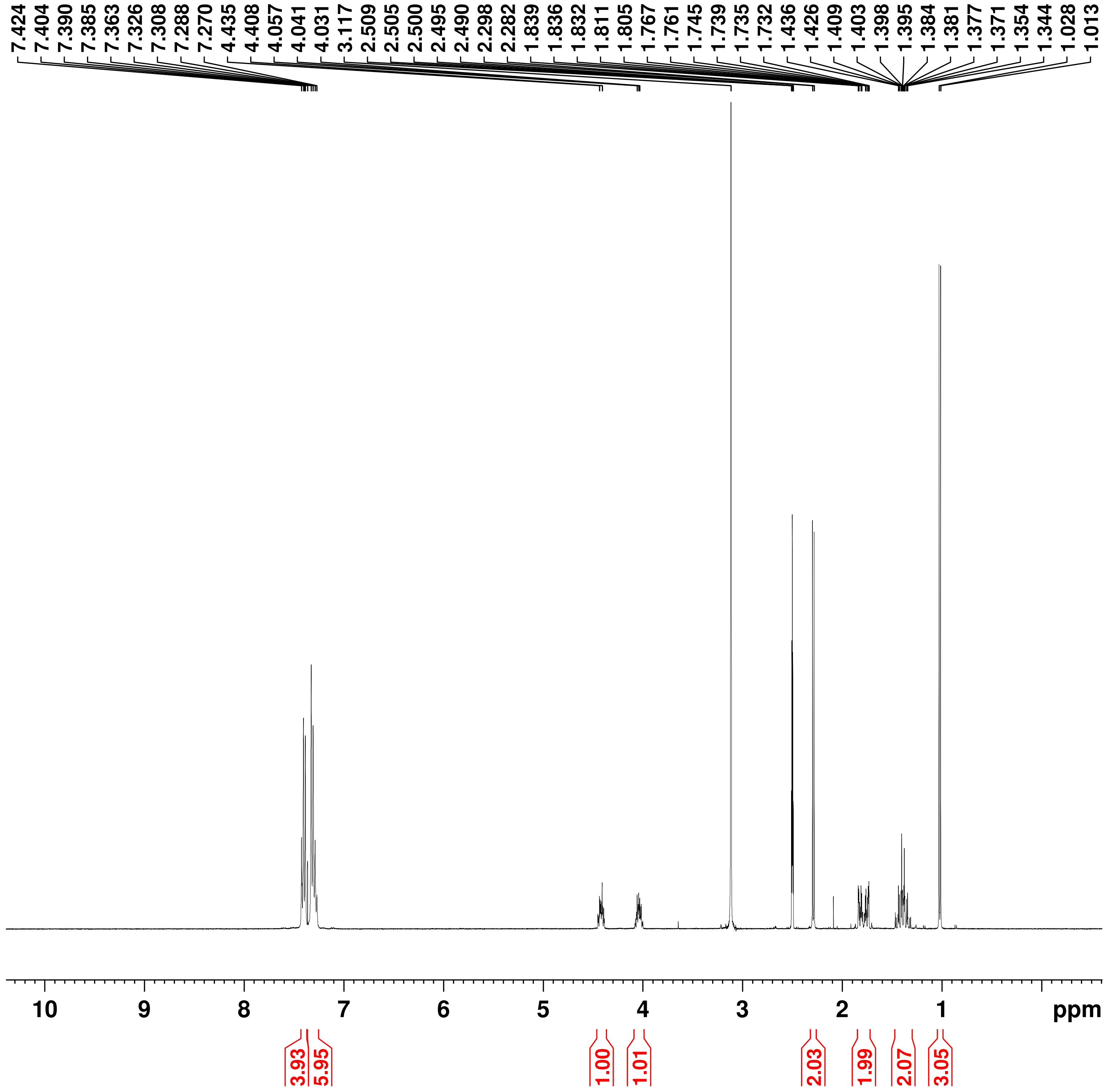

^{1}H spectrum of compound **16h**

400 MHz, $(CD_3)_2SO$

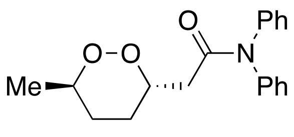

16h

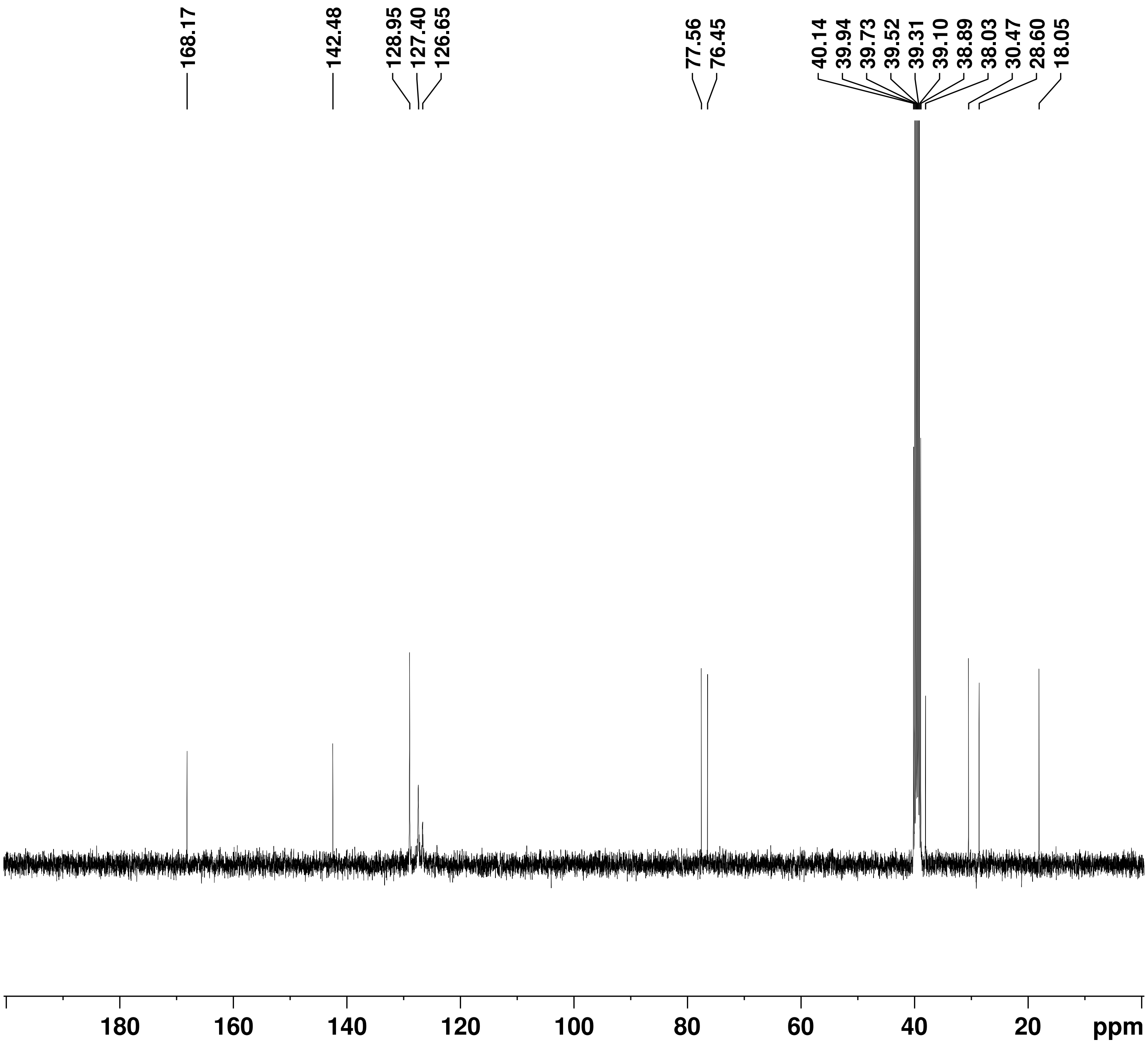

^{13}C spectrum of compound **16h**

100 MHz, $(CD_3)_2SO$

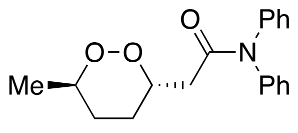

16h

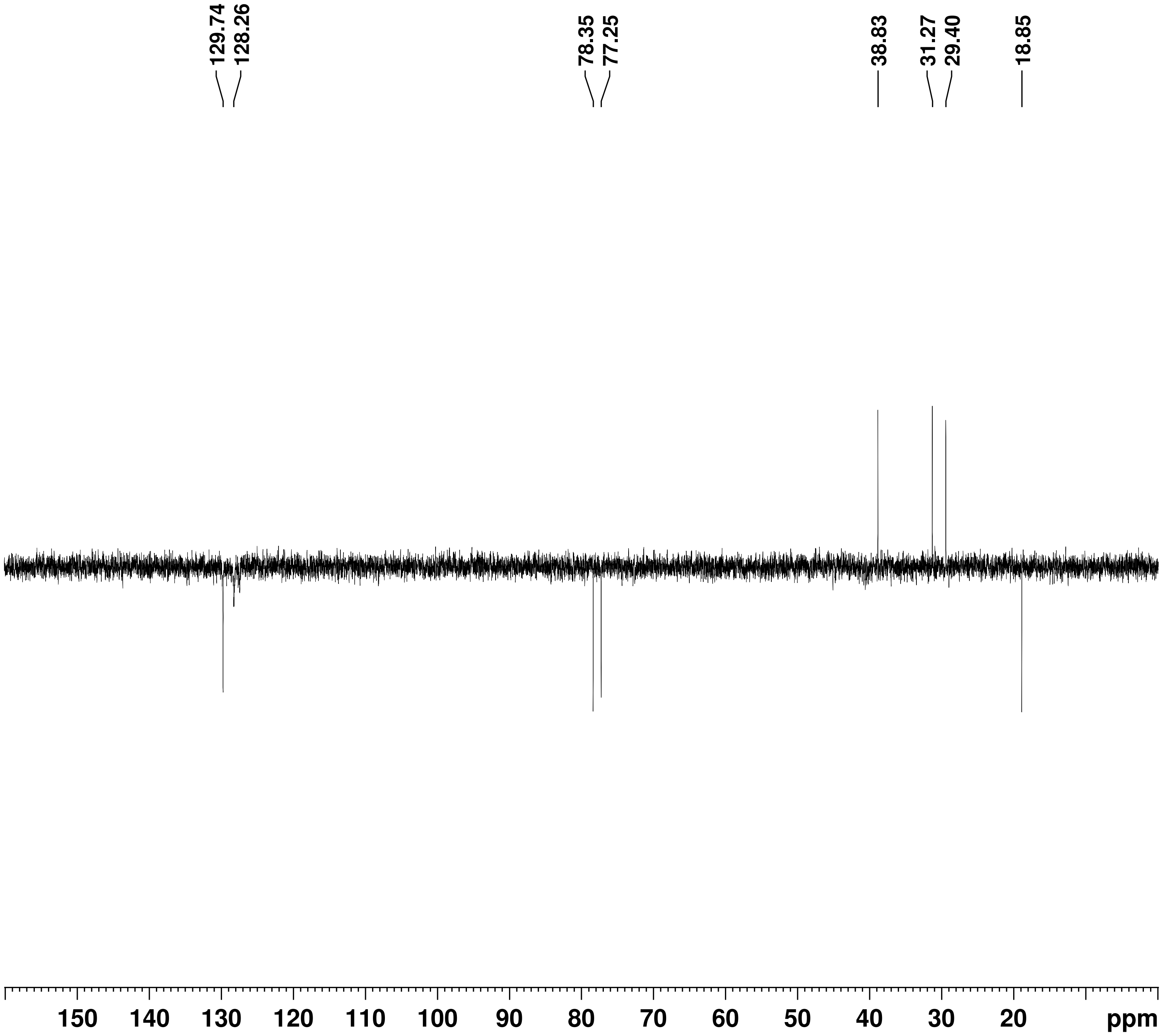

^{13}C DEPT spectrum of compound **16h**

600 MHz, C_6D_6

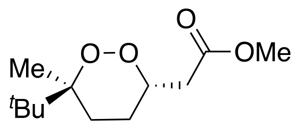

16i

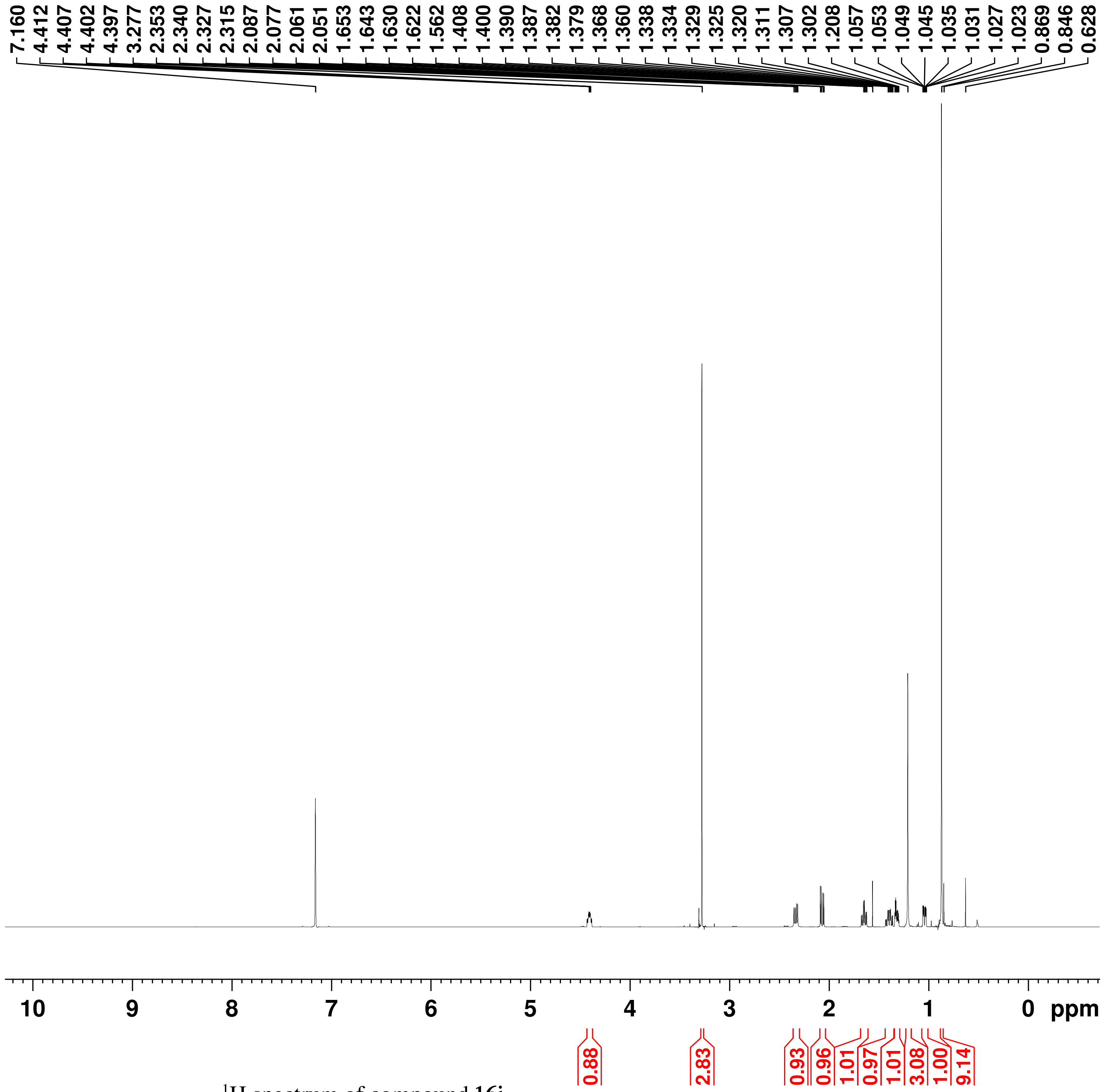

^{1}H spectrum of compound **16i**

100 MHz, C_6D_6

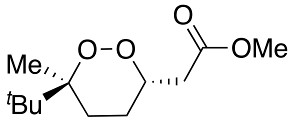

16i

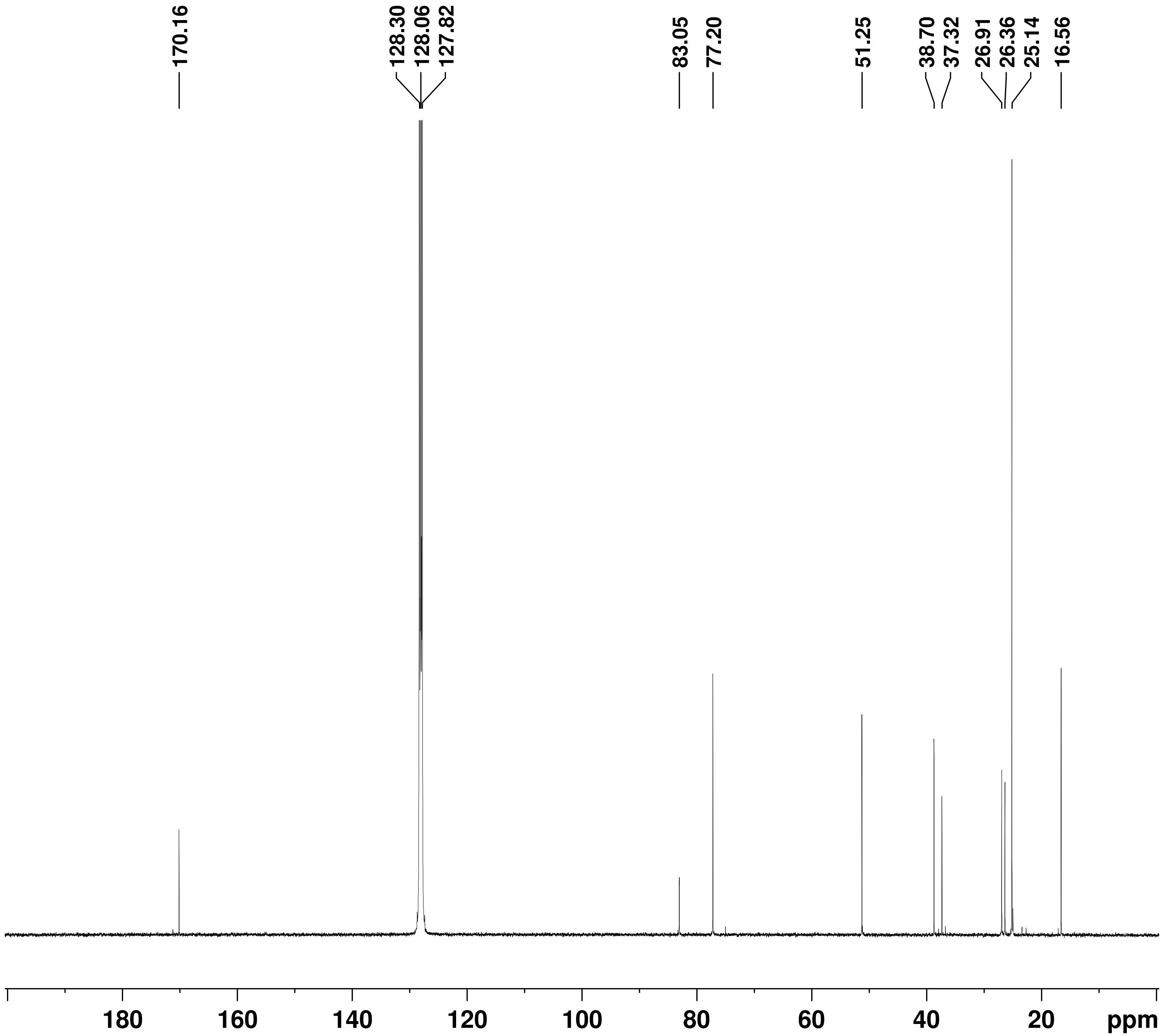

^{13}C spectrum of compound **16i**

400 MHz, C_6D_6

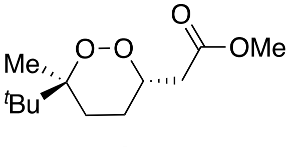

16i

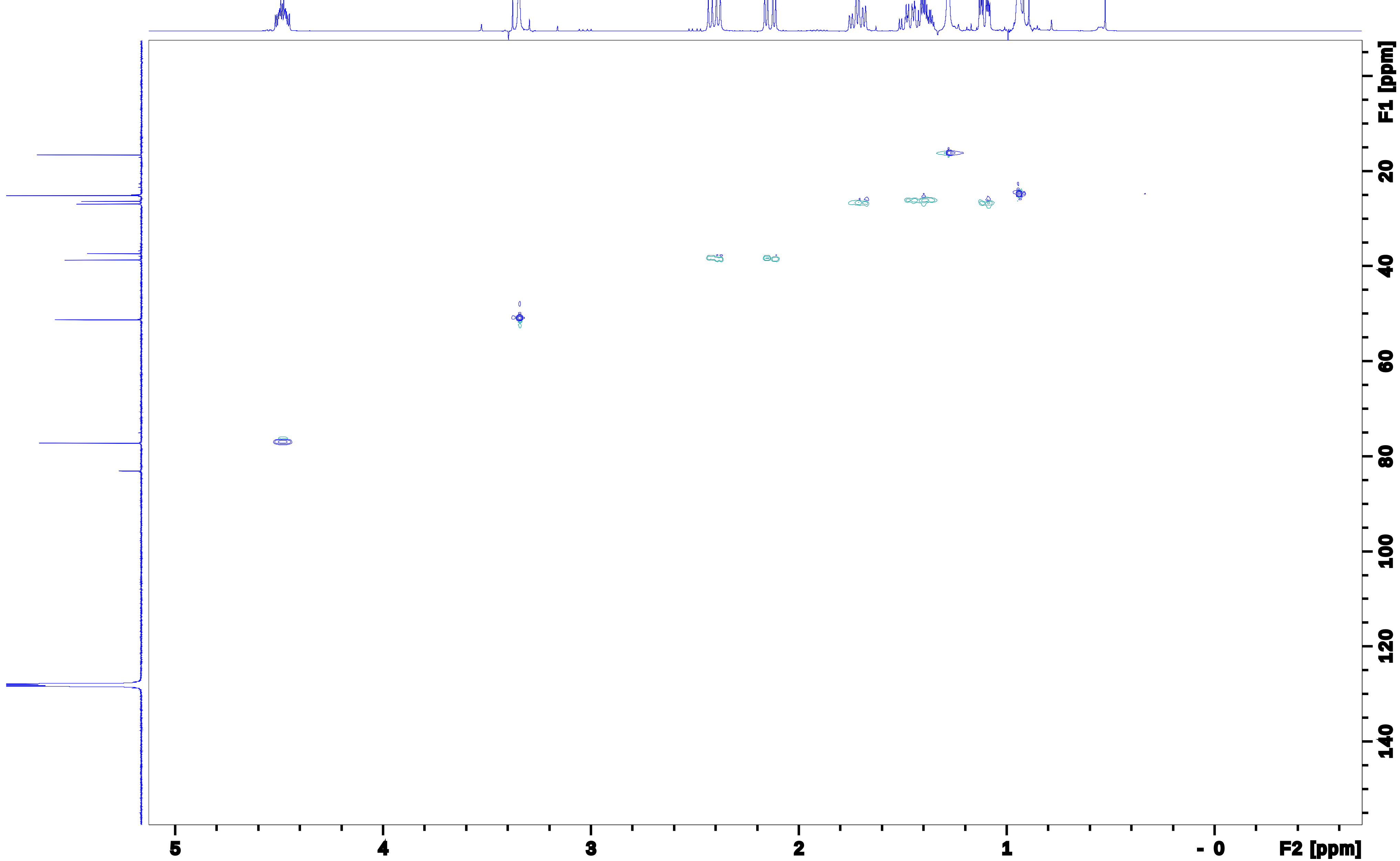

$^{1}H/^{13}C$ HSQC spectrum of compound **16i**

400 MHz, $(CD_3)_2SO$

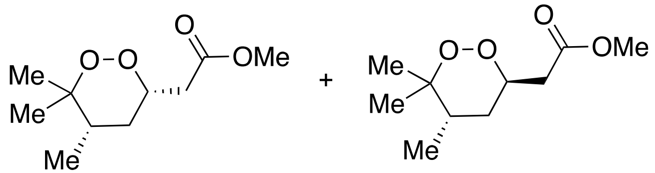

16j **16j'**

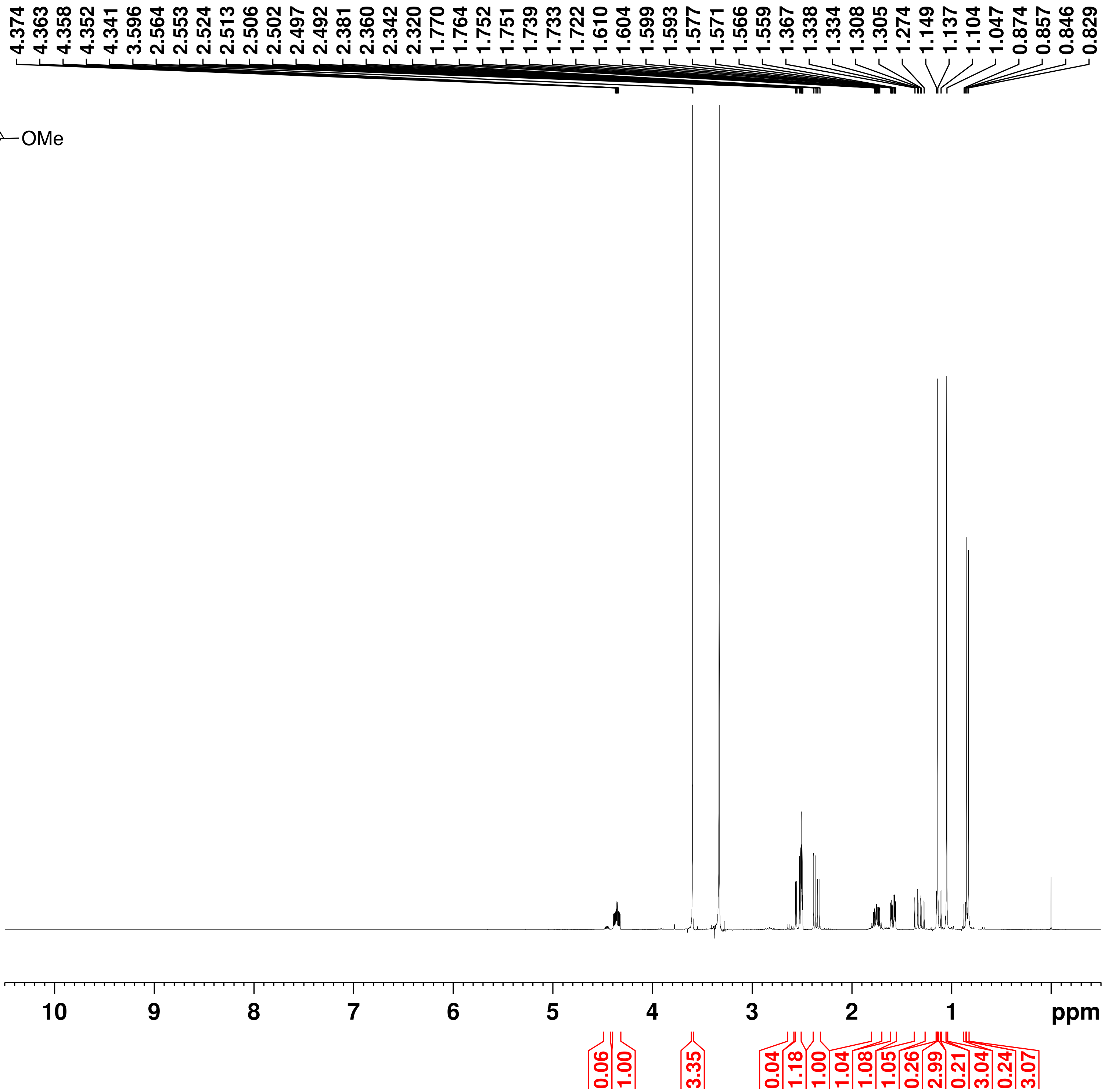

^{1}H spectrum of compound **16j**

100 MHz, $(CD_3)_2SO$

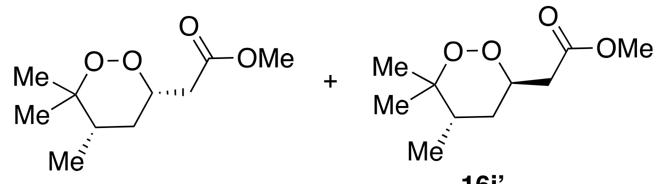

16j + 16j'

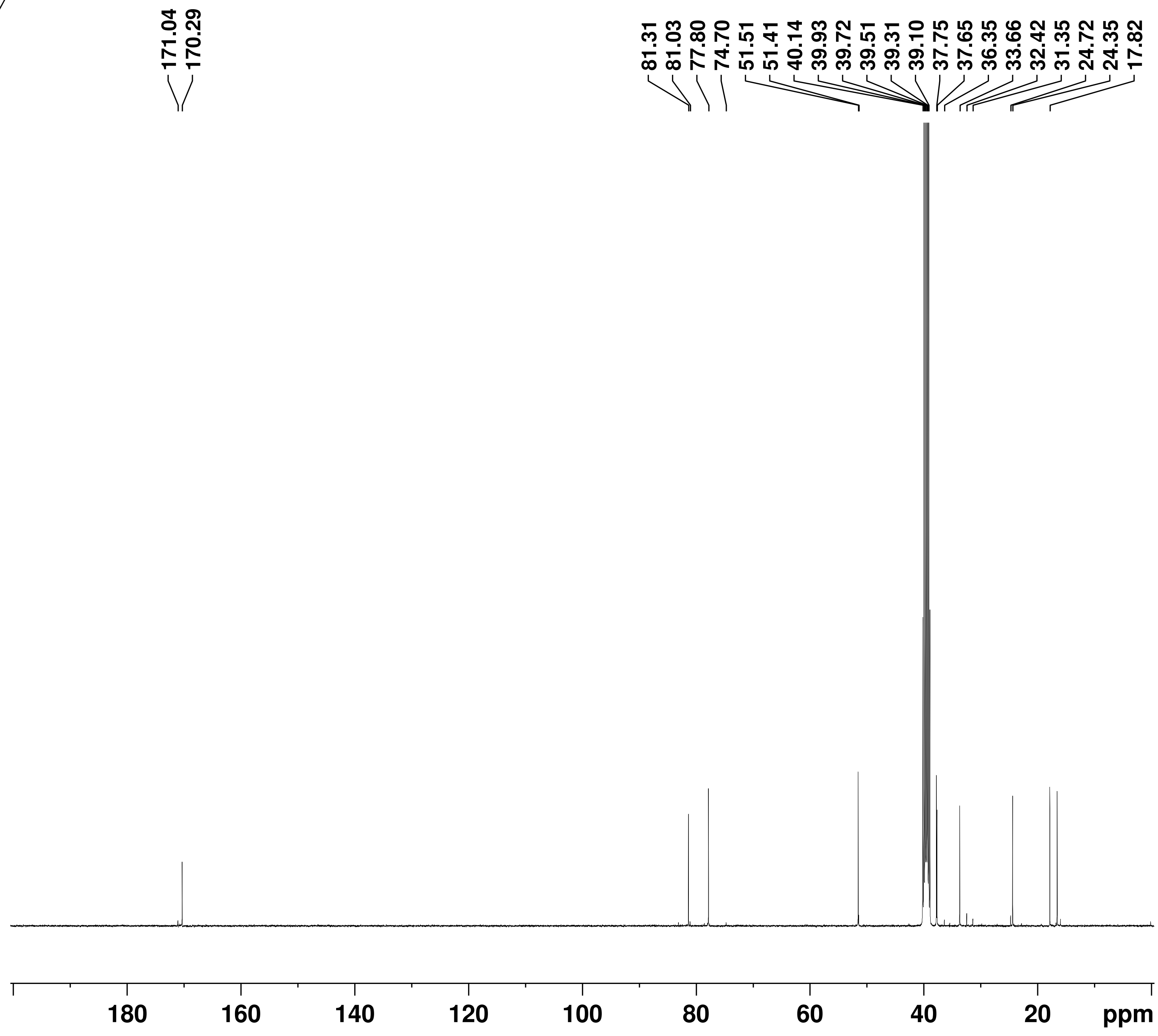

^{13}C HSQC spectrum of compound **16j**

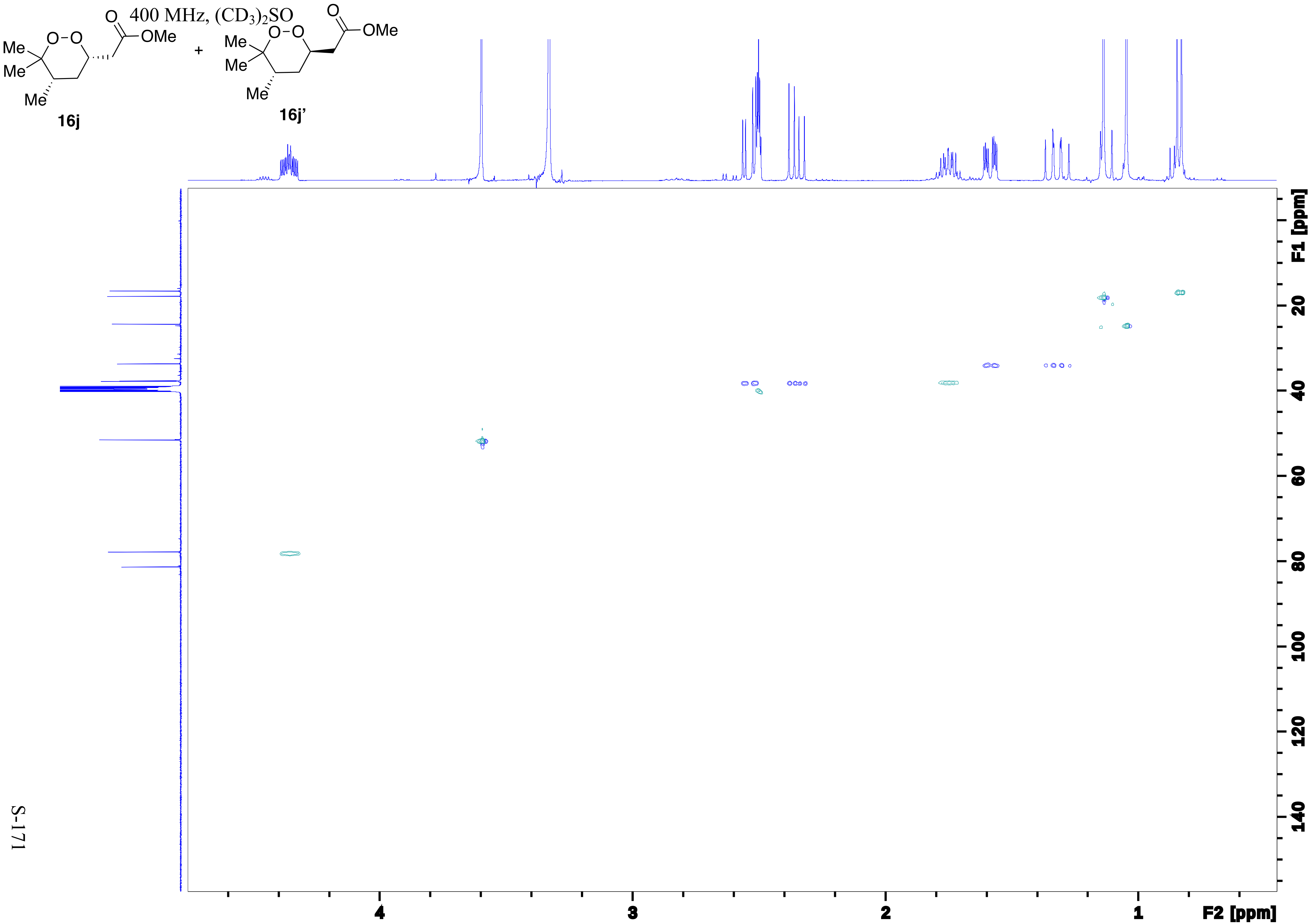

$^1H/^{13}C$ HSQC spectrum of compound **16j**

400 MHz, C_6D_6

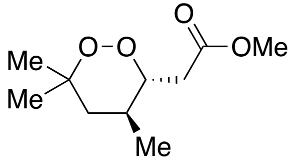

16k

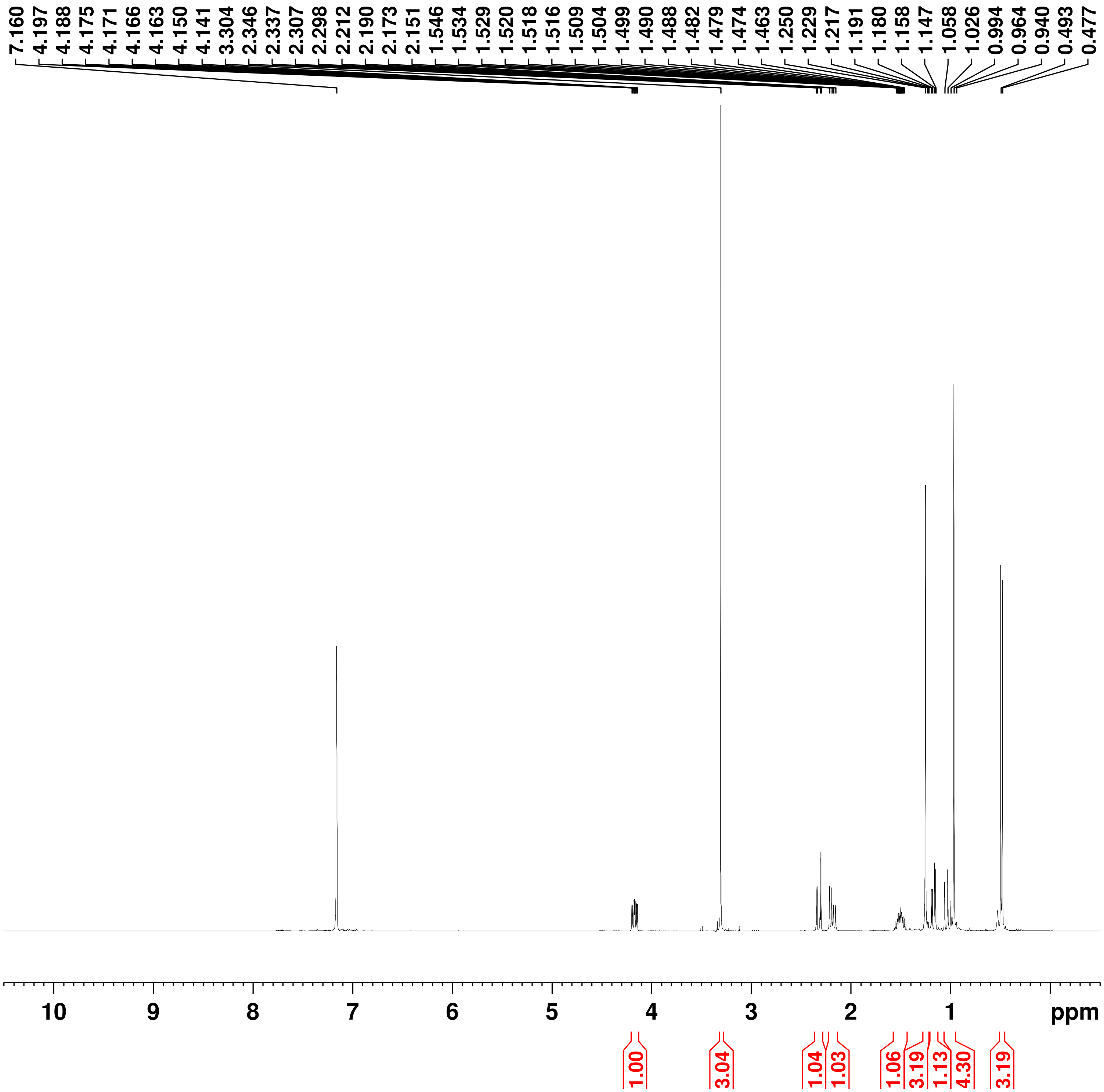

^{1}H spectrum of compound **16k**

100 MHz, C_6D_6

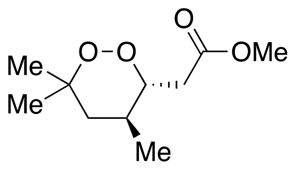

16k

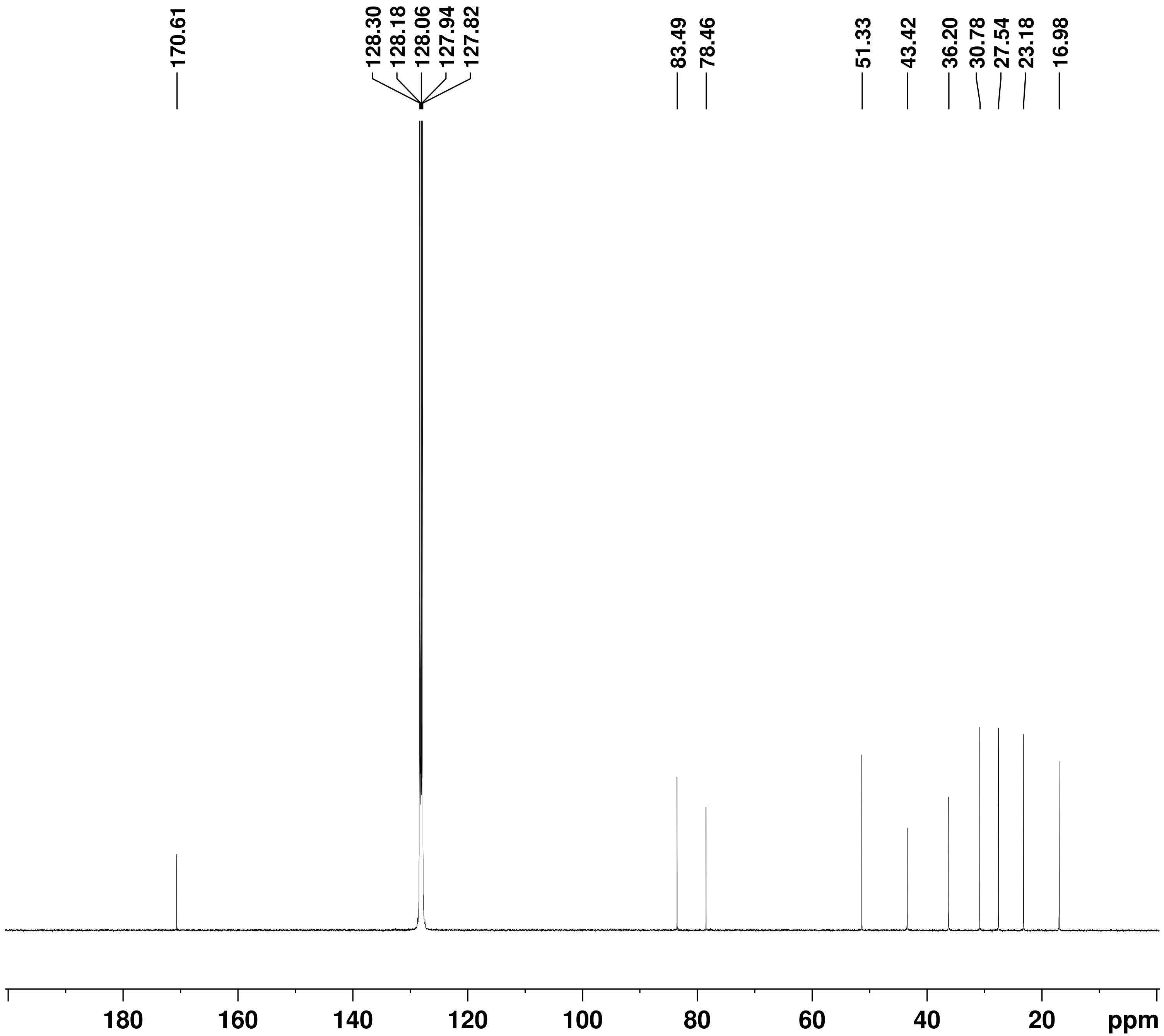

^{13}C spectrum of compound **16k**

400 MHz, C_6D_6

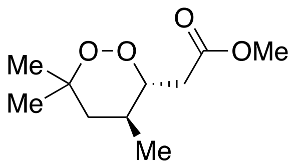

16k

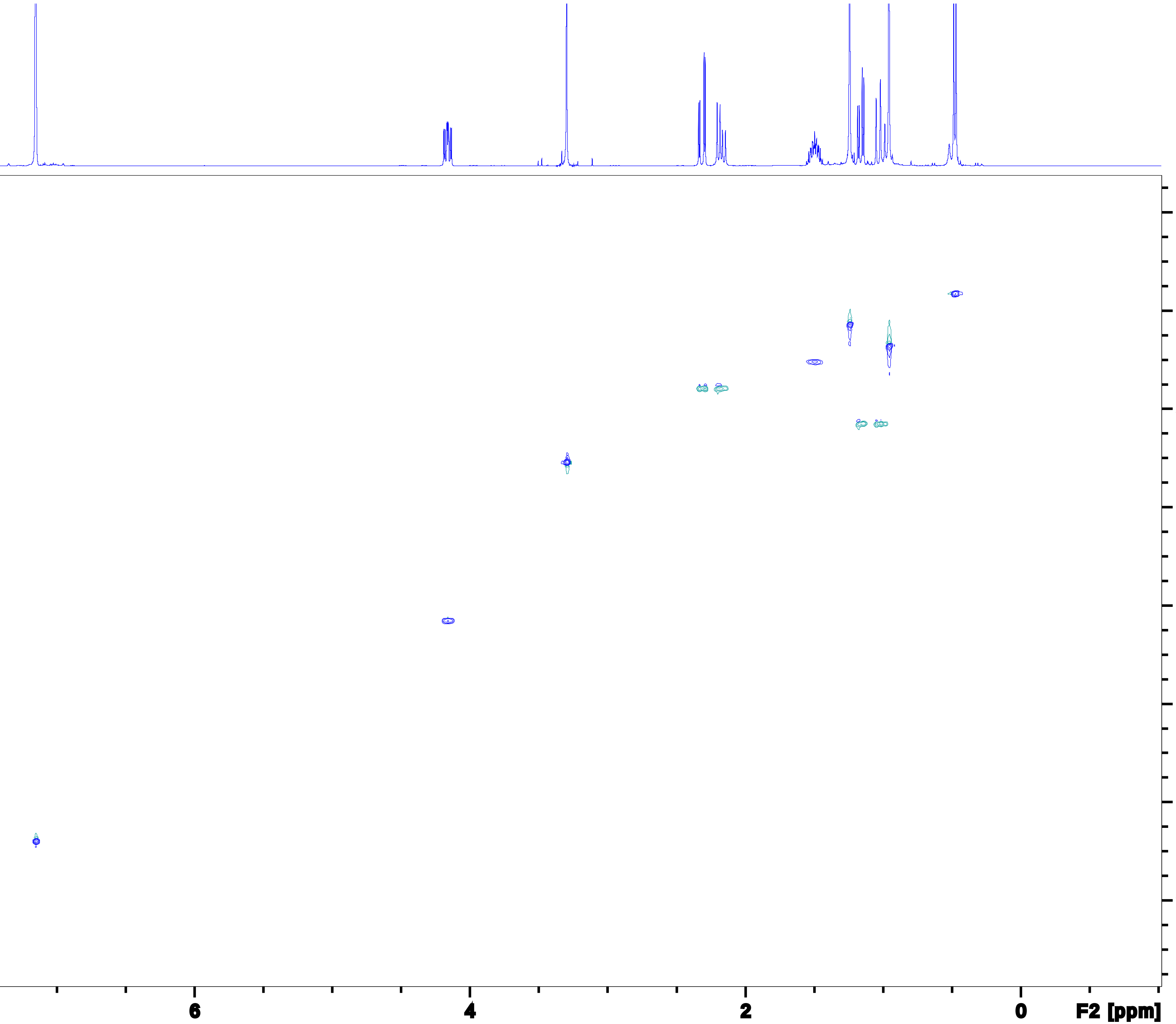

$^1H/^{13}C$ HSQC spectrum of compound **16k**

400 MHz, C_6D_6

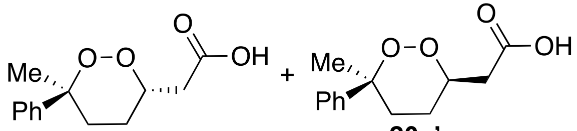

20a + 20a’

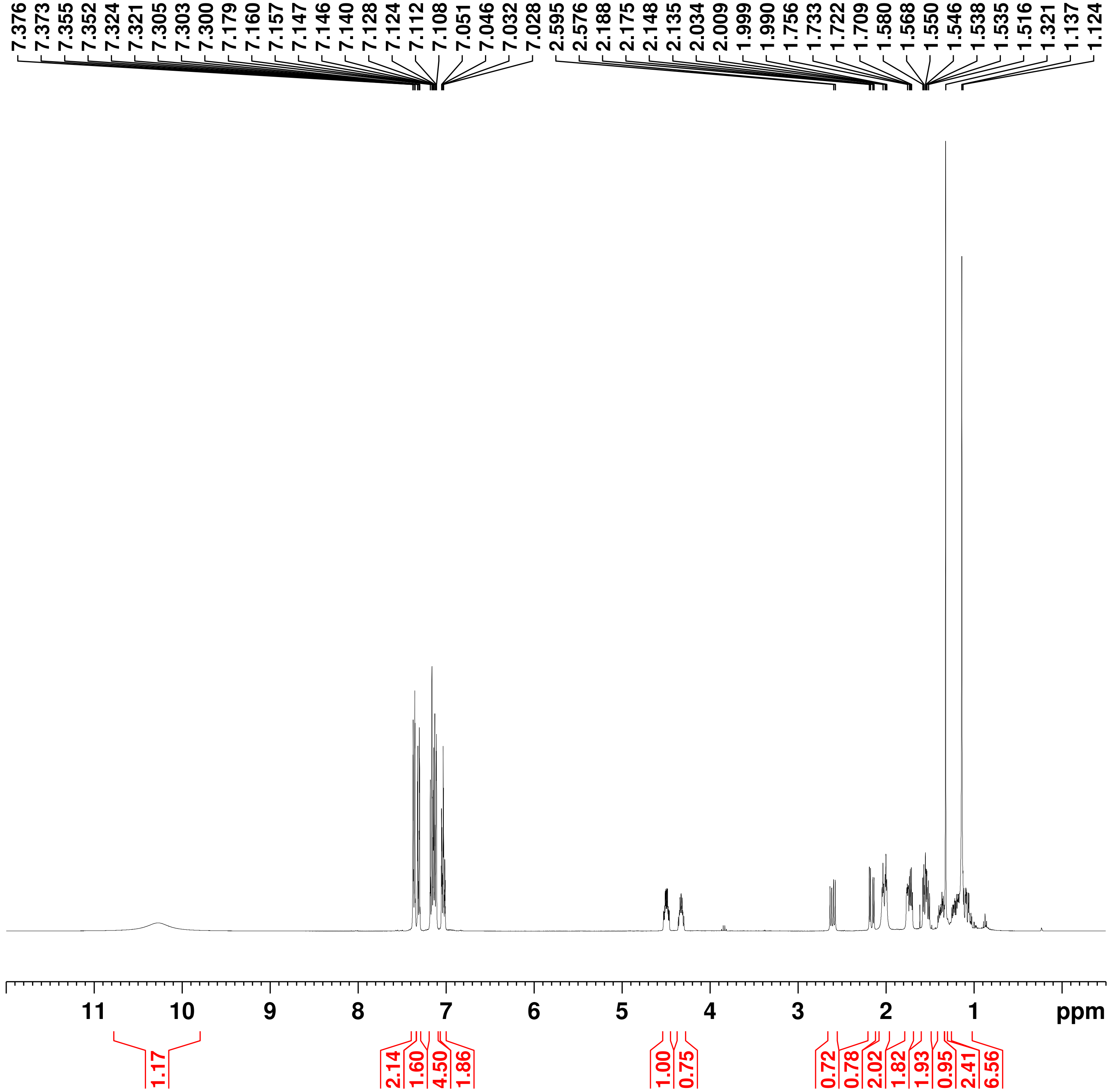

^{1}H spectrum of compounds **20a & 20a'**

100 MHz, C_6D_6

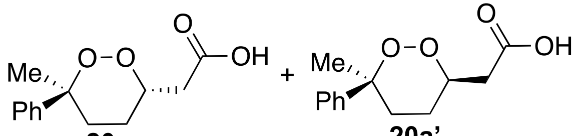

20a + **20a'**

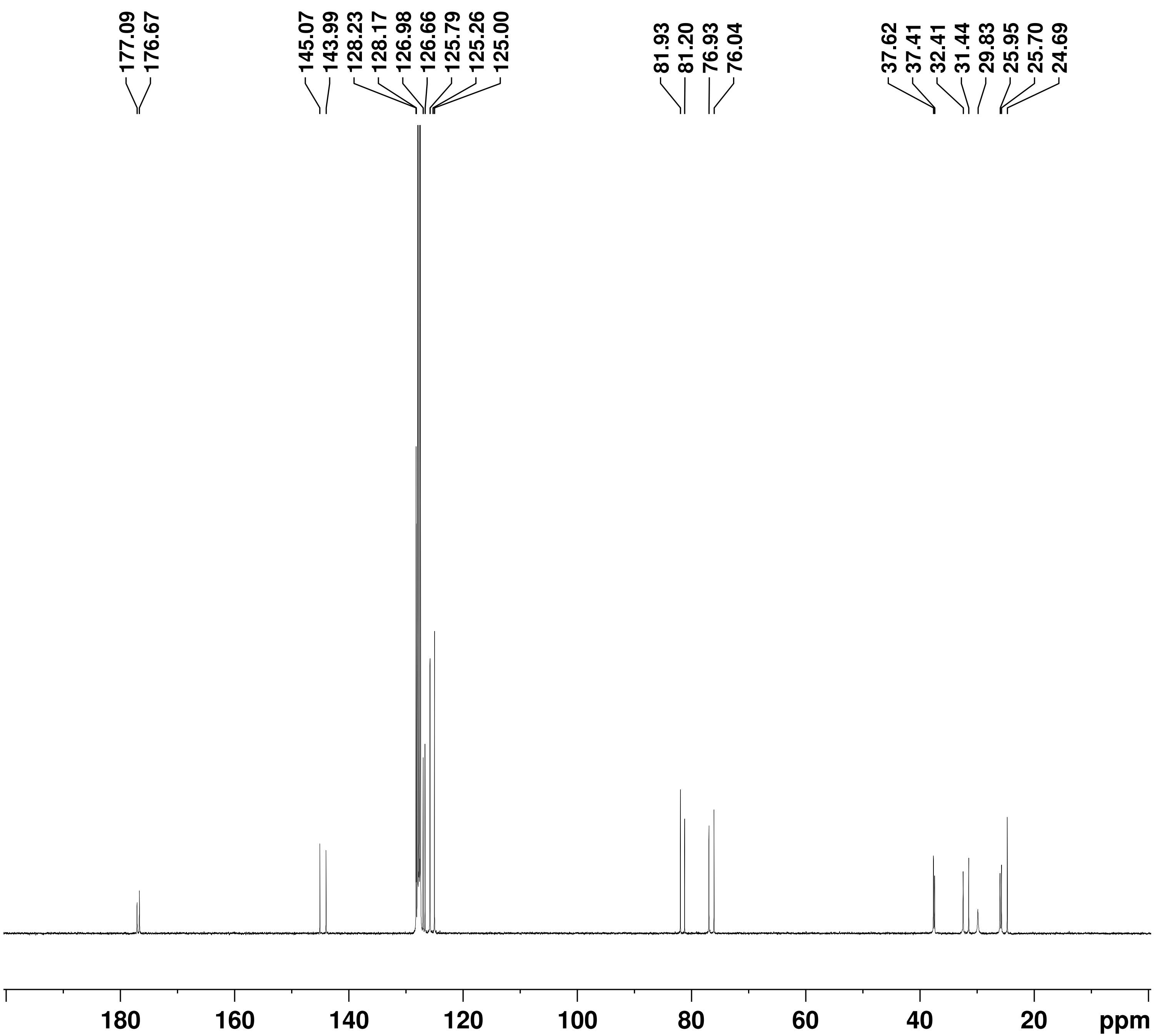

^{13}C spectrum of compounds **20a & 20a'**

400 MHz, C_6D_6

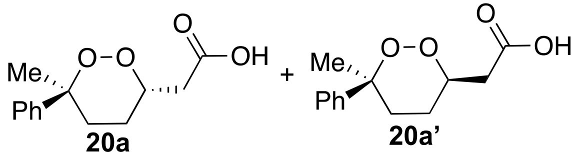

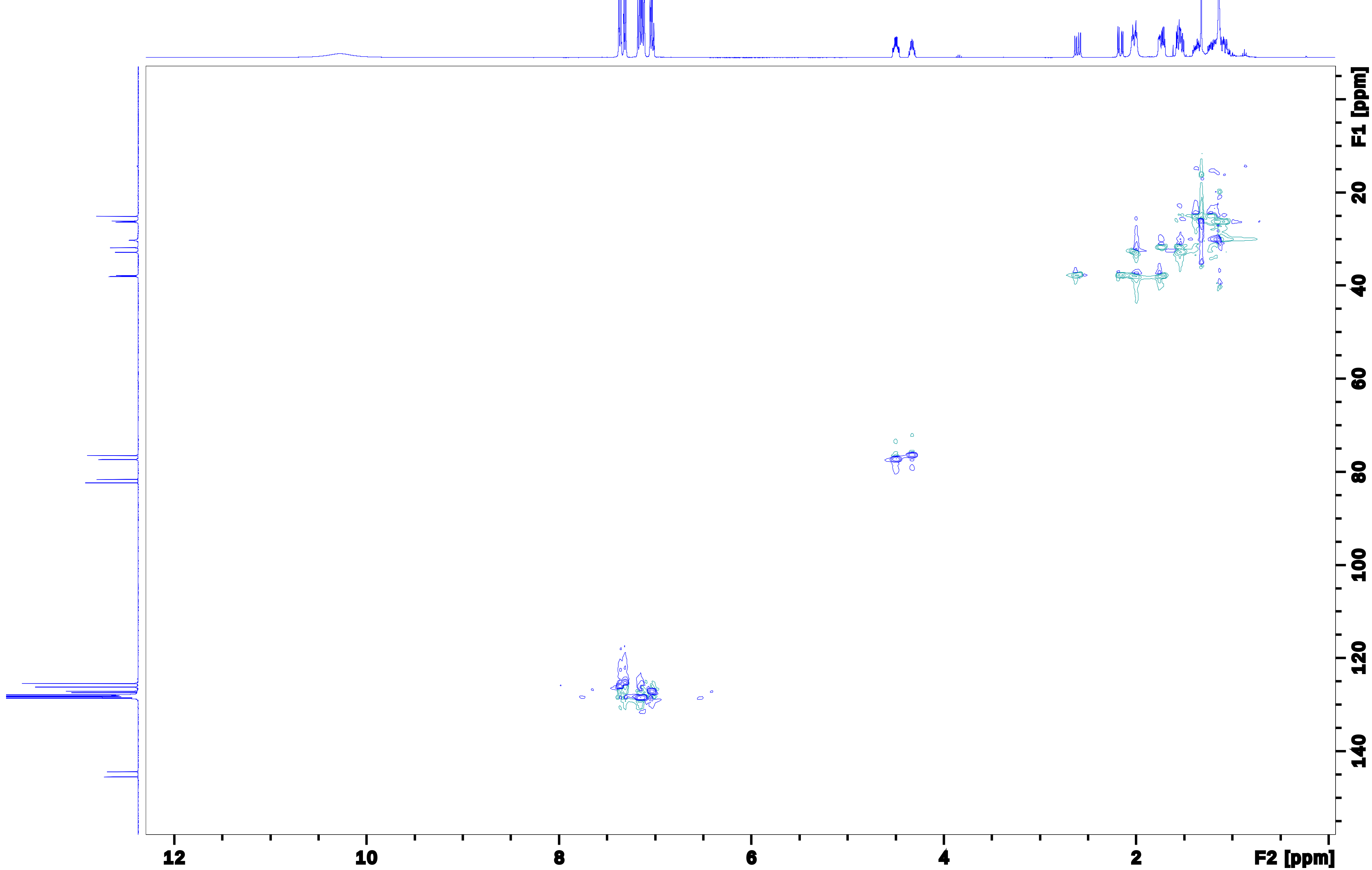

$^{1}H/^{13}C$ HSQC spectrum of compounds **20a & 20a'**

400 MHz, C_6D_6

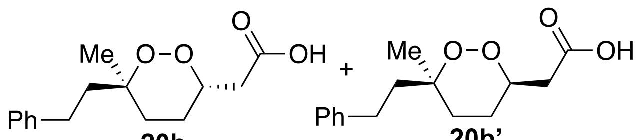

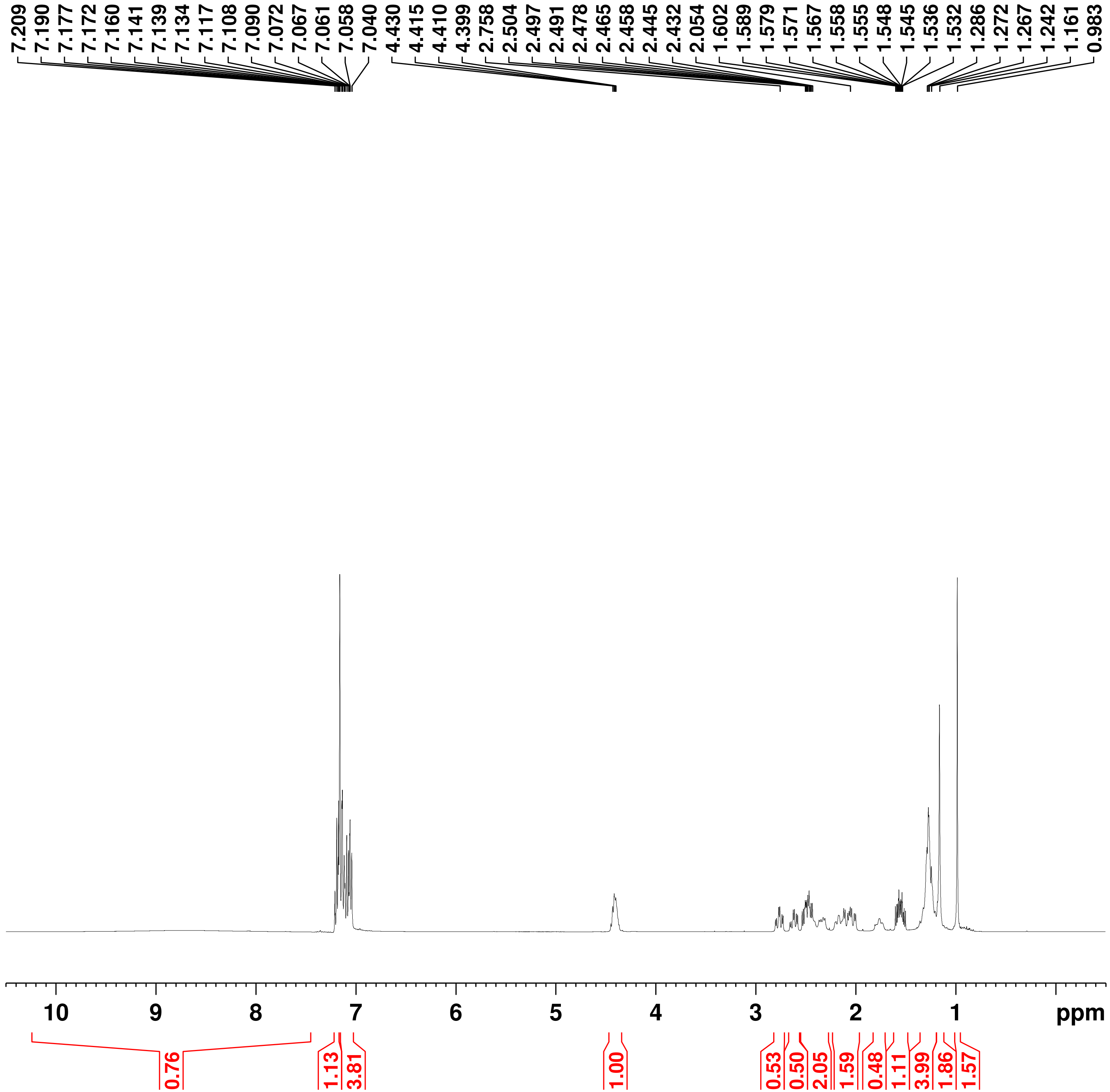

^{1}H spectrum of compounds **20b & 20b'**

100 MHz, C_6D_6

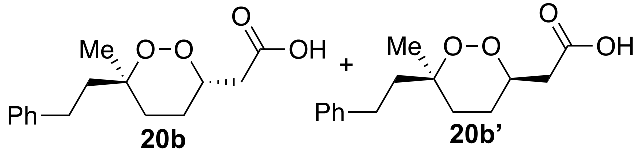

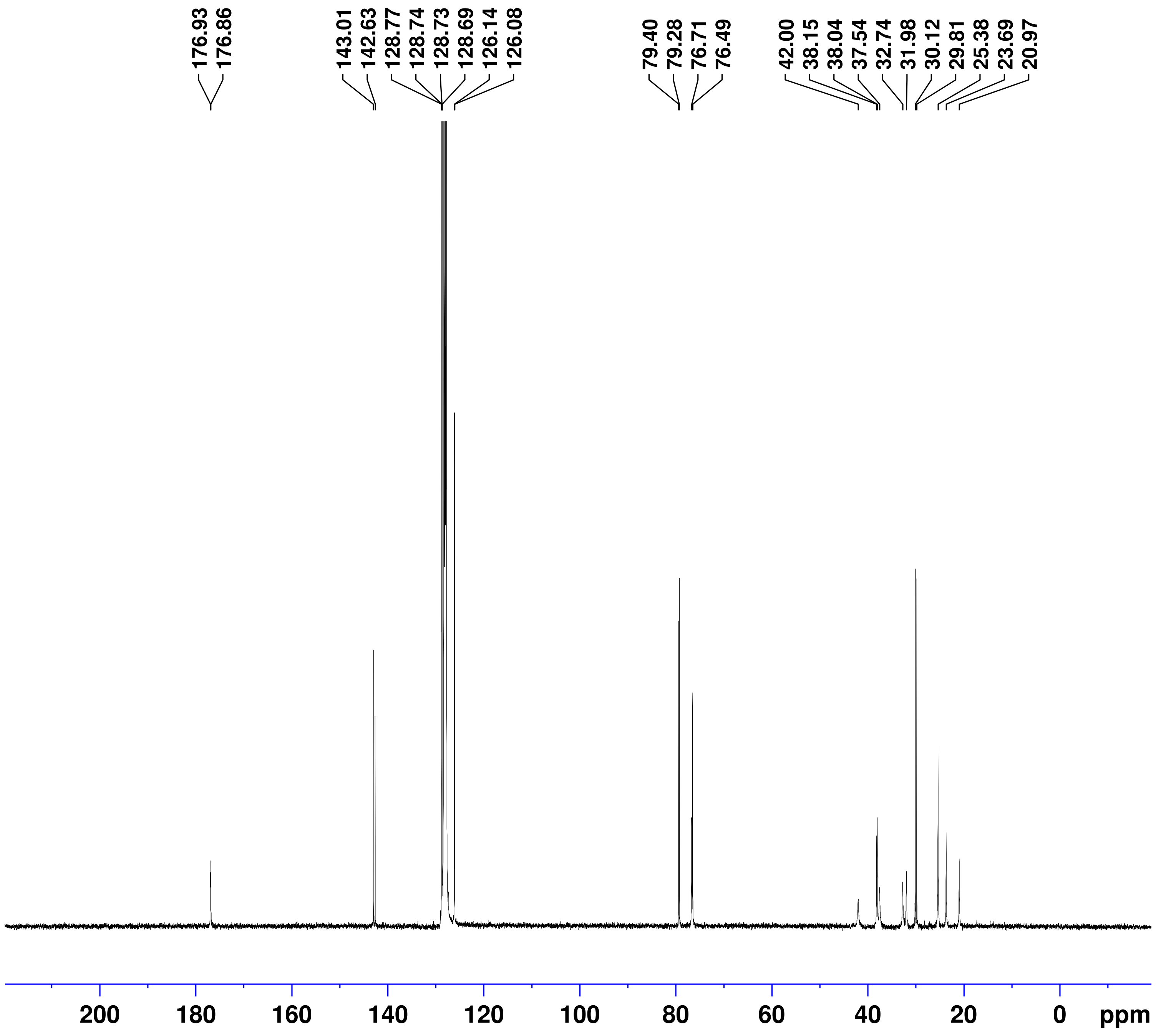

^{13}C spectrum of compounds **20b & 20b'**

400 MHz, C_6D_6

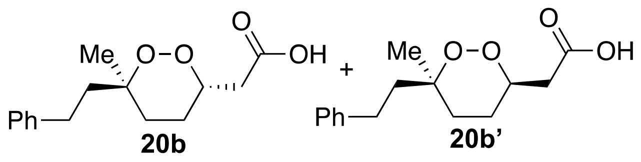

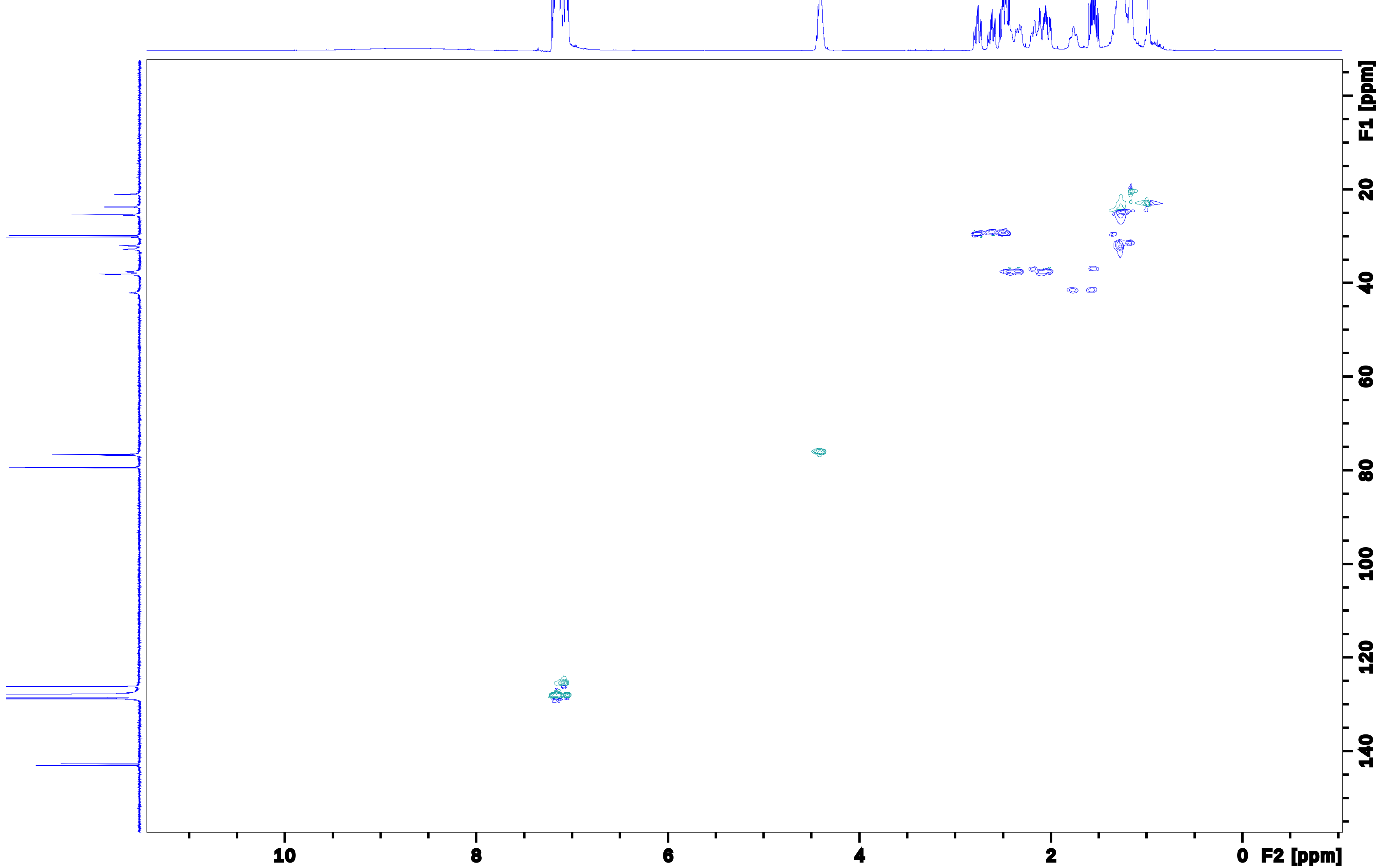

$^1H/^{13}C$ HSQC spectrum of compounds **20b & 20b'**

400 MHz, C_6D_6

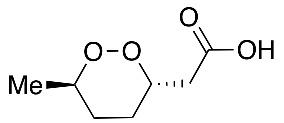

20c

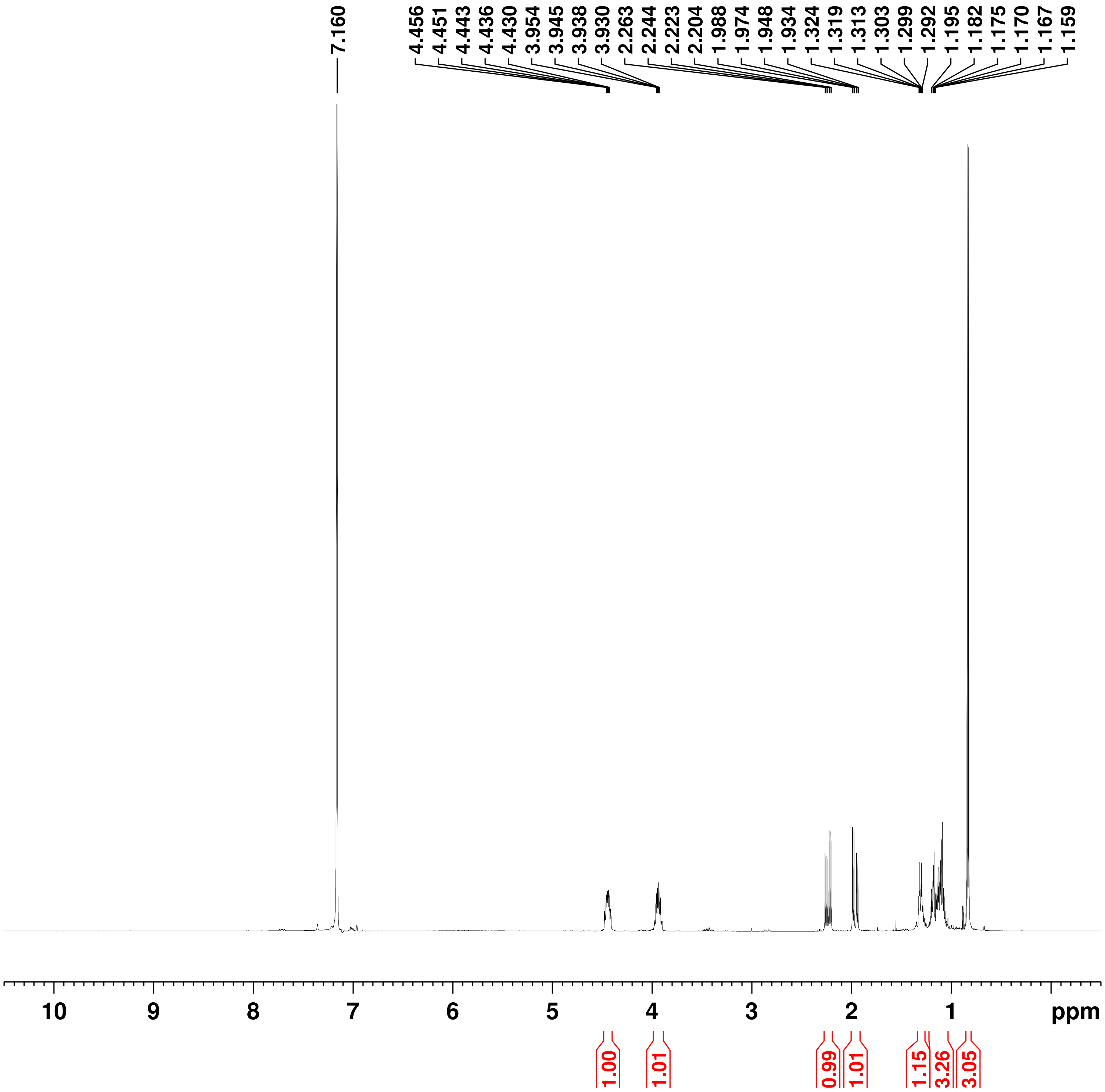

^{1}H spectrum of compound **20c**

100 MHz, C_6D_6

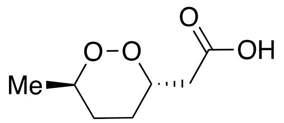

20c

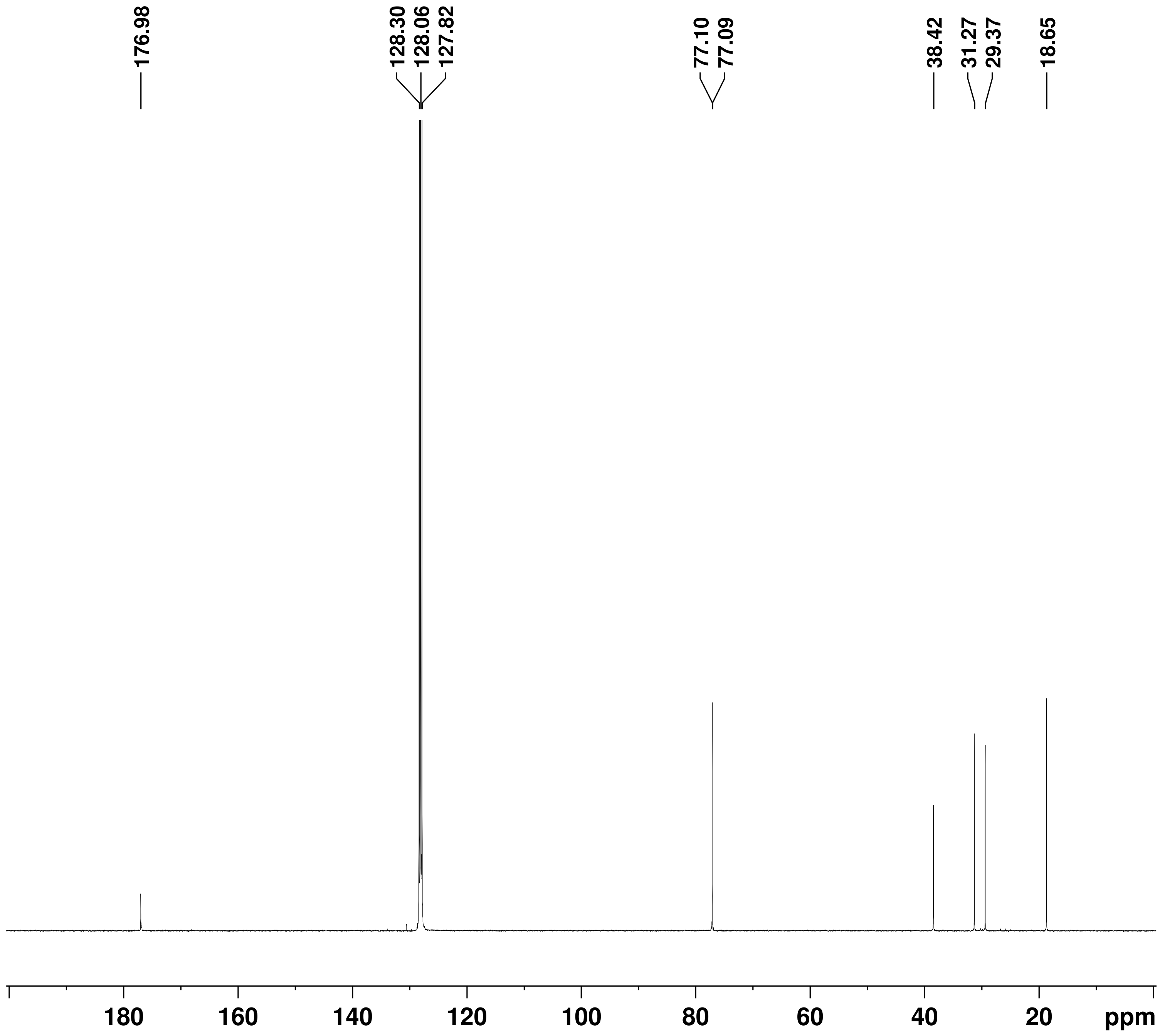

^{13}C spectrum of compound **20c**

400 MHz, C_6D_6

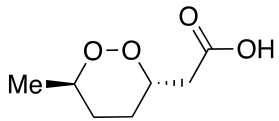

20c

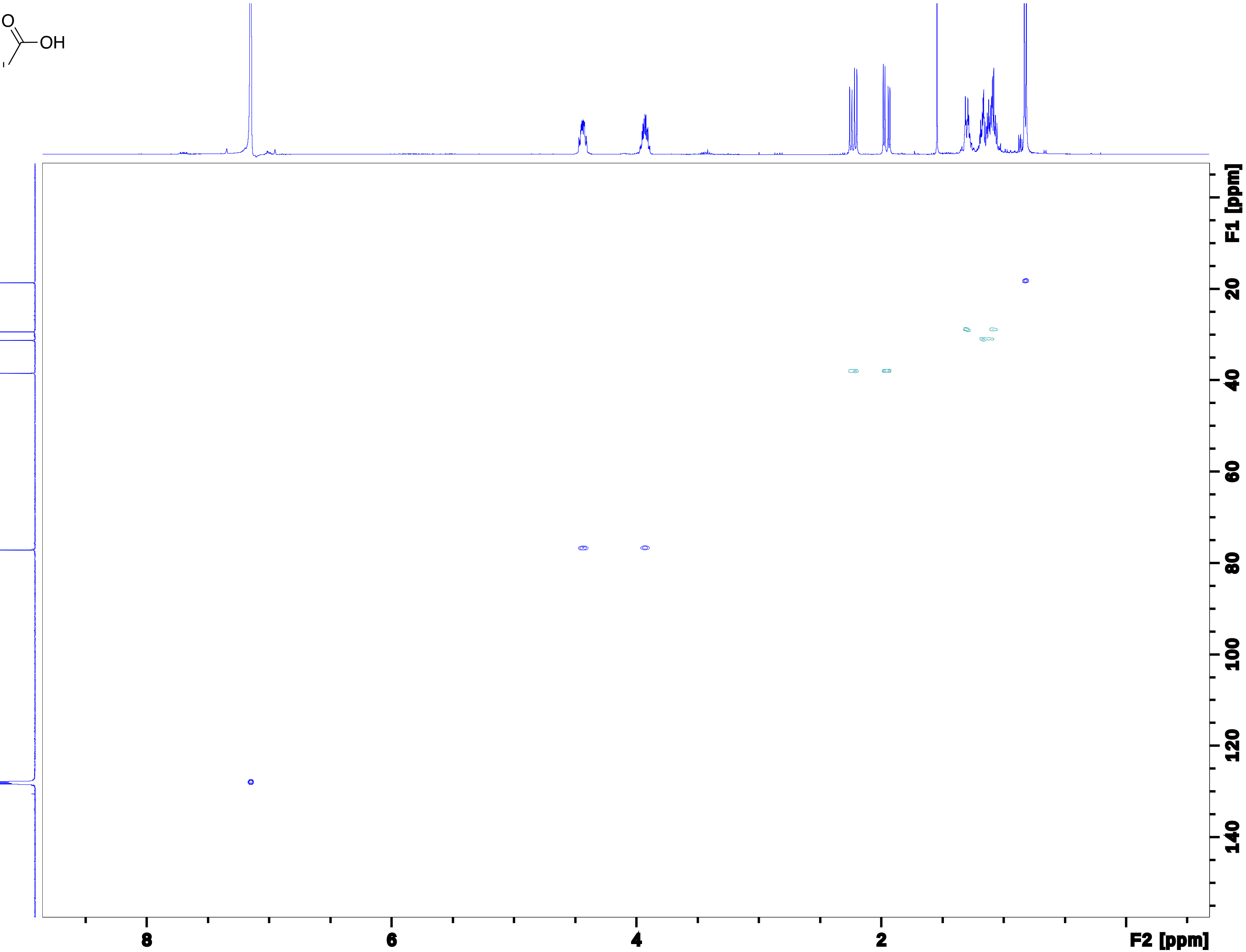

$^1H/^{13}C$ HSQC spectrum of compound **20c**

400 MHz, C_6D_6

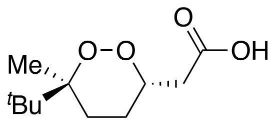

20d

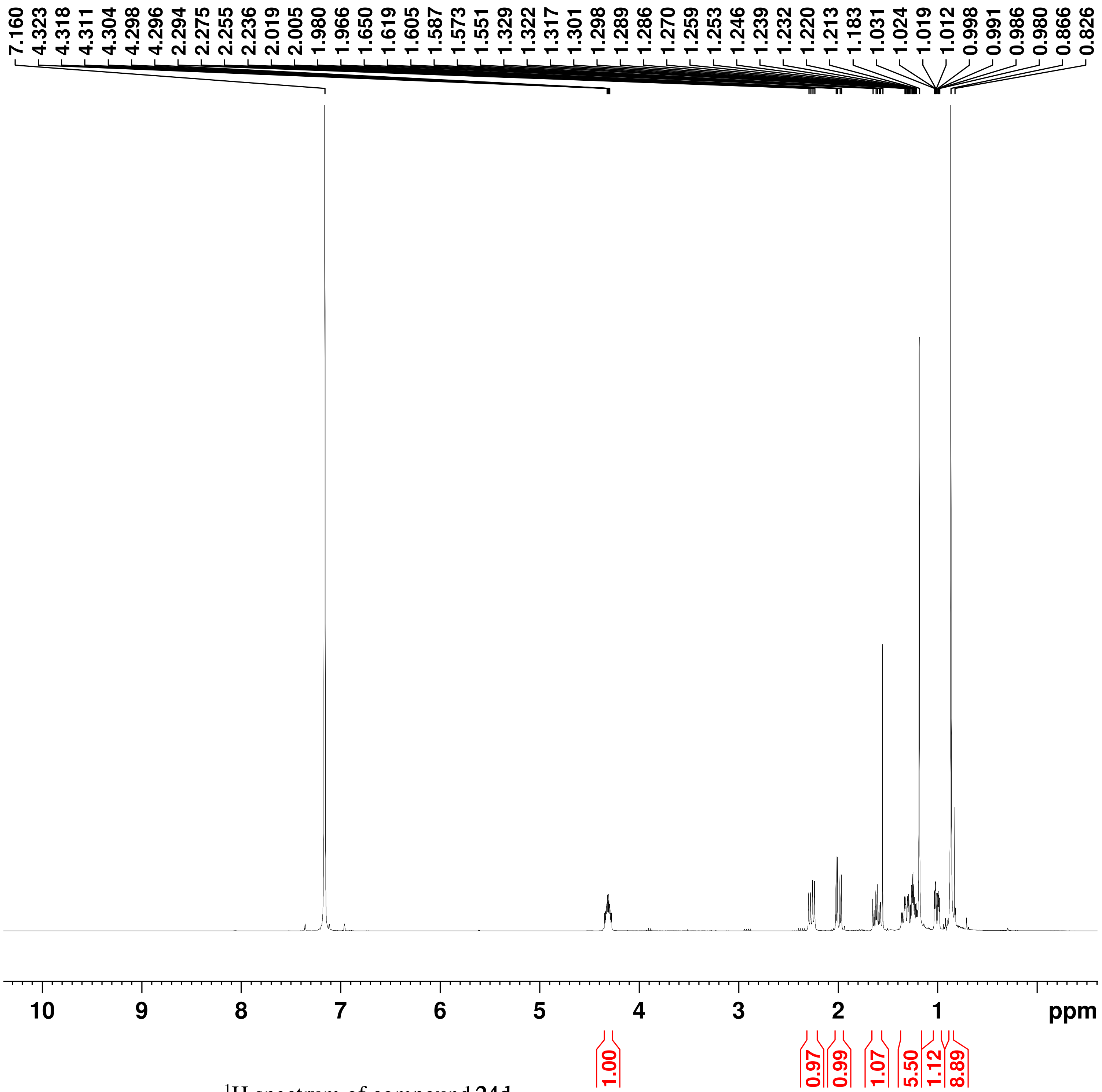

^{1}H spectrum of compound **24d**

100 MHz, C_6D_6

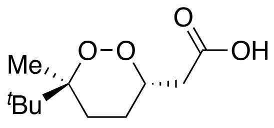

20d

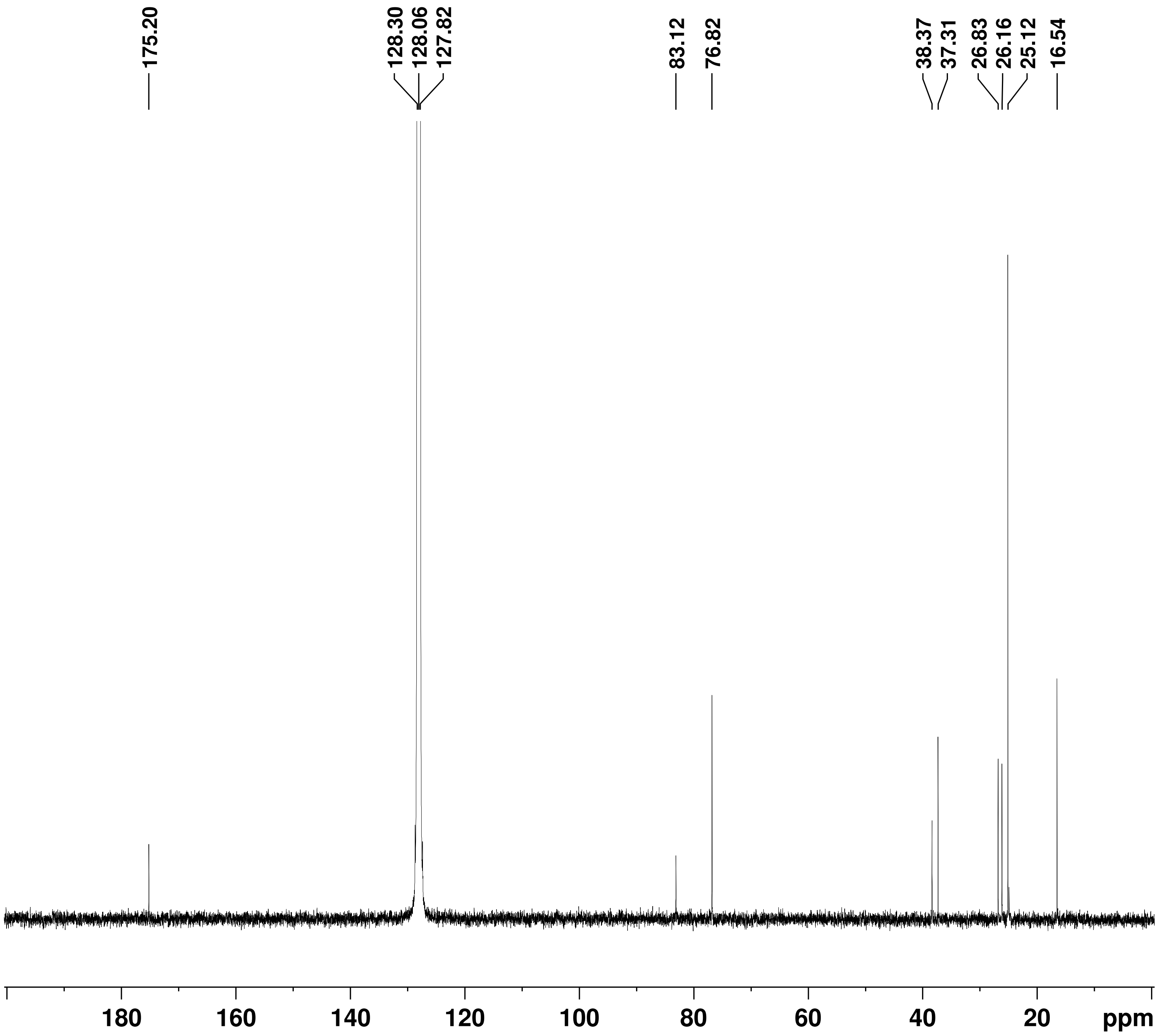

^{13}C spectrum of compound **24d**

400 MHz, C_6D_6

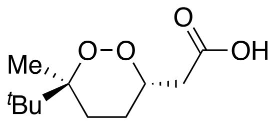

20d

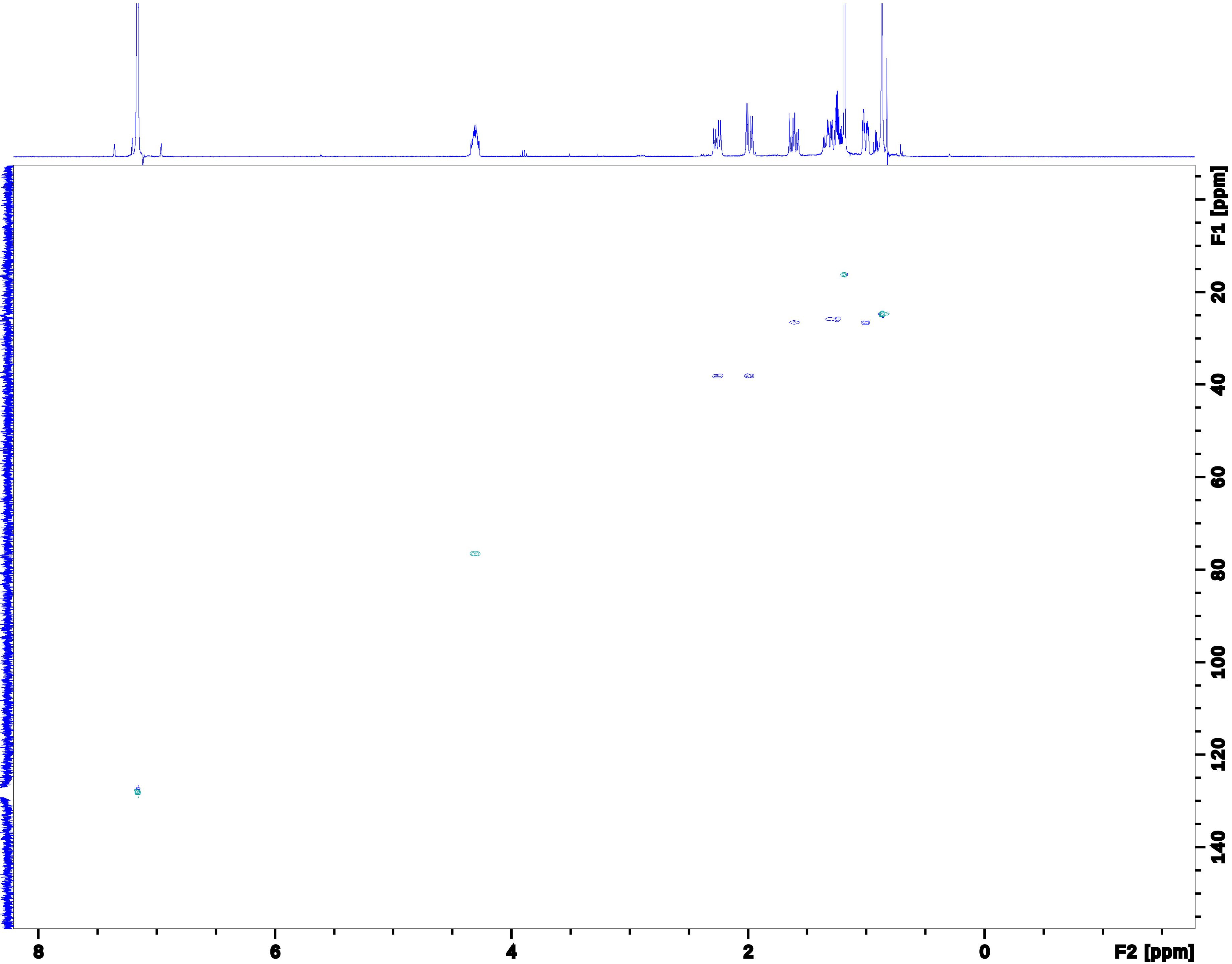

$^{1}H/^{13}C$ HSQC spectrum of compound **24d**
